# Chan-Evans-Lam Cu(II)-Catalyzed C–O Cross-Couplings: Broadening Synthetic Access to Functionalized Vinylic Ethers

**DOI:** 10.1021/acs.orglett.5c01966

**Published:** 2025-06-27

**Authors:** San L. Pham, Frank E. McDonald

**Affiliations:** Department of Chemistry, 1371 Emory University, Atlanta, Georgia 30322, United States

## Abstract

We report a general and effective Cu­(OAc)_2_-catalyzed C–O cross-coupling synthesis of di- and trisubstituted vinylic ethers, using a combination of *N*-isopropylimidazole ligand and dicumyl peroxide as stoichiometric oxidant. This method couples functionalized vinylic pinacolboronates with various 1° and 2° alcohols to provide single-step syntheses of novel and structurally complex vinylic ethers. The optimized conditions do not require excess alcohol reactant and substantially suppress competing side reactions.

## Graphical abstract

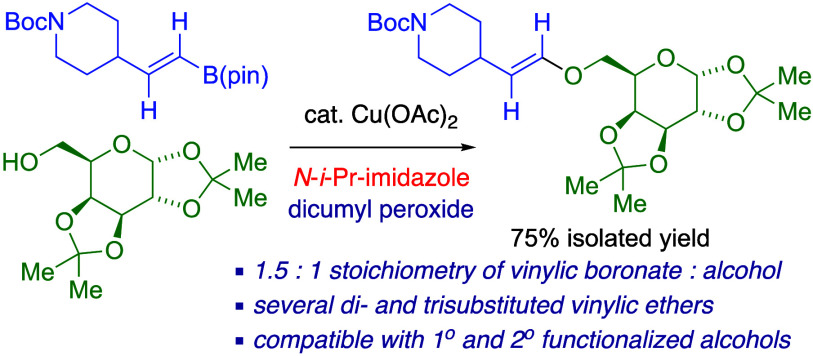

Vinylic ethers are electron-rich alkenes with many applications as synthetic intermediates.^1^ For instance, allylic vinylic ethers undergo Claisen rearrangements^2^ and are substrates for other cyclization and cycloaddition processes,^3^ including bioorthogonal reactions.^4^ Historically, the paucity of reliable synthetic methods for substituted vinylic ethers has limited their applications. C–O cross-couplings developed for aromatic synthons are less effective than those developed for structurally complex vinylic synthons and functionalized alcohols. We recently reported stereospecific C–O cross-couplings of vinylic halides with 1° and 2° alcohols, catalyzed by CuI and *trans*-*N*,*N*’-dimethylcyclohexyldiamine (CyDMEDA) (Figure 1a),^5^ and applied this method to nontraditional disaccharide synthesis through intermediates featuring structural complexity on both sides of the vinylic ether.^6^ As a complementary “Chan-Evans-Lam” approach,^7^ vinylic boron compounds undergo Cu­(II)-promoted oxidative C–O cross-couplings with simple alcohols (Figure 1b).^8–10^ However, the variation with vinylic pinacolboronates requires excess alcohol, often as solvent.^9^ Prior to this report, there were only two examples of Cu­(II)-promoted cross-couplings of vinylic boron reactants with 2° alcohols.^11^ Herein, we describe major enhancements in reaction conditions for Cu­(II)-catalyzed C–O cross-coupling synthesis of several di- and trisubstituted vinylic pinacolboronates (B­(pin)) with a range of 1° and 2° alcohols to synthesize novel and structurally complex vinylic ethers (Figure 1c).

**Figure 1:**
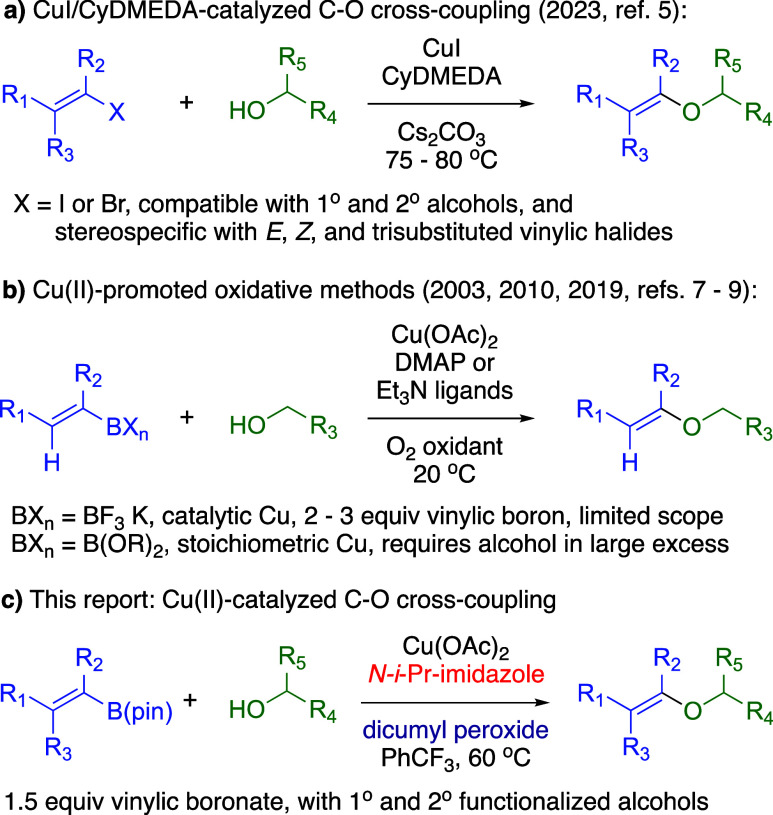
C–O cross-couplings with aliphatic alcohols.

Initial experiments with (*E*)-1-decenyl pinacolboronate (**1**) and galactose-derived 1° alcohol (**2**) required excess Cu­(OAc)_2_ and Et_3_N as ligand/base,^9^ giving vinylic ether **3** in only 23% yield. Side products incorporated acetate ligand, including unexpected acetate ester **4** (4%)^12^ and (*E*)-1-decenyl acetate (**5**, 24%).^13^ Mechanistic insights from other Cu­(II)-catalyzed C–O and C–N cross-couplings guided experiments to suppress side products.^14,15^ *N*-Isopropylimidazole (NPI) improved catalytic reactivity by favoring [Cu­(OAc)_2_]_2_ paddlewheel dissociation,^16^ giving a higher yield of vinylic ether **3** (50%), along with acetate ester **4** (12%). Dialkyl peroxides outperformed O_2_ as stoichiometric oxidant,^17,18^ and *tert*-butanol (*t*-BuOH) was a beneficial additive. Vinylic pinacolboronates and Cu­(OAc)_2_ were superior to other boron substituents or Cu sources (Tables S5, S7). ^1^H NMR time course experiments with di-*tert*-butyl peroxide (DTBP) revealed a ∼30 min induction period before significant reaction occurred (Figure S8). In contrast, the induction time with dicumyl peroxide (DCP) was less than 15 min, and reaction progress concluded within 35 min (Figure 2).

**Figure 2:**
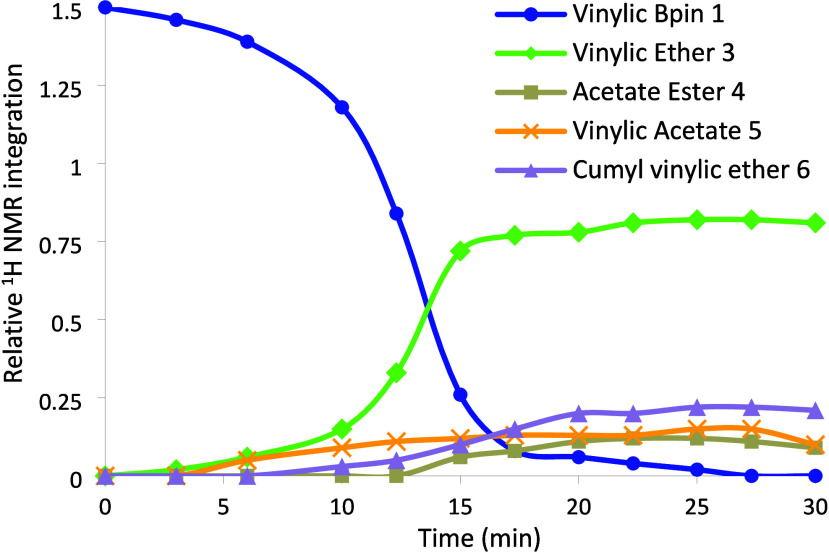
Time course of Cu­(OAc)_2_/NPI-catalyzed C–O cross-coupling of **1** (1.5 equiv) + **2** (1.0 equiv) with DCP oxidant. ^1^H NMR integrations relative to key resonances of alcohol **2**.

During the induction period, we detected both vinylic ether **3** and vinylic acetate **5**. However, the acetate ester **4** side product did not form in significant amounts until vinylic boronate **1** was consumed. Under optimized conditions, a preparative-scale reaction produced vinylic ether **3** in excellent yield from 1.5 equiv vinylic boronate **1** and alcohol **2** as limiting reactant, completed within 50 min (Figure 3). Vinylic ether **3** was easily separated from minor side products **4**–**7**.

**Figure 3:**
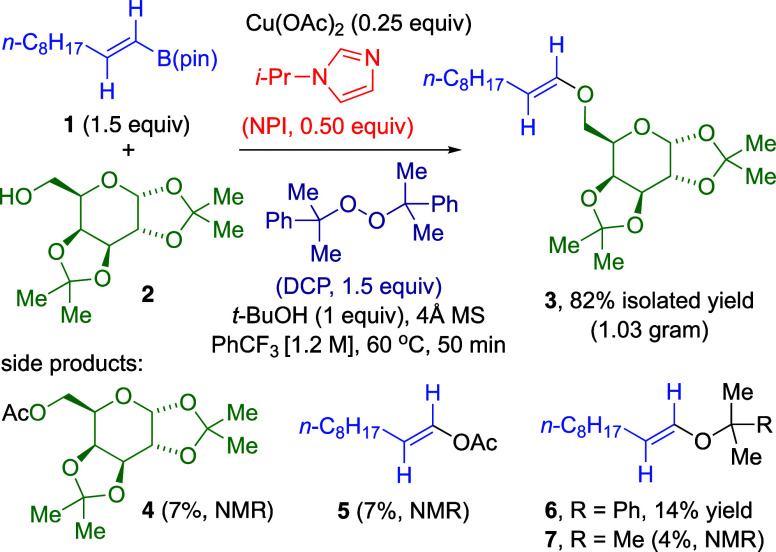
Optimized conditions for Cu­(OAc)_2_/NPI-catalyzed C–O cross-coupling with DCP oxidant.

These conditions have a broad scope with di- and trisubstituted vinylic boronates **8a**–**f**, including reactants with Boc- and Cbz-protected nitrogen. Cross-couplings with alcohol **2** produced vinylic ethers **10**–**14** (Figure 4). A (*Z*)-vinylic boronate **8f** required a higher temperature to produce (*Z*)-vinylic ether **15** in 52% isolated yield, with the acetate ester **4** side product in 30% NMR yield.

**Figure 4:**
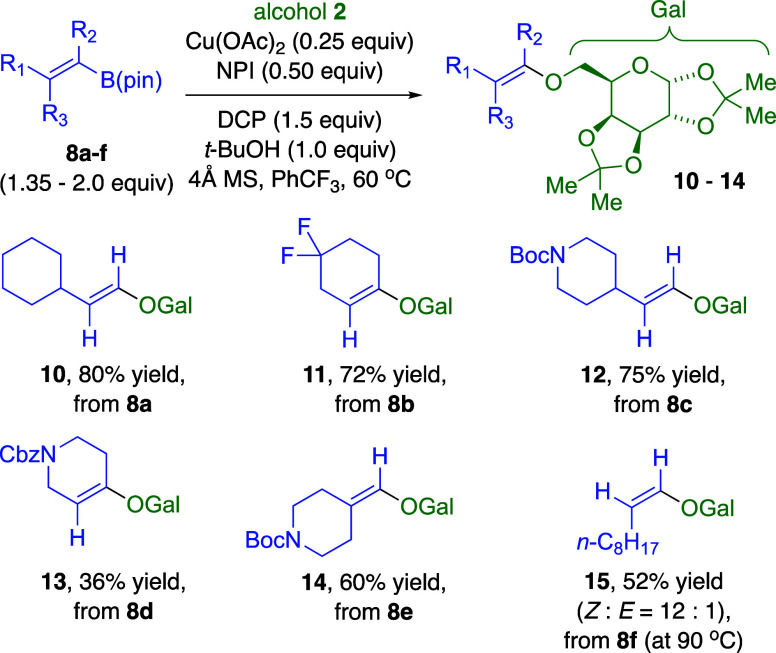
Vinylic BPin scope with alcohol **2** for Cu­(OAc)_2_/NPI-catalyzed C–O cross-coupling with DCP oxidant. Isolated yields based on alcohol **2** as limiting reactant.

We also demonstrated the scope with several 1° and 2° alcohols **9a**–**j** (Figure 5). *N*-Boc derivatives of d-serine and l-threonine methyl esters (**9a**, **9b**) gave the corresponding vinylic ethers **16** and **17**. We prepared compounds **18** and **19** by coupling vinylic boronate **8g** bearing an allylic oxygen substituent, with geraniol (**9c**) and tetrahydropyranol (**9d**), respectively. Cross-coupling reactions with other 2° alcohols cholesterol (**9e**), menthol (**9f**), and propargylic alcohol **9g** produced vinylic ethers **20**–**23** in yields comparable to CuI-CyDMEDA catalysis,^5^ but menthyl vinylic ethers **21** and **22** were accompanied by substantial side products **6** and **7**, revealing that tertiary alkoxides from the DCP oxidant and *tert*-butanol additive competed with menthol. Dichloropyridine-containing alcohol **9h** afforded vinylic ether **24** in excellent yield, and an aromatic iodide-benzylic alcohol **9i** was converted into vinylic ether **25** in moderate yield, consistent with other Cu­(II)-catalyzed cross-couplings with iodine-containing reactants.^7a,7b^ Aminoalcohol **9j** gave satisfactory generation of vinylic ether **26**. However, vinylic boronates with basic 3° amines **27** and **28** and shorter-chain aminoalcohol **29** were unreactive substrates, attributed to the proximity of the basic nitrogen to the reaction site. Cyclic aminoalcohol **30** was also unreactive, even though this alcohol was a viable substrate for CuI-DMEDA-catalyzed cross-coupling with vinylic iodides.^5^

**Figure 5:**
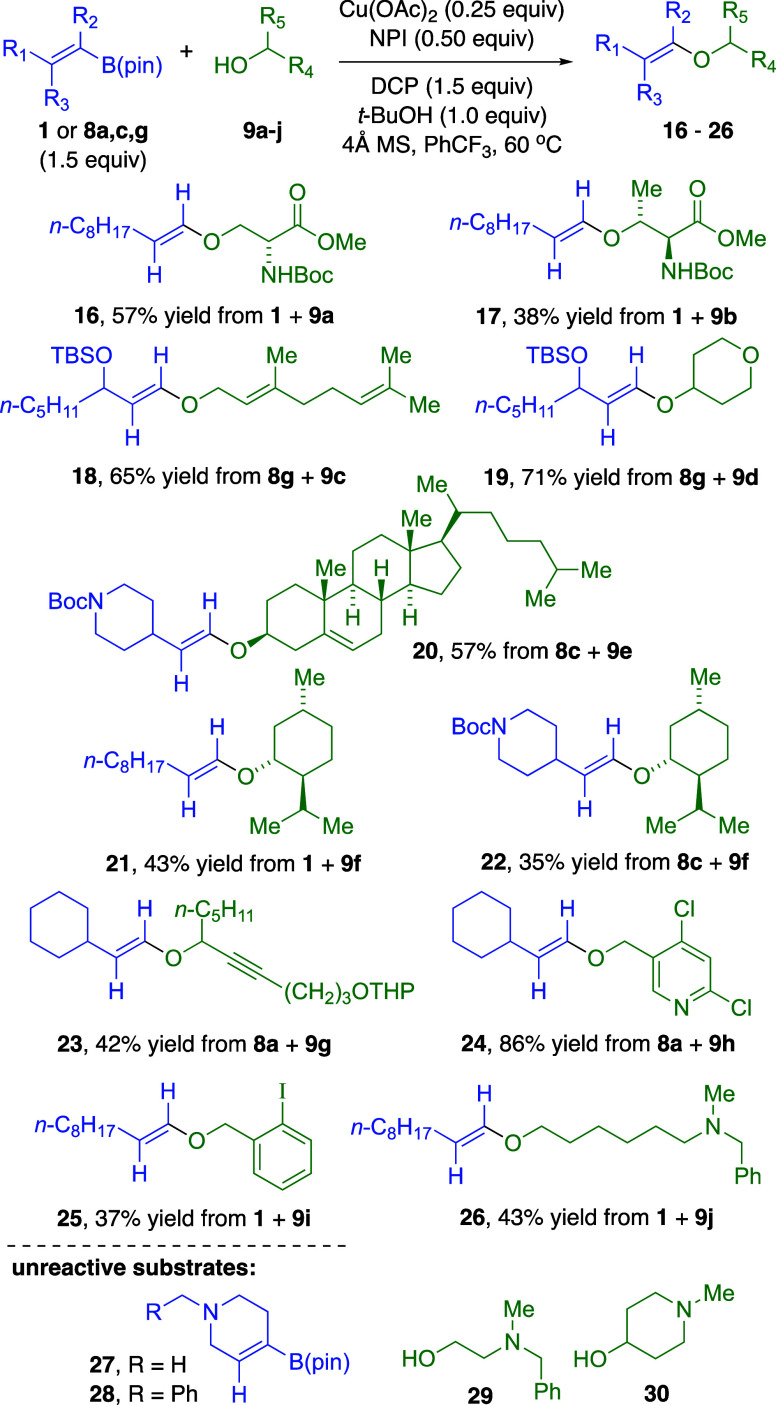
1° and 2° alcohol scope for Cu­(OAc)_2_/NPI-catalyzed C–O cross-coupling with DCP oxidant. Isolated yields based on alcohols **9a**–**j** as limiting reactant.

With galactose-derived 1° alcohol **2**, we tested this optimized Cu­(II)-catalyzed process in competition between vinylic boronate **1** and *p*-tolyl pinacolboronate **31** (eq 1). ^1^H NMR analysis of reaction mixture showed vinylic ether product **3** predominating over aryl ether **32**, along with side products **4**–**6**. Moreover, vinylic boronate **1** was ≥90% consumed, whereas only ∼20% of arylboronate **31** reacted.

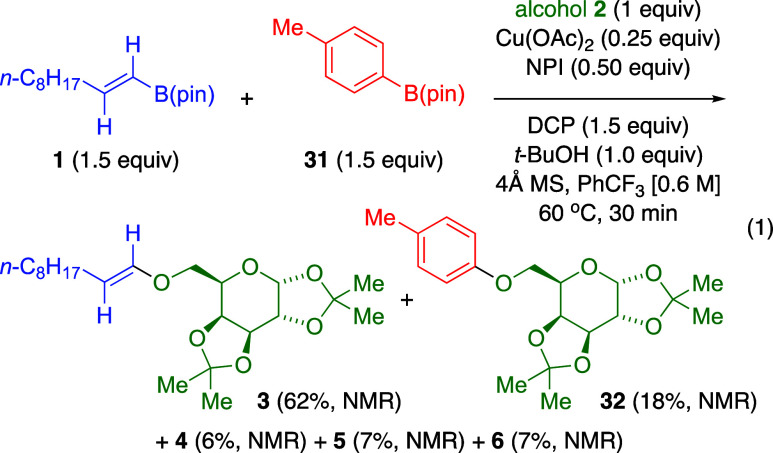

1



The canonical mechanism for Cu­(II)-catalyzed cross-couplings, established with arylboronate esters and methanol solvent, describes boron- to copper-transmetalation as turnover-limiting, with O_2_ as a four-electron oxidant, generating water as byproduct.^14^ Our results with vinylic boronates and alcohols as the limiting reactant and dialkyl peroxides as stoichiometric oxidants indicate 1) transmetalation is faster with vinylic boronates than with arylboronate **31**, so the turnover-limiting step may involve an alcohol reactant or Cu-coordinated alkoxide and 2) the dialkyl peroxide-dependent induction period may generate Cu­(II)-alkoxide^17,18^ as a more reactive catalyst than Cu­(OAc)_2_. The shorter induction period with DCP correlates with weaker O–O bond energy of DCP relative to DTBP.^17a^ Although dialkyl peroxide oxidants occasionally produce 3° vinylic ether side products **6** and **7**, these oxidants do not generate water.^7b,14c,19^ Initial transmetalation from Cu­(OAc)_2_ precatalyst under anhydrous conditions may generate AcO-Bpin byproduct^20^ for competing *O*-acetylation of alcohol, perhaps with NPI as a transacylation catalyst.

In conclusion, this combination of NPI ligand and DCP oxidants is highly effective for synthesizing di- and trisubstituted vinylic ethers from several types of vinylic boronates and 1° and 2° alcohols. The reaction conditions do not require excess alcohol reactant, and with most 1° alcohols and less hindered 2° alcohols, side products are minimal relative to vinylic ether products. This Cu­(II)-catalyzed method from vinylic boronates, complementing the Cu­(I)-CyDMEDA process from vinylic halides,^5^ enables another pathway for synthetic access to novel vinylic ethers. The combination of NPI ligand and DCP oxidant may also benefit other Cu­(II)-catalyzed reactions.^21^

## Supplementary Material





## Data Availability

The data underlying this study are available in the published article and its .

## Notes

Detailed experimental procedures, compound characterization, optimization experiments, and copies of ^1^H, ^13^C­{^1^H}, ^19^F, and 2D NMR spectra (COSY, HSQC, HMBC) for new compounds (PDF)
FAIR data, including the primary NMR FID files, for compounds **3**–**7**, **10**–**26**, **32**, and **S2** (ZIP)
