# Supplementary material for: Chan-Evans-Lam Cu(II)-Catalyzed C–O Cross-Couplings: Broadening Synthetic Access to Functionalized Vinylic Ethers
Source: Org Lett. 2025 Jun 27;27(27):7326–30. doi: 10.1021/acs.orglett.5c01966 (PMC12261398; doi:10.1021/acs.orglett.5c01966)

Chan-Evans-Lam Cu(II)-Catalyzed C-O Cross-Couplings: Broadening Synthetic Access to Functionalized Vinylic Ethers

San L. Pham and Frank E. McDonald*

Department of Chemistry, Emory University, Atlanta, GA, United States 30322

*Corresponding author: fmcdona@emory.edu

Supporting Information: Detailed experimental procedures, compound characterization, optimization experiments, and copies of ^{1}H, $^{13}C\{^{1}H\}$, ^{19}F, and 2D NMR spectra (COSY, HSQC, HMBC) for new compounds

A. General Experimental S-2
B. Procedures for Cu(II)-catalyzed C-O cross-coupling reactions S-3
1. Optimized protocol for synthesizing vinylic ether **3**, with DCP as oxidant
2. Optimized protocol for large-scale synthesis of vinylic ether **3** (3.1 mmol)
3. Notes on preliminary scale-up experiments
4. ^{1}H NMR yield determination from crude reaction mixtures
5. Species profile of test reactions between vinylic pinacolboronate **1** and alcohol **2**

C. Reactant scope S-13
1. Representative general procedures
2. Vinylic boronate scope, giving vinylic ethers **10** - **15**
3. Alcohol scope, giving vinylic ethers **16** - **26**

D. Optimization studies S-33
1. Initial extension of previously published Chan-Evans-Lam conditions (ref. 12)
2. Mechanistic hypotheses for optimization strategies
3. Reaction optimization studies (0.31 mmol scale, 0.6 M reaction concentration based on alcohol **2** as limiting reagent)
4. Other reaction optimization studies (0.31 mmol scale, 0.6 M reaction concentration based on limiting reagent)

E. Syntheses of other vinylic borons S-45
F. Mechanistic experiments S-47
1. Time studies by ^{1}H NMR spectroscopy of aliquots
2. Competition study with *p*-tolyl pinacolboronate **31** with optimized conditions
3. Control experiment with DTBP oxidant and *t*-BuOH, without 1° or 2° alcohol reactant
4. Control experiment without vinylic pinacolboronate **1**

G. Mechanistic hypothesis for optimized procedure S-55
H. Incompatible Substrates S-56
I. References Cited S-57
J. Copies of NMR spectra for new compounds S-60

A. General Experimental

^1H and ^{13}C NMR spectra were recorded with Varian AVIII 400, Bruker NEO 400, and AVANCE 600 equipped with a cryogen probe. NMR spectra were measured from solutions in deuterated chloroform ($CDCl_3$) or deuterated benzene (C_6D_6), with chemical shifts in parts per million (ppm) referenced to residual chloroform resonances (7.27 ppm for ^1H NMR and 77.23 ppm for ^{13}C NMR) or residual benzene resonances (7.16 ppm for ^1H NMR and 128.06 ppm for ^{13}C NMR). Structural assignments were made with additional information from gCOSY, gHSQC, and gHMBC experiments. Deuterated chloroform was neutralized with anhydrous K_2CO_3 and kept dry with anhydrous Na_2SO_4 to prevent acid-catalyzed hydrolysis of vinylic ethers. Abbreviations for signal coupling are: s, singlet; d, doublet; t, triplet; q, quartet; dd, doublet of doublet; ddd, doublet of doublet of doublet; dt, doublet of triplet; m, multiplet. Mass spectra (high resolution ESI and APCI) were recorded with a Thermo LTQ FTMS Mass spectrometer. Optical rotations were measured using a Perkin-Elmer 341 polarimeter (concentration in g/100 mL). Melting points were measured using DigiMelt MPA 160 apparatus from SRS (Stanford Research System). Thin layer chromatography (TLC) was performed on pre-coated aluminum-backed plates purchased from Silicycle (silica gel 60F254; 0.25 mm thickness), visualized with ultraviolet light and/or stained with phosphomolybdic acid / ethanol stain (PMA). Flash column chromatography was conducted with silica gel 60 (230-400 mesh ASTM) from Silicycle, Sigma Aldrich.

All reactions were conducted with anhydrous solvents in oven-dried and argon-charged vials or flasks, unless otherwise stated. Reactions conducted under inert argon atmosphere were conducted by sealing reaction vessel with a septum and replacing the atmosphere with argon by sparging with a stream of argon. After sparging, reactions set up in ≤ 20 mL vials were capped with solid screw-on caps and sealed with Teflon tape and electrical tape. All other reactions were conducted under a positive stream of argon in the reaction headspace. Reactants were used as received from commercial suppliers without prior purification, as were solvents used for extractions and chromatographic separations. All chemicals were purchased from Sigma Aldrich, Oakwood Chemical, TCI Chemicals, Ambeed, and Combi-blocks. $Cu(OAc)_2$ (99.99% trace metal basis, anhydrous, catalog number: 229601) was purchased from Sigma Aldrich and stored at room temperature. Anhydrous trifluorotoluene ($PhCF_3$) was obtained from Sigma Aldrich (catalog number: 547948). Anhydrous acetonitrile (MeCN), dichloromethane (DCM), and 1,2-dichloroethane (DCE) were purchased from Acros Organic. 4Å molecular sieves (4Å MS) were obtained from Sigma Aldrich, stored in 180 °C oven, and re-activated prior to use by flame-drying. Reactions conducted above room temperature were heated: 1) in an external silicone oil bath within an evaporating dish placed on top of a dual stirrer / hot plate (Ceramag Midi), magnetically stirred, with a thermometer measuring the oil bath temperature, or 2) in a 9-vial capacity aluminum heating block (Pie-Blocki CG-1991-P-13*), monitoring temperature with a temperature probe in a 4 mL vial of silicone oil (wrapped with aluminum foil), in the heating block.

Compounds **1**,[1] **8a**,[1] **8c**,[2] **8f**,[33] **8g**,[1,3] **9g**,[4] **9j**,[5] (*E*)-dec-1-en-1-yl trifluoroborate potassium salt,[6] and *p*-tolyl pinacolboronate **31**[7] were synthesized according to published protocols. All other vinylic pinacolboronate and alcohol reactants were purchased and used as received from Ambeed, Sigma Aldrich, and Combi-Blocks without prior purification.

Vinylic ethers are susceptible to hydrolysis, due to the mildly acidic nature of silica gel used for flash column chromatography and traces of DCl in $CDCl_3$. Vinylic ethers were purified by flash column chromatography with silica gel pre-treated with 2% triethylamine (Et_3N) in hexanes.

B. Procedures for Cu(II)-catalyzed C-O cross-coupling reactions

1. Optimized protocol for synthesizing vinylic ether 3, with DCP as oxidant:

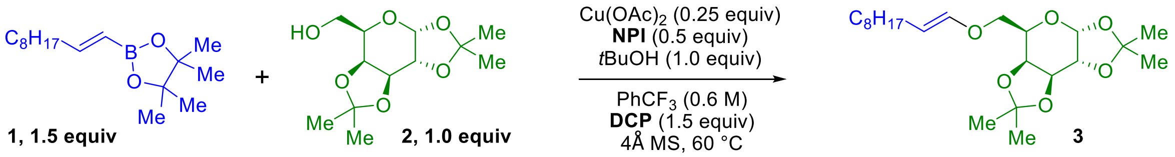

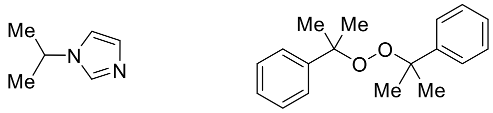

N-isopropylimidazole (NPI) **Dicumyl peroxide (DCP)**

Reaction set-up protocol: A 4 mL reaction vial was charged with a stir bar and 110-115 mg of 4Å molecular sieves (MS), and flame-dried, with cooling in air. Alcohol **2** (81 mg, 0.31 mmol, 1.0 equiv), *N*-isopropylimidazole (NPI, 18 µL, 0.16 mmol, 0.50 equiv), *t*-butanol (*t*-BuOH, 30 µL, 0.31 mmol, 1.0 equiv), dicumyl peroxide (DCP, 126 mg, 0.47 mmol, 1.5 equiv), copper(II) acetate ($Cu(OAc)_2$, 14 mg, 0.08 mmol, 0.25 equiv), and vinylic pinacolboronate **1** (124 mg, 0.47 mmol, 1.5 equiv) were added sequentially to the reaction vial. The vial was sealed with a septum cap and sparged with argon. Trifluorotoluene ($PhCF_3$, 0.5 mL, 0.6 M based on mmol of **2**) was added to the reaction vial and the mixture was further purged with argon for 3-5 minutes. The septum cap was replaced with a solid cap and the vial was sealed with Teflon and electrical tape. The vial was stirred at 60 °C on a pre-heated heating block with an internal temperature probe. The reaction mixture changed from a deep blue mixture to a dark green mixture after 10-15 minutes of stirring.

Standard work-up protocol: After the reaction stirred for 35 minutes (note: also repeated with 1-hour and 18-hour reaction times), the reaction vial was removed from the heating block and cooled to room temperature. The contents were diluted with DCM, the product mixture was transferred to a 20 mL vial using DCM to rinse, which was concentrated by rotary evaporation. A ^1H NMR spectrum of the crude product mixture in C_6D_6 was taken for NMR yield analysis (details discussed in section 1.4, S-5).

Purification protocol: The crude product mixture (dry-loaded on 1.1 g of Celite) was purified by column chromatography in silica gel treated with 2% Et_3N in hexanes. The product fractions were eluted with a gradient of 98:2 – 97:3 – 95:5 – 92:8 – 90:10 – 80:20 hexanes : ethyl acetate (EtOAc), with fractions checked by TLC in 90:10 hexanes/EtOAc and visualized with PMA stain. The appropriate fractions were concentrated by rotary evaporation, and high-vacuum evaporation overnight. Vinylic ether **3** was obtained as a pale-yellow oil (84% NMR yield; 99 mg, 80% isolated yield). Similar yields of vinylic ether **3** were obtained from experiments conducted at similar scale (~0.31 mmol scale) for 1-hour reaction times (87% NMR yield; 102 mg, 82% isolated yield) or for 18-hour reaction times (85% NMR yield; 104 mg, 84% isolated yield).

<u>Note:</u> Side products were usually observed in crude product mixtures, but this purification protocol effectively separated the desired vinylic ether **3** from side products, therefore isolated yields of side products were generally not reported. Relatively pure samples of side products were obtained for characterization in some exploratory experiments, when substantial formation was observed in ^1H NMR spectra of crude reaction mixtures.

2. Optimized protocol for large-scale synthesis of vinylic ether 3 (3.1 mmol):

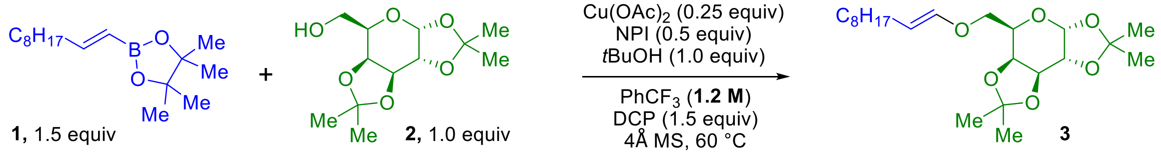

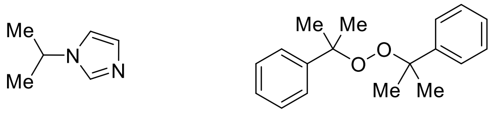

N-isopropylimidazole (NPI) **Dicumyl peroxide (DCP)**

Experimental protocol: An oven-dried 25 mL round bottom flask (RBF), charged with a stir bar (25 mm x 7.9 mm) and 1.15 g of activated 4Å MS, was flame-dried, with cooling in air. Alcohol **2** (810 mg, 3.1 mmol, 1.0 equiv), NPI (180 mL, 1.6 mmol, 0.5 equiv, added by *p*200 micropipette), *t*-BuOH (300 mL, 3.1 mmol, 1.0 equiv, added by *p*200 micropipette), DCP (1.26 g, 4.7 mmol, 1.5 equiv), $Cu(OAc)_2$ (141 mg, 0.8 mmol, 0.25 equiv), and vinylic pinacolboronate **1** (1.24 g, 4.7 mmol, 1.5 equiv) were added sequentially to the flask under air. The reaction mixture was placed under argon atmosphere by sparging, followed by addition of $PhCF_3$ (2.5 mL, 1.2 M based on alcohol **2**). The mixture was stirred at room temperature and further purged with argon for 10 minutes, which gave a deep blue mixture. _Note_: we took extra care to rinse and mix all the reagents into the reaction solvent. The mixture was added to an oil bath preheated to 64 °C (the reaction mixture's internal temperature was monitored as 60 °C with an internal thermometer probe when the oil bath was heated at 64 °C). The reaction mixture was stirred at 350 revolutions per minute (rpm) under argon for 50 minutes. The reaction mixture was monitored by TLC (90:10 hexanes/EtOAc, stained with PMA) at 25-minute mark to show almost full conversion of vinylic pinacolboronate **1**. After the reaction was complete, the mixture was cooled to room temperature, diluted and transferred to a 250-mL RBF with DCM, and concentrated by rotary evaporation to give a dark green-brownish oil. ^1H NMR analysis of the crude product mixture in C_6D_6 revealed full conversion of vinylic pinacolboronate **1**, ~82% NMR yield of vinylic ether **3**, ~6-8% NMR yield of acetate ester **4**, ~14% NMR yield of cumyl vinylic ether **6**, ~7% NMR yield of vinylic acetate **5**, ~4% NMR yield of *t*-butyl vinylic ether **7**, and traces of 1-decene (**S1**). The crude product mixture was purified by column chromatography in silica gel pre-treated with 2% Et_3N in hexanes (by wet loading with minimal amount of 2% Et_3N in hexanes). The product fractions were eluted with a gradient of 100:0 – 98:2 – 97:3 hexanes/EtOAc. The appropriate fractions were checked with TLC (90:10 hexanes/EtOAc, stained with PMA), and concentrated using rotary evaporation and high-vacuum evaporation. Cumyl vinylic ether **6** was collected as a pale-yellow clear oil (176 mg, 14% isolated yield). Vinylic ether **3** was more polar than cumyl vinylic ether **6** and easily separated as a light-yellow clear oil (1.03 g, 82% isolated yield).

3. Notes on preliminary scale-up experiments:

Prior to the optimized conditions above, several different scale-up experiments were attempted. With an oil bath heated to 60 °C, the internal temperature only reached between 55 – 57 °C within 10 minutes and gave low conversion of vinylic pinacolboronate **1**. On 3.1 mmol scale, with 5 mL $PhCF_3$ (0.6 M concentration), conversion of vinylic pinacolboronate **1** was also low. However, increasing the reaction concentration to 1.2 M, and heating the reaction mixture in a 65 °C oil bath with vigorous stirring gave reproducible results.

4. ^{1}H NMR yield determination from crude reaction mixtures:

Most ^{1}H NMR spectra of crude reaction mixtures were analyzed in C_6D_6 as the deuterated solvent. Superimposed and stacked spectra of crude mixture showed the anomeric protons of the galactose-derived alcohol **2** (5.49 ppm), vinylic ether **3** (5.54 ppm), and acetate ester **4** (5.48 ppm) (**Figure S1**). Due to the high molecular weights of compounds **2**, **3** and **4**, we assumed that these materials were not lost in the standard work-up protocol. Thus, these distinct ^{1}H NMR resonances were used to estimate NMR yields of identifiable species derived from alcohol **2** in the crude product mixture.

Specifically, for vinylic ether **3** and acetate ester **4** derived from alcohol **2** as limiting reactant, NMR yields were estimated based on the relative integrations of their anomeric protons. Hence, in this example (**Figure S2**), the NMR yield for vinylic ether **3** was 70%, and for acetate ester **4** was 25%.

For side products "A" derived from vinylic pinacolboronate **1** (A = vinylic acetate **5**, cumyl vinylic ether **6**, *tert*-butyl vinylic ether **7**, or 1-decene **S1**), their respective NMR yields were back-calculated from their relative integrations using the formula below:

$$\%NMR\ yield\ of\ A = \frac{NMR\ integration\ of\ A\ \times mmol\ alcohol\ \mathbf{2}}{mmol\ of\ vinylic\ Bpin\ \mathbf{1}} \times 100\%$$

For side product diene **S2**, NMR yields were calculated using the following formula, to account for the 2 : 1 ratio of reactant vinylic boronate **1** : side product diene **S2**, also considering that NMR integration of the diene signature peaks represented 2 protons:

$$\%NMR\ yield\ of\ diene\ \mathbf{S2} = \frac{(NMR\ integration\ of\ \mathbf{S2} \div 2)\ \times mmol\ alcohol\ \mathbf{2}}{(mmol\ of\ vinylic\ Bpin\ \mathbf{1} \div 2)} \times 100\%$$

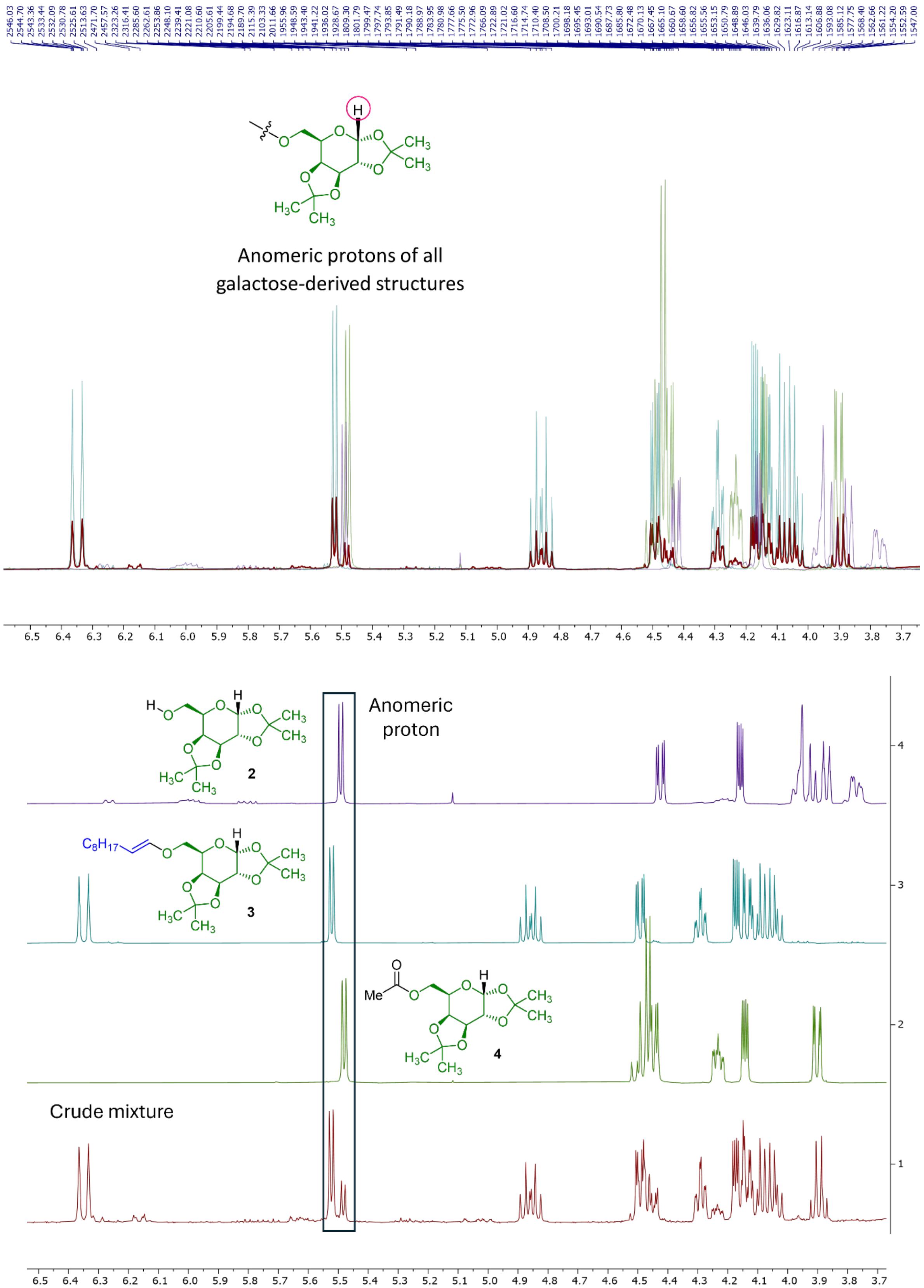

Figure S1. ^{1}H NMR spectra (in C_6D_6) overlaying compounds **2**, **3**, and **4** (top); pure compounds **2**, **3**, and **4**; and crude product mixture (**Table S1**, entry 4) (bottom)

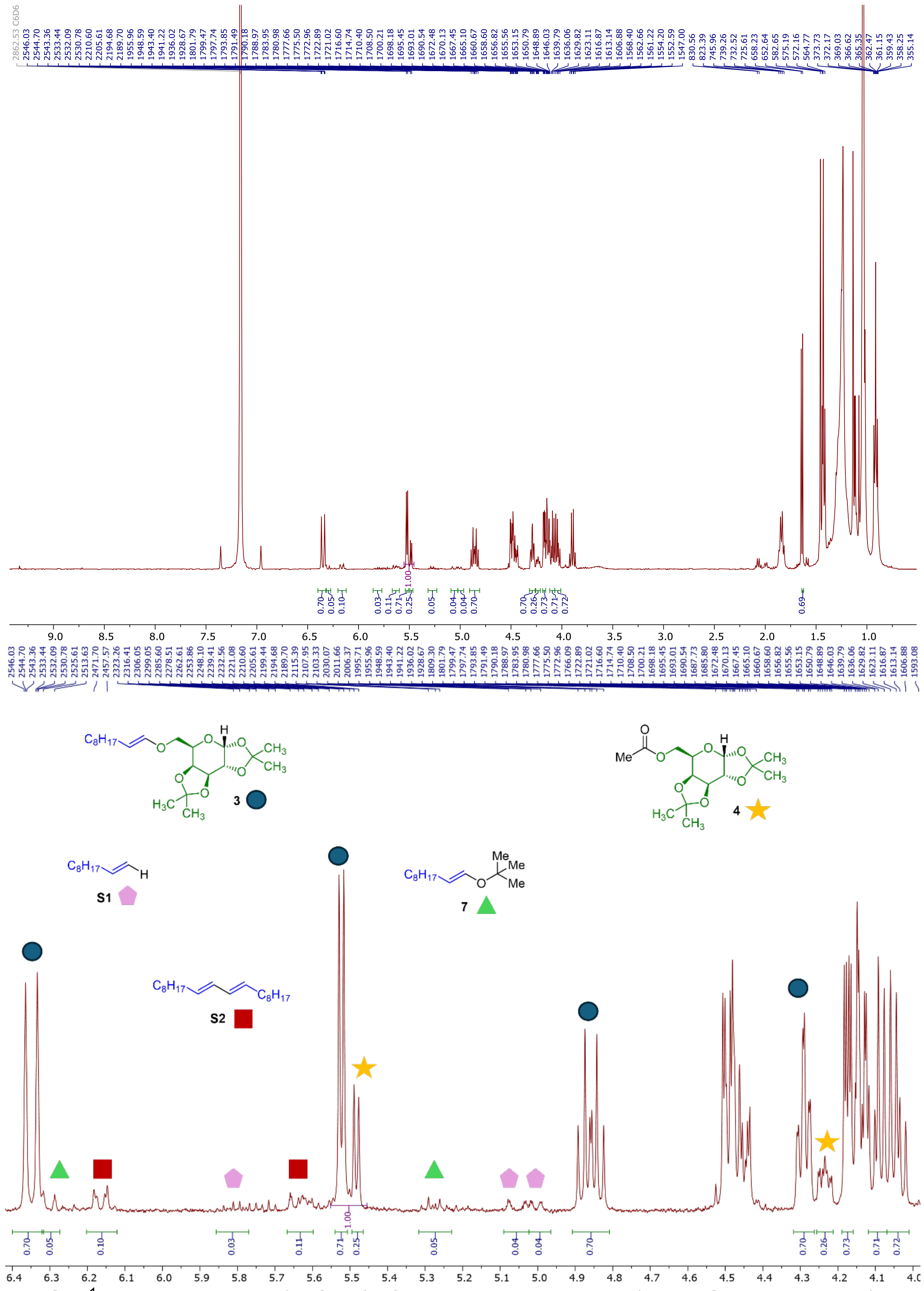

Figure S2. ^{1}H NMR spectrum (in C_6D_6) of crude product mixture (**Table S1, entry 4**; top). The expansion (bottom) shows signature peaks used for NMR yield determination.

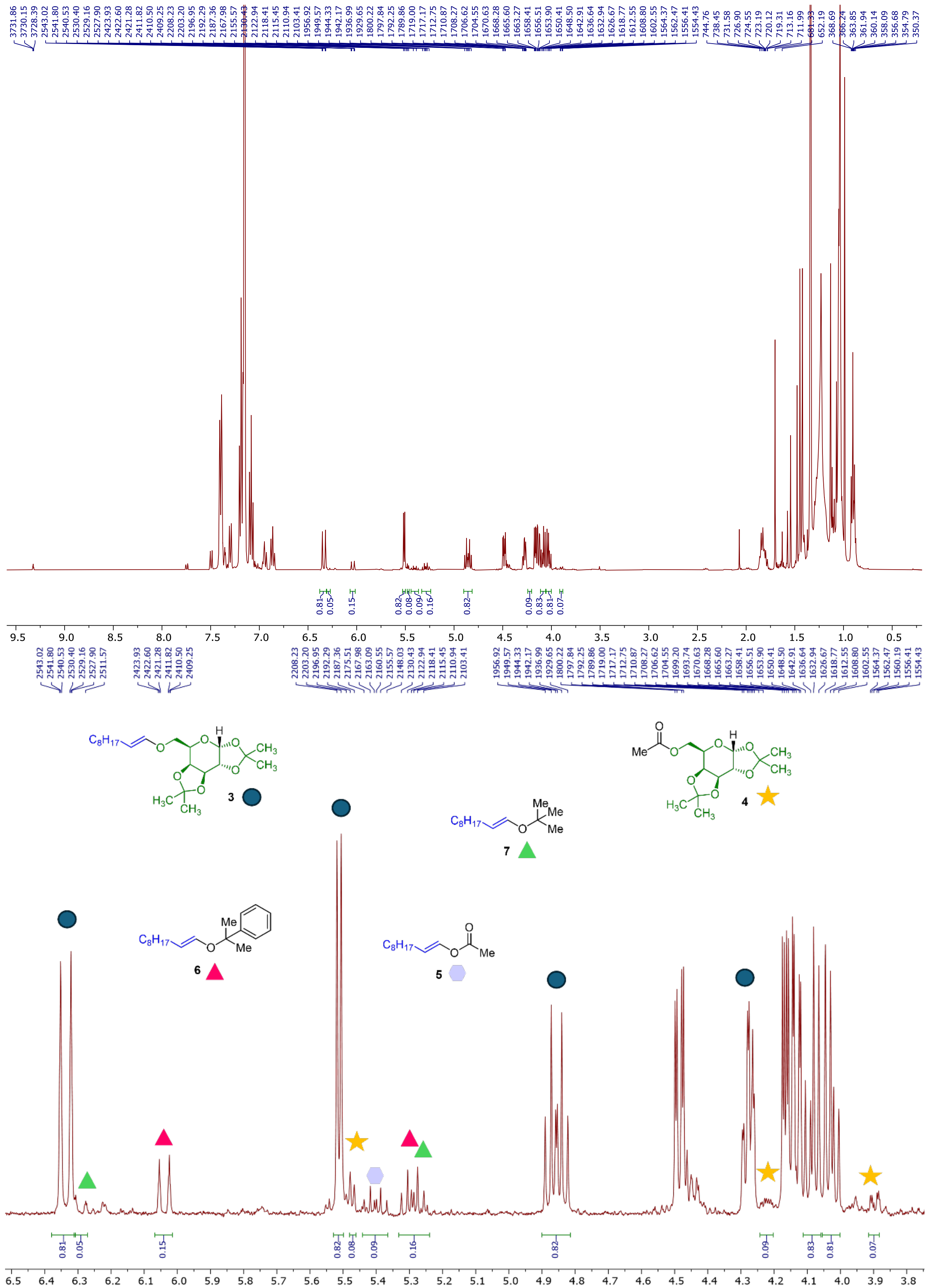

Figure S3. ^{1}H NMR spectrum (in C_6D_6) of crude product mixture (**Table S1, entry 6**; top). The expansion (bottom) shows signature peaks used for NMR yield determination.

5. Species profile of test reactions between vinylic pinacolboronate 1 and alcohol 2:

a. *Structures identified in crude reaction mixtures:*

Reactants:

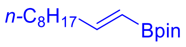

1

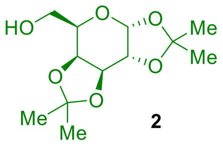

2

Major products:

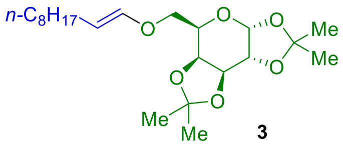

3

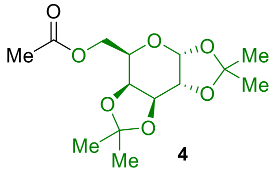

4

Minor products:

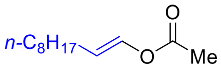

5, mostly suppressed

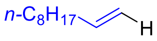

S1, mostly suppressed

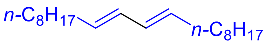

S2, up to 10% when using vinylic Bpin in excess

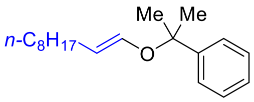

6, up to 15% found when using DCP as oxidant

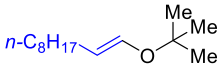

7, traces found for DTBP conditions

**also observed in DCP conditions due to tert-butanol additives*

b. *Characterizations of the major product and minor side products:*

(*E*)-Vinylic ether 3:

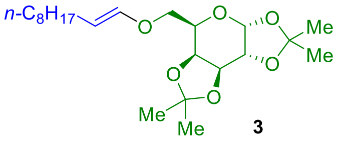

3

Physical appearance: clear pale-yellow oil

^{1}H NMR spectrum of vinylic ether **3** recorded in $CDCl_3$ matched previously published data.[8]

^{1}H NMR (400 MHz, $CDCl_3$) δ 6.26 (dt, *J* = 12.6, 1.3 Hz, 1H), 5.54 (d, *J* = 5.0 Hz, 1H), 4.80 (dt, *J* = 12.6, 7.4 Hz, 1H), 4.61 (dd, *J* = 7.9, 2.4 Hz, 1H), 4.32 (dd, *J* = 5.0, 2.4 Hz, 1H), 4.26 (dd, *J* = 7.9, 1.9 Hz, 1H), 4.02 (ddd, *J* = 7.2, 5.6, 1.9 Hz, 1H), 3.84 (dd, *J* = 10.5, 5.8 Hz, 1H), 3.79 (dd, *J* = 10.4, 7.0 Hz, 1H), 1.94 – 1.84 (m, 2H), 1.53 (s, 3H), 1.45 (s, 3H), 1.34 (s, 3H), 1.33 (s, 3H), 1.31 – 1.22 (m, 12H), 0.90 – 0.84 (t, *J* = 6.5 Hz, 3H).

^{1}H NMR spectrum was also recorded in C_6D_6 for direct comparison with crude NMR spectra.

^{1}H NMR (400 MHz, C_6D_6) δ 6.34 (dt, *J* = 12.6, 1.3 Hz, 1H), 5.51 (d, *J* = 5.0 Hz, 1H), 4.85 (dt, *J* = 12.5, 7.3 Hz, 1H), 4.48 (dd, *J* = 8.0, 2.4 Hz, 1H), 4.28 (td, *J* = 6.2, 1.9 Hz, 1H), 4.16 (dd, *J* = 5.0, 2.3 Hz, 1H), 4.13 (dd, *J* = 8.0, 1.9 Hz, 1H), 4.09 (dd, *J* = 10.0, 6.3 Hz, 1H), 4.03 (dd, *J* = 10.0, 6.3 Hz, 1H), 1.83 (qd, *J* = 7.3, 1.4 Hz, 2H), 1.45 (s, 3H), 1.42 (s, 3H), 1.32 – 1.19 (m, 12H), 1.13 (s, 3H), 1.03 (s, 3H), 0.91 (t, *J* = 6.9 Hz, 3H).

Acetate ester 4:

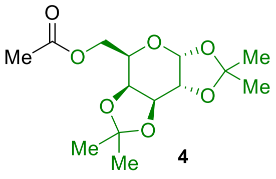

4

Physical appearance: yellow oil

^{1}H NMR spectrum of acetate ester **4** taken in $CDCl_3$ matched previously published data.[9]

^{1}H NMR (400 MHz, $CDCl_3$) δ 5.54 (d, *J* = 5.0 Hz, 1H), 4.62 (dd, *J* = 7.9, 2.5 Hz, 1H), 4.33 (dd, *J* = 5.0, 2.5 Hz, 1H), 4.29 (dd, *J* = 11.6, 4.7 Hz, 1H), 4.24 (dd, *J* = 7.9, 1.9 Hz, 1H), 4.18 (dd, *J* = 11.6, 7.7 Hz, 1H), 4.02 (ddd, *J* = 7.7, 4.6, 1.8 Hz, 1H), 2.09 (s, 3H), 1.52 (s, 3H), 1.47 – 1.43 (m, 3H), 1.36 – 1.31 (m, 6H).

^{1}H NMR spectrum was also measured in C_6D_6 for direct comparison with crude NMR spectra.

^{1}H NMR (400 MHz, C_6D_6) δ 5.48 (d, *J* = 5.0 Hz, 1H), 4.54 – 4.42 (m, 3H), 4.23 (ddd, *J* = 7.3, 4.9, 1.9 Hz, 1H), 4.14 (dd, *J* = 5.0, 2.5 Hz, 1H), 3.90 (dd, *J* = 7.9, 2.0 Hz, 1H), 1.64 (s, 3H), 1.44 (s, 3H), 1.41 (s, 3H), 1.11 (s, 3H), 1.02 (s, 3H).

(*E*)-Vinylic acetate 5:

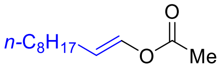

5, mostly suppressed

Compound **5** was volatile when attempting to remove all solvent traces under high vacuum. Hence, ^{1}H NMR characterization (in C_6D_6) was conducted with a 15 mg sample of vinylic acetate **5** (in a mixture with ~30% hexanes from column chromatography, containing ~10% 1-decanal from hydrolysis of vinylic ether compounds).

Physical appearance: clear colorless oil

^{1}H NMR (400 MHz, C_6D_6) δ, 7.35 (dt, *J* = 12.5, 1.5 Hz, 1H), 5.41 (dt, *J* = 12.5, 7.5 Hz, 1H), 1.80 (apparent qd, *J* = 7.5, 1.5 Hz, 2H), 1.58 (s, 3H), 1.35 – 1.14 (m, 18H, overlapping with hexanes and 1-decanal), 0.91 (t, *J* = 6.8 Hz, 3H, overlapping with hexanes and 1-decanal).

$^{13}C\{^{1}H\}$ NMR (101 MHz, C_6D_6) δ 167.5, 136.1, 114.5, 32.3, 30.0, 29.8, 29.7, 29.4, 27.6, 23.1, 20.1, 14.4.

HRMS (APCI) *m/z*: $[M+H]^+$ Calcd for $C_{12}H_{23}O_2$ 199.1693; Found 199.1689.

Identifiable NMR signals of 1-decanal impurity: **^{1}H NMR (400 MHz, C_6D_6)** δ 9.33 (t, *J* = 1.7 Hz, 1H). **$^{13}C\{^{1}H\}$ NMR (101 MHz, C_6D_6)** δ 200.7.

1-Decene (S1):

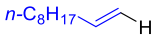

S1, mostly suppressed

Signature alkenyl proton peaks of compound **S1** detected in crude ^{1}H NMR spectrum in $CDCl_3$ (matched literature values for 1-decene[10]):

^{1}H NMR (400 MHz, $CDCl_3$) δ 5.84 (ddt, *J* = 16.9, 10.2, 6.7 Hz, 1H), 5.04 (dq, *J* = 17.1, 1.6 Hz, 1H), 4.98 (ddt, *J* = 10.2, 2.2, 1.2 Hz, 1H).

Signature alkenyl proton peaks (with similar *J*-coupling constants but at different chemical shifts) detected in crude ^{1}H NMR spectrum in C_6D_6:

^{1}H NMR (400 MHz, C_6D_6) δ 5.79 (ddt, *J* = 16.9, 10.1, 6.7 Hz, 1H), 5.04 (dq, *J* = 17.1, 1.7 Hz, 1H), 4.99 (ddt, *J* = 10.2, 2.3, 1.2 Hz, 1H).

(*E,E*) Diene S2:

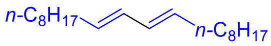

S2, ~5-10% when using vinylic Bpin in excess

Compound **S2** was obtained (~14 mg, ~20% isolated yield, containing trace hexanes from column chromatography) from a substrate scope exploratory experiment with (-)-menthol (**9c**). ^{1}H NMR characterization of diene **S2** was measured in C_6D_6. The coupling pattern of the alkenyl protons and the allylic protons matched published ^{1}H NMR spectra of related symmetric dienes such as (*E*,*E*)-7,9-hexadecadiene (reported in $CDCl_3$).[11] <u>*Note*</u>: Diene **S2** underwent oxidative decomposition after 6 months at ambient atmosphere and temperature.

Physical appearance: clear pale-yellow oil

^{1}H NMR (400 MHz, C_6D_6) δ 6.20 – 6.09 (m, 2H), 5.71 – 5.52 (m, 2H), 2.06 (q, *J* = 7.1 Hz, 4H), 1.36 (m, 5H), 1.27 (m, 19H), 0.90 (t, *J* = 6.8 Hz, 6H).

(*E*)-Cumyl vinylic ether 6:

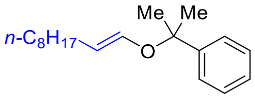

6, ~10-15% found when using DCP as oxidant

A relatively pure sample (42 mg) of cumyl vinyl ether **6**, containing traces of vinylic acetate **5**, was obtained from flash column chromatography of the crude reaction mixture from the preparation of compound **19.**

Physical appearance: clear pale-yellow oil

^{1}H NMR (400 MHz, C_6D_6) δ 7.39 – 7.36 (m, 2H), 7.20 – 7.17 (m, 2H), 7.07 (tt, *J* = 7.3, 1.3 Hz, 1H), 6.05 (dt, *J* = 12.1, 1.3 Hz, 1H), 5.30 (dt, *J* = 12.1, 7.5 Hz, 1H), 1.82 (qd, *J* = 7.2, 1.3 Hz, 2H), 1.48 (s, 6H), 1.31 – 1.18 (m, 12H), 0.90 (t, *J* = 7.0 Hz, 3H).

^{13}C{^{1}H} NMR (101 MHz, C_6D_6) δ 146.6, 141.9, 128.6, 127.3, 126.2, 109.0, 78.7, 32.3, 31.1, 29.85, 29.78, 29.4, 29.1, 28.1, 23.1, 14.4.

HRMS (APCI) *m/z*: $[M+H]^+$ Calcd for $C_{19}H_{31}O$ 275.2369; Found 275.2374.

(*E*)-*t*-Butyl vinylic ether 7:

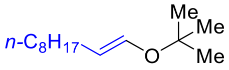

7, traces found for both DTBP and DCP conditions

Physical appearance: clear oil

^{1}H NMR (400 MHz, C_6D_6) δ 6.29 (dt, *J* = 12.0, 1.3 Hz, 1H), 5.26 (dt, *J* = 12.0, 7.5 Hz, 1H), 1.98 (qd, *J* = 7.3, 1.3 Hz, 2H), 1.41 – 1.19 (m, 12H), 1.12 (s, 9H), 0.90 (t, *J* = 6.8 Hz, 3H).

^{13}C{^{1}H} NMR (101 MHz, C_6D_6) δ 140.7, 109.0, 74.9, 32.3, 31.2, 29.9, 29.8, 29.5, 28.4, 28.0, 23.1, 14.4.

HRMS (APCI) *m/z*: $[M+H]^+$ Calcd for $C_{20}H_{39}O$ 295.3006; Found 295.3001.

C. Reactant scope

Vinylic pinacolboronate esters:

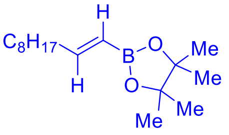

1

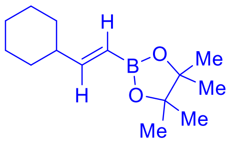

8a

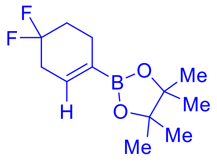

8b

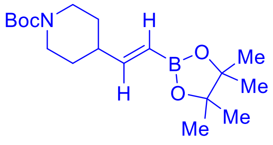

8c

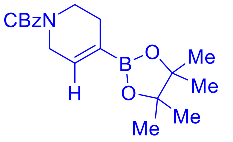

8d

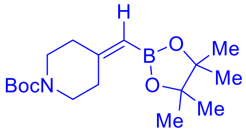

8e

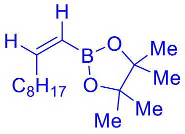

8f

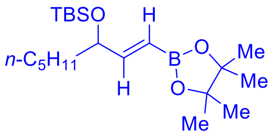

8g

Alcohols:

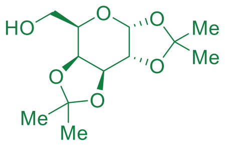

2

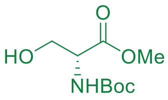

9a

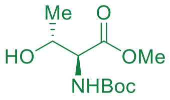

9b

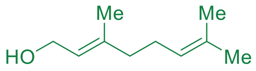

9c

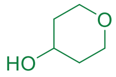

9d

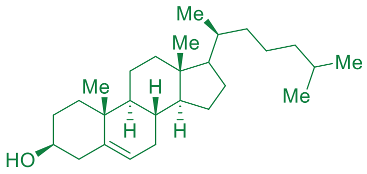

9e

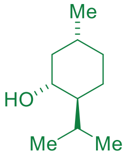

9f

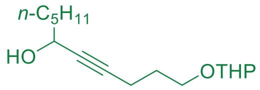

9g

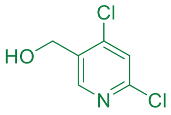

9h

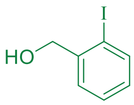

9i

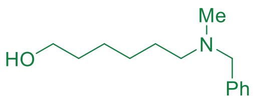

9j

1. **Representative general procedures:**

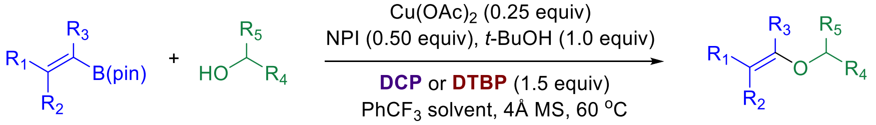

Conditions A with DCP oxidant: A 4 mL reaction vial was charged with a stir bar and 110-115 mg of 4Å MS, then flame-dried and cooled in air. The alcohol reactant (0.31 mmol, 1.0 equiv), NPI (18 μL, 0.16 mmol, 0.50 equiv), *t*-BuOH (30 μL, 0.31 mmol, 1.0 equiv), DCP (126 mg, 0.47 mmol, 1.5 equiv), $Cu(OAc)_2$ (14 mg, 0.08 mmol, 0.25 equiv), and vinylic pinacolboronate reactant (0.47 mmol, 1.5 equiv) were added sequentially under air to the vial. The vial was sealed with a septum cap and sparged with argon. $PhCF_3$ (0.5 mL, 0.6 M based on mmol of the alcohol reactant) was added to the reaction vial and the mixture was further purged with argon for 3-5 minutes. The septum cap was replaced with a solid cap and the vial was sealed with Teflon and electrical tape. The vial was stirred at 60 °C on a pre-heated heating block with an internal temperature probe. After the reaction was done stirring at time ranging from 35 minutes to 18 hours, the reaction vial was cooled to room temperature, and its contents were diluted with DCM. The crude reaction mixture was transferred to a 20 mL vial using DCM to rinse and was concentrated by rotary evaporation. A ^{1}H NMR spectrum of the crude reaction mixture was measured in C_6D_6. The crude reaction mixture was purified by flash column chromatography with silica gel treated with 2% Et_3N in hexanes and eluted with hexanes/EtOAc mixtures to isolate the desired vinylic ether products.

Conditions B with DTBP oxidant: Protocols were similar to conditions A but with DTBP as oxidant (68 mg, 0.47 mmol, 1.5 equiv) instead of DCP.

Procedure for parallel reaction set-up (usually done for >4 parallel reactions at a time): In each 4 mL reaction vial (flame-dried with a stir bar and 110-115 mg of 4 Å MS), alcohol reactant (0.31 mmol, 1.0 equiv), DCP or DTBP oxidant (0.47 mmol, 1.5 equiv), $Cu(OAc)_2$ (14 mg, 0.08 mmol, 0.25 equiv), and vinylic pinacolboronate reactant (0.47 mmol, 1.5 equiv) were added sequentially under air. The reaction vials were placed under argon through septum caps. In a separate oven-dried vial, a stock solution of NPI (0.32 M) and *t*-BuOH (0.62 M) in $PhCF_3$ was prepared under argon ($V_{solution}$ = [0.55 mL x ($n_{reaction}$+1)]). Afterwards, 0.55 mL of the $PhCF_3$ solution was added to each reaction vial. The reaction vials were further sparged with argon for 3-5 minutes. The septum caps were replaced with solid caps and the vials were sealed with Teflon and electrical tape. The reaction vials were stirred at 60 °C on a pre-heated heating block with an internal temperature probe. Work-up and purification were conducted for each reaction vial as described above.

Note: Due to the difficulty of isolating pure side products under optimized cross-coupling conditions, isolated yields were generally not reported for the side products.

2. Vinylic boronate scope, giving vinylic ethers 10 - 15:

(*E*)-vinylic ether 10:

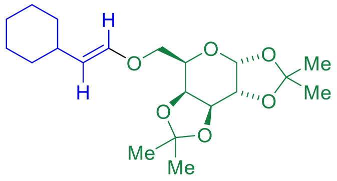

10, **80% yield (DCP), 1.5 h**
76% yield (DTBP), 3.0 h

Using conditions A for 1.5-hour reaction time, the vinylic ether **10** was prepared from alcohol **2** (81 mg, 0.31 mmol, 1.0 equiv) and vinylic pinacolboronate **8a** (110 mg, 0.47 mmol, 1.5 equiv). ^{1}H NMR analysis of crude reaction mixture showed an 80% NMR yield of vinylic ether **10**.

The crude product mixture was purified by flash column chromatography with silica gel pre-treated with 2% Et_3N in hexanes (by dry loading on 1.1 g Celite), eluting with a gradient of 100:0 – 98:2 – 97:3 hexanes/EtOAc. Fractions were checked by TLC in 90:10 hexanes/EtOAc (visualized with PMA stain), collected, and concentrated by rotary evaporation to afford the vinylic ether **10** as a clear pale-yellow oil (91 mg, 80% isolated yield). ^{1}H NMR characterization of the pure product **10** matched published data.[8] ^{1}H NMR spectrum was also recorded in C_6D_6.

Note: Reactions on similar scales (~0.31 mmol scale, following **conditions A**) were also conducted at 35-minute, 60-minute, and 18-hour reaction times and gave similar NMR yields for vinylic ether **10** (~80-82% yield). Compound **10** was isolated from 35-minute experiment (85 mg, 74% isolated yield) and 18-hour experiment (90 mg, 79% isolated yield), respectively.

Using conditions B for 3-hour reaction time, the vinylic ether **10** was prepared on a similar reaction scale (~0.31 mmol scale; 87 mg, 76% isolated yield).

Characterization data of compound **10**:

^{1}H NMR (400 MHz, $CDCl_3$) δ 6.27 (dd, *J* = 12.8, 1.0 Hz, 1H), 5.54 (d, *J* = 5.0 Hz, 1H), 4.76 (dd, *J* = 12.7, 7.8 Hz, 1H), 4.62 (dd, *J* = 7.9, 2.4 Hz, 1H), 4.32 (dd, *J* = 5.0, 2.4 Hz, 1H), 4.26 (dd, *J* = 7.9, 1.9 Hz, 1H), 4.02 (ddd, *J* = 7.2, 5.6, 2.0 Hz, 1H), 3.82 (dd, *J* = 10.4, 5.6 Hz, 1H), 3.77 (dd, *J* = 10.4, 7.0 Hz, 1H), 1.86 (tdt, *J* = 10.9, 7.0, 3.4 Hz, 1H), 1.74 – 1.55 (m, 5H), 1.53 (s, 3H), 1.46 (s, 3H), 1.35 (s, 3H), 1.33 (s, 3H), 1.30 – 0.98 (m, 5H).

^{1}H NMR (600 MHz, C_6D_6) δ 6.34 (dd, *J* = 12.7, 0.9 Hz, 1H), 5.52 (d, *J* = 5.0 Hz, 1H), 4.82 (dd, *J* = 12.7, 7.9 Hz, 1H), 4.49 (dd, *J* = 8.0, 2.4 Hz, 1H), 4.28 (td, *J* = 6.2, 1.7 Hz, 1H), 4.17 (dd, *J* = 5.0, 2.3 Hz, 1H), 4.14 (dd, *J* = 8.0, 1.9 Hz, 1H), 4.07 (dd, *J* = 10.0, 6.3 Hz, 1H), 4.02 (dd, *J* = 10.0, 6.2 Hz, 1H), 1.76 (tdtd, *J* = 11.2, 7.8, 3.4, 0.9 Hz, 1H), 1.62 (ttd, *J* = 8.7, 4.1, 2.6 Hz, 4H), 1.55 (dddt, *J* = 12.8, 5.2, 3.4, 1.6 Hz, 1H), 1.45 (s, 3H), 1.43 (s, 3H), 1.20 – 1.16 (apparent dt, *J* = 12.7, 3.6 Hz, 1H), 1.16 – 1.12 (m, 1H), 1.14 (s, 3H), 1.07 (tt, *J* = 12.3, 3.0 Hz, 1H), 1.04 (s, 3H), 1.03 – 0.95 (m, 2H).

(*E*)-vinylic ether 11:

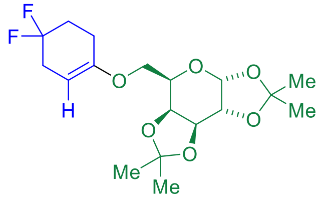

11, 72% yield (DCP), 35 min

Using conditions A for 35-minute reaction time with minor modifications, vinylic ether **11** was prepared from alcohol **2** (81 mg, 0.31 mmol, 1.0 equiv) and vinylic pinacolboronate **8b** (121 mg, 0.50 mmol, **1.6 equiv**). ^{1}H NMR analysis of crude reaction mixture showed estimated yield of 74% vinylic ether **11**.

The crude product mixture was purified by flash column chromatography with silica gel pre-treated with 2% Et_3N in hexanes (by dry loading on 1.1 g celite), eluting with a gradient of 100:0 – 98:2 – 97:3 – 94:6 – 92:8 – 90:10 hexanes/EtOAc. Fractions were checked by TLC in 80:20 hexanes/EtOAc (visualized with PMA stain), collected, and concentrated by rotary evaporation to afford the vinylic ether **11** as an light-yellow crystalline solid (84 mg, 72% yield).

Note: Reactions on similar scale (~0.31 mmol scale, following **conditions A**) conducted for 60 minutes and 18 hours gave vinylic ether **11** (60 minutes → 82 mg, 70% isolated yield; 18 hours → 88 mg, 75% isolated yield).

Characterization of compound **11**:

^{1}H NMR (400 MHz, C_6D_6) δ 5.51 (d, *J* = 5.0 Hz, 1H), 4.51 (dd, *J* = 7.9, 2.4 Hz, 1H), 4.25 (td, *J* = 6.2, 1.9 Hz, 1H), 4.18 (dd, *J* = 5.1, 2.4 Hz, 1H), 4.12 (dd, *J* = 8.0, 1.9 Hz, 1H), 4.09 – 4.05 (broad, 1H), 3.96 (dd, *J* = 9.6, 6.4 Hz, 1H), 3.89 (dd, *J* = 9.6, 6.1 Hz, 1H), 2.28 (apparent tt, *J* = 14.7, 2.0 Hz, 2H), 2.14 (apparent t, *J* = 6.9 Hz, 2H), 1.75 – 1.56 (m, 2H), 1.46 (s, 3H), 1.43 (s, 3H), 1.15 (s, 3H), 1.05 (s, 3H).

^{13}C{^{1}H} NMR (101 MHz, C_6D_6) δ 153.2 (t, $^{4}J_{CF}$ = 1.02 Hz), 123.0 (t, $^{1}J_{CF}$ = 239.9 Hz), 109.3, 108.5, 96.9, 89.6 (t, $^{3}J_{CF}$ = 6.0 Hz), 71.5, 71.2, 71.1, 66.6, 66.4, 33.0 (t, $^{2}J_{CF}$ = 27.1 Hz), 30.3 (t, $^{2}J_{CF}$ = 25.1 Hz), 26.3, 26.2, 25.9 (t, $^{3}J_{CF}$ = 5.6 Hz), 24.9, 24.4.

^{19}F NMR (376 MHz, C_6D_6) δ -97.73 (m), -97.64 (m)

HRMS (ESI) *m/z*: $[M+H]^+$ Calcd for $C_{18}H_{27}F_2O_6$ 377.1770; Found 377.1769.

$[\alpha]^{22}_{D}$ -58.0 (c = 0.10, acetone).

Melting point: m.p. = 79.8 – 82.6 °C

(*E*)-vinylic ether 12:

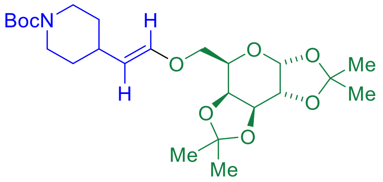

12, 75% yield (DCP), 18 h
**similar NMR yields observed at 35 mins*

Using conditions A for 18-hour reaction time, the vinylic ether **12** was prepared from alcohol **2** (81 mg, 0.31 mmol, 1.0 equiv) and vinylic pinacolboronate **8c** (157 mg, 0.47 mmol, 1.5 equiv). ^{1}H NMR analysis of crude reaction mixture showed an NMR yield of 73% vinylic ether **12**.

The crude product mixture was purified by flash column chromatography with silica gel pre-treated with 2% Et_3N in hexanes (by wet loading with hexane and minimal DCM), eluting with a gradient of 100:0 – 98:2 – 95:5 – 93:7 – 90:10 – 80:20 hexanes/EtOAc. Fractions were checked with TLC in 85:15 hexanes/EtOAc (visualized with PMA stain), collected, and concentrated by rotary evaporation to afford the vinylic ether **12** as a clear yellow oil (109 mg, 75% yield, >97% pure by ^{1}H NMR, containing traces of aldehyde decomposition product).

Note: A reaction on similar scale (~0.31 mmol scale, following **conditions A**) conducted for 35 minutes gave similar results based on ^{1}H NMR crude spectrum (72~74% NMR yield of vinylic ether **12**).

Characterization of compound **12**:

^{1}H NMR (400 MHz, C_6D_6) δ 6.21 (d, *J* = 12.7 Hz, 1H), 5.50 (d, *J* = 5.0 Hz, 1H), 4.56 (dd, *J* = 12.7, 7.8 Hz, 1H), 4.48 (dd, *J* = 8.0, 2.3 Hz, 1H), 4.29 (broad, 1H), 4.22 (td, *J* = 6.2, 1.8 Hz, 1H), 4.17 (dd, *J* = 5.1, 2.3 Hz, 1H), 4.09 (dd, *J* = 8.0, 1.9 Hz, 1H), 3.98 (dd, *J* = 10.0, 6.1 Hz, 1H), 3.93 (dd, *J* = 10.0, 6.3 Hz, 1H), 4.05 – 3.85 (broad, 1H) 2.54 – 2.33 (broad, 2H), 1.64 – 1.51 (broad, 1H), 1.47 (s, 9H), 1.44 (s, 3H), 1.41 (s, 3H), 1.28 (broad, 1H), 1.24 (broad, 1H), 1.13 (s, 3H), 1.08 (broad m, 1H), 1.04 (s, 3H), 1.02 (broad m, 1H).

$^{13}C\{^1H\}$ NMR (101 MHz, C_6D_6) δ 154.6, 146.0, 109.3, 108.8, 108.5, 96.8, 78.7, 71.4, 71.2, 71.0, 68.1, 66.8, 44.3 (broad), 43.7 (broad), 35.5, 33.3 (broad, two carbons overlapping based on HSQC), 28.6, 26.2 (two carbons overlapping based on HSQC), 24.9, 24.4.

HRMS (ESI) *m/z*: $[M+Na]^+$ Calcd for $C_{24}H_{39}NO_8Na$ 492.2568; Found 492.2544.

HRMS (ESI) *m/z*: $[M+H]^+$ Calcd for $C_{24}H_{40}NO_8$ 470.2748; Found 470.2726.

HRMS (ESI) *m/z*: $[M-COOC_4H_9+H]^+$ Calcd for fragment $C_{19}H_{32}NO_6$ 370.2224; Found 370.2222.
Proposed fragment structure:

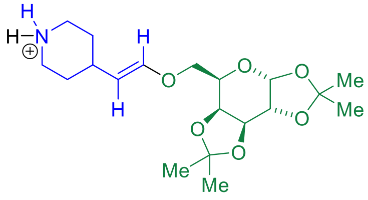

$[\alpha]^{22}_D$ -48.1 (c = 0.21, acetone).

(*E*)-vinylic ether 13:

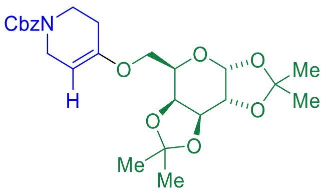

13, 36% yield (DCP), 18 h
41% yield (DTBP), 18 h

Using conditions B for 18-hour reaction time with minor modifications, the vinylic ether **13** was prepared from alcohol **2** (81 mg, 0.31 mmol, 1.0 equiv) and vinylic pinacolboronate **8d** (170 mg, 0.50 mmol, **1.6 equiv**).

^{1}H NMR analysis of crude reaction mixture showed formation of vinylic ether **13**, but NMR yield was not accurately determined due to broadening of NMR peaks. The crude product mixture was purified by flash column chromatography with silica gel pre-treated with 2% Et_3N in hexanes (by dry loading on 1.1 g celite), eluting with a gradient of of 95:5 – 93:7 – 90:10 – 80:20 hexanes/EtOAc. Fractions were checked with TLC in 80:20 hexanes/EtOAc (visualized with PMA stain), collected, and concentrated by rotary evaporation to afford the vinylic ether **13** as a clear yellow oil (60 mg, 41% yield).

Using conditions A for 18-hour reaction time with minor modifications, alcohol **2** (81 mg, 0.31 mmol, 1.0 equiv) and vinylic pinacolboronate **8d** (144 mg, 0.50 mmol, **1.35 equiv**) gave vinylic ether **13** (53 mg, 36% isolated yield).

Characterization data for compound **13**:

^{1}H and ^{13}C NMR spectra were measured at 60 °C, due to significant broadening of resonances at 20 °C.

^{1}H NMR (600 MHz, C_6D_6, 60 °C) δ 7.23 (broad d, *J* = 7.6 Hz, 2H), 7.11 (tt, *J* = 7.6, 1.1 Hz, 2H), 7.05 (tt, *J* = 7.3, 1.6 Hz, 1H), 5.48 (d, *J* = 5.0 Hz, 1H), 5.12 (s, 2H), 4.49 (dd, *J* = 7.9, 2.4 Hz, 1H), 4.30 – 4.25 (broad*, 1H), 4.21 (td, *J* = 6.2, 1.9 Hz, 1H), 4.16 (dd, *J* = 5.0, 2.4 Hz, 1H), 4.13 (dd, *J* = 7.9, 1.9 Hz, 1H), 3.97 (dd, *J* = 9.7, 6.5 Hz, 1H), 3.88 (dd, *J* = 9.7, 5.9 Hz, 1H), 3.75 – 3.93 (broad*, 2H), 3.36 (broad, 2H), 1.99 (broad, 2H), 1.43 (s, 3H), 1.41 (s, 3H), 1.16 (s, 3H), 1.07 (s, 3H). *Broadening of ^{1}H NMR signals in compound **13** was attributed to carbamate rotamers.

^{13}C{^{1}H} NMR (151 MHz, C_6D_6, 60 °C) δ 155.2, 153.3 (broad), 137.9, 128.6, 128.3, 128.2**, 109.4, 108.6, 97.0, 92.3 (broad), 71.7, 71.4, 71.3, 67.1, 66.7, 66.2, 42.3 (broad), 40.9, 28.2 (broad), 26.3, 26.2, 25.0, 24.6. ** This resonance was obscured by C_6D_6, however HSQC shows correlation with ^{1}H resonance at 7.05 ppm.

HRMS (APCI) *m/z*: $[M+H]^+$ Calcd for $C_{25}H_{34}NO_8$ 476.2279; Found 476.2281.

$[\alpha]^{22}_D$ -53.2 (c = 0.10, acetone).

(*E*)-vinylic ether 14:

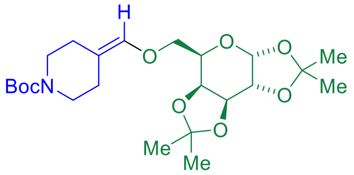

14, 60% yield (DCP), 18 h

Using conditions A for 18-hour reaction time, the vinylic ether **14** was prepared from alcohol **2** (81 mg, 0.31 mmol, 1.0 equiv) and vinylic pinacolboronate **8e** (150 mg, 0.47 mmol, 1.5 equiv). ^{1}H NMR analysis of crude reaction mixture showed estimated 63% NMR yield of vinylic ether **14**.

The crude product mixture was purified by flash column chromatography with silica gel pre-treated with 2% Et_3N in hexanes (by dry loading on 1.1 g celite), eluting with a gradient of 95:5 – 93:7 – 90:10 – 80:20 hexanes/EtOAc. Fractions were checked with TLC in 80:20 hexanes/EtOAc (visualized with PMA stain), collected, and concentrated by rotary evaporation to afford the vinylic ether **14** as a clear yellow oil (85 mg, 60% yield).

Note: Reactions on similar scales (~0.31 mmol scale, following **conditions A**) were conducted for 35 minutes and 60 minutes. These gave similar results based on ^{1}H NMR crude spectrum (62~65% NMR yield of vinylic ether **14**).

Characterization of compound **14**:

^{1}H NMR (400 MHz, C_6D_6) δ 5.76 (broad s, 1H), 5.46 (d, *J* = 5.0 Hz, 1H), 4.47 (dd, *J* = 7.9, 2.4 Hz, 1H), 4.15 (dd, *J* = 5.0, 2.4 Hz, 1H), 4.09 (td, *J* = 6.2, 1.9 Hz, 1H), 4.02 (dd, *J* = 7.9, 1.9 Hz, 1H), 3.93 (m, 2H), 3.40 (broad, 4H), 2.36 (t, *J* = 5.8 Hz, 2H), 1.81 (t, *J* = 5.7 Hz, 2H), 1.44 (s, 9H), 1.43 (s, 3H), 1.38 (s, 3H), 1.13 (s, 3H), 1.03 (s, 3H).

^{13}C{^{1}H} NMR (151 MHz, C_6D_6) δ 154.6, 140.1, 114.5, 109.3, 108.5, 96.8, 78.8, 71.4, 71.2, 71.0, 67.2, 46.1 (doublet for rotamer pair), 44.8 (doublet for rotamer pair), 29.8 (broad), 28.5, 26.3, 26.2, 25.9 (broad), 24.9, 24.4.

Note: ^{13}C NMR recorded on the Varian 400 AVIII NMR spectrometer (101 MHz) at room temperature with 2-second relaxation delay did not show all carbon signals (i.e. carbons bonded directly to the amide nitrogen atom). Therefore, the reported ^{13}C NMR was re-recorded using Bruker NEO600 NMR instrument with 8-second relaxation delay at room temperature (23-25 °C), allowing observation of all ^{13}C resonances.

HRMS (ESI) *m/z*: $[M+H]^+$ Calcd for $C_{23}H_{38}NO_8$ 456.2592; Found 456.2591.

HRMS (APCI) *m/z*: $[M-COOC_4H_9+H]^+$ Calcd for fragment $C_{18}H_{30}NO_6$ 356.2068; Found 356.2080 as a major ion peak. Proposed fragment structure:

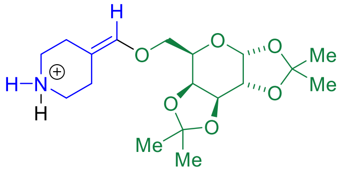

$[\alpha]^{22}_{D}$ -35.2 (c = 0.10, acetone).

(*Z*)-vinylic ether 15:

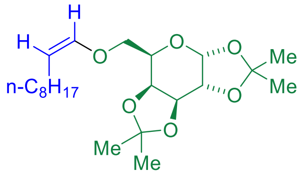

15, **52% yield (DCP), 4 h at 90 °C**

Using conditions A for 4-hour reaction time at *90 °C* with some modifications, the vinylic ether **15** was prepared from alcohol **2** (81 mg, 0.31 mmol, 1.0 equiv) and (*Z*)-vinylic pinacolboronate **8f** (>10 : 1 *Z/E*, 165 mg, 0.47 mmol, **2.0 equiv**). ^{1}H NMR analysis of crude reaction mixture showed full consumption of **8f**, estimated 46% NMR yield of vinylic ether **15**, and ~30% NMR yield of acetate ester **4**.

The crude product mixture was purified by flash column chromatography with silica gel pre-treated with 2% Et_3N in hexanes (by dry loading on 1.1 g celite), eluting with a gradient of 100:0 – 98:2 hexanes/EtOAc. Fractions were checked with TLC in 90:10 hexanes/EtOAc (visualized with PMA stain), collected, and concentrated by rotary evaporation to afford the vinylic ether **15** as a clear yellow oil (64 mg, 52% yield, 12 : 1 *Z/E*). ^{1}H NMR characterization of compound **15** in $CDCl_3$ matched reported values.[8]

^{1}H NMR (400 MHz, $CDCl_3$) δ 5.96 (dt, *J* = 6.2, 1.5 Hz, 1H), 5.53 (d, *J* = 5.0 Hz, 1H), 4.61 (dd, *J* = 8.0, 2.4 Hz, 1H), 4.37 (td, *J* = 7.3, 6.2 Hz, 1H), 4.31 (dd, *J* = 5.0, 2.4 Hz, 1H), 4.26 (dd, *J* = 7.9, 1.8 Hz, 1H), 3.98 (td, *J* = 6.2, 1.8 Hz, 1H), 3.92 (dd, *J* = 10.7, 6.1 Hz, 1H), 3.84 (dd, *J* = 10.6, 6.4 Hz, 1H), 2.06 (qd, *J* = 7.2, 1.5 Hz, 2H), 1.53 (s, 3H), 1.45 (s, 3H), 1.34 (s, 3H), 1.33 (s, 3H), 1.20 – 1.33 (m, 16H, overlapping with minor *E*-isomer), 0.87 (apparent t, *J* = 6.7 Hz, 4H, overlapping with minor *E*-isomer).

3. Alcohol scope, giving vinylic ethers 16 - 26:

(*E*)-vinylic ether 16:

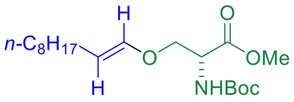

16, 57% yield (DCP), 18 h
68% yield (DTBP), 18 h

Using conditions B for 18-hour reaction time, the vinylic ether **16** was prepared from alcohol **9a** (68 mg, 0.31 mmol, 1.0 equiv) and vinylic pinacolboronate **1** (124 mg, 0.47 mmol, 1.5 equiv). ^{1}H NMR spectrum in C_6D_6 of the crude reaction mixture showed that vinylic pinacolboronate **1** was fully consumed and vinylic ether **16** was the major product (estimated ~72% NMR yield). Traces of vinylic acetate **5** and protodeboronation 1-decene **S1** were also observed.

The crude reaction mixture was purified by flash column chromatography with silica gel pre-treated with 2% Et_3N in hexanes (by dry loading on 1.1 g celite), eluting with a gradient of 98:2 – 97:3 – 96:4 – 95:5 – 90:10 hexanes/EtOAc. Fractions were checked with TLC in 90:10 hexanes/EtOAc (visualized with PMA stain), collected, and concentrated by rotary evaporation to afford the vinylic ether **16** as a yellow oil (75 mg, 68% yield).

Note: A reaction trial on similar scale (~0.31 mmol) using **conditions B** was conducted for 70 minutes. ^{1}H NMR crude spectrum showed that vinylic pinacolboronate **1** was not fully converted (~40% recovered).

Using conditions A for 18-hour reaction time, the vinylic ether **16** was prepared from a similar-scale reaction as above (61% estimated NMR yield; 63 mg, 57% isolated yield).

Characterization data of compound **16**:

^{1}H NMR (400 MHz, C_6D_6) δ 6.07 (d, *J* = 12.6 Hz, 1H), 5.47 (d, *J* = 8.8 Hz, 1H), 4.63 (dt, *J* = 12.6, 7.4 Hz, 1H), 4.61 (m, 1H), 3.85 (dd, *J* = 9.8, 3.0 Hz, 1H), 3.65 (dd, *J* = 9.8, 3.2 Hz, 1H), 3.21 (s, 3H), 1.77 (q, *J* = 6.7 Hz, 2H), 1.42 (s, 9H), 1.34 – 1.18 (m, 14H), 0.91 (t, *J* = 6.9 Hz, 3H).

^{13}C{^{1}H} NMR (101 MHz, C_6D_6) δ 170.6, 155.6, 145.9, 105.1, 79.6, 69.1, 54.0, 52.0, 32.3, 31.0, 29.9, 29.8, 29.5, 28.3, 28.0, 23.1, 14.4.

HRMS (ESI) *m/z*: $[M+Na]^+$ Calcd for $C_{19}H_{35}NO_5Na$ 380.2407; Found 380.2408.

HRMS (ESI) *m/z*: $[M-COOC_4H_9+H]^+$ Calcd for fragment $C_{14}H_{28}NO_3$ 258.2064; Found 258.2069.
Proposed fragment structure:

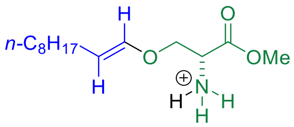

$[\alpha]^{22}_{D}$ -5.2 (c = 0.1, acetone).

(*E*)-vinylic ether 17:

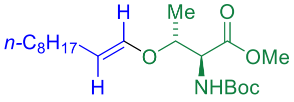

17, 38% yield (DCP)
48% yield (DTBP)

Using conditions B for 18-hour reaction time, the vinylic ether **17** was prepared from alcohol **9b** (72 mg, 0.31 mmol, 1.0 equiv) and vinylic pinacolboronate **1** (124 mg, 0.47 mmol, 1.5 equiv). ^{1}H NMR spectrum of the crude reaction mixture in C_6D_6 showed full consumption of vinylic pinacolboronate **1** and formation of compound **17** as the major product (estimated ~54% NMR yield). Traces of protodeboronation 1-decene **S1** and *t*-butyl vinylic ether **7** were observed (<2% NMR yields).

The crude reaction mixture was purified by flash column chromatography with silica gel pre-treated with 2% Et_3N in hexanes (by dry loading on 1.1 g celite), eluting with a gradient of 98:2 – 97:3 – 96:4 – 95:5 – 90:10 hexanes/EtOAc. Fractions were checked with TLC in 90:10 hexanes/EtOAc (visualized with PMA stain), collected, and concentrated by rotary evaporation to afford the vinylic ether **17** as a yellow oil (55 mg, 48% yield).

Note: Another reaction trial of similar scale (~0.31 mmol) using **conditions B** was also conducted for 70 minutes. ^{1}H NMR crude spectrum showed that vinylic pinacolboronate **1** was not fully converted (>90% recovered starting material).

Using conditions A for 18-hour reaction time, the vinylic ether **17** was prepared from a similar-scale reaction as above (~39% estimated NMR yield; 44 mg, 38% isolated yield).

Characterization data of compound **17**:

^{1}H NMR (400 MHz, C_6D_6) δ 5.87 (dt, *J* = 12.3, 1.3 Hz, 1H), 5.41 (d, *J* = 9.8 Hz, 1H), 4.93 (dt, *J* = 12.4, 7.4 Hz, 1H), 4.59 (dd, *J* = 9.7, 2.3 Hz, 1H), 4.22 (qd, *J* = 6.3, 2.3 Hz, 1H), 3.26 (s, 3H), 1.80 (apparent q, *J* = 6.5 Hz, 2H), 1.42 (s, 9H), 1.31 – 1.21 (m, 12H), 1.09 (d, *J* = 6.4 Hz, 3H), 0.91 (t, *J* = 6.8 Hz, 3H).

^{13}C{^{1}H} NMR (101 MHz, C_6D_6) δ 171.0, 156.3, 144.7, 107.8, 79.6, 76.1, 58.2, 51.9, 32.3, 30.9, 29.9, 29.8, 29.5, 28.3, 28.0, 23.1, 16.7, 14.4.

HRMS (ESI) *m/z*: $[M+Na]^+$ Calcd for $C_{20}H_{37}NO_5Na$ 394.2569; Found 394.2559.

HRMS (ESI) *m/z*: $[M-COOC_4H_9+H]^+$ Calcd for fragment $C_{15}H_{30}NO_3$ 272.2220; Found 272.2213.
Proposed fragment structure:

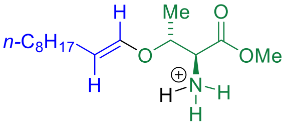

$[\alpha]^{22}_{D}$ +29.2 (c = 0.1, acetone).

(*E*)-vinylic ether 18:

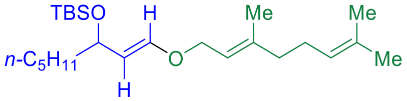

18, **65% yield (DCP), 40 min**

Using conditions A for 40-minute reaction time, the vinylic ether **18** was prepared from geraniol (**9c**, 48 mg, 0.31 mmol, 1.0 equiv) and vinylic pinacolboronate **8g** (171 mg, 0.47 mmol, 1.5 equiv). ^{1}H NMR analysis of the crude reaction mixture showed estimated NMR yield of 72% for vinylic ether **18**.

The crude reaction mixture was purified by flash column chromatography with silica gel pre-treated with 2% Et_3N in hexanes (by dry loading on 1.1 g celite), eluting with a gradient of 98:2 – 97:3 – 96:4 – 95:5 hexanes/EtOAc. Fractions were checked with TLC in 95:5 hexanes/EtOAc (visualized with PMA stain), collected, and concentrated by rotary evaporation to afford the vinylic ether **18** as a yellow oil (80 mg, 65% yield).

Characterization data of compound **18**:

^{1}H NMR (400 MHz, C_6D_6) δ 6.49 (dd, *J* = 12.7, 0.7 Hz, 1H), 5.49 (tq, *J* = 6.5, 1.3 Hz, 1H), 5.16 (apparent triplet of quintets, *J* = 6.9, 1.4 Hz, 1H), 4.96 (dd, *J* = 12.7, 8.2 Hz, 1H), 4.17 (apparent dd, *J* = 6.5, 2.6 Hz, 2H), 4.07 (ddd, *J* = 8.2, 6.8, 5.7 Hz, 1H), 2.10 (apparent broad quartet, *J* = 8.0 Hz, 2H), 1.99 (apparent dd, *J* = 8.0, 7.3 Hz, 2H), 1.72 (dddd, *J* = 12.5, 9.8, 6.7, 5.0 Hz, 1H), 1.66 (q, *J* = 1.3 Hz, 3H), 1.58 (dt, *J* = 9.8, 5.6 Hz, 1H), 1.54 – 1.51 (broad m, 3H), 1.46 (broad m, 3H), 1.44 – 1.25 (m, 6H), 1.04 (s, 9H), 0.91 (t, *J* = 6.8 Hz, 3H), 0.16 (s, 3H), 0.14 (s, 3H).

^{13}C{^{1}H} NMR (101 MHz, C_6D_6) δ 147.7, 140.2, 131.6, 124.4, 120.6, 108.5, 72.1, 65.9, 40.2, 39.8, 32.2, 26.8, 26.2, 25.84, 25.76, 23.1, 18.5, 17.7, 16.5, 14.3, -3.5, -4.4.

HRMS (APCI) *m/z*: $[M+H]^+$ Calcd for $C_{24}H_{47}O_2{}^{28}Si$ 395.3340; Found 395.3356.

(*E*)-vinylic ether 19:

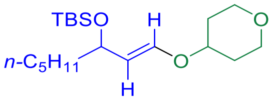

19, 71% yield (DCP), 40 min

Using conditions A for 40-minute reaction time, the vinylic ether **19** was prepared from alcohol **9d** (32 mg, 0.31 mmol, 1.0 equiv) and vinylic pinacolboronate **8g** (171 mg, 0.47 mmol, 1.5 equiv). Due to the possible volatility of alcohol **9d**, estimated NMR yield was not calculated.

The crude reaction mixture was purified by flash column chromatography with silica gel pre-treated with 2% Et_3N in hexanes (by wet loading with hexanes), eluting with a gradient of 100:0 – 98:2 – 97:3 hexanes/EtOAc. Fractions were checked with TLC in 90:10 hexanes/EtOAc (visualized with PMA stain), collected, and concentrated by rotary evaporation to afford the vinylic ether **19** as a yellow oil (75 mg, 71% yield).

Characterization data of compound **19**:

^{1}H NMR (400 MHz, C_6D_6) δ 6.20 (dd, *J* = 12.6, 0.7 Hz, 1H), 5.07 (dd, *J* = 12.5, 8.1 Hz, 1H), 4.07 (broad q, *J* = 7.0 Hz, 1H), 3.77 (dd, *J* = 5.5, 4.1 Hz, 1H), 3.74 (dd, *J* = 5.4, 4.2 Hz, 1H), 3.55 (tt, *J* = 8.2, 4.2 Hz, 1H), 3.20 (dd, *J* = 8.4, 3.3 Hz, 1H), 3.17 (dd, *J* = 8.5, 3.4 Hz, 1H), 1.70 (m, 1H), 1.64 – 1.48 (m, 5H), 1.47 – 1.35 (m, 2H), 1.36 – 1.25 (m, 4H), 1.04 (s, 9H), 0.90 (t, *J* = 6.9 Hz, 3H), 0.15 (s, 6H).

^{13}C{^{1}H} NMR (101 MHz, C_6D_6) δ 145.8, 110.5, 74.2, 71.8, 65.03, 65.02, 40.0, 32.55, 32.46, 32.2, 26.2, 25.7, 23.1, 18.5, 14.3, -3.6, -4.4.

HRMS (APCI) *m/z*: $[M+H]^+$ Calcd for $C_{19}H_{37}O_3{}^{28}Si$ 341.2507; Found 341.2521.

(*E*)-vinylic ether 20:

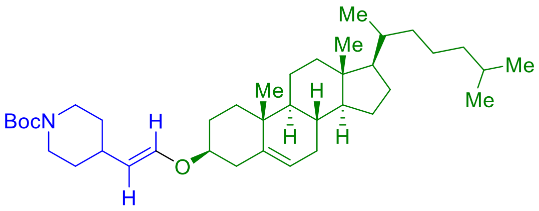

20, 57% yield (DCP), 14 h

Using conditions A for 14-hour reaction time, the vinylic ether **20** was prepared from cholesterol (**9e**, 119 mg, 0.31 mmol, 1.0 equiv) and vinylic pinacolboronate **8c** (157 mg, 0.47 mmol, 1.5 equiv).

The crude reaction mixture was purified by flash column chromatography with silica gel pre-treated with 2% Et_3N in hexanes (by wet loading with 2% Et_3N in hexanes), eluting with a gradient of 100:0 – 98:2 – 97:3 hexanes/EtOAc. Fractions were checked with TLC in 95:5 hexanes/EtOAc (visualized with PMA stain), collected, and concentrated by rotary evaporation to afford the vinylic ether **20** as a white solid (106 mg, 57% yield).

Characterization data of compound **20**:

^{1}H NMR (600 MHz, C_6D_6) δ 6.09 (dd, *J* = 12.5, 1.0 Hz, 1H), 5.39 (dt, *J* = 5.4, 2.1 Hz, 1H), 4.91 (dd, *J* = 12.5, 7.7 Hz, 1H), 4.34 (broad, 1H), 4.05 (broad, 1H), 3.53 (tt, *J* = 11.2, 4.6 Hz, 1H), 2.64 – 2.48 (broad, ~2H)*, 2.53 (ddd, *J* = 13.3, 4.9, 2.3 Hz, 1H), 2.45 (ddtd, *J* = 13.4, 11.2, 3.1, 2.4 Hz, 1H), 2.04 (dt, *J* = 12.6, 3.4 Hz, 1H), 1.95 (dtd, *J* = 17.5, 5.3, 2.6 Hz, 1H), 1.95 – 1.90 (m, 1H), 1.86 (dtd, *J* = 13.3, 9.5, 6.3 Hz, 1H), 1.75 – 1.68 (m, 1H), 1.71 (dt, *J* = 12.7, 3.5 Hz, 1H), 1.67 – 1.60 (m, 1H), 1.59 – 1.52 (m, ~3H)*, 1.49 (s, 9H), 1.46 – 1.41 (m, ~5H)*, 1.41 – 1.35 (m, ~5H)*, 1.32 – 1.22 (m, ~4H)*, 1.21 – 1.14 (m, ~4H)*, 1.13 – 1.08 (m, 2H), 1.06 (q, *J* = 6.2 Hz, 1H), 1.03 (d, *J* = 6.6 Hz, 3H), 0.99 (td, *J* = 13.9, 4.3 Hz, 1H), 0.96 – 0.94 (m, 1H), 0.94 - 0.93 (overlapping peaks for three methyl groups, corresponding to two doublets *J* = ~7 Hz and one singlet, 9H), 0.93 – 0.91 (m, 1H), 0.67 (s, 3H).

* ^{1}H NMR integrations are estimated, due to overlapping resonances. Assignments were assisted by using HSQC data, and referring to ^{1}H NMR spectrum of cholesterol (**9e**) in C_6D_6.

^{13}C{^{1}H} NMR (151 MHz, C_6D_6) δ 154.7, 144.6, 140.6, 122.3, 110.6, 79.5, 78.8, 57.0, 56.6, 50.6, 44.6 (broad), 43.8 (broad), 42.6, 40.2, 40.0, 39.6, 37.5, 37.1, 36.7, 36.3, 35.7, 33.5 (broad, correlating to two overlapping carbons based on HSQC), 32.4, 32.2, 28.9, 28.7, 28.6, 28.4, 24.6, 24.4, 23.1, 22.8, 21.5, 19.5, 19.1, 12.1.

Note: ^{13}C assignments were assisted by using HSQC data. ^{13}C NMR spectrum showed other minor peaks which we attribute to carbamate rotamers.

HRMS (ESI) *m/z*: $[M+H]^+$ Calcd for $C_{39}H_{66}NO_3$ 596.5037; Found 596.5036.

HRMS (ESI) *m/z*: $[M-COOC_4H_9+H]^+$ Calcd for fragment $C_{34}H_{58}NO$ 496.4513 ; Found 496.4512.
Proposed fragment structure:

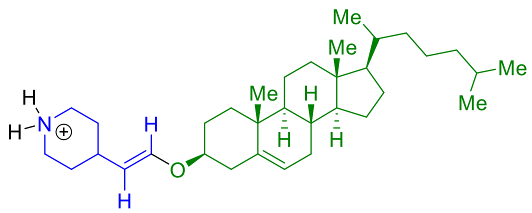

$[\alpha]^{22}_D$ -11.8 (c = 0.1, acetone).

Melting point: m.p. = 124.5 – 128.0 °C

(*E*)-vinylic ether 21:

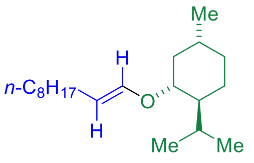

21, 43% yield (DCP), 40 min
38% yield (DTBP), 18 h

Using conditions A for 40-minute reaction time, the vinylic ether **21** was prepared from menthol (**9f**, 48 mg, 0.31 mmol, 1.0 equiv) and vinylic pinacolboronate **1** (124 mg, 0.47 mmol, 1.5 equiv). ^{1}H NMR analysis of the crude reaction mixture in C_6D_6 showed ~42% NMR yield of vinylic ether **21**, ~34% NMR yield of cumyl vinylic ether **6**, and ~11% NMR yield of *t*-butyl vinylic ether **7**.

The crude reaction mixture was purified by flash column chromatography with silica gel pre-treated with 2% Et_3N in hexanes (by wet loading with hexanes), eluting with a gradient of 100:0 hexanes. Fractions were checked with TLC in 100% hexanes (visualized with PMA stain), collected, and concentrated by rotary evaporation to afford the vinylic ether **21** as a yellow oil (39 mg, 43% yield). Cumyl vinylic ether **6** was isolated (42 mg, 33% yield calculated based on vinylic pinacolboronate **1** as the limiting reagent). ^{1}H NMR characterization of compound **21** in $CDCl_3$ matched published data.[8] ^{1}H NMR spectrum was also recorded in C_6D_6.

Using conditions B for 18-hour reaction time, the vinylic ether **21** was prepared from a similar-scale reaction (35 mg, 38% isolated yield).

Note: A reaction on similar scale as above was conducted with **conditions B**, albeit without *t*-BuOH additive, resulted in lower NMR yields of vinylic ether **21** (31% yield) and *t*-butyl vinylic ether **7** (12% yield), in comparison to 38% NMR yield of vinylic ether **21** and 19% NMR yield of *t*-butyl vinylic ether **7** from the above experiment with *t*-BuOH additive.

Characterization data of compound **21**:

^{1}H NMR (400 MHz, $CDCl_3$) δ 6.07 (d, *J* = 12.3 Hz, 1H), 4.86 (dt, *J* = 12.2, 7.4 Hz, 1H), 3.38 (td, *J* = 10.7, 4.3 Hz, 1H), 2.13 (septet of doublets, *J* = 7.0, 2.7 Hz, 1H), 2.04 (dtd, *J* = 12.6, 4.0, 2.0 Hz, 1H), 1.88 (q, *J* = 6.9 Hz, 2H), 1.64 (m, 2H), 1.44 – 1.22 (m, 14H), 1.05 – 0.94 (m, 3H), 0.91 (d, *J* = 6.5 Hz, 3H), 0.89 (d, *J* = 7.1 Hz, 3H), 0.88 (t, *J* = 6.7 Hz, 3H), 0.77 (d, *J* = 7.0 Hz, 3H).

^{1}H NMR (400 MHz, C_6D_6) δ 6.16 (dt, *J* = 12.3, 1.3 Hz, 1H), 5.12 (dt, *J* = 12.3, 7.4 Hz, 1H), 3.34 (td, *J* = 10.6, 4.2 Hz, 1H), 2.37 (septet of doublets, *J* = 7.0, 2.8 Hz, 1H), 2.08 (broad doublet, *J* = 11.8 Hz, 1H), 1.98 (qd, *J* = 7.0, 1.3 Hz, 2H), 1.51 (dt, *J* = 6.3, 3.3 Hz, 1H), 1.47 (quintet, *J* = 3.7 Hz, 1H), 1.42 – 1.30 (m, 6H), 1.28 (m, 8H), 1.17 (dtt, *J* = 15.1, 6.3, 3.3 Hz, 1H), 1.01 (td, *J* = 12.2, 10.7 Hz, 1H), 0.91 (t, *J* = 6.8 Hz, 6H), 0.84 (apparent triplet, *J* = 7.0 Hz, 6H), 0.71 (multiplet, 1H).

(*E*)-vinylic ether 22:

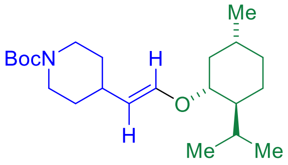

22, 35% yield (DCP), 40 min
37% yield (DTBP), 18 h

Using conditions A for 40-minute reaction time, the vinylic ether **22** was prepared from menthol (**9f**, 48 mg, 0.31 mmol, 1.0 equiv) and vinylic pinacolboronate **8c** (157 mg, 0.47 mmol, 1.5 equiv). ^{1}H NMR analysis of the crude reaction mixture in C_6D_6 showed ~40% NMR yield of vinylic ether **22**, ~31% NMR yield of cumyl vinylic ether **6**, ~11% NMR yield of *t*-butyl vinylic ether **7**, and traces of protodeboronation 1-decene **S1** and diene **S2**.

The crude reaction mixture was purified by flash column chromatography with silica gel pre-treated with 2% Et_3N in hexanes (by wet loading with hexanes), eluting with a gradient of 98:2 - 95:5 hexanes/EtOAc. Fractions were checked with TLC in 100% hexanes (visualized with PMA stain), collected, and concentrated by rotary evaporation to afford the vinylic ether **22** as a yellow oil (40 mg, 35% yield).

Using conditions B for 18-hour reaction time, the vinylic ether **22** was prepared from a similar-scale reaction (42 mg, 37% isolated yield).

Characterization data of compound **22**:

^{1}H NMR (400 MHz, C_6D_6) δ 6.01 (dd, *J* = 12.5, 1.0 Hz, 1H), 4.87 (dd, *J* = 12.4, 7.6 Hz, 1H), 4.33 (broad, 1H), 4.06 (broad, 1H), 3.30 (td, *J* = 10.6, 4.2 Hz, 1H), 2.53 (broad d, *J* = 11.7 Hz, 1H), 2.48 (broad d, *J* = 11.7 Hz, 1H), 2.30 (septet of doublets, *J* = 7.0, 2.8 Hz, 1H), 2.01 (dtd, *J* = 12.2, 3.6, 2.0 Hz, 1H), 1.71 (tdt, *J* = 11.4, 7.5, 3.7 Hz, 1H), 1.53 – 1.46 (m, 2H, confirmed by HSQC) 1.47 (s, 9H), 1.39 – 1.31 (m, 3H), 1.15 (m, 3H), 0.96 (td, *J* = 12.3, 10.9 Hz, 1H), 0.89 (d, *J* = 7.0 Hz, 3H), 0.88 – 0.83 (m, 1H, confirmed by HSQC), 0.83 (d, *J* = 6.4 Hz, 3H), 0.81 (d, *J* = 7.0 Hz, 3H), 0.70 (qdd, *J* = 12.0, 3.0, 1.4 Hz, 1H).

^{13}C{^{1}H} NMR (101 MHz, C_6D_6) δ 154.6, 145.3, 110.2, 80.2, 78.8, 48.2, 44.3 (broad, correlating to two carbons by HSQC), 41.4, 35.6, 34.7, 33.5 (broad, correlating to two carbons by HSQC), 31.7, 28.6, 26.2, 23.9, 22.3, 20.9, 16.8.

HRMS (ESI) *m/z*: $[M+H]^+$ Calcd for $C_{22}H_{40}NO_3$ 366.3003; Found 366.3009.

HRMS (ESI) *m/z*: $[M\text{-}COOC_4H_9+H]^+$ Calcd for fragment $C_{17}H_{32}NO$ 266.2478; Found 266.2477.
Proposed fragment structure:

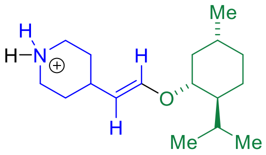

$[\alpha]^{22}_{D}$ -29.0 (c = 0.1, acetone).

(*E*)-vinylic ether 23:

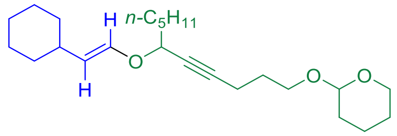

23, 42% yield (DCP), 40 min
39% yield (DTBP), 18 h

Using conditions A for 40-minute reaction time, the vinylic ether **23** was prepared from alcohol **9g** (83 mg, 0.31 mmol, 1.0 equiv) and vinylic pinacolboronate **8a** (110 mg, 0.47 mmol, 1.5 equiv). ^{1}H NMR analysis of the crude reaction mixture in C_6D_6 showed ~41% NMR yield of vinylic ether **23**, 20% NMR yield of diene derivative (based on similar chemical shifts and coupling constants to diene **S2**), and traces of protodeboronation alkene.

The crude reaction mixture was purified by flash column chromatography with silica gel pre-treated with 2% Et_3N in hexanes (by wet loading with hexanes), eluting with a gradient of 100:0 – 98:2 hexanes/EtOAc. Fractions were checked with TLC in 95:5 hexanes/EtOAc (visualized with PMA stain), collected, and concentrated by rotary evaporation to afford the vinylic ether **23** as a yellow oil (49 mg, 42% yield).

Using conditions B for 18-hour reaction time, the vinylic ether **23** was prepared from a similar scale reaction (45 mg, 39% isolated yield).

Characterization data of compound **23**:

^{1}H NMR (400 MHz, C_6D_6) δ 6.46 (dd, *J* = 12.5, 1.0 Hz, 1H), 5.22 (dd, *J* = 12.5, 7.9 Hz, 1H), 4.56 (t, *J* = 3.4 Hz, 1H), 4.36 (tt, *J* = 6.6, 2.0 Hz, 1H), 3.85 (dtd, *J* = 10.1, 6.2, 3.9 Hz, 1H), 3.77 (dddd, *J* = 11.1, 9.4, 3.0, 1.6 Hz, 1H), 3.44 – 3.33 (m, 2H), 2.25 (tt, *J* = 6.9, 1.8 Hz, 2H), 1.96 – 1.48 (m, 16H), 1.42 – 1.03 (m, 14H), 0.84 (t, *J* = 6.9 Hz, 3H).

^{13}C{^{1}H} NMR (101 MHz, C_6D_6) δ 143.7*, 113.6*, 98.6, 86.3, 79.9*, 70.1*, 65.8*, 61.6, 37.5, 36.4, 34.7*, 31.9, 31.0, 29.4*, 26.7, 26.6, 26.5, 26.0, 25.4, 23.0, 19.6, 16.0, 14.2.

* These resonances are doubled due to diastereomers from the tetrahydropyranyl (THP) ether protective group.

HRMS (ESI) *m/z*: $[M+Na]^+$ Calcd for $C_{24}H_{40}O_3Na$ 399.2870; Found 399.2863.

(*E*)-vinylic ether 24:

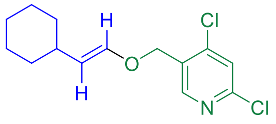

24, 86% yield (DCP), 1.5 h
90% yield (DTBP), 18 h

Using conditions A for 1.5-hour reaction time, the vinylic ether **24** was prepared from alcohol **9h** (55 mg, 0.31 mmol, 1.0 equiv) and vinylic pinacolboronate **8a** (110 mg, 0.47 mmol, 1.5 equiv). The ^{1}H NMR spectrum of the crude reaction mixture in C_6D_6 showed >95% consumption of vinylic pinacolboronate **8a** and presumably quantitative formation of vinylic ether **24**. A small amount of **8a**-derived cumyl vinylic ether was observed (based on similarities in chemical shifts and alkenyl protons' coupling constants to those of cumyl vinylic ether **6**).

The crude product mixture was purified by flash column chromatography with silica gel pre-treated with 2% Et_3N in hexanes (by dry loading on 1.1 g celite), eluting with a gradient of 98:2 – 97:3 – 96:4 – 95:5 hexanes/EtOAc. Fractions were checked with TLC in 90:10 hexanes/EtOAc (visualized with UV light and PMA stain), collected, and concentrated by rotary evaporation, to afford the vinylic ether **24** as a pale-yellow crystalline solid (76 mg, 86% yield).

Using conditions B for 18-hour reaction time, the vinylic ether **24** was prepared on similar scale as above (80 mg, 90% isolated yield). The ^{1}H NMR spectrum of the crude reaction mixture in C_6D_6 showed >95% consumption of vinylic pinacolboronate **8a** and presumably quantitative formation of vinylic ether **24**. No **8a**-derived *t*-butyl vinylic ether was observed in the crude mixture.

Characterization data of compound **24**:

^{1}H NMR (400 MHz, C_6D_6) δ 8.20 (s, 1H), 6.73 (s, 1H), 6.15 (dd, *J* = 12.7, 1.0 Hz, 1H), 4.78 (dd, *J* = 12.7, 7.8 Hz, 1H), 4.24 (s, 2H), 1.83 – 1.72 (m, 1H), 1.66 (d, *J* = 2.9 Hz, 2H), 1.64 (t, *J* = 2.3 Hz, 2H), 1.62 – 1.55 (m, 1H), 1.25 – 1.07 (m, 3H), 1.07 – 0.97 (m, 2H).

^{13}C{^{1}H} NMR (101 MHz, C_6D_6) δ 151.7, 150.0, 144.6, 144.2, 130.1, 124.3, 112.1, 65.3, 37.3, 34.6, 26.5, 26.4.

HRMS (APCI) *m/z*: $[M+H]^+$ Calcd for $C_{14}H_{18}{}^{35}Cl_2NO$ 286.0760; Found 286.0765.

Melting point: m.p. = 46.7-48.7 °C

(*E*)-vinylic ether 25:

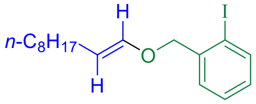

25, 37% yield (DCP), 18 h

Using conditions A for 18-hour reaction time, the vinylic ether **25** was prepared from alcohol **9i** (73 mg, 0.31 mmol, 1.0 equiv) and vinylic pinacolboronate **1** (124 mg, 0.47 mmol, 1.5 equiv). The ^{1}H NMR spectrum of the crude reaction mixture in C_6D_6 showed full consumption of vinylic pinacolboronate **1** and formation of desired vinylic ether **25**. Due to the complexity of the spectrum, no estimated yield was obtained. Other detectable minor products included vinylic acetate **5** and cumyl vinylic ether **6**.

The crude product mixture was purified by flash column chromatography with silica gel pre-treated with 2% Et_3N in hexanes (by dry loading on 1.1 g celite), eluting with a gradient of 100:0 – 98:2 hexanes/EtOAc. Fractions were checked with TLC in 95:5 hexanes/EtOAc (visualized with UV light and PMA stain), collected, and concentrated by rotary evaporation, to afford the vinylic ether **25** as a clear pale-yellow oil at room temperature, or a white crystalline solid when stored in a -20 °C freezer (43 mg, 37% yield).

Note: A similar scale reaction was conducted using **conditions A for 35 minutes**, but we observed low conversion of vinylic pinacolboronate **1**.

Characterization data of compound **25**:

^{1}H NMR (400 MHz, C_6D_6) δ 7.59 (dd, *J* = 7.9, 1.2 Hz, 1H), 7.38 (ddd, *J* = 7.7, 1.7, 0.8 Hz, 1H), 6.97 (td, *J* = 7.5, 1.2 Hz, 1H), 6.53 (td, *J* = 7.6, 1.7 Hz, 1H), 6.28 (dt, *J* = 12.6, 1.3 Hz, 1H), 4.94 (dt, *J* = 12.6, 7.3 Hz, 1H), 4.62 (s, 2H), 1.87 (qd, *J* = 7.2, 1.4 Hz, 2H), 1.28 (m, 14H), 0.92 (t, *J* = 6.9 Hz, 3H).

^{13}C{^{1}H} NMR (101 MHz, C_6D_6) δ 146.2, 140.2, 139.3, 129.3, 128.8, 128.0*, 105.6, 97.3, 75.0, 32.3, 31.1, 29.9, 29.8, 29.5, 28.2, 23.1, 14.4.

* This resonance was obscured by C_6D_6, however HSQC shows correlation with ^{1}H resonance at 6.97 ppm.

HRMS (APCI) *m/z*: $[M+H]^+$ Calcd for $C_{17}H_{26}IO$ 373.1023; Found 373.1041.

(*E*)-vinylic ether 26:

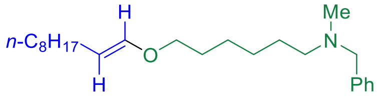

26, **43% yield (DCP), 35 min**
49% yield (DTBP), 18 h

Using conditions A for 35-min reaction time, the vinylic ether **26** was prepared from alcohol **9j** (69 mg, 0.31 mmol, 1.0 equiv) and vinylic pinacolboronate **1** (124 mg, 0.47 mmol, 1.5 equiv). ^{1}H NMR analysis in C_6D_6 of the crude reaction mixture indicated 55% NMR yield of vinylic ether **26**. Other side products observed were protodeboronation alkene product **S1**, diene **S2**, and alcohol **9j**-derived acetate ester.

The crude product mixture was purified by flash column chromatography with silica gel pre-treated with 2% Et_3N in hexanes (by dry loading on 1.1 g celite), eluting with a gradient of 90:10 – 85:15 – 80:20 hexanes/EtOAc. Fractions were checked with TLC in 70:30 hexanes/EtOAc (visualized under UV light and with PMA stain), collected, and concentrated by rotary evaporation to afford the vinylic ether **26** as a yellow oil (48 mg, 43% yield).

Using conditions B for 18-hour reaction time, the vinylic ether **26** was prepared from a similar scale reaction as above (~58% NMR yield; 54 mg, 49% isolated yield).

Characterization data of compound **26**:

^{1}H NMR (400 MHz, C_6D_6) δ 7.38 (broad d, *J* = 7.5 Hz, 2H), 7.22 (broad t, *J* = 7.7 Hz, 2H), 7.12 (tt, *J* = 7.4, 1.4 Hz, 1H), 6.37 (dt, *J* = 12.6, 1.3 Hz, 1H), 4.86 (dt, *J* = 12.6, 7.3 Hz, 1H), 3.50 (t, *J* = 6.5 Hz, 2H), 3.36 (s, 2H), 2.25 (t, *J* = 7.1 Hz, 2H), 2.08 (s, 3H), 1.96 (qd, *J* = 7.2, 1.3 Hz, 2H), 1.55 (tt, *J* = 8.1, 6.5 Hz, 2H), 1.46 – 1.35 (m, 4H), 1.35 – 1.19 (m, 14H), 0.91 (t, *J* = 6.7 Hz, 3H).

^{13}C{^{1}H} NMR (101 MHz, C_6D_6) δ 147.0, 140.4, 129.2, 128.5, 127.2, 103.7, 68.9, 62.9, 57.7, 42.2, 32.3, 31.4, 30.0, 29.8, 29.8, 29.5, 28.4, 27.9, 27.4, 26.4, 23.1, 14.4.

HRMS (ESI) *m/z*: $[M+H]^+$ Calcd for $C_{24}H_{42}NO$ 360.3261; Found 360.3279.

D. Optimization studies

1. Initial extension of previously published Chan-Evans-Lam conditions (ref 12)

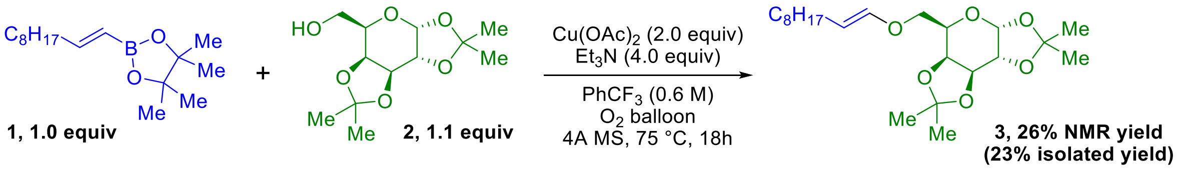

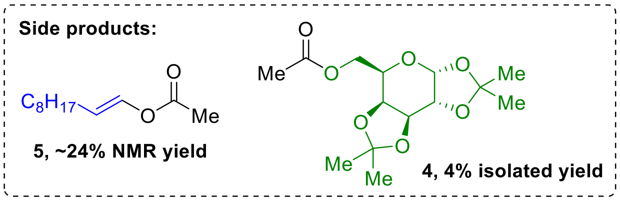

***Experimental protocol**:* Alcohol **2** (1.1 equiv, 89 mg, 0.34 mmol), vinylic pinacolboronate **1** (1.0 equiv, 83 mg, 0.31 mmol), triethylamine (Et_3N, 4.0 equiv, 125 mg, 1.24 mmol), and $Cu(OAc)_2$ (2.0 equiv, 113 mg, 0.62 mmol) were added sequentially to an oven-dried 4 mL reaction vial charged with a stir bar under air. $PhCF_3$ (0.5 mL, 0.6 M based on limiting Bpin) were added to the reaction vial. The vial was capped with a septum cap, and solution was sparged with O_2 balloon for 5 minutes. The punctured septum cap was replaced with a new sealed septum cap, and the vial was sealed with electrical tape. The reaction vial was placed under O_2 atmosphere with an O_2 balloon and stirred at 75 °C overnight. After 18 hours, the reaction was cooled to room temperature and diluted with dichloromethane. The mixture was transferred to a 20 mL vial using DCM to rinse, which was then concentrated by rotary evaporation. A ^1H NMR sample of the crude reaction mixture was measured in C_6D_6 for NMR yield analysis.

Flash column chromatography was conducted as described in the optimized procedures (section B1). Vinylic ether **3** was obtained as a pale-yellow oil (28.5 mg, 23% yield). Acetate ester **4** was obtained as a yellow oil (4 mg, 4% yield). Vinylic acetate **5** was obtained as a pale-yellow oil after rotary evaporation (15 mg, containing hexanes); however, compound **5** was volatile under high-vacuum and only 1.5 mg of side-product **5** remained afterwards.

2. Mechanistic hypotheses for optimization strategies

As the conditions based on ref. 12 gave low yield of vinylic ether **3** and new side product acetate ester **4** with near-stoichiometric combinations of vinylic boronate **1** and alcohol **2** (section D1), we evaluated the mechanistic literature of Cu(II)-catalyzed C–O cross-coupling reactions.

There are significant differences between our desired transformation and published Cu(II)-promoted C-O cross-couplings of organoboronic esters, summarized below:

Literature Chan-Evans-Lam methods	Desired Chan-Evans-Lam method
**Aryl** boron + alcohol **solvent**	**Vinylic** boron + aliphatic alcohol
Simple reactants	Structurally complex reactants
Alcohol reactant as solvent	~ 1 : 1 vinylic boron : alcohol reactants
Aryl boron transmetalation	Vinylic boron transmetalation

Analysis of literature mechanism

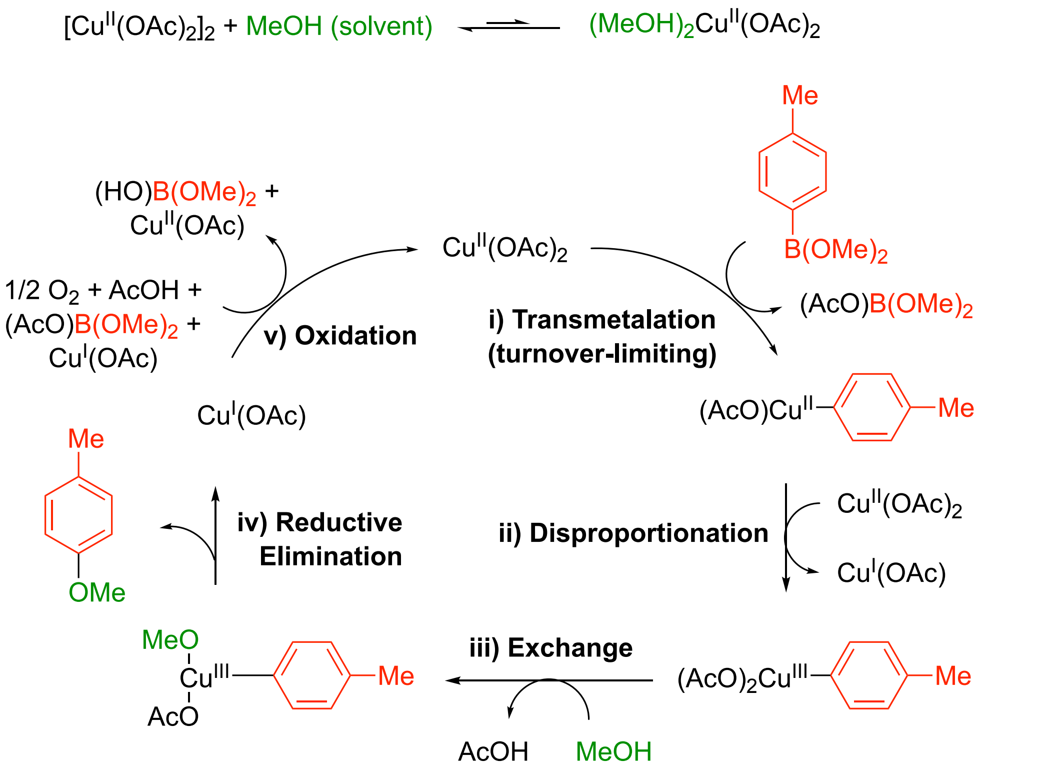

Figure S4. Canonical mechanism for aryl ether synthesis by Cu(II)-catalyzed process, with methanol solvent. This is depicted as in ref. 13, but replacing generic X as OAc, Ar with *p*-tolyl, and showing an implied Cu(III)-methoxide intermediate prior to reductive elimination. Cu species may be solvated with $(MeOH)_n$.

Cu(II)-catalyzed Chan-Evans-Lam-type C-O cross-coupling mechanism was studied by the Stahl laboratory,[13] with *p*-tolyl boronate ester and solvent amount of methanol (**Figure S4**). Dissociation of the catalytically inactive $[Cu(OAc)_2]_2$ paddlewheel preceded the active catalytic cycle and was promoted by Lewis bases, which was the methanol reactant in Stahl case study.[13,14] However, mechanistic studies of Cu(II)-catalyzed C-N cross-couplings indicated that nitrogen-containing ligands also promoted paddlewheel dissociation.[15] The reaction rate was dependent on [Cu] and [arylboronate], indicating that transmetalation was the turnover-limiting step (**step i**). Moreover, the Stahl laboratory suggested that a methoxide-bridged intermediate was involved in the transmetalation step.[13]

To reach a Cu(III) intermediate, the Warren laboratory has studied the Cu(II)-mediated reaction of phenols with triarylborane, observing a conversion ratio of 2 equiv of aryl-Cu(II) complex to give 1 equiv of aryl ether.[16] This is consistent with disproportionation (**step ii**) between a Cu(II)-aryl and an anionic Cu(II)-phenoxide-aryl complex, to give a Cu(III)-aryl-phenoxide complex and a catalytically inactive anionic Cu(I)-aryl. On the other hand, the Stahl laboratory proposed that alcohol exchange (**step iii**) occurred after the Cu(III)-aryl complex formed, possibly due to the stronger Lewis acidity of Cu(III) over Cu(II).[13] Other mechanistic studies demonstrated viable pathways for reductive elimination (**step iv**) through a Cu(III) intermediate to give aryl ether product.[17] The resulting Cu(I) species was re-oxidized to Cu(II) by molecular oxygen and HOAc (**step v**).

Adapting literature mechanism to vinylic boron + aliphatic alcohol, using reaction conditions described in section D.1. **(S-30)**

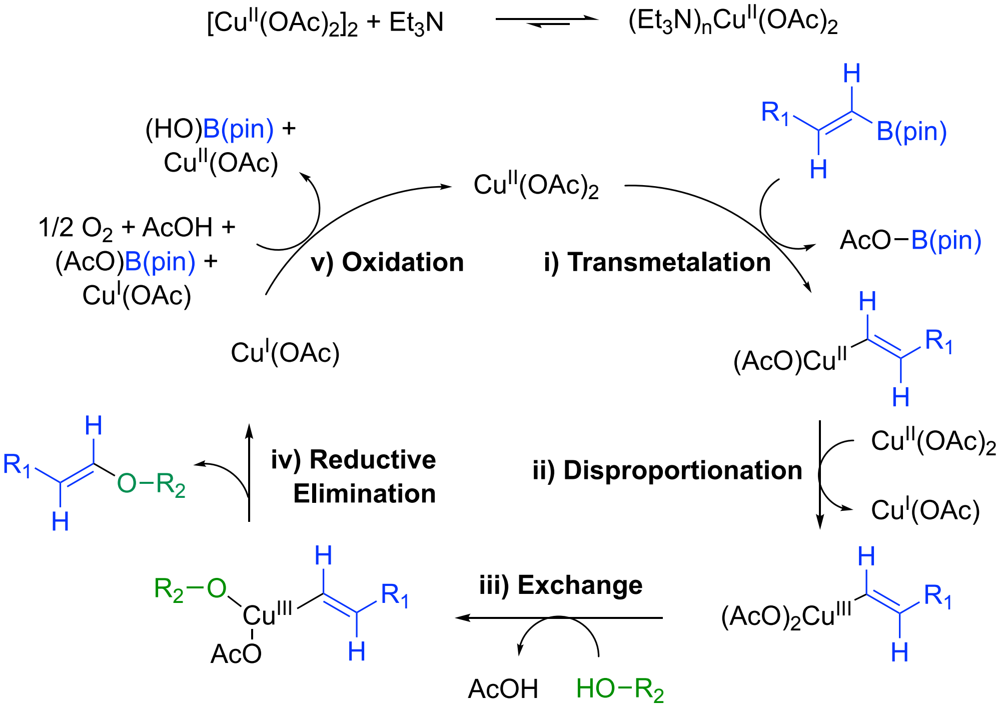

Figure S5. Fitting canonical mechanism to vinylic boron + aliphatic alcohol cross-coupling. Cu species may be ligated with $(Et_3N)_n$.

Considering the differences between literature precedents and our desired reaction scope, we proposed the following optimization strategies:

1. *Paddlewheel dissociation*: As we do not wish to use our alcohol in solvent quantity as the Lewis base to promote dissociation, a suitable Lewis basic ligand was appropriate. Triethylamine as a ligand may have played this role in our initial experiment, however we were aware that *N*-methylimidazole effectively promoted $[Cu(OAc)_2]_2$ paddlewheel dissociation.[18]

2. *Transmetalation*: With a low concentration of a 1° alcohol, we hypothesized that transmetalation may not benefit from alcohol coordination. However, aryl borons undergo transmetalation with the *β*-diketiminate-Cu(II)-O-*t*-Bu complex developed by the Warren laboratory.[16,19,20] This alternative Cu(II) source does not have acetate ligand, which is presumably the source of side products acetate ester **4** and vinylic acetate **5**. We hypothesize that the AcO-B(pin) byproduct of transmetalation is the source of electrophilic acetyl, leading to acetate ester **4**.[13,21]

3. *Cu(II)-to-Cu(III) and alcohol addition*: The mechanism to generate Cu(III)-complex in Chan-Evans-Lam reactions is not yet well-studied, but the consensus has mostly been disproportionation. However, the Warren laboratory has shown that dialkyl peroxides promote Cu(I) to Cu(II) oxidation,[16,20] and Sueki and Kuninobu have used di-*tert*-butyl peroxide (DTBP) in place of O_2 in C-N and C-O cross-couplings with alkylborane reactants.[22] We also hypothesize that a weakly nucleophilic and neutral alcohol reactant is more likely to coordinate to the Lewis acidic Cu(III)-species over Cu(II) for the required ligand exchange.

4. *Reductive elimination:* Precedents indicate that reductive elimination does not readily occur from Cu(II), but is well-established for Cu(III) to Cu(I).[17] As vinylic acetate **5** was a substantial side product, presumably from acetate ligand competing with alkoxide in the reductive elimination step, can we replace the acetate ligand with another ligand?

5. *Catalytic turnover*: Molecular oxygen is the most common oxidant for regenerating Cu(II)-catalyst from the Cu(I) species produced by reductive elimination. However, using an oxygen balloon complicated the reaction set-up and potentially compromised reproducibility. Thus, alternative oxidants were of interest. For example, the Warren laboratory generated the active *β*-diketiminate-Cu(II)-O-*t*-Bu catalyst by single-electron oxidation of the Cu(I)-complex with DTBP.[16,19,20] We hypothesized that dialkyl peroxides may not only improve catalytic turnover, but also generate a catalytically active pre-transmetalation complex. These oxidants may also potentially change the mechanism of Cu(II)-to-Cu(III) transformation.

3. Reaction optimization studies (0.31 mmol scale, 0.6 M reaction concentration based on alcohol 2 as limiting reagent)

Table S1. Optimization table

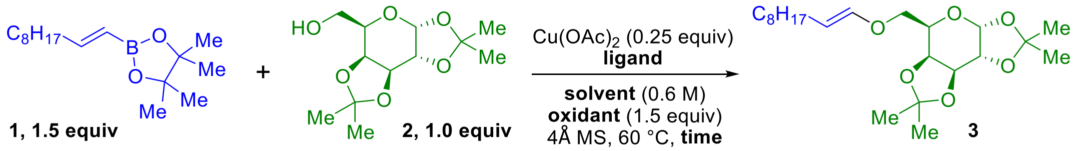

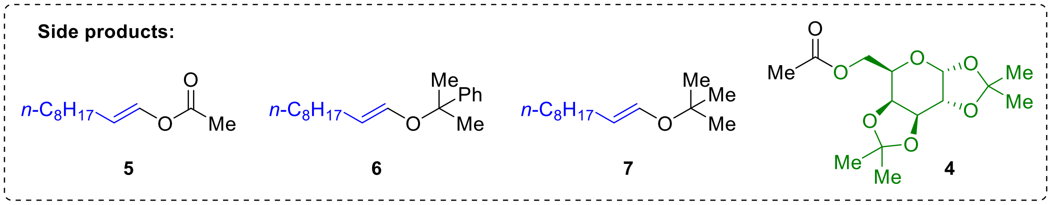

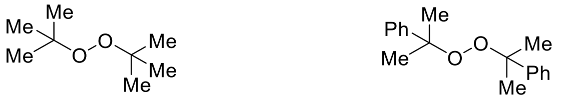

di-*tert*-butylperoxide (DTBP) **dicumyl peroxide (DCP)**

Entry[a]	Ligand (equiv)	Oxidant	Solvent	Time	3	4	5	6
1	NPI (0.50)	O_2 (balloon)	MeCN	18 h	50%	12%	<2%	N.A.
2	NPI (0.50)	DTBP	DCM	18 h	70% (67%)	24%	N.D.	N.A.
3	NPI : *t*BuOH (0.50 : 1.0)				75% (71%)	25% (29%)	<2%	N.A.
4	NPI : *t*BuOH (0.50 : 1.0)	DTBP	$PhCF_3$	18 h	72% (71%)	21% (18%)	6%	N.A.
5				75 min	72%	16%	10%	N.A.
6	**NPI : *t*BuOH (0.50 : 1.0)**	**DCP**	**$PhCF_3$**	18 h	85% (84%)	8%	6%	10%
7				60 min	81%	7%	6%	11%
**8**				**35 min**	**84% (80%)**	**9%**	**5%**	**13%**

N.D. = not detected; N.A. = not applicable

a. ^{1}H NMR yields estimated as described in section B4 (isolated yields in parentheses); vinylic pinacolboronate **1** was fully consumed; contained traces of *t*-butyl vinylic ether **7** (<5% NMR yield).

Table S2. Additional experiments

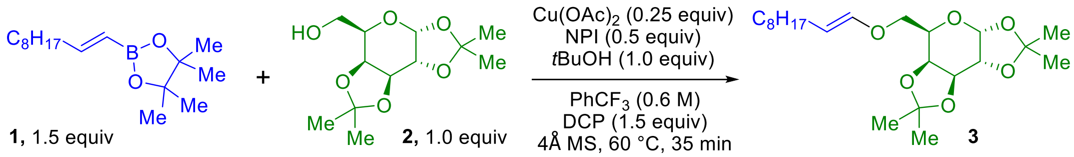

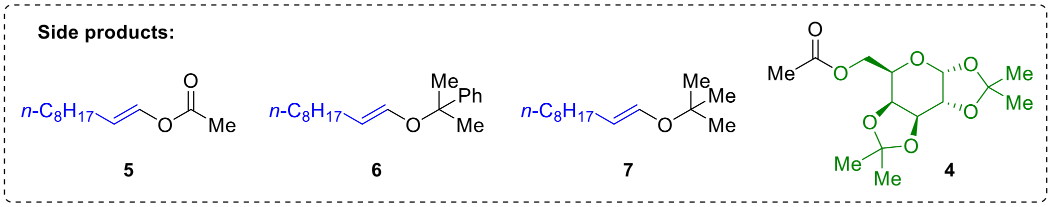

Entry[a]	Changes from optimal conditions	3	4	5	6	Conversion of 1
**1**	**None**	**84% (80%)**	**9%**	**5%**	**13%**	**100%**
2	Cu(OAc)$_2$ (0.14 equiv)<br>NPI (0.28 equiv)<br>35 min reaction time	76%	9%	6%	7%	100%
3	1.3 equiv of vinylic pinacolboronate **1**<br>20 min reaction time	68%	10%	5%	3%	86%
4	1.2 equiv of DCP<br>20 min reaction time	63%	18%	7%	<1%	60%

a. ^{1}H NMR yields estimated as described in section B4 (isolated yields in parentheses); contained traces of *t*-butyl vinylic ether **7** (<3% NMR yield).

Summary of optimization study (Table S1 and S2): Systematic screening revealed that NPI ligand and *t*-BuOH additive enabled high yields of vinylic ether **3**. Oxidant screening revealed significant improvement when switching from molecular oxygen to dialkyl peroxides, especially with DCP oxidant.[20] DCP oxidant enabled higher yields of vinylic ether **3**, reduced the *O*-acylation reaction producing acetate ester **4**, and proceeded more rapidly than with DTBP. However, we observed measureable formation of cumyl vinylic ether **6**, in addition to traces of *t*-butyl vinylic ether **7** (coming from *t*-BuOH and/or DTBP). Attempts to further decrease catalytic loading or oxidant loading conditions resulted in less efficient cross-coupling processes (**Table S2**, entries 2 - 4).

4. Other optimization studies (0.31 mmol scale, 0.6 M reaction concentration based on limiting reagent)

Table S3. Ligand screening (under oxygen atmosphere)

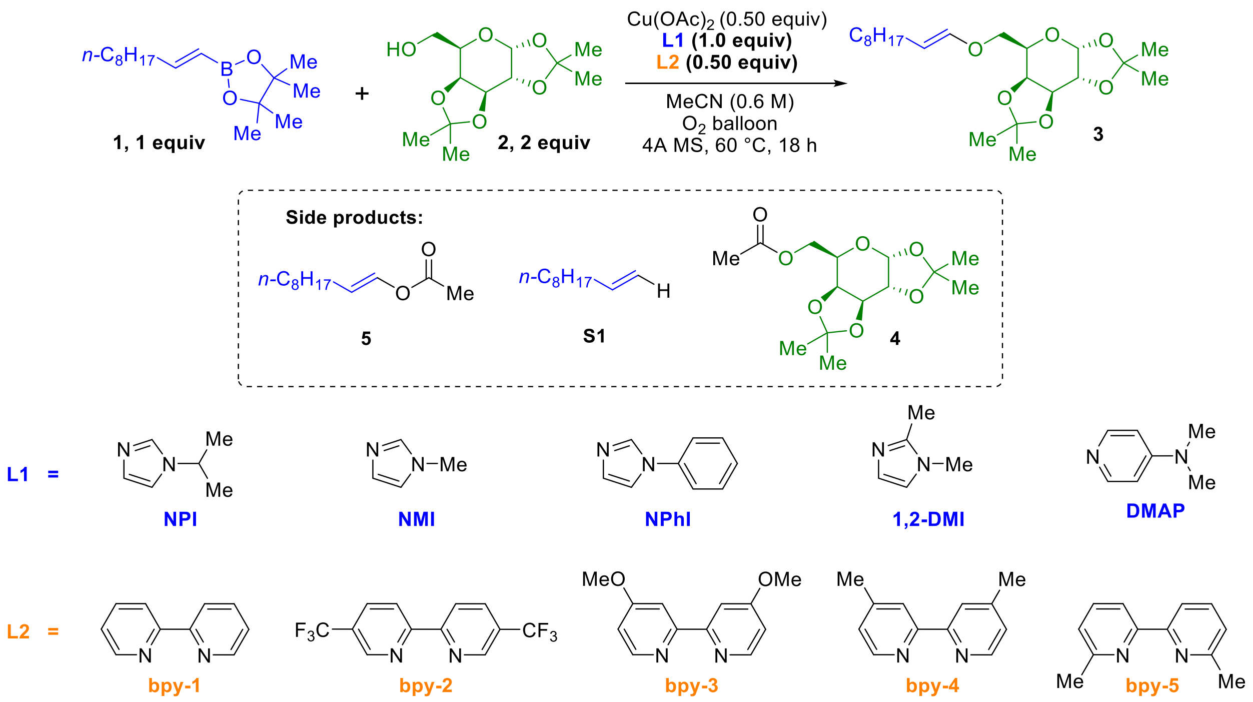

Entry[a]	L1	L2	3	4	5	S1	Conversion of 1
1	NPI	bpy-1	45% (45%)	12%	<2%	<2%	100%
2[b]	NMI		43%	16%	<2%	<2%	100%
3	NPhI		46%	15%	5%	<2%	100%
4	1,2-DMI		21%	16%	<2%	<2%	100%
5	NPI	bpy-2	47% (46%)	N.D.	<2%	<2%	100%
6		bpy-3	15%	N.D.	<2%	<2%	25%
7		bpy-4	45%	13%	11%	<2%	95%
8		bpy-5	27%	20%	8%	<2%	45%
9	NPI	bpy-1[c]	51%	15%	<2%	<2%	100%
10	DMAP		32%	17%	<2%	<2%	100%
**11**	**NPI**	**None**	**50%**	**12%**	**<2%**	**<2%**	**100%**
12	None	bpy-1	33%	20%	9%	8%	87%

a. ^{1}H NMR yields estimated as described in section B4 (isolated yields in parentheses).
b. Observed 16% NMR yield of 1-decanal.
c. 0.10 equiv of bpy-1 was used.

Summary of ligand screening (Table S3): We hypothesized that a combination of bipyridine and substituted imidazole ligands may promote $Cu(OAc)_2$ paddlewheel dissociation *and* also increase reactivity of a monomeric Cu(II) complex, inspired in part by Cu(I)-catalyzed aerobic oxidation of aliphatic alcohols.[23,24] However, this study revealed that *N*-alkylimidazoles without bipyridine ligands improved the yield of vinylic ether **3**, and decreased competing formation of vinylic acetate **5**. We selected *N*-isopropylimidazole (NPI) over *N*-methylimidazole (NMI) for further development, primarily because we more accurately measured small quantities of NPI.

Table S4. Oxidant screening:

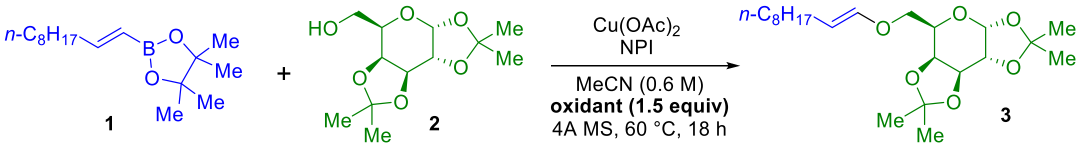

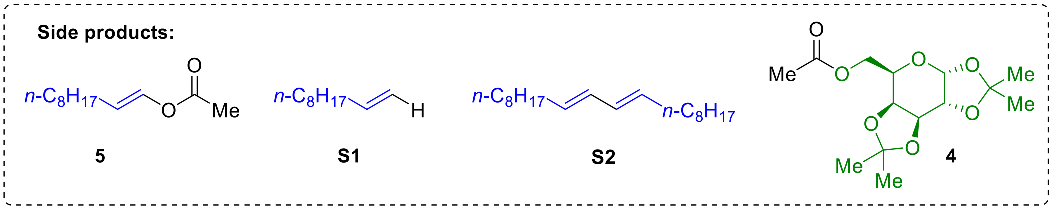

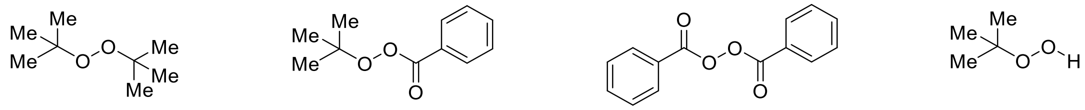

di-*tert*-butylperoxide (DTBP) | ***t*-BuOOBz** | **BzOOBz** | ***t*-BuOOH**

Entry [a]	**1** : **2** equiv	Cu : NPI equiv	Oxidant	**3**	**4**	**5**	**S1**	Conversion of **1**
1	1.0 : 2.0	0.50 : 1.0	O_2 (balloon)	45% (45%)	12%	<2%	<2%	100%
2	1.0 : 2.0	0.25 : 0.50	DTBP	60% (62%)	15%	<2%	<2%	100%
3		0.10 : 0.20		45%	18%	<2%	8%	78%
4	1.0 : 1.0	**0.25 : 0.50**	**DTBP**	46%	17%	<2%	<2%	100%
**5**[b]	**1.5 : 1.0**			**65% (66%)**	**15%**	**3%**	**5%**	**100%**
6	1.5 : 1.0	0.25 : 0.50	*t*-BuOOBz	No conversion of pinacolboronate **1**				
7			BzOOBz	No conversion of pinacolboronate **1**				
8			*t*-BuOOH[c]	16%	~50%	8%	7%	100%

a. ^1H NMR yields estimated as described in section B4 (isolated yields in parentheses).
b. Traces of *t*-butyl vinylic ether **7** were observed (<1% NMR yield).
c. *t*-BuOOH as a 5.0 – 6.0 M solution in decane (purchased from Sigma Aldrich).

Summary of oxidant screening (Table S4): We found that using O_2 balloon complicated reaction set-up and compromised reproducibility of our experiment, we opted for organic peroxides. As shown in table S4, DTBP improved vinylic ether yields at lower Cu-ligand loading and a ratio of 1.5 : 1.0 vinylic pinacolboronate **1** : alcohol **2**. Organic peroxyesters such as *t*-BuOOBz or BzOOBz inhibited catalytic activity altogether. This suggests that dialkyl peroxides do improve the reaction in two ways, improving catalytic turnover as well as regenerating a highly reactive pre-transmetalation complex.

Table S5. Vinylic boronate ester screening (with DTBP oxidant):

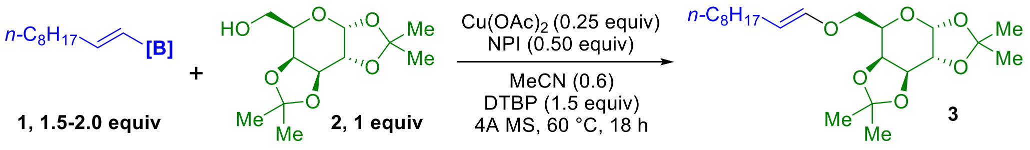

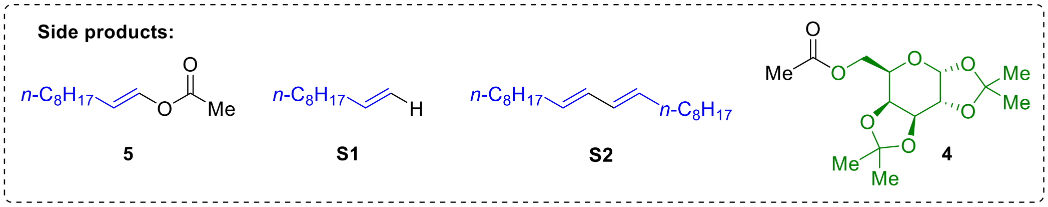

Entry[a]	Vinylic boronate ester	3	4	5	Conversion of **1**
**1**[b]	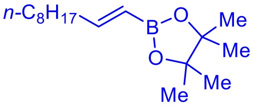	**65% (66%)**	**15%**	**3%**	**100%**
2	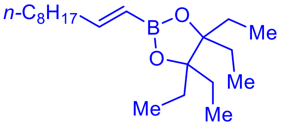	11%	<2%	<5%	<10%
3	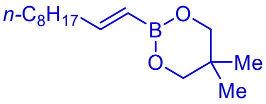	38%	14%	20%	100%
4	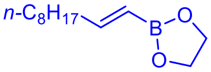	20%	<2%	22%	90%

a. ^1H NMR yields estimated as described in section B4 (isolated yields in parentheses). Side product **S1** and **S2** were formed in negligible amounts.

<u>*Note*</u>: Initial screening (using conditions similar to **Table S3**, entry 1) also explored (*E*)-dec-1-en-1-yl trifluoroborate potassium salt (BF_3K) as the boron source. However, vinylic ether **3**'s yield was lower despite full conversion of the trifluoroborate salt. We did not revisit reaction optimization with trifluoroborate salt with the later-developed conditions. We also screened vinylic boronic *N*-methyliminodiacetic acid (MIDA) ester but only observed catalyst inhibition.[25]

Summary of vinylic boronate ester screening (Table S5): For Pd-catalyzed Suzuki cross-couplings, different cyclic boronate ring sizes and substitution patterns significantly affect transmetalation rates.[26] When testing this hypothesis for our Cu(II)-catalyzed system, we found that sterically encumbered vinylic boron (entry 2) was significantly less reactive.[27] Less sterically encumbered vinylic boronate esters gave lower yields of vinylic ether **3**, with substantial formation of vinylic acetate **5** (entries 3, 4). We concluded that transmetalation rates were affected by the boronate ester types, and vinylic pinacolboronate **1** was most compatible with the weak nucleophilicity and lower reactivity of alcohol **2**.

Table S6. Study on solvent effects (with DTBP oxidant):

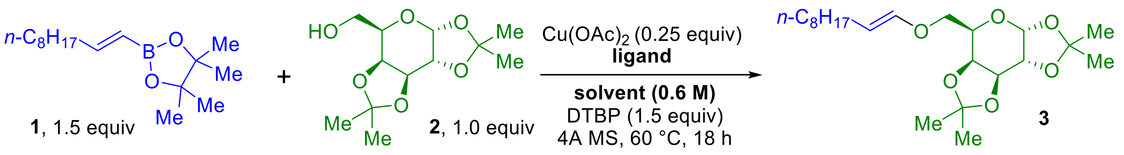

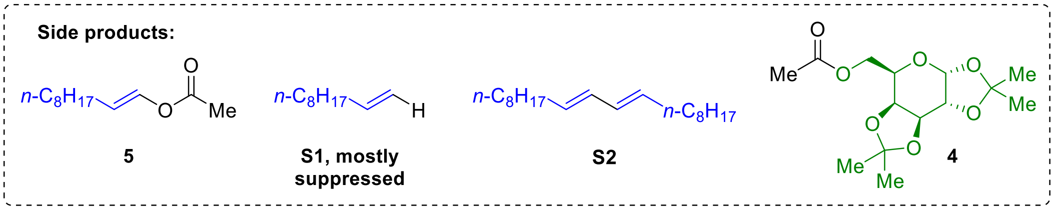

Entry[a]	Ligand (equiv)	Solvent	3	4	5	S2	Conversion of 1
1	none	MeCN	11%	44%	26%	<2%	60%
2	none	NPI	>80% conversion of Bpin, complex mixture containing trace of vinylic ether **3**				
3	NPI (0.50)	PhCN[b]	45%	30%	15%	<2%	100%
4[c]		DCE	60%	26%	<2%	10%	100%
**5[c]**	**NPI (0.50)**	**DCM**	**70% (67%)**	**24%**	**N.D.**	**10%**	**100%**
6[c]	DMAP		48%	38%	<2%	8%	100%

N.D. = not detected.
a. ^{1}H NMR yields estimated as described in section B4 (isolated yields in parentheses).
b. Non-anhydrous benzonitrile (PhCN) was used.
c. Traces of *t*-butyl vinylic ether **7** were observed (<1% NMR yield).

<u>*Note*</u>: We also tested tetrahydrofuran (THF) as the reaction solvent with preliminary reaction conditions (similar to **Table S3**, entry 1) and observed comparable results to MeCN solvent. However, THF did not fully dissolve the reactants, thus we stopped pursuing it as a solvent.

Summary of solvent effects (Table S6): Non-coordinating polar solvents, specifically halogenated solvents, were more beneficial to the reaction than acetonitrile or benzonitrile.

Table S7. Cu source screening (with DTBP oxidant)

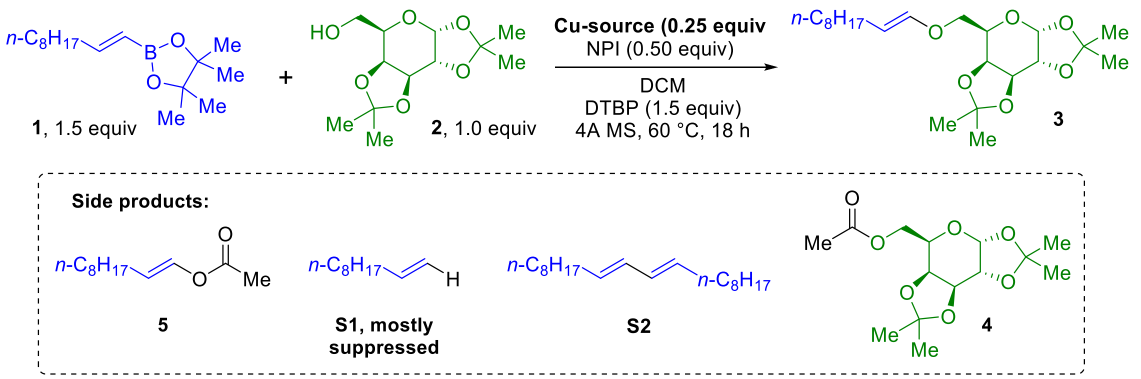

Entry[a]	Cu source	3	4	5	S2	Conversion of **1**
**1**	**$Cu(OAc)_2$**	**70% (67%)**	**24%**	**N.D.**	**10%**	**100%**
2	$Cu(OAc)_2$•H_2O	55%	10%	7%	<2%	100%
3	$Cu(OTf)_2$	30% (28%)	[b]	N.A.	<2%	95%
4	$[Cu(MeCN)_4]PF_6$	41%	22%[c]	N.D.	3%	100%
5[d]	$CuCl_2$•$(H_2O)_2$	8%	42%	N.A.	<5%	100%
6	CuF_2 (in MeCN solvent)	5%	56%[c]	N.D.	<2%	100%

N.D. = not detected; N.A. = not applicable

a. ^{1}H NMR yields estimated as described in section B4 (isolated yields in parentheses).

b. ^{1}H NMR of crude mixture showed possible triflation of galactose-derived alcohol **2** (based on similar deshielding effect observed with acetate ester **4**); however, only alcohol **2** (~60%) was recovered due to possible hydrolysis of *O*-triflate side product on silica gel column chromatography.

c. ^{1}H NMR of crude mixture showed ^{1}H NMR signals of acetate ester **4**; source of acetate may have originated from acetonitrile ligands.

d. 1,2-disubstituted vinylic chloride was observed as the major cross-coupling product; 1-decene **S1** was also obtained in 21% NMR yield based on consumption of compound **1**.

<u>*Note:*</u> In an earlier experiment with $Cu(OTf)_2$ catalyst and THF solvent (similar conditions to **Table S3**, entry 1, under O_2 atmosphere), vinylic ether product was not formed despite partial consumption of vinylic pinacolboronate **1**.

Summary of Cu-source screening (Table S7): Anhydrous $Cu(OAc)_2$ was most effective. Other alternatives reported for Chan-Evans-Lam C-heteroatom cross-couplings[14,28-30] were subpar in comparison, giving significantly lower yields of vinylic ether **3** despite full conversion of vinylic pinacolboronate **1**. We observed traces of possible sulfonylation and acetylation of alcohol **2** with $Cu(OTf)_2$ and $[Cu(MeCN)_4]PF_6$, respectively. It appears that MeCN (ligand or solvent) contributed to formation of acetate ester **4**, mediated by Cu(II) as a Lewis acid, even in the absence of $Cu(OAc)_2$. Additionally, unlike O_2 oxidant, we hypothesized that DTBP oxidant enables catalytic activity of $Cu(OTf)_2$ for the C-O cross-coupling of vinylic pinacolboronate **1** with alcohol **2** through the formation of a Cu(II)-O-*t*-Bu species, which may mediate an effective transmetalation step.[16]

Table S8. Initial attempts to synthesize *Z*-vinylic ether (**15**)

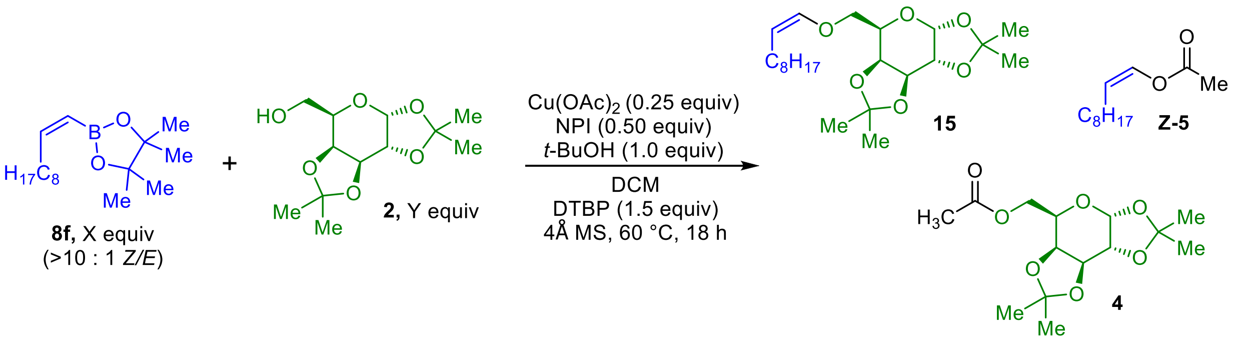

Entry[a]	X equiv	Y equiv	15[a]	4	(Z)-5	Conversion of 1
1	1.5	1.0	17%[b]	~50%	N.D.	100%
2	1.0	2.0	31%[c]	33%	N.D.	100%

a. Vinylic ether **15** was obtained as a >10 : 1 *Z/E* mixture, as observed in the ^{1}H NMR spectra of the crude reaction mixtures.
b. NMR yields were calculated based on alcohol **2** as the limiting reagent.
c. NMR yields were calculated based on *Z*-vinylic pinacolboronate **8f** as the limiting reagent.

Note: Compound **8f** was synthesized following the literature protocol.[33] Characterization by ^{1}H NMR showed a >10 : 1 *Z/E* ratio.

Discussion: Initial attempts to apply our *E*-specific cross-coupling method (with DTBP) for *Z*-vinylic ether synthesis resulted in appreciable yields of vinylic ether **15**. We observed full consumption of vinylic pinacolboronate **8f** in both cases (whether in slight excess or as limiting reagent) as well as substantial formation of acetate ester **4**. These results indicated that effective transmetalation occurred, but we were unable to account for the full mass balance. We hypothesize that steric hindrance of the *Z*-vinylic-Cu(II) complex may hamper the rate of reactant alcohol ligand exchange. This may explain the increased yield of vinylic ether **15** when vinylic boronate **8f** is the limiting reactant.

E. Syntheses of other vinylic borons

(*E*)-vinylic boronic acid S3

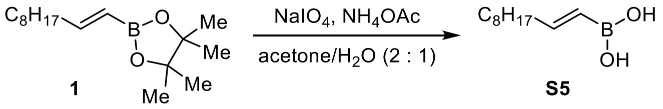

Compound **S3** was synthesized from compound **1** (800 mg, 3.0 mmol) according to the published procedure for a similar compound (i.e. (*E*)-oct-1-en-1-ylboronic acid[32]). The crude product mixture was obtained as a yellow oil (555 mg). The mixture was analyzed by thin layer chromatography (95:5 hexanes/EtOAc) to show mostly full conversion of vinylic pinacolboronate **1**, and the reaction was assumed to be quantitative. Crude compound **S3** was used in subsequent reactions without further purification.

General protocols for synthesizing vinylic boronate esters S4-S6

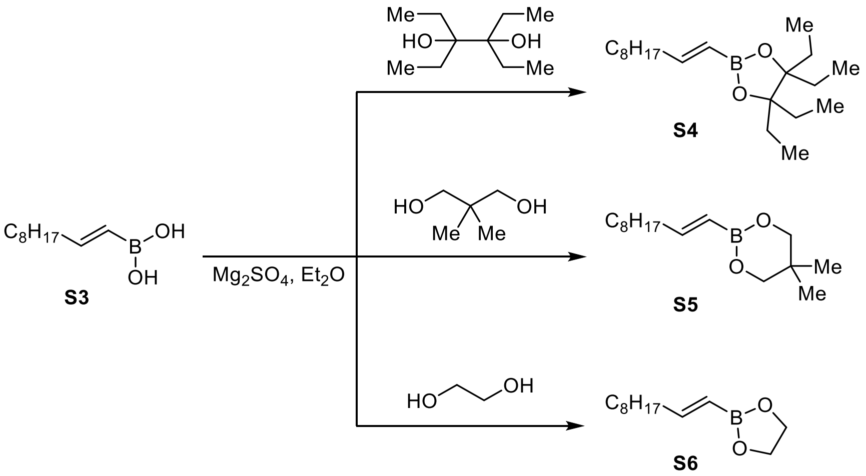

Crude compound **S3** (1.0 equiv) was added to a 20 mL vial charged with stir bar and dissolved with non-anhydrous diethyl ether (0.5 M based on mmol of **S3**). Anhydrous Mg_2SO_4 (2.0 equiv) and diol (1.1 equiv) were added subsequently to the reaction mixture under air. The reaction vial was sealed with a screw cap and stirred at room temperature under ambient atmosphere overnight. After 18 hours, the reaction mixture was filtered over a celite plug (using diethyl ether to rinse vial and wash the celite pad). The resulting light-yellow liquid was concentrated by rotary evaporation to give a dark yellow oil with precipitate. The crude mixture was redissolved in diethyl ether and re-filtered to remove the precipitate. After concentration, the crude product was obtained as a yellow oil and used in further reactions without purification. ^{1}H NMR analysis in C_6D_6 indicated 90~92% purity.

(*E*)-vinylic boronate ester S4

The title crude compound **S4** (320 mg) was synthesized according to the general protocol from crude **S3** (267 mg, 1.45 mmol). ^{1}H NMR characterization was measured from crude mixture.

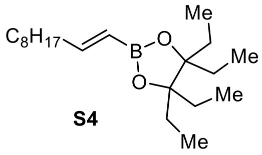

S4

^{1}H NMR (400 MHz, C_6D_6) δ 7.00 (dt, *J* = 17.9, 6.6 Hz, 1H), 5.84 (dt, *J* = 17.9, 1.6 Hz, 1H), 2.13 – 2.04 (m, 2H), 1.69 (dq, *J* = 15.0, 7.5 Hz, 4H), 1.54 (dq, *J* = 14.3, 7.9 Hz, 5H), 1.48 – 1.37 (m, 2H), 1.30 (dt, *J* = 21.1, 6.9 Hz, 5H), 1.18 (d, *J* = 8.1 Hz, 8H), 0.88 (td, *J* = 7.5, 3.1 Hz, 17H).

(*E*)-vinylic boronate ester S5

The title crude compound **S5** (360 mg) was synthesized according to the general protocol from crude **S3** (276 mg, 1.5 mmol). ^{1}H NMR characterization was measured from crude mixture.

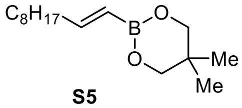

S5

^{1}H NMR (400 MHz, C_6D_6) δ 7.03 (dt, *J* = 17.6, 6.7 Hz, 1H), 5.87 (dt, *J* = 17.6, 1.6 Hz, 1H), 3.35 (s, 4H), 2.22 – 2.11 (m, 2H), 1.46 – 1.34 (m, 2H), 1.31 – 1.15 (m, 11H), 0.89 (t, *J* = 7.0 Hz, 3H), 0.59 (s, 6H).

(*E*)-vinylic boronate ester S6

The title crude compound **S6** (320 mg) was synthesized according to the general protocol from crude **S3** (276 mg, ~1.5 mmol). ^{1}H NMR characterization was measured from crude mixture.

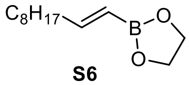

S6

^{1}H NMR (400 MHz, C_6D_6) δ 7.01 (dt, *J* = 17.8, 6.6 Hz, 1H), 5.82 (dt, *J* = 17.9, 1.6 Hz, 1H), 3.61 (s, 4H), 2.14 – 2.02 (m, 2H), 1.35 – 1.15 (m, 14H), 0.92 – 0.87 (m, 3H).

F. Mechanistic experiments

1. Time studies by ^{1}H NMR spectroscopy of aliquots:

a. *With DTBP as oxidant:*

vinylic Bpin 1, 1.5 equiv + **alcohol 2**, 1.0 equiv → [Cu(OAc)$_2$ (0.25 equiv), NPI (0.50 equiv), *t*-BuOH (1.0 equiv) / **DTBP** (1.5 equiv), PhCF$_3$, 4Å MS, 60 °C] **vinylic ether 3** + **acetate ester 4**, **vinylic acetate 5**

Protocol for reaction set-up: Alcohol **2** (81 mg, 0.31 mmol, 1.0 equiv), NPI (18 µL, 0.16 mmol, 0.50 equiv), *t*-BuOH (30 µL, 0.31 mmol, 1.0 equiv), di-*tert*-butyl peroxide (DTBP, 126 mg, 0.47 mmol, 1.5 equiv), Cu(OAc)$_2$ (14 mg, 0.08 mmol, 0.25 equiv), and vinylic pinacolboronate(Bpin) **1** (124 mg, 0.47 mmol, 1.5 equiv) were added sequentially to a flame-dried 4 mL reaction vial (charged with a stir bar and 110-115 mg of 4Å MS) under air. The vial was sealed with a septum cap and sparged with argon. PhCF$_3$ (0.5 mL, 0.6 M based on mmol of **2**) was added to the reaction vial. The vial was stirred at 60 °C on a pre-heated heating block under a stream of argon. Aliquots were taken directly from the heated stirring mixture (by needle and syringe, 0.02-0.05 mL/aliquot) at 10-, 20-, 30-, 45-, 60-, and 75-minute time points and transferred into 20 mL vial (using DCM to wash the needle). Each aliquot was concentrated by rotary evaporation and dissolved in C_6D_6 for ^{1}H NMR analysis. ^{1}H NMR showed full conversion of vinylic pinacolboronate **1** at 75 minutes. ^{1}H NMR integrations of vinylic pinacolboronate **1** (5.83 ppm), vinylic ether **3** (6.34 ppm and 4.86 ppm), acetate ester **4** (5.48 ppm and 4.22 ppm distinctly detectable), and vinylic acetate **5** (5.41 ppm) were calibrated using the anomeric protons' resonances of alcohol **2**-derived materials (~5.5 ppm). We assume that the initial ratio of vinylic pinacolboronate **1** and alcohol **2** at 0-minute time point was approximately 1.5 : 1.0.

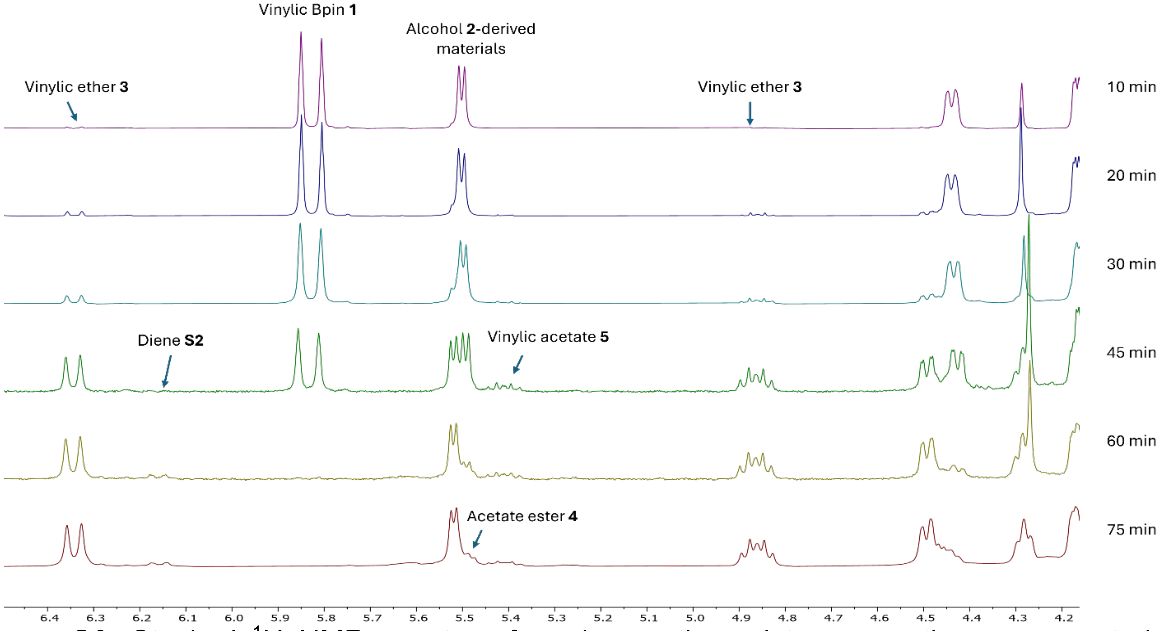

Figure S6. Stacked ^{1}H NMR spectra of crude reaction mixture over time course study for Cu(OAc)$_2$/NPI-catalyzed C-O cross-coupling of **1** + **2**, with DTBP oxidant.

b. With DCP as oxidant:

vinylic Bpin 1, 1.5 equiv + **alcohol 2**, 1.0 equiv → [$Cu(OAc)_2$ (0.25 equiv), NPI (0.50 equiv), *t*-BuOH (1.0 equiv) / **DCP** (1.5 equiv), $PhCF_3$, 4Å MS, 60 °C] → **vinylic ether 3**, **acetate ester 4**, **vinylic acetate 5**, **vinylic cumyl ether 6**

Protocol for reaction set-up: The experiment was performed similar to the protocol described for the NMR experiment above, albeit with DCP (126 mg, 0.47 mmol, 1.5 equiv). Aliquots were analyzed at 3.0-, 6.0-, 10.0-, 12.3-, 15.0-, 17.3-, 20.0-, 22.3-, 25.0-, 27.3-, and 30.0-minute time points.

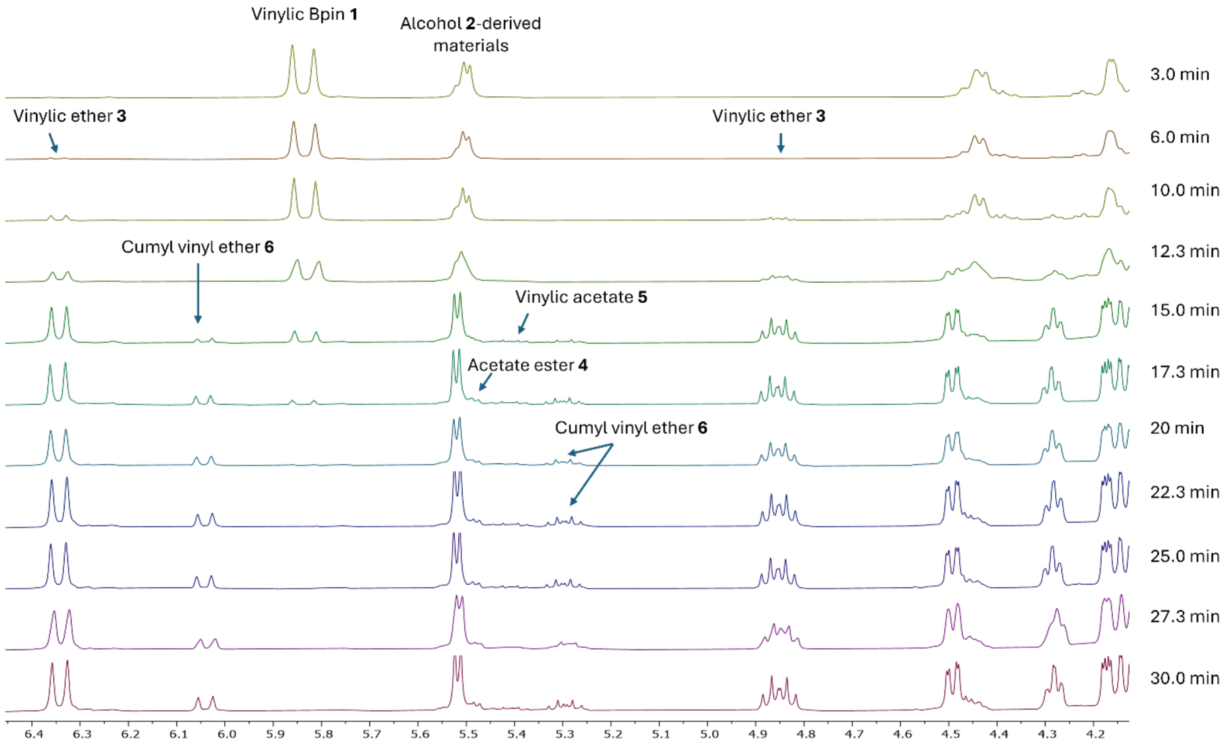

Figure S7. Stacked NMR spectra of crude mixture over reaction time course study for $Cu(OAc)_2$/NPI-catalyzed C-O cross-coupling of **1** + **2**, with DCP oxidant.

c. Results and discussion

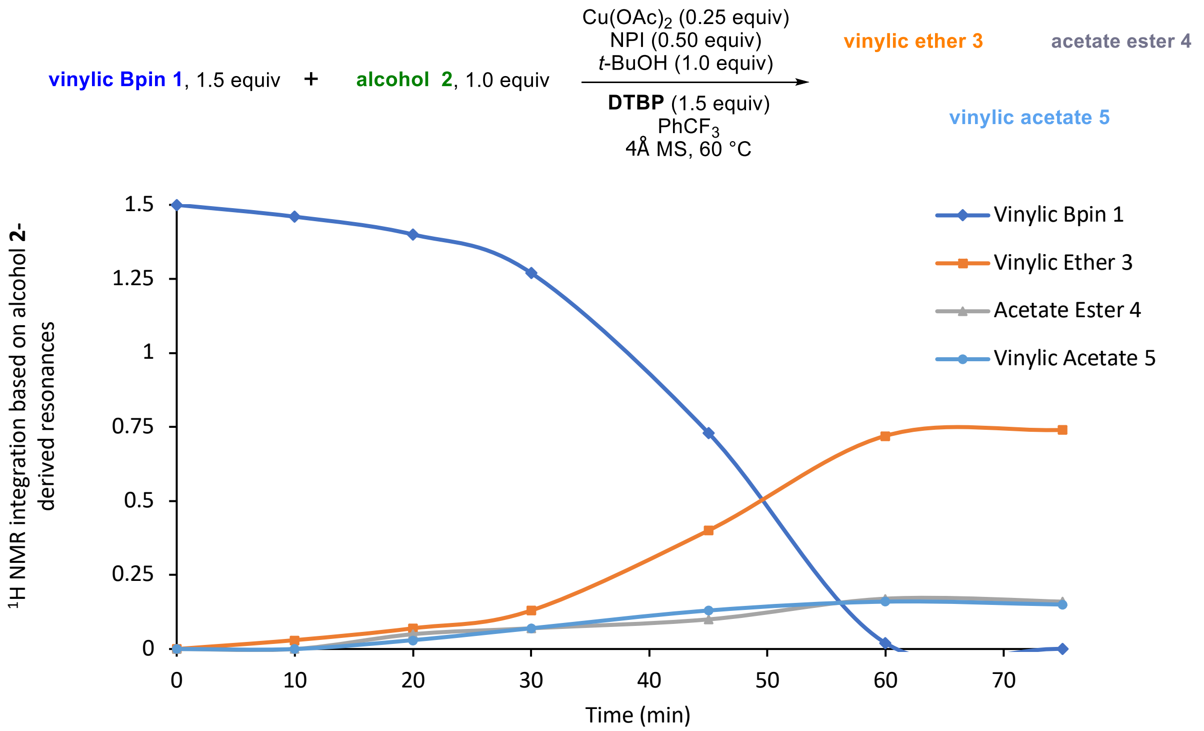

Figure S8. Time course of $Cu(OAc)_2$/NPI-catalyzed C-O cross-coupling of **1+2** with **DTBP** oxidant.

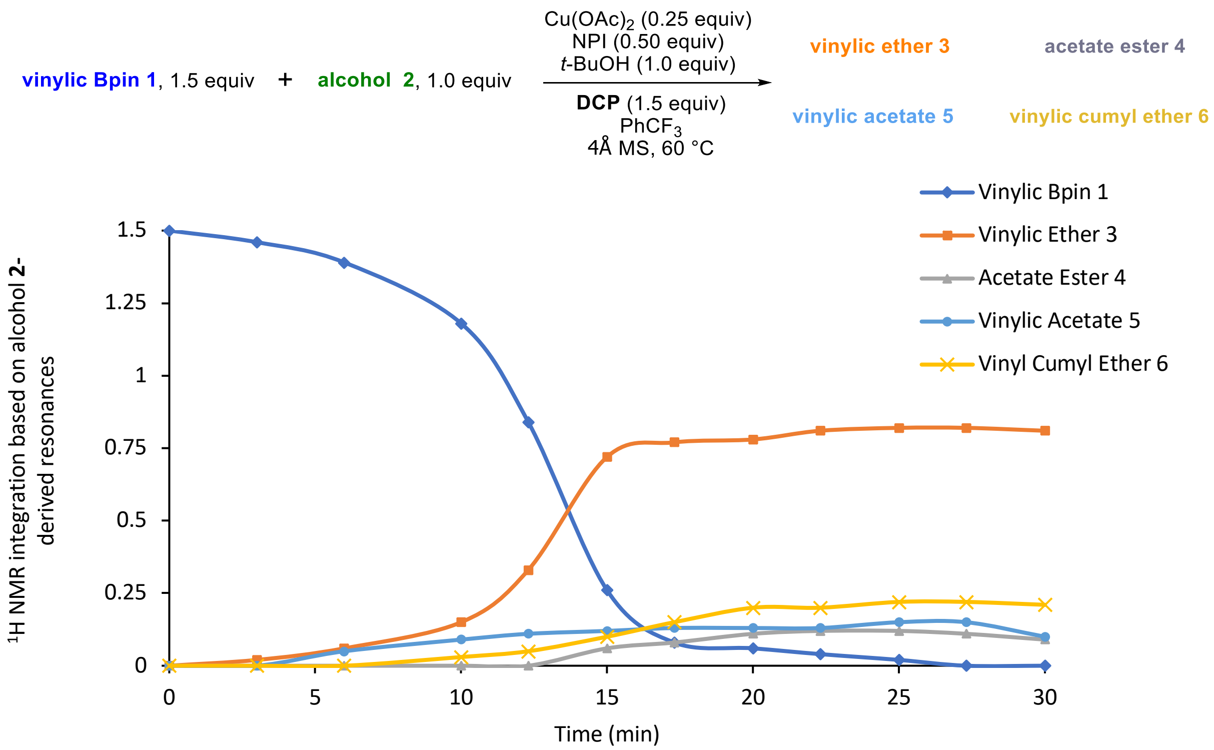

Figure S9. Time course of $Cu(OAc)_2$/NPI-catalyzed C-O cross-coupling of **1+2** with **DCP** oxidant.

Both reaction systems (either with DTBP or DCP) proceeded in relatively short reaction times (30-70 minutes) (**Figures S8**, **S9**). The DCP oxidant consumed vinylic pinacolboronate **1** approximately 2x faster than DTBP, indicating that DCP was more efficient at oxidatively regenerating the active catalytic Cu-species. This increased rate correlates with DCP having a weaker O-O bond than DTBP.[31] Overall, the desired vinylic ether formation outcompeted all other side pathways. We observed a ~2 : 1 ratio of consumed vinylic pinacolboronate **1** and vinylic ether **3**, thus justifying the slight excess amount of vinylic pinacolboronate **1**. Gradual formation of acetate ester **4** side product was observed only as vinylic pinacolboronate **1** was depleted (**Figure S10**). We hypothesized that alcohol **2** underwent the side acylation pathway with AcO-Bpin[13,21] (proposed as a by-product of transmetalation in the initial catalytic cycle), for which NPI may have served as an acylation catalyst.

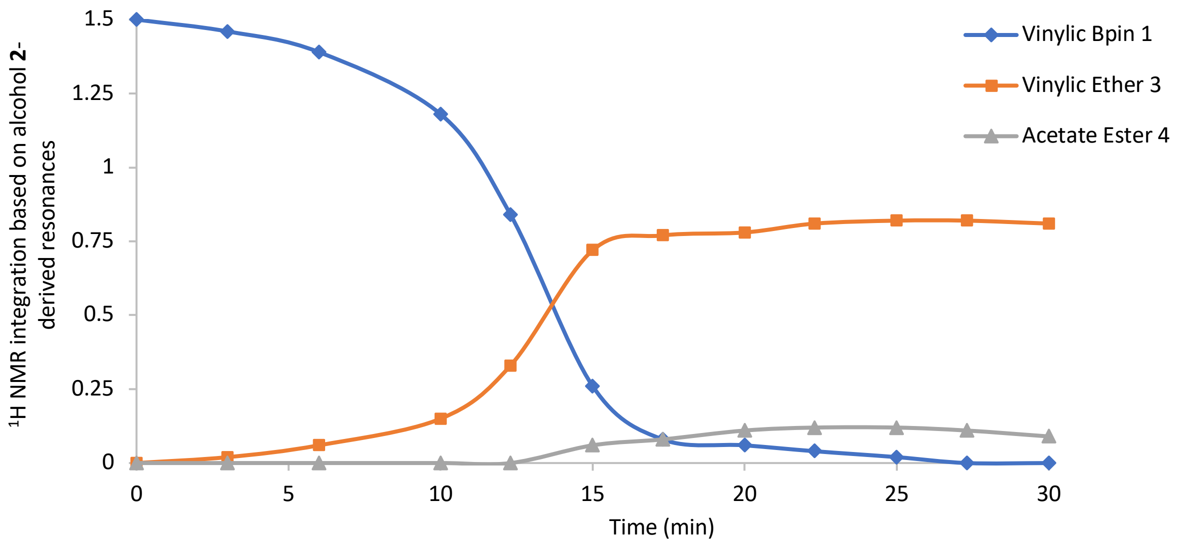

Figure S10. Simplified time course of $Cu(OAc)_2$/NPI-catalyzed C-O cross-coupling of **1+2** with **DCP** oxidant (showing only consumption of vinylic pinacolboronate **1** and formation of compounds **3** and **4**).

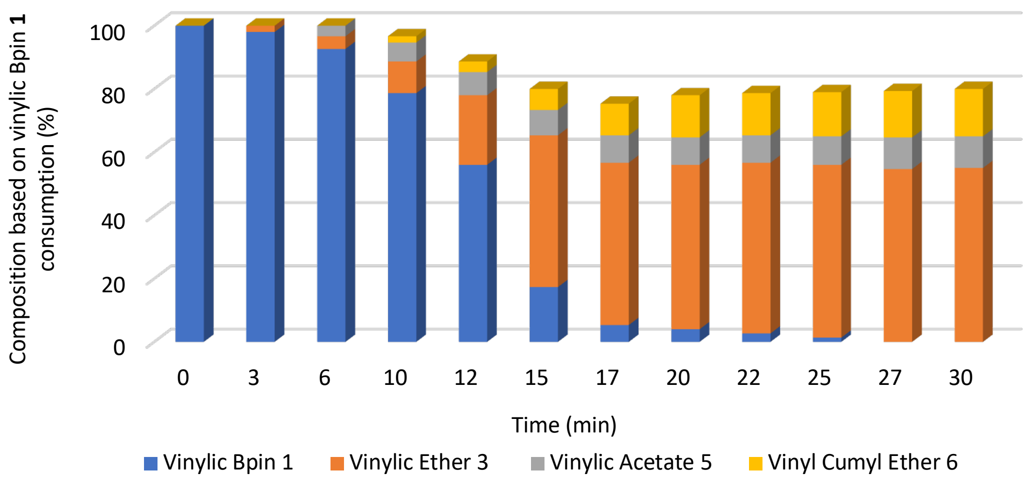

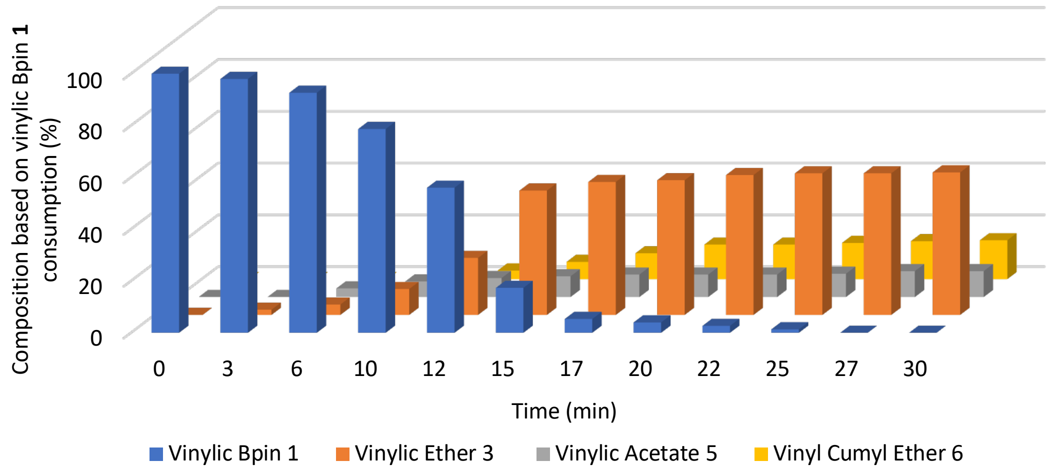

Figure S11. 2D stacked column chart (top) and 3D column chart (bottom) demonstrating mass balance of vinylic pinacolboronate **1** during the reaction progress of $Cu(OAc)_2$/NPI-catalyzed C-O cross-coupling of **1**+**2** with **DCP** oxidant.

With DCP oxidant, approximately 80% of the mass balance of vinylic pinacolboronate **1** was accounted by the formation of vinylic ether **3** and the various alkene-containing side products **5** – **7** (**Figure S11**). As DCP-derived cumyl vinylic ether **6** was generated, this indicates that a cumyl alkoxide Cu-adduct is formed during the catalytic cycle, allowing for reaction with vinylic pinacolboronate **1**. This also indicates that an alcohol ligand exchange step occurs between alcohol **2** and a reactive Cu-cumyl alkoxide species, prior to reductive elimination. However, we do not know if this alcohol ligand exchange occurs with a Cu(II) or Cu(III) reactive intermediate.

2. Competition study with *p*-tolyl pinacolboronate 31 with optimized conditions:

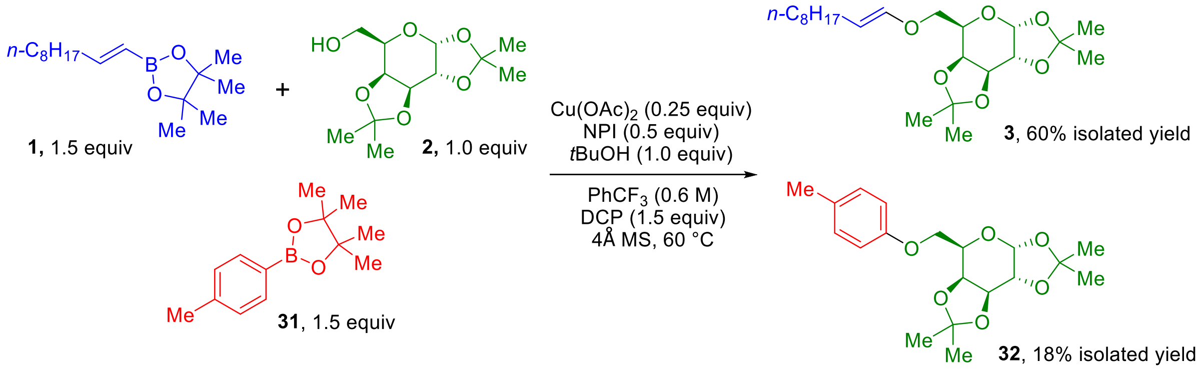

The reaction was conducted at 0.31 mmol scale according to the optimized protocol described in section B1, with the addition of *p*-tolyl pinacolboronate **31** (101 mg, 0.47 mmol, 1.5 equiv). The reaction mixture was stirred for 30 minutes before it was cooled to room temperature. The mixture was diluted with DCM, transferred into a 20 mL vial, and concentrated by rotary evaporation. ^{1}H NMR spectrum of the crude reaction mixture was obtained in C_6D_6, and estimated NMR yields were analyzed according to section B4.

The ^{1}H NMR spectrum of the crude mixture showed ~92% consumption of vinylic pinacolboronate **1**, and only ~18% consumption of *p*-tolyl pinacolboronate **31**. The spectrum also showed 62% NMR yield of vinylic ether **3**, 7% NMR yield of vinylic acetate **5**, ~6% NMR yield of acetate ester **4**, 7% NMR yield of cumyl vinylic ether **6**, 6% NMR yield of alkene **S1** from protodeboronation, and traces of diene **S2**. A new peak arose at the galactose-derived materials' anomeric protons (5.54 ppm), which was confirmed after purification to correspond to *p*-tolyl ether **32**.

The crude mixture was subjected to flash column chromatography in silica gel pre-treated with 2% Et_3N in hexanes and eluting with a gradient of 98:2-96:4-93:7-90:10 hexanes/EtOAc. The obtained fractions were analyzed by TLC in 90:10 hexanes/EtOAc and stained with PMA. ^{1}H NMR spectra were measured in C_6D_6.Vinylic ether **3** was obtained in a mixture of products including *p*-tolyl pinacolboronate **31** and trace vinylic pinacol boronate **1** (85 mg total). The isolated yield of **3** was estimated at 60% yield (74 mg). *p*-Tolyl ether **32** was obtained in a mixture with *p*-tolyl pinacolboronate **31** (31 mg in total). The isolated yield of compound **32** was estimated at 18% yield (20 mg). About 80% *p*-tolyl pinacolboronate **31** was unconsumed.

Characterization of *p*-tolyl ether **32** (from a mixture of 0.35 : 1.0 *p*-tolyl pinacolboronate **31** : *p*-tolyl ether **32**, containing traces of hexanes from column chromatography):

^{1}H NMR (400 MHz, C_6D_6) δ 6.90 – 6.82 (m, 4H), 5.54 (d, *J* = 5.0 Hz, 1H), 4.51 (dd, *J* = 7.9, 2.4 Hz, 1H), 4.39 (td, *J* = 6.2, 1.9 Hz, 1H), 4.32 (dd, *J* = 9.4, 6.4 Hz, 1H), 4.25 (dd, *J* = 9.4, 6.1 Hz, 1H), 4.20 (dd, *J* = 7.9, 1.9 Hz, 1H), 4.18 (dd, *J* = 5.2, 2.4 Hz, 1H), 2.06 (s, 4H, overlapping with tolyl Bpin **31**), 1.45 (s, 3H), 1.40 (s, 3H), 1.13 (s, 3H), 1.04 (s, 3H).

^{13}C{^{1}H} NMR (101 MHz, C_6D_6) δ 157.4, 130.2, 115.1, 109.3, 108.5, 96.9, 71.5, 71.3, 71.2, 67.3, 66.8, 26.3, 26.2, 25.0, 24.9, 24.4, 20.5 (overlapping with *p*-tolyl pinacolboronate **31**). (confirmed by HSQC and HMBC)

HRMS (ESI) *m/z*: $[M+Na]^+$ Calcd for $C_{19}H_{26}O_6Na$ 373.1622; Found 373.1621.

HRMS (ESI) *m/z*: $[M+H]^+$ Calcd for $C_{19}H_{27}O_6$ 351.18022; Found 351.18020.

Identifiable ^{1}H NMR signals of tolyl Bpin 31 in C_6D_6: 8.13 (dt, *J* = 7.9, 1.4 Hz, 2H), 7.05 (dt, *J* = 7.4, 0.9 Hz, 2H), 2.05 (s, 3H), 1.12 (s, 12H).

Identifiable ^{13}C NMR signals of tolyl Bpin 31 in C_6D_6 (based on HSQC and HMBC): 141.5, 135.6, 130.0, 83.6, 25.0, 20.5 (overlapping with tolyl ether **32**), *^{13}C bonded to B is not visible.

3. Control experiment with DTBP oxidant and *t*-BuOH, without 1° or 2° alcohol reactant:

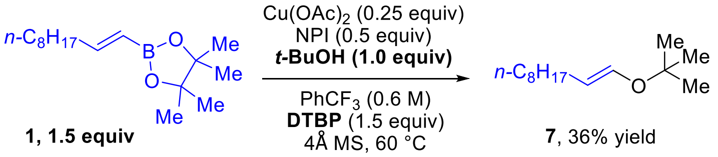

Experimental protocol: *N*-isopropylimidazole (NPI, 18 µL, 0.16 mmol, 0.50 equiv), *t*-butanol (30 µL, 0.31 mmol, 1.0 equiv), DTBP (68 mg, 0.47 mmol, 1.5 equiv), $Cu(OAc)_2$ (14 mg, 0.08 mmol, 0.25 equiv), and vinylic pinacolboronate **1** (124 mg, 0.47 mmol, 1.5 equiv) were added sequentially to a flame-dried 4 mL reaction vial (charged with a stir bar and 110-115 mg of 4Å MS before flame-drying) under air. The vial was sealed with a septum cap and sparged with argon. Trifluorotoluene ($PhCF_3$, 0.5 mL, 0.6 M based on mmol of *t*-BuOH) was added to the reaction vial and the mixture was further purged with argon for 3-5 minutes. The septum cap was replaced with a solid cap and the vial was sealed with Teflon and electrical tape. The vial was stirred at 60 °C on a pre-heated heating block with an internal temperature probe. The reaction mixture changed from a deep blue mixture to a dark green mixture after 10-15 minutes of stirring. The reaction mixture was cooled down to room temperature after stirring for 18 hours, diluted with DCM, transferred into 20 mL vial, and concentrated by rotary evaporation. ^1H NMR spectrum of the crude product mixture was obtained in C_6D_6, showing complete conversion of vinylic pinacolboronate **1**, *tert*-butyl vinylic ether **7** as major product, and substantial amounts of vinylic acetate **5**, alkene **S1**, and diene **S2**. The crude product mixture was purified by flash column chromatography in silica gel pre-treated with 2% Et_3N in pentane and eluting with 100% pentane. *Tert*-butyl vinylic ether **7** was obtained as clear pale-yellow oil (24 mg, 36% isolated yield). Characterization data of compound **7** was reported in section B5.

4. Control experiment without vinylic pinacolboronate 1 (unoptimized conditions):

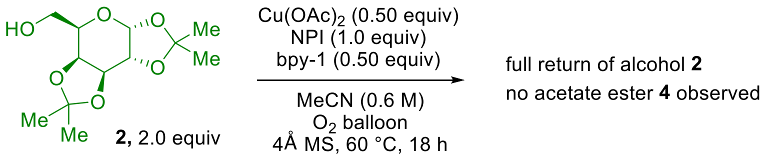

Experimental protocol: Alcohol **2** (80 mg, 0.31 mmol, 2.0 equiv), $Cu(OAc)_2$ (15 mg, 0.08 mmol, 0.50 equiv), NPI (17 µL, 0.16 mmol, 1.0 equiv), and bpy-1 (12 mg, 0.08 mmol, 0.50 equiv) were added to a flame-dried 4 mL vial (charged with a stir bar and 90 mg of 4Å MS before flame-drying). Anhydrous MeCN (0.3 mL) was added to the reaction vial under argon atmosphere. The mixture was placed under O_2 atmosphere (in a balloon). The reaction vial was taped with Teflon and electrical tapes and stirred in a pre-heated heating block at 60 °C over 18 hours. Afterwards, the reaction was cooled to room temperature, diluted with DCM, transferred to a 20 mL vial, and concentrated by rotary evaporation. The crude reaction mixture was a dark blue oil and analyzed with ^1H NMR in C_6D_6. The NMR spectrum of the crude reaction mixture showed no conversion of alcohol **2**.

G. Mechanistic hypothesis for optimized procedure

Based on the optimization study, preliminary mechanistic study, and substrate scope study, we proposed the following reaction mechanism (modified based on King et al. 2012).[13]

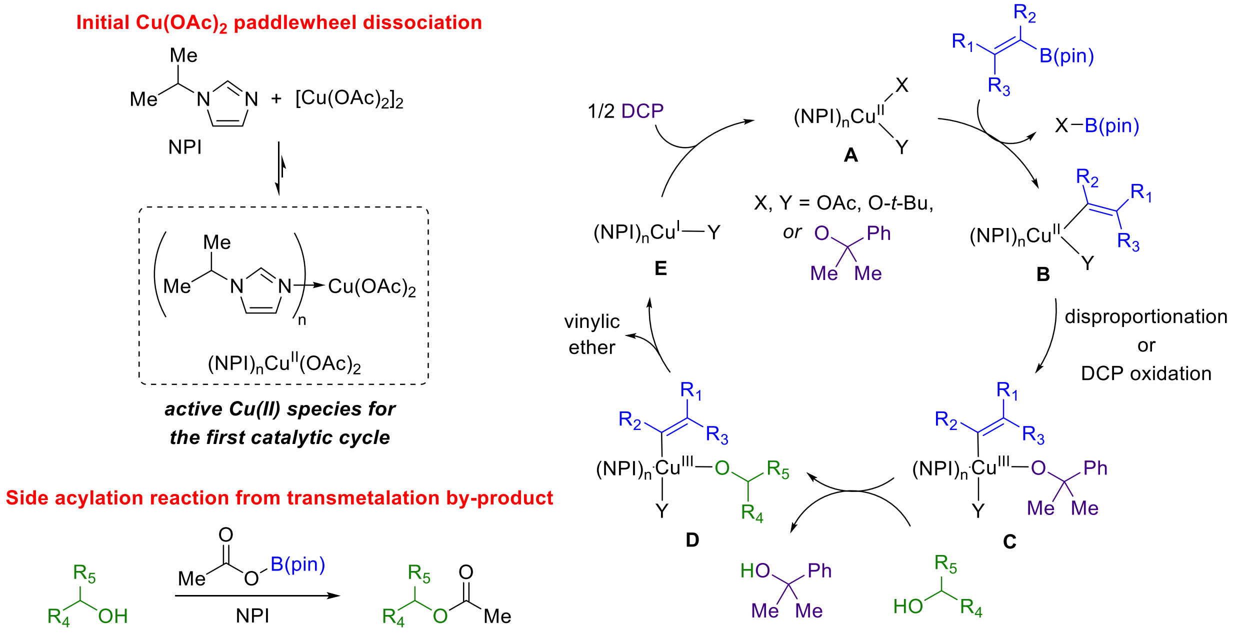

Figure S12. Proposed mechanism for Cu(II)-catalyzed vinylic C-O cross-coupling reaction.

Following well-established precedents, we propose that the NPI ligand favors dissociation of dimeric $[Cu(OAc)_2]_2$, to generate $(NPI)_nCu^{II}(OAc)_2$.[18] Having observed an induction period, we hypothesize that the reaction proceeds by two different catalytically active Cu species. In the first catalytic cycle, we propose that X and Y are acetate (OAc) ligands in **A**. Transmetalation assisted by acetate ligand gives the vinylic-Cu(II) intermediate **B**, also generating AcO-B(pin) byproduct, which may be the source of alcohol *O*-acetylation.[21] Although redox disproportionation has been canonically accepted as the mechanism for generating Cu(III) intermediate **C** from Cu(II) **B**, we hypothesize that dicumyl peroxide (DCP) may also convert Cu(II) **B** to Cu(III) **C**. The increased Lewis acidity of the Cu(III) intermediate **C** may promote alcohol reactant coordination. The basic 3° alkoxide ligand may favor proton transfer/ligand exchange with the 1° or 2° alcohol reactant, to give Cu(III) alkoxide intermediate **D**. This alcohol exchange step is sensitive to steric hindrance, as evident with lower yields from (-)-menthol (**9c**) and substantial amounts of cumyl vinylic ether side product **6**. Reductive elimination to release the vinylic ether product generates Cu(I) intermediate **E**, which undergoes one-electron oxidation with DCP to regenerate Cu(II) catalyst **A**, now with X or Y = $OCMe_2Ph$. DCP with weaker O-O bond strength than DTBP correlates with increased catalytic activity, hence a much shorter induction period. Thus, for subsequent catalytic cycles, the resulting Cu(II)-alkoxide pre-transmetalation complex is more reactive than the initial $(NPI)_nCu^{II}(OAc)_2$, increasing the rate of transmetalation.[16] In the previously studied aryl ether mechanism, transmetalation was the rate-limiting step.[13] However, with the much faster reaction rate of vinylic pinacolboronate **1** and the alcohol reactant as the limiting reagent, we hypothesize that the rate-limiting step in our reaction may involve the alcohol reactant reacting with Cu(III)-alkoxide **C**.

H. Incompatible substrates

1. Unreactive substrates (resulting in catalyst inhibition and no conversion of starting materials)

Vinylic borons:

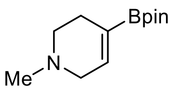

27

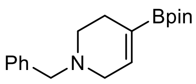

28

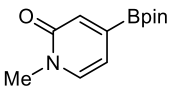

S7

Alcohols:

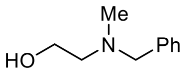

29

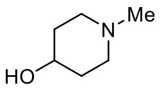

30

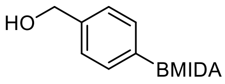

S8

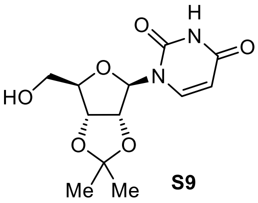

S9

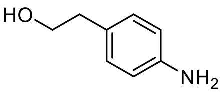

S10

2. Substrates with low reactivity or with other side reactions

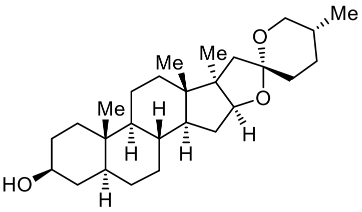

tigogenin (S11)

trace of desired vinylic ether found, protodeboronation predominated

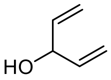

S12

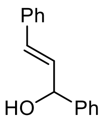

S13

only trace of desired vinylic ether found, diene formation and protodeboronation predominated

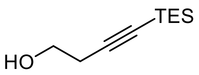

S14

complex mixtures of vinylic ether products

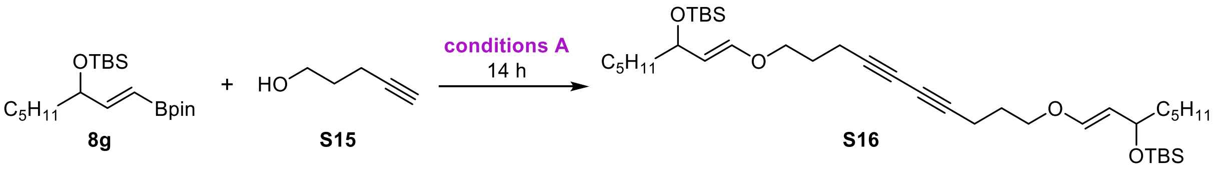

8g **S15** **S16**

observed as major product with precedented Glaser-type homocoupling reactivity at the terminal alkyne position[34]

I. References Cited

(1) Wang, Y. D.; Kimball, G.; Prashad, A. S.; Wang, Y. Zr-Mediated Hydroboration: Stereoselective Synthesis of Vinyl Boronic Esters. *Tetrahedron Lett.* **2005**, *46* (50), 8777–8780. https://doi.org/10.1016/j.tetlet.2005.10.031.

(2) Baumann, J. E.; Chung, C. P.; Lalic, G. Stereoselective Copper-Catalyzed Olefination of Imines. *Angew. Chem. Int. Ed.* **2024**, *63* (6), e202316521. https://doi.org/10.1002/anie.202316521.

(3) Macé, A.; Touchet, S.; Andres, P.; Cossío, F.; Dorcet, V.; Carreaux, F.; Carboni, B. [3,3]-Sigmatropic Rearrangement/Allylboration/Cyclization Sequence: Enantioenriched Seven-Membered-Ring Carbamates and Ring Contraction to Pyrrolidines. *Angew. Chem. Int. Ed.* **2016**, *55* (3), 1025–1029. https://doi.org/10.1002/anie.201509824.

(4) Arredondo, V. M.; Tian, S.; McDonald, F. E.; Marks, T. J. Organolanthanide-Catalyzed Hydroamination/Cyclization. Efficient Allene-Based Transformations for the Syntheses of Naturally Occurring Alkaloids. *J. Am. Chem. Soc.* **1999**, *121* (15), 3633–3639. https://doi.org/10.1021/ja984305d.

(5) Luo, L.; Song, Q.; Li, Y.; Cao, Z.; Qiang, X.; Tan, Z.; Deng, Y. Design, Synthesis and Evaluation of Phthalide Alkyl Tertiary Amine Derivatives as Promising Acetylcholinesterase Inhibitors with High Potency and Selectivity against Alzheimer's Disease. *Bioorg. Med. Chem.* **2020**, *28* (8), 115400. https://doi.org/10.1016/j.bmc.2020.115400.

(6) Sader, J. K.; Molder, B. A.; Wulff, J. E. A Chan–Evans–Lam Approach to Trisubstituted Vinyl Ethers. *Org. Biomol. Chem.* **2021**, *19* (44), 9649–9653. https://doi.org/10.1039/D1OB01827B.

(7) Bastick, K. A. C.; Watson, A. J. B. Pd-Catalyzed Organometallic-Free Homologation of Arylboronic Acids Enabled by Chemoselective Transmetalation. *ACS Catal.* **2023**, *13* (10), 7013–7018. https://doi.org/10.1021/acscatal.3c00921.

(8) Pham, S. L.; Kim, T.; McDonald, F. E. Stereospecific Cu(I)-Catalyzed C–O Cross-Coupling Synthesis of Acyclic 1,2-Di- and Trisubstituted Vinylic Ethers from Alcohols and Vinylic Halides. *Org. Lett.* **2023**, *25* (28), 5297–5301. https://doi.org/10.1021/acs.orglett.3c01849.

(9) Seepersaud, M.; Seecharan,S.; Lalgee,L. J.; Jalsa, N. K. Ceric Ammonium Nitrate/Acetic Anhydride: A Tunable System for the O-Acetylation and Mononitration of Diversely Protected Carbohydrates. *Synth. Commun.* **2017**, *47* (9), 853–871. https://doi.org/10.1080/00397911.2016.1230219.

(10) Arceo, E.; Marsden, P.; Bergman, R. G.; Ellman, J. A. An Efficient Didehydroxylation Method for the Biomass-Derived Polyols Glycerol and Erythritol. Mechanistic Studies of a Formic Acid-Mediated Deoxygenation. *Chem. Commun.* **2009**, No. 23, 3357–3359. https://doi.org/10.1039/B907746D.

(11) Maji, M. S.; Pfeifer, T.; Studer, A. Oxidative Homocoupling of Aryl, Alkenyl, and Alkynyl Grignard Reagents with TEMPO and Dioxygen. *Angew. Chem. Int. Ed.* **2008**, *47* (49), 9547–9550. https://doi.org/10.1002/anie.200804197.

(12) Shade, R. E.; Hyde, A. M.; Olsen, J.-C.; Merlic, C. A. Copper-Promoted Coupling of Vinyl Boronates and Alcohols: A Mild Synthesis of Allyl Vinyl Ethers. *J. Am. Chem. Soc.* **2010**, *132* (4), 1202–1203. https://doi.org/10.1021/ja907982w.

(13) King, A. E.; Ryland, B. L.; Brunold, T. C.; Stahl, S. S. Kinetic and Spectroscopic Studies of Aerobic Copper(II)-Catalyzed Methoxylation of Arylboronic Esters and Insights into Aryl Transmetalation to Copper(II). *Organometallics* **2012**, *31* (22), 7948–7957. https://doi.org/10.1021/om300586p.

(14) Roy, T.; Mondal, K.; Sengupta, A.; Das, P. CuF_2/MeOH-Catalyzed N3-Selective Chan-Lam Coupling of Hydantoins: Method and Mechanistic Insight. *J. Org. Chem.* **2023**, *88* (9), 6058–6070. https://doi.org/10.1021/acs.joc.3c00408.

(15) Vantourout, J. C.; Miras, H. N.; Isidro-Llobet, A.; Sproules, S.; Watson, A. J. B. Spectroscopic Studies of the Chan–Lam Amination: A Mechanism-Inspired Solution to Boronic Ester Reactivity. *J. Am. Chem. Soc.* **2017**, *139* (13), 4769–4779. https://doi.org/10.1021/jacs.6b12800.

(16) Kundu, S.; Greene, C.; Williams, K. D.; Salvador, T. K.; Bertke, J. A.; Cundari, T. R.; Warren, T. H. Three-Coordinate Copper(II) Aryls: Key Intermediates in C–O Bond Formation. *J. Am. Chem. Soc.* **2017**, *139* (27), 9112–9115. https://doi.org/10.1021/jacs.7b04046.

(17) Ribas, X.; Güell, I. Cu(I)/Cu(III) Catalytic Cycle Involved in Ullmann-Type Cross-Coupling Reactions. *Pure Appl. Chem.* **2014**, *86* (3), 345–360. https://doi.org/10.1515/pac-2013-1104.

(18) Kühl, O.; Millinghaus, S.; Palm, G. Green, Purple, Blue — [Cu(N-Methylimidazole)$_n$ $(OOCCH_3)_2]_m$, What Is in a Colour. *Cent. Eur. J. Chem.* **2011**, *9* (4), 706–711. https://link.springer.com/article/10.2478/s11532-011-0049-z.

(19) Salvador, T. K.; Arnett, C. H.; Kundu, S.; Sapiezynski, N. G.; Bertke, J. A.; Raghibi Boroujeni, M.; Warren, T. H. Copper Catalyzed sp^3 C–H Etherification with Acyl Protected Phenols. *J. Am. Chem. Soc.* **2016**, *138* (51), 16580–16583. https://doi.org/10.1021/jacs.6b09057.

(20) Gephart, R. T. I.; McMullin, C. L.; Sapiezynski, N. G.; Jang, E. S.; Aguila, M. J. B.; Cundari, T. R.; Warren, T. H. Reaction of CuI with Dialkyl Peroxides: CuII-Alkoxides, Alkoxy Radicals, and Catalytic C–H Etherification. *J. Am. Chem. Soc.* **2012**, *134* (42), 17350–17353. https://doi.org/10.1021/ja3053688.

(21) Gudun, K. A.; Zakarina, R.; Segizbayev, M.; Hayrapetyan, D.; Slamova, A.; Khalimon, A. Y. Cobalt-Catalyzed Deoxygenative Hydroboration of Nitro Compounds and Applications to One-Pot Synthesis of Aldimines and Amides. *Adv. Synth. Catal.* **2022**, *364* (3), 601–611. https://doi.org/10.1002/adsc.202101043.

(22) Sueki, S.; Kuninobu, Y. Copper-Catalyzed *N*- and *O*-Alkylation of Amines and Phenols using Alkylborane Reagents. *Org. Lett.* **2013**, *15*, 1544-1547. https://doi.org/10.1021/ol400323z

(23) Hoover, J. M.; Stahl, S. S. Highly Practical Copper(I)/TEMPO Catalyst System for Chemoselective Aerobic Oxidation of Primary Alcohols. *J. Am. Chem. Soc.* **2011**, *133* (42), 16901–16910. https://doi.org/10.1021/ja206230h.

(24) Hoover, J. M.; Ryland, B. L.; Stahl, S. S. Mechanism of Copper(I)/TEMPO-Catalyzed Aerobic Alcohol Oxidation. *J. Am. Chem. Soc.* **2013**, *135* (6), 2357–2367. https://doi.org/10.1021/ja3117203.

(25) Halford-McGuff, J. M.; Israel, E. M.; West, M. J.; Vantourout, J. C.; Watson, A. J. B. Direct Chan–Lam Amination and Etherification of Aryl BMIDA Reagents. *Eur. J. Org. Chem.* **2022**, *2022* (45), e202200993. https://doi.org/10.1002/ejoc.202200993.

(26) Delaney, C. P.; Zahrt, A. F.; Kassel, V. M.; Denmark, S. E. Effects of Ring Size and Steric Encumbrance on Boron-to-Palladium Transmetalation from Arylboronic Esters. *J. Org. Chem.* **2024**. https://doi.org/10.1021/acs.joc.3c02629.

(27) Oka, N.; Yamada, T.; Sajiki, H.; Akai, S.; Ikawa, T. Aryl Boronic Esters Are Stable on Silica Gel and Reactive under Suzuki–Miyaura Coupling Conditions. *Org. Lett.* **2022**, *24* (19), 3510–3514. https://doi.org/10.1021/acs.orglett.2c01174.

(28) Mondal, K.; Patra, S.; Halder, P.; Mukhopadhyay, N.; Das, P. CuF_2/DMAP-Catalyzed N-Vinylation: Scope and Mechanistic Study. *Org. Lett.* **2022**. https://doi.org/10.1021/acs.orglett.2c03856.

(29) Chen, J.-Q.; Li, J.-H.; Dong, Z.-B. A Review on the Latest Progress of Chan-Lam Coupling Reaction. *Adv. Synth. Catal.* **2020**, *362* (16), 3311–3331. https://doi.org/10.1002/adsc.202000495.

(30) Vantourout, J. C.; Li, L.; Bendito-Moll, E.; Chabbra, S.; Arrington, K.; Bode, B. E.; Isidro-Llobet, A.; Kowalski, J. A.; Nilson, M. G.; Wheelhouse, K. M. P.; Woodard, J. L.; Xie, S.; Leitch, D. C.; Watson, A. J. B. Mechanistic Insight Enables Practical, Scalable, Room Temperature Chan–Lam *N*-Arylation of *N*-Aryl Sulfonamides. *ACS Catal.* **2018**, *8* (10), 9560–9566. https://doi.org/10.1021/acscatal.8b03238.

(31) Duh, Y.-S.; Yo, J.-M.; Lee, W.-L.; Kao, C.-S.; Hsu, J.-M. Thermal Decompositions of Dialkyl Peroxides Studied by DSC. *J. Therm. Anal. Calorim.* **2014**, *118* (1), 339–347. https://doi.org/10.1007/s10973-014-3998-6.

(32) Wang, Q.; Biosca, M.; Himo, F.; Szabó, K. J. Electrophilic Fluorination of Alkenes via Bora-Wagner–Meerwein Rearrangement. Access to β-Difluoroalkyl Boronates. *Angew. Chem. Int. Ed.* **2021**, *60* (50), 26327–26331. https://doi.org/10.1002/anie.202109461.

(33) Agata, R.; Lu, S.; Matsuda, H.; Isozaki, K.; Nakamura, M. Regio- and Stereoselective Synthesis of 1,4-Enynes by Iron-Catalysed Suzuki–Miyaura Coupling of Propargyl Electrophiles under Ligand-Free Conditions. *Org. Biomol. Chem.* **2020**, *18* (16), 3022–3026. https://doi.org/10.1039/D0OB00357C.

(34) Quach, T. D.; Batey, R. A. Copper(II)-Catalyzed Ether Synthesis from Aliphatic Alcohols and Potassium Organotrifluoroborate Salts. *Org. Lett.* **2003**, *5*, 1381-1384. https://doi.org/10.1021/ol034454n.

J. Copies of NMR spectra for new compounds

^{1}H NMR spectrum of compound **3** (400 MHz, $CDCl_3$)

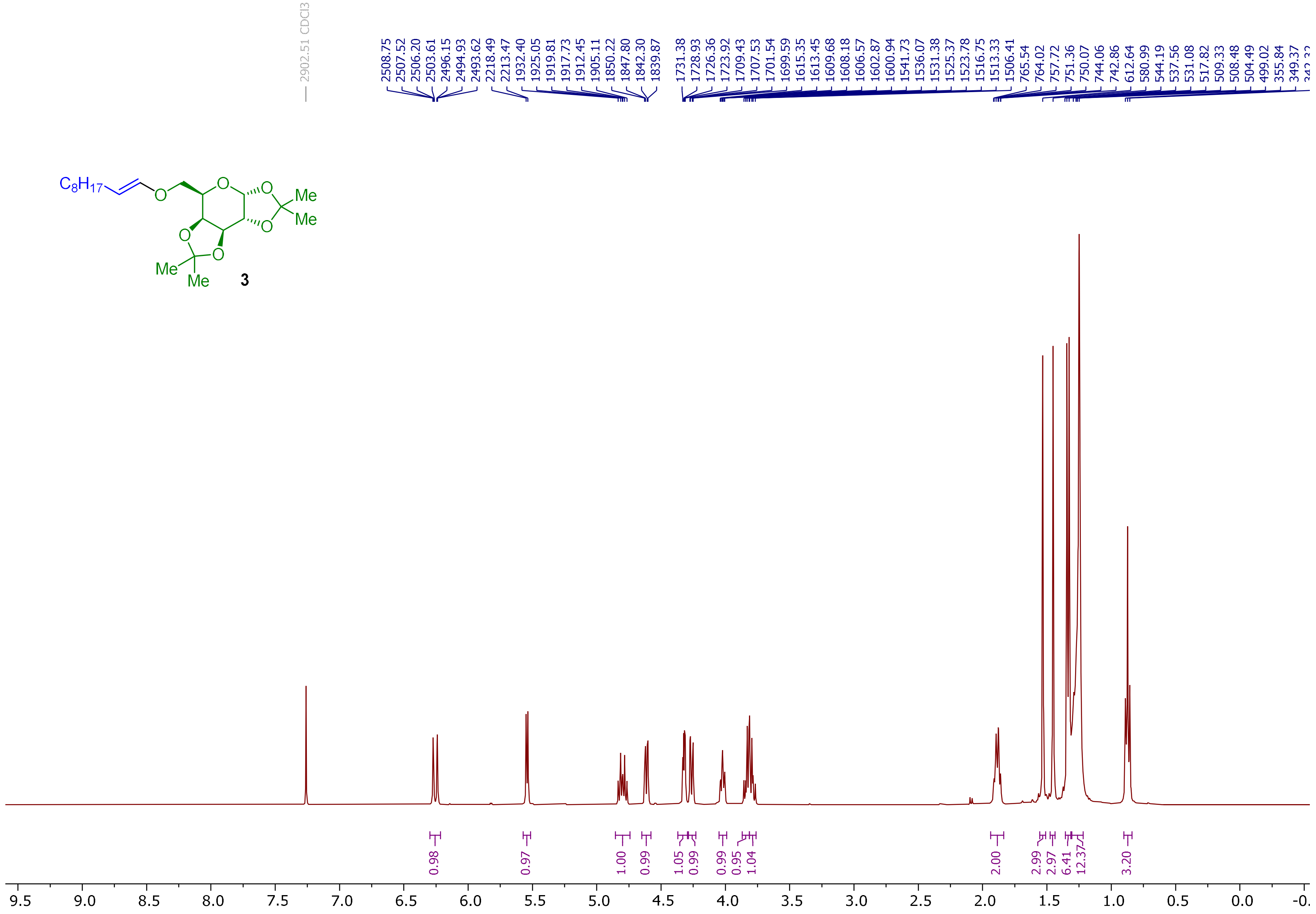

^{1}H NMR spectrum of compound **3** (400 MHz, C_6D_6)

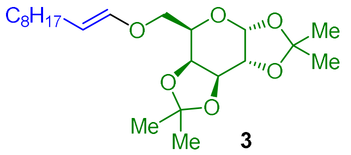

3

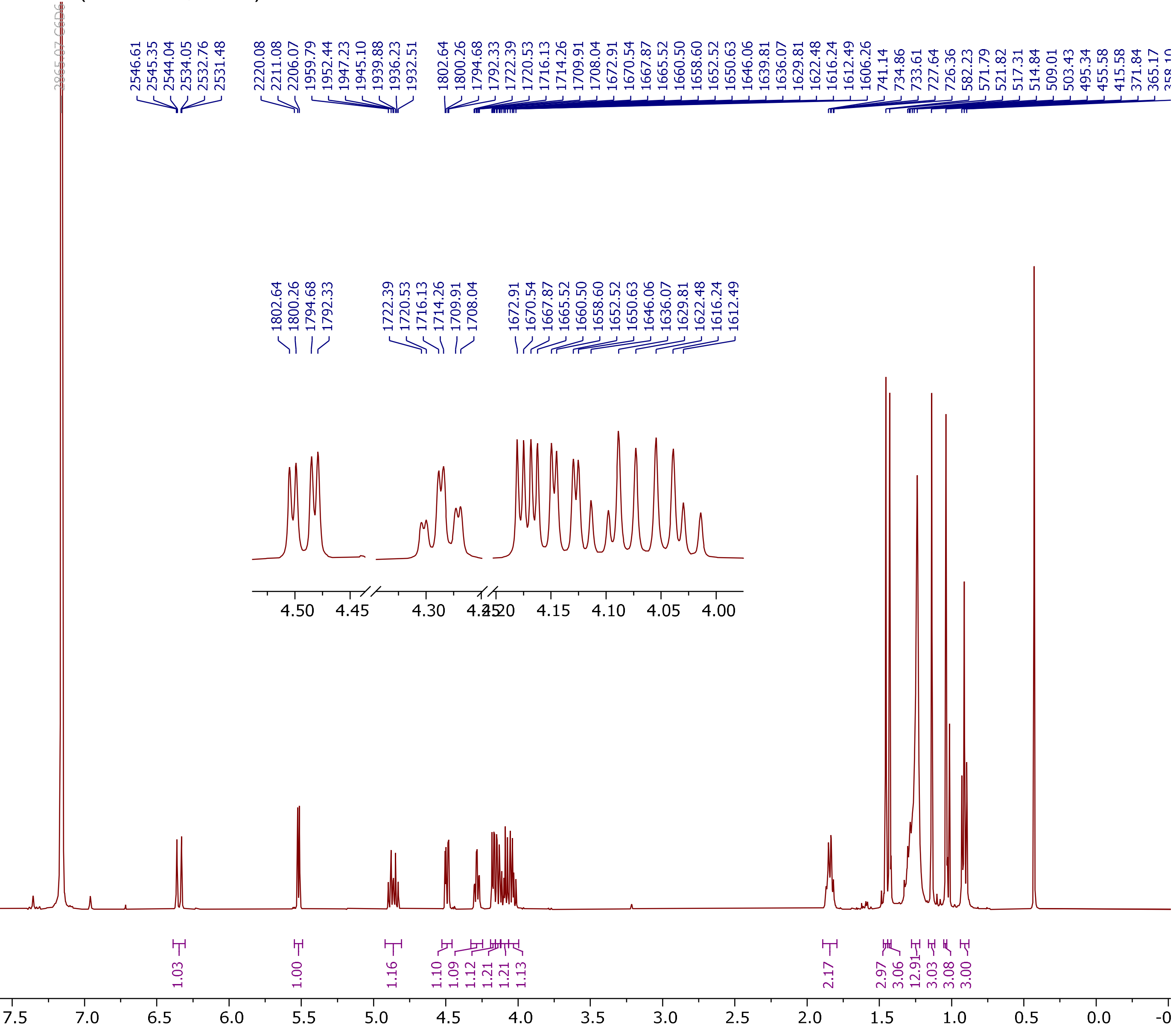

^{1}H NMR spectrum of compound **4** (containing hexanes) (400 MHz, $CDCl_3$)

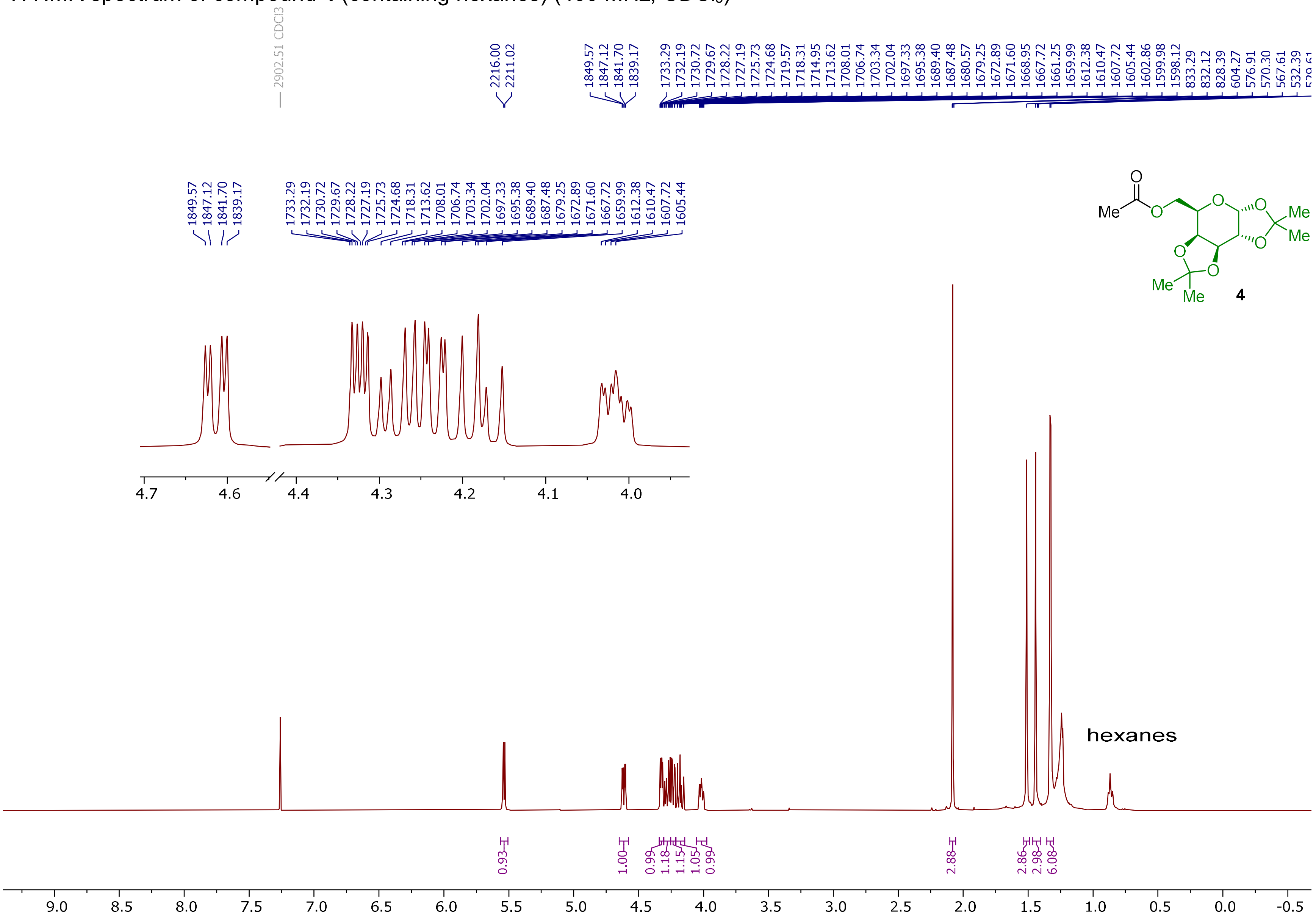

^1H NMR spectrum of compound **4** (containing hexanes from column chromatography) (400 MHz, C_6D_6)

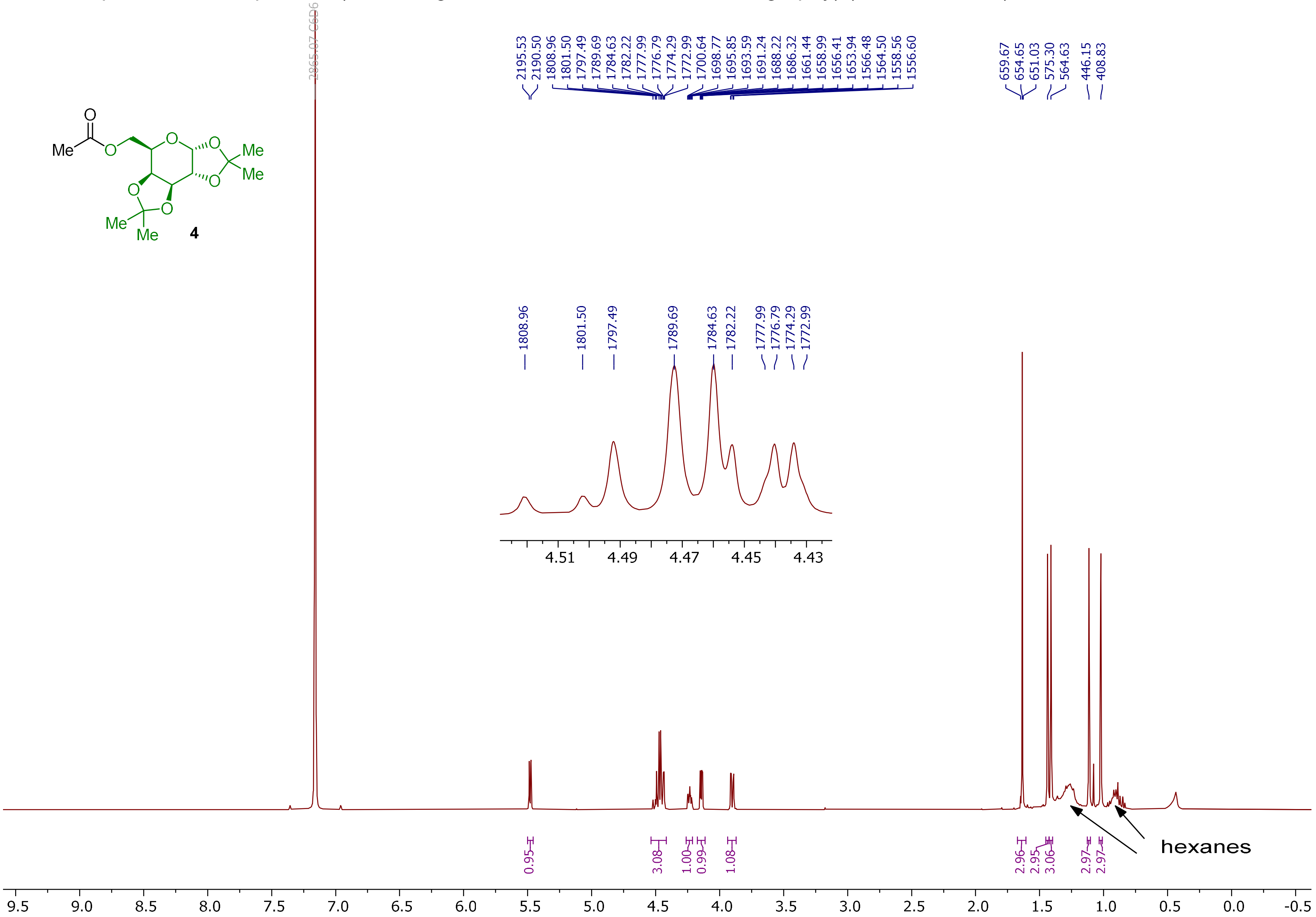

^{1}H NMR spectrum of compound **5** (containing ~10% 1-decanal) (400 MHz, C_6D_6)

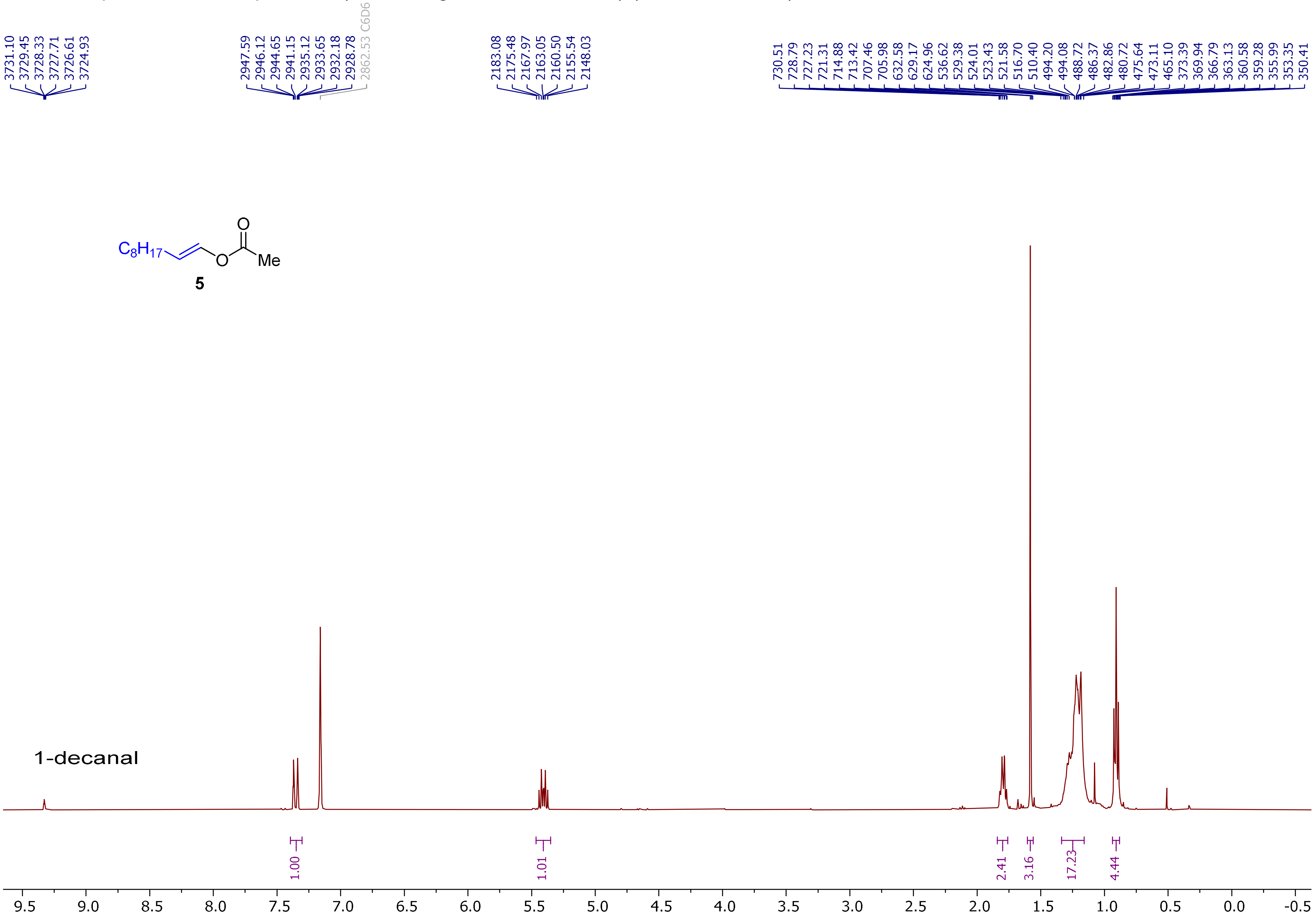

$^{13}C\{^{1}H\}$ NMR spectrum of compound **5** in C_6D_6 (containing ~10% 1-decanal) (101 MHz, C_6D_6)

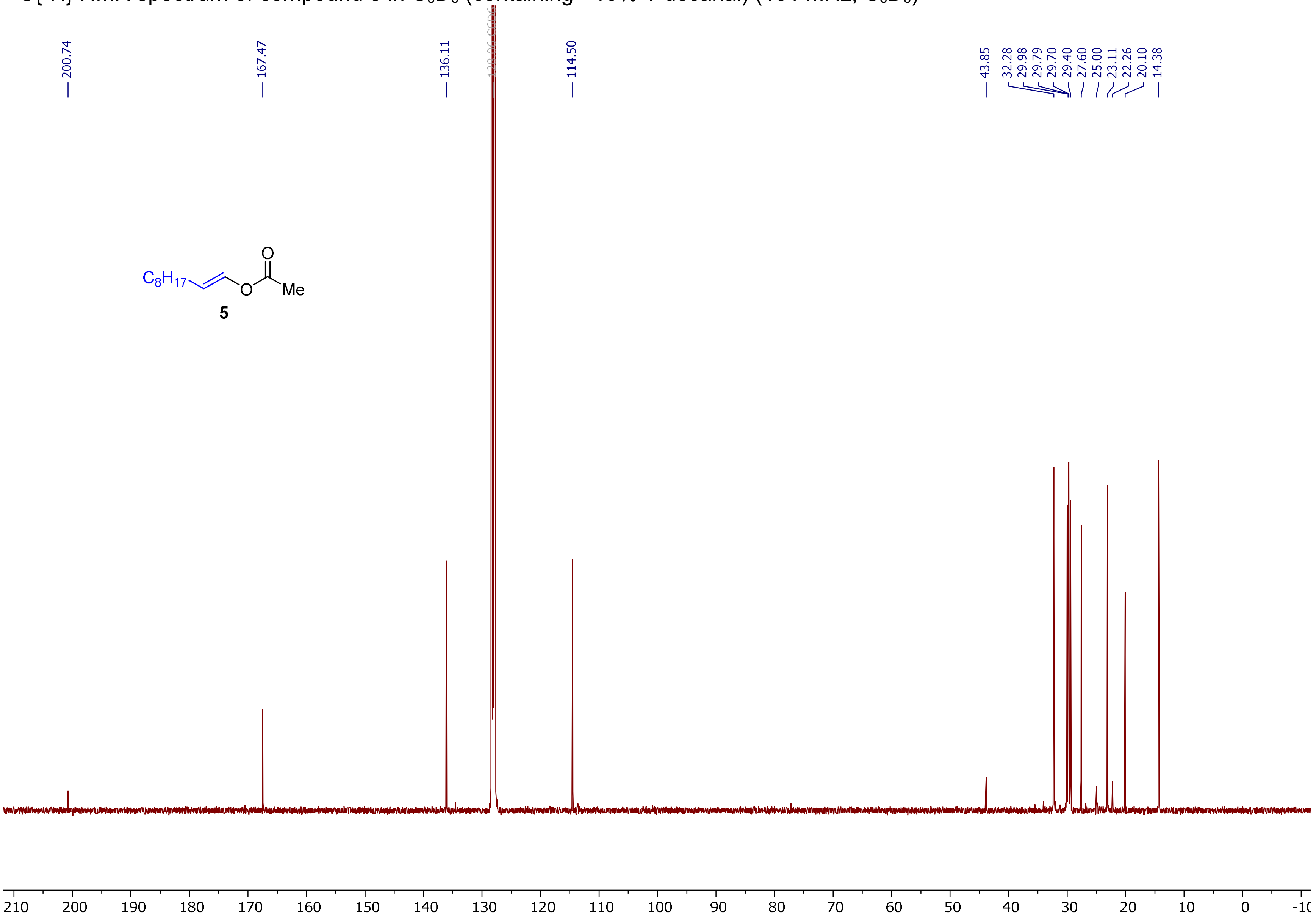

^{1}H NMR spectrum of compound **S2** (400 MHz, C_6D_6)

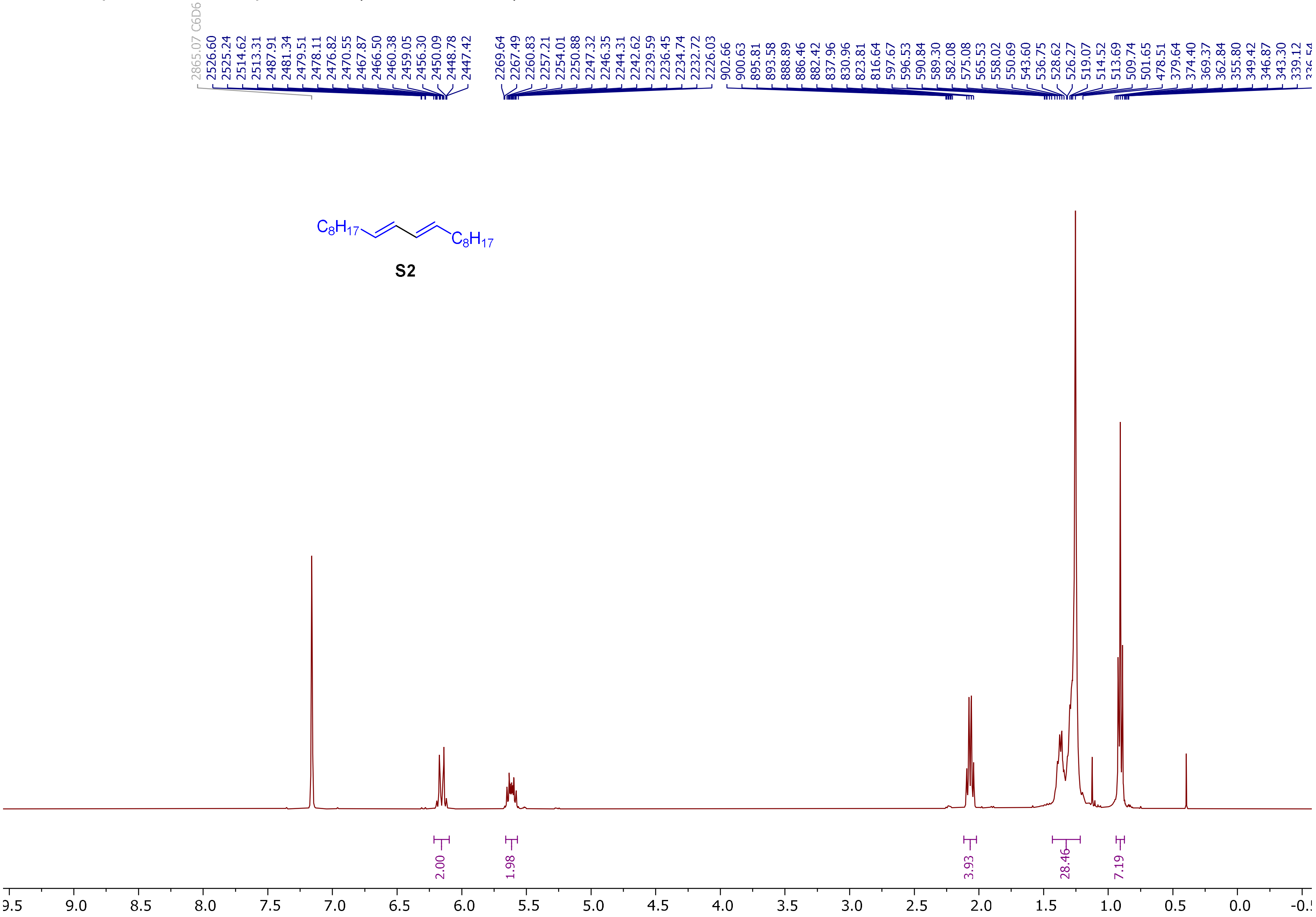

^{1}H NMR spectrum of compound **6** (400 MHz, C_6D_6)

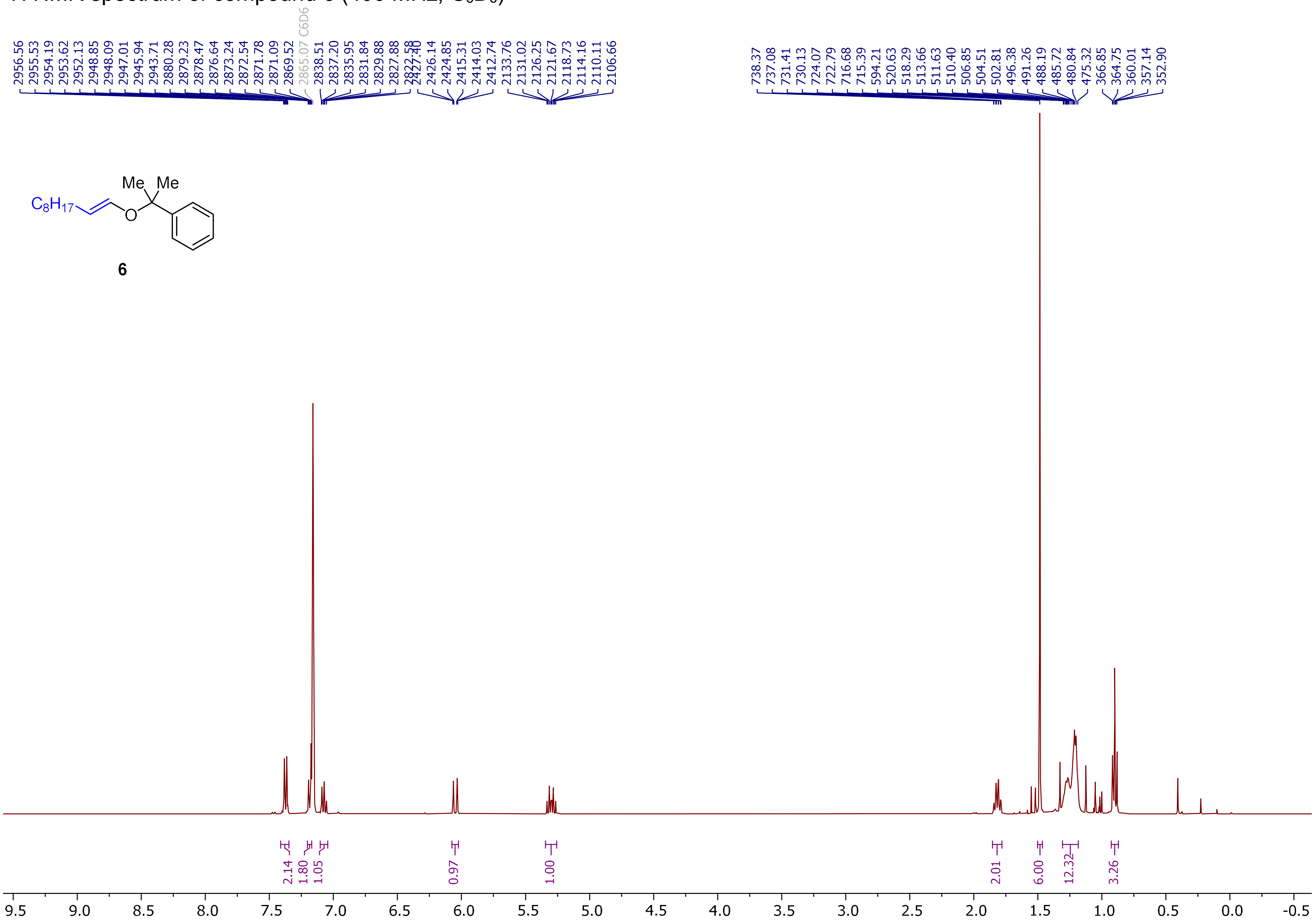

$^{13}C\{^1H\}$ NMR spectrum of compound **6** (101 MHz, C_6D_6)

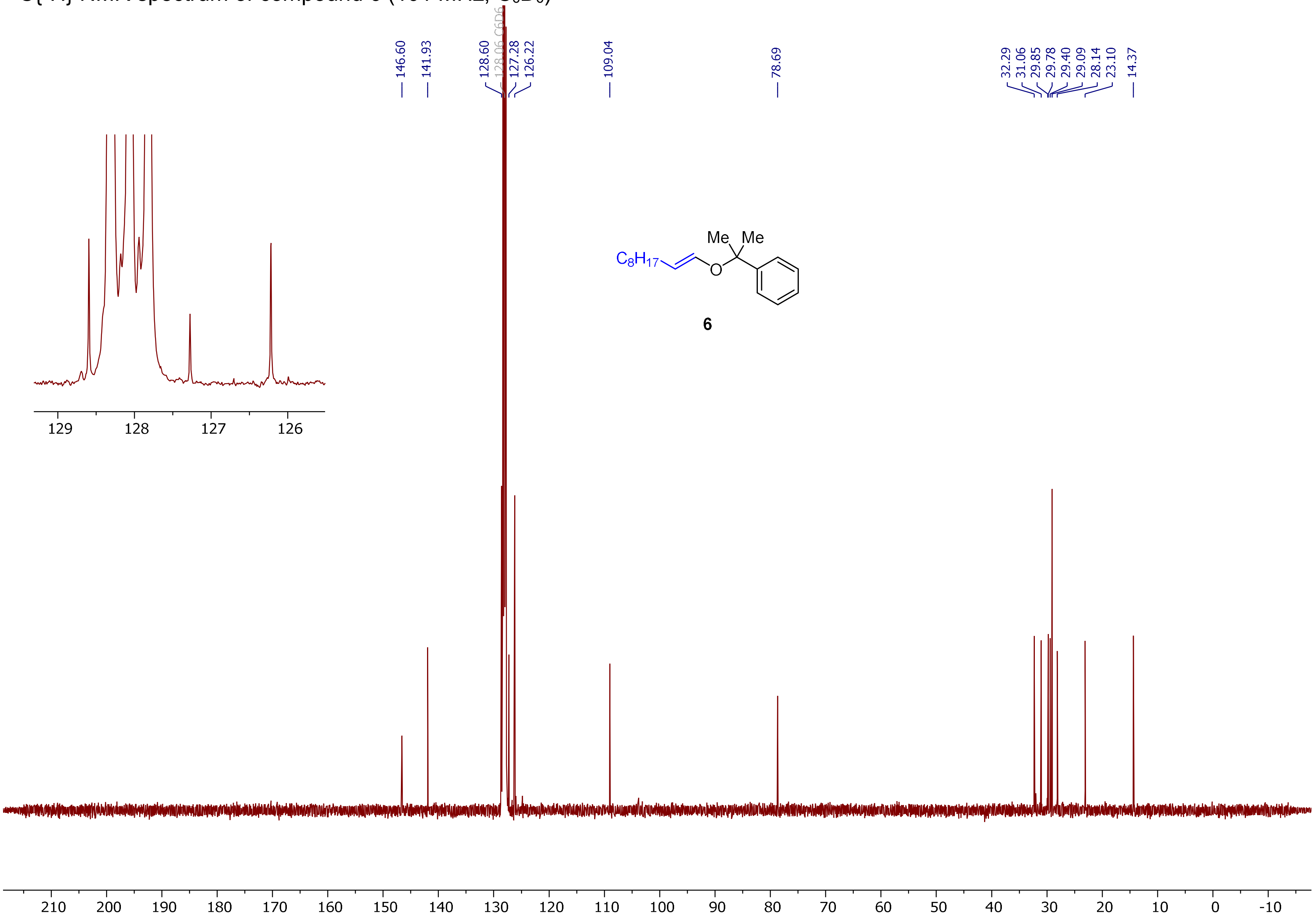

^{1}H NMR spectrum of compound **7** (400 MHz, C_6D_6)

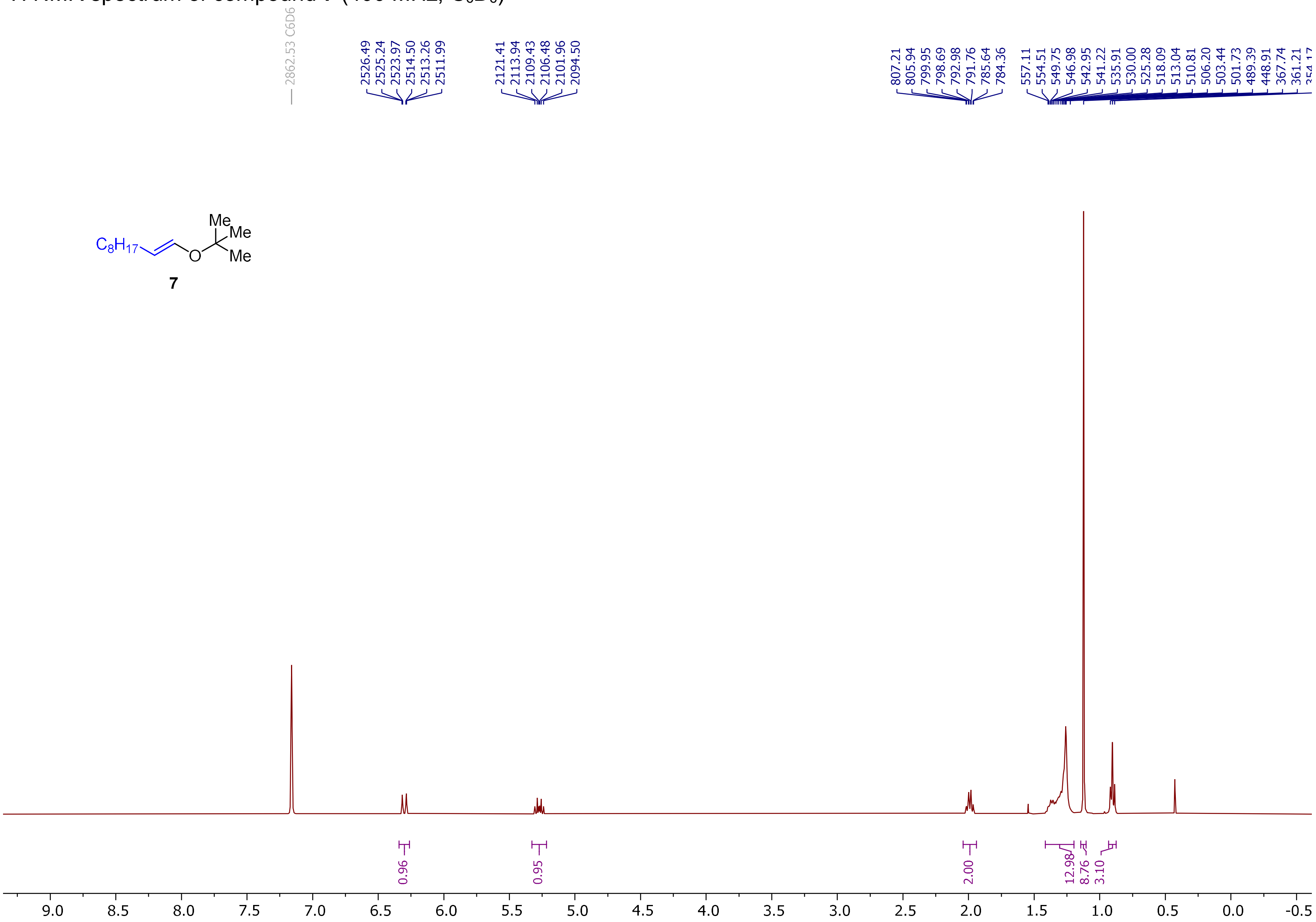

$^{13}C\{^1H\}$ NMR spectrum of compound **7** (101 MHz, C_6D_6)

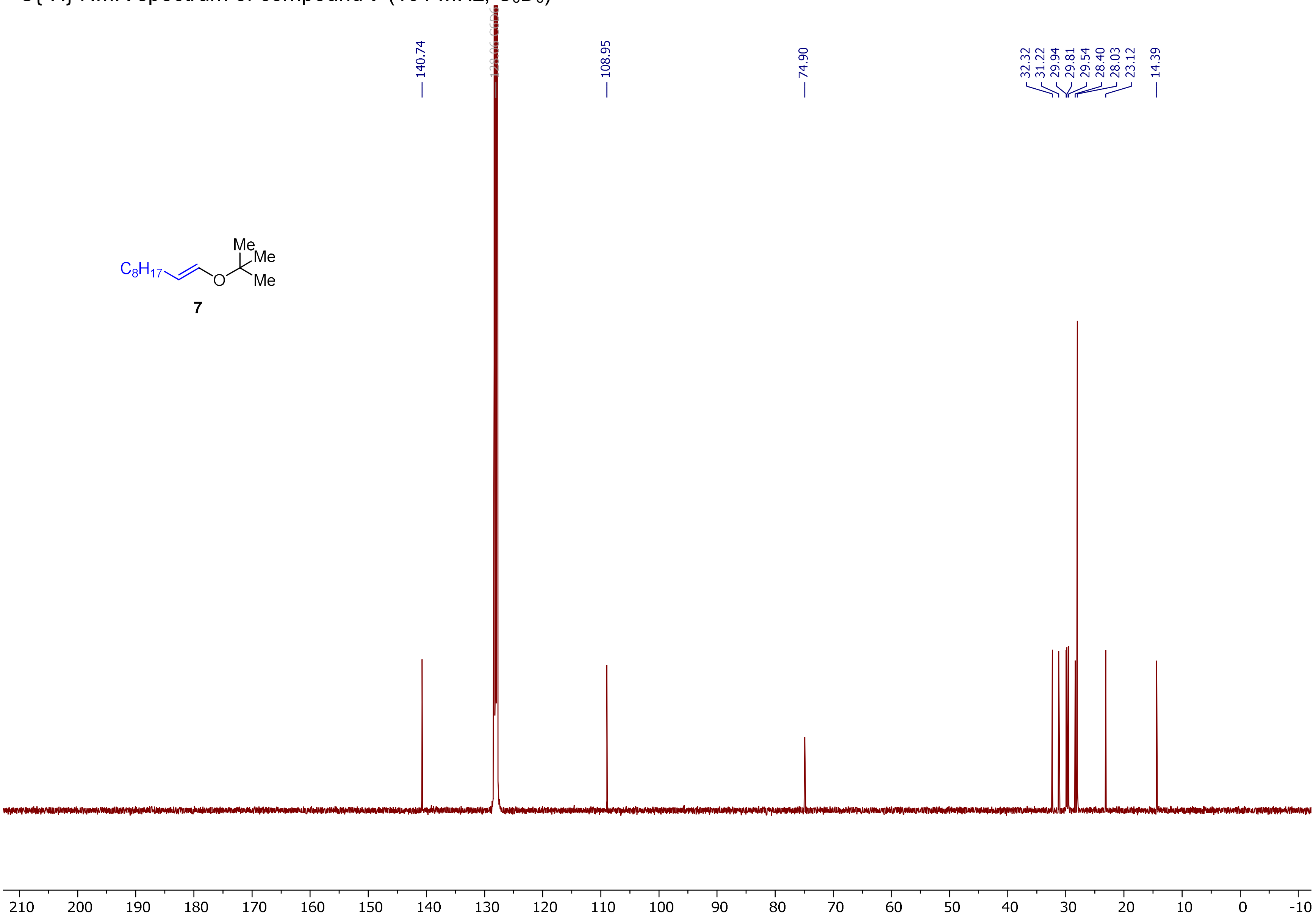

^{1}H NMR spectrum of compound **10** (400 MHz, $CDCl_3$)

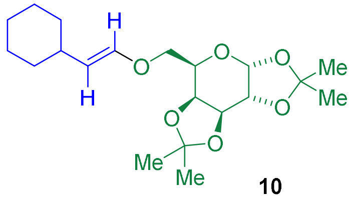

10

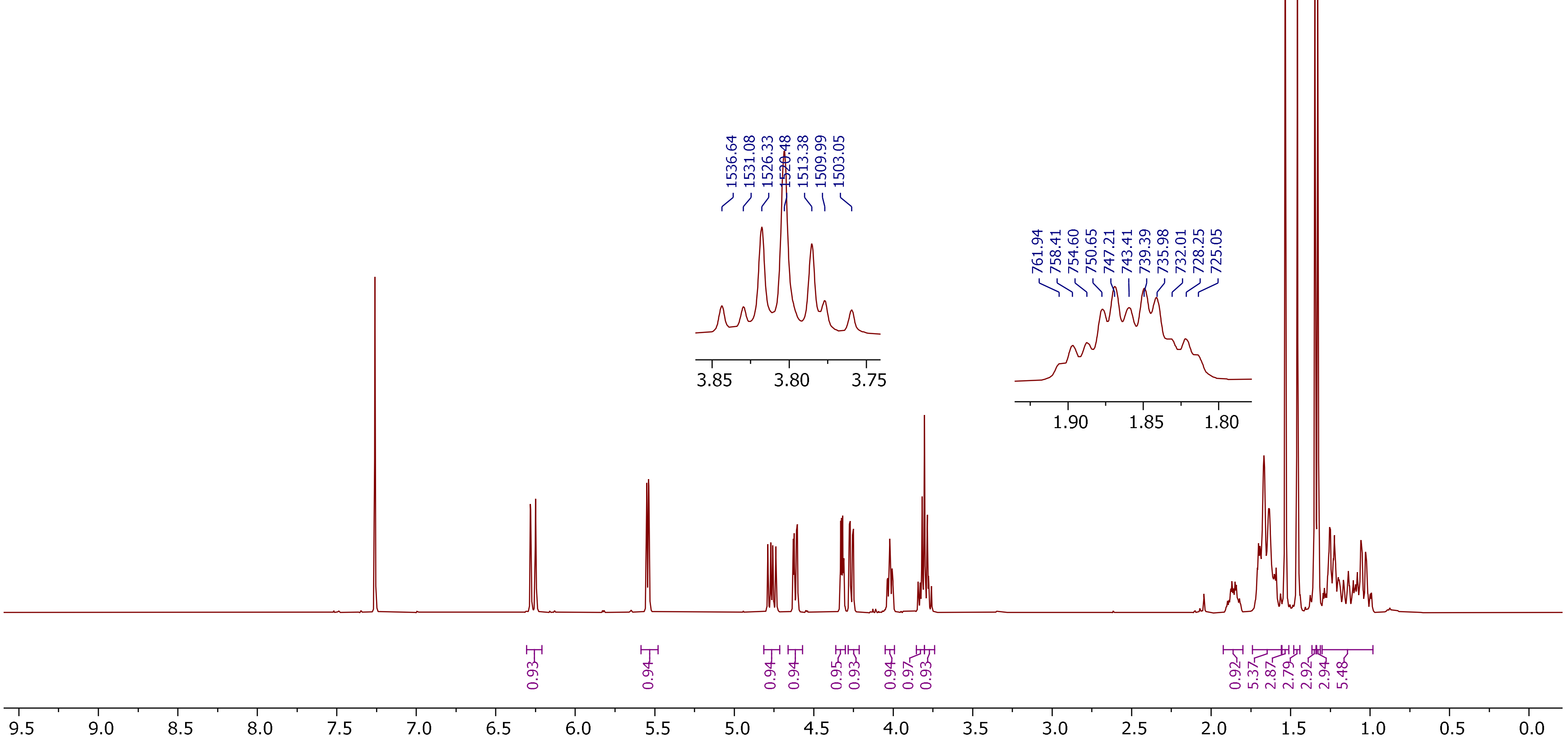

^{1}H NMR spectrum of compound **10** (400 MHz, C_6D_6)

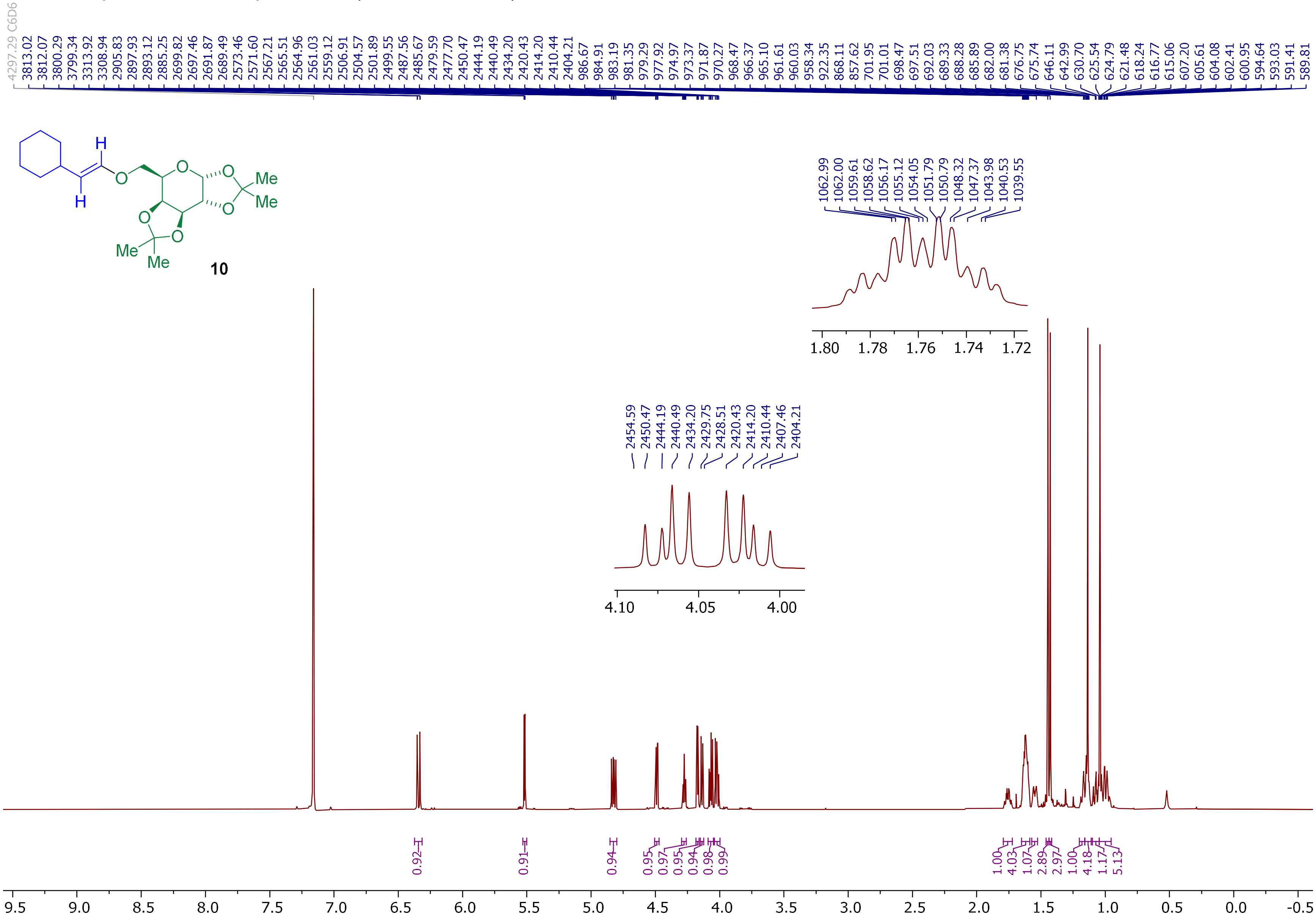

^{1}H NMR spectrum of compound **11** (400 MHz, C_6D_6) – containing trace impurity

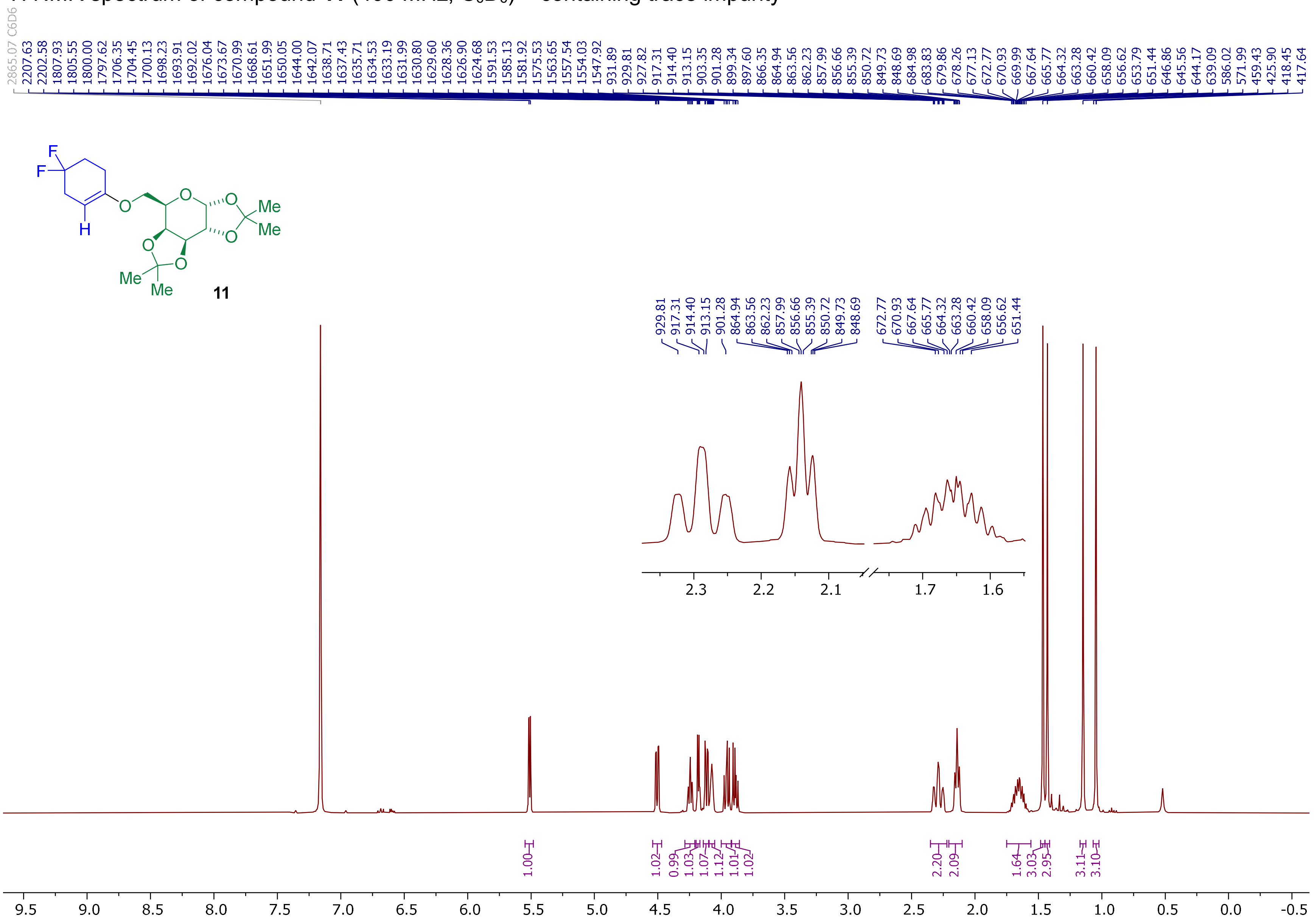

$^{13}C\{^1H\}$ NMR spectrum of compound **11** (101 MHz, C_6D_6) – containing trace impurity

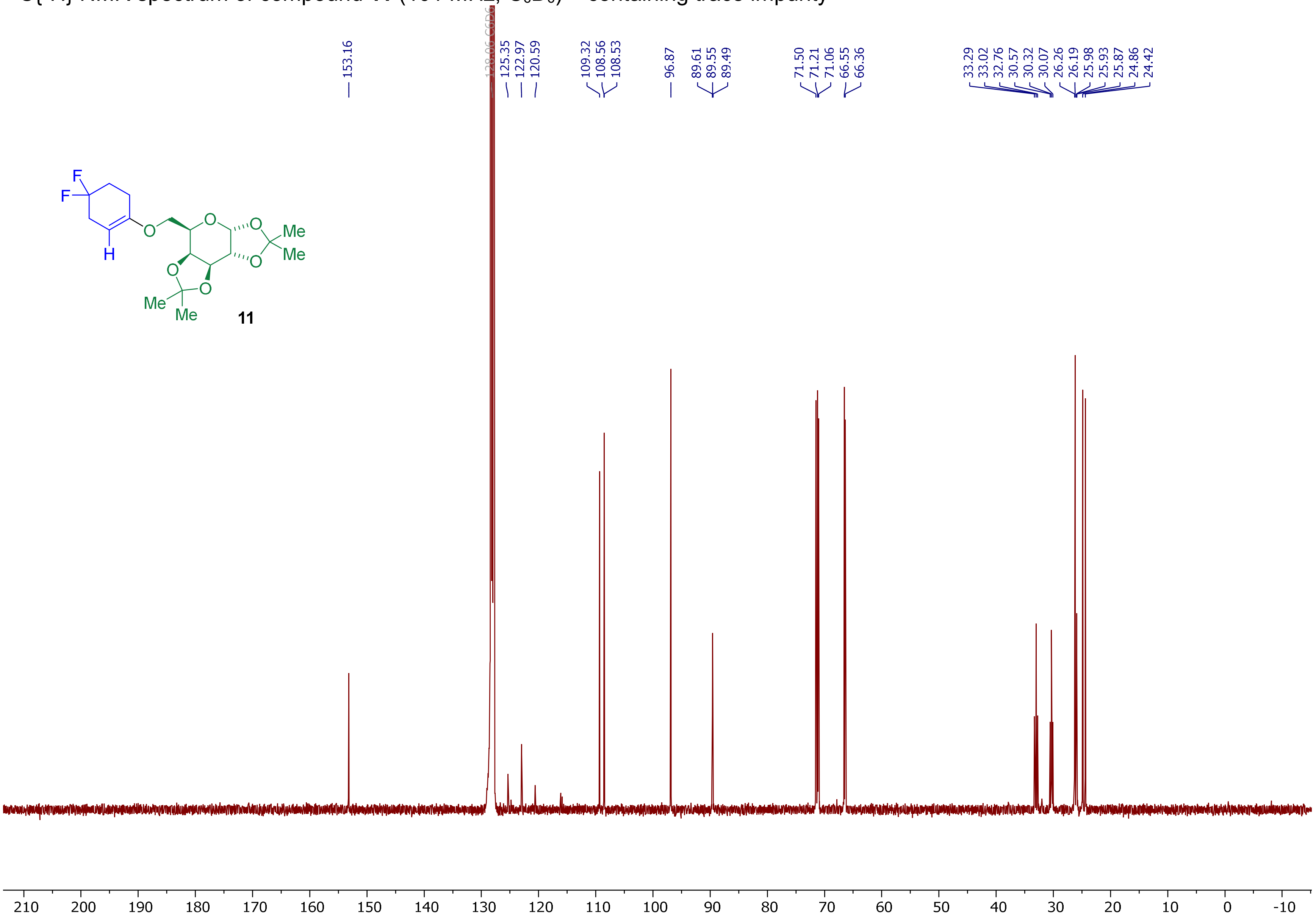

¹⁹F NMR spectrum of compound **11** (376 MHz, C_6D_6)

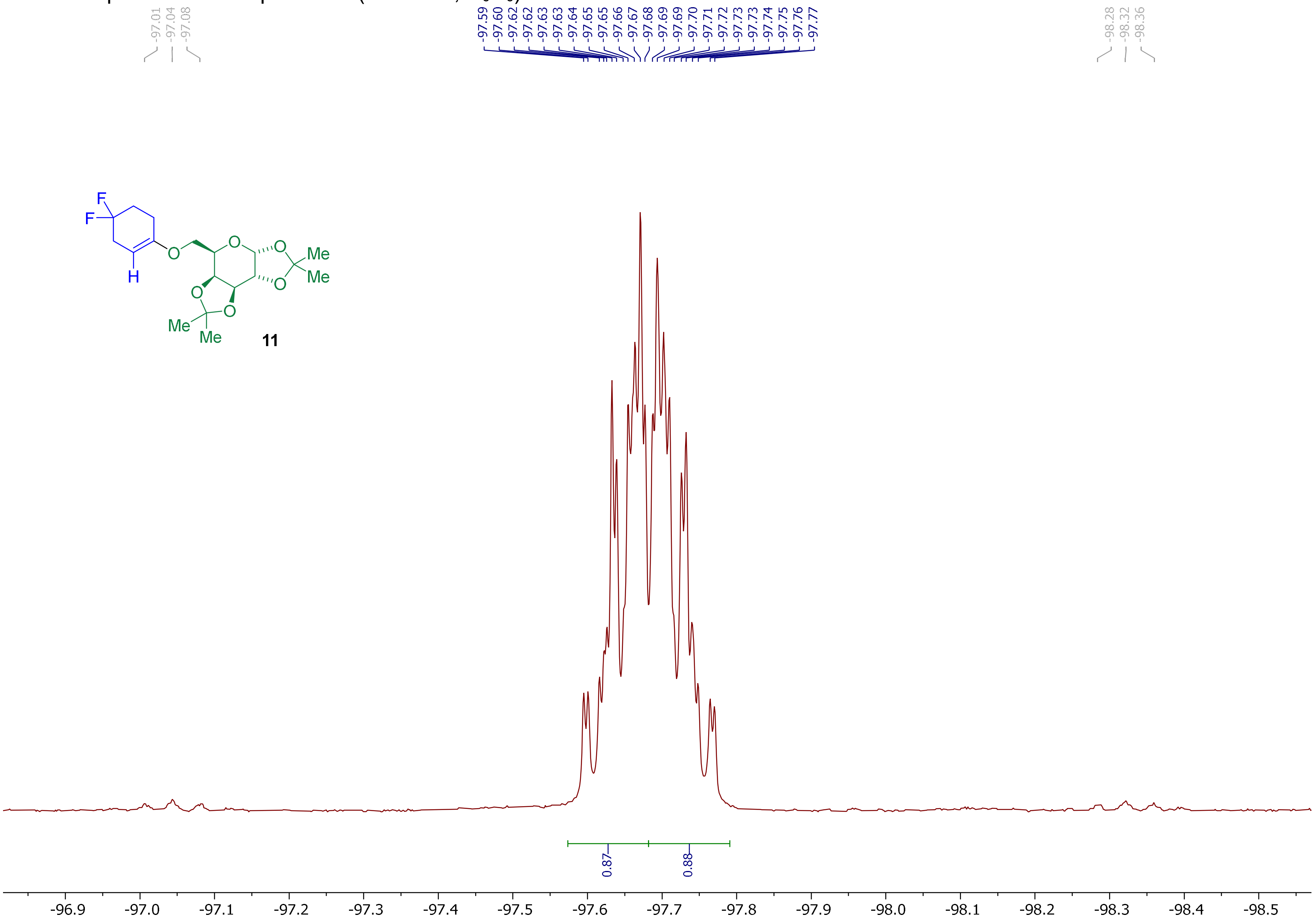

^{1}H NMR spectrum of compound **12** (400 MHz, C_6D_6) – containing trace aldehyde decomposition product

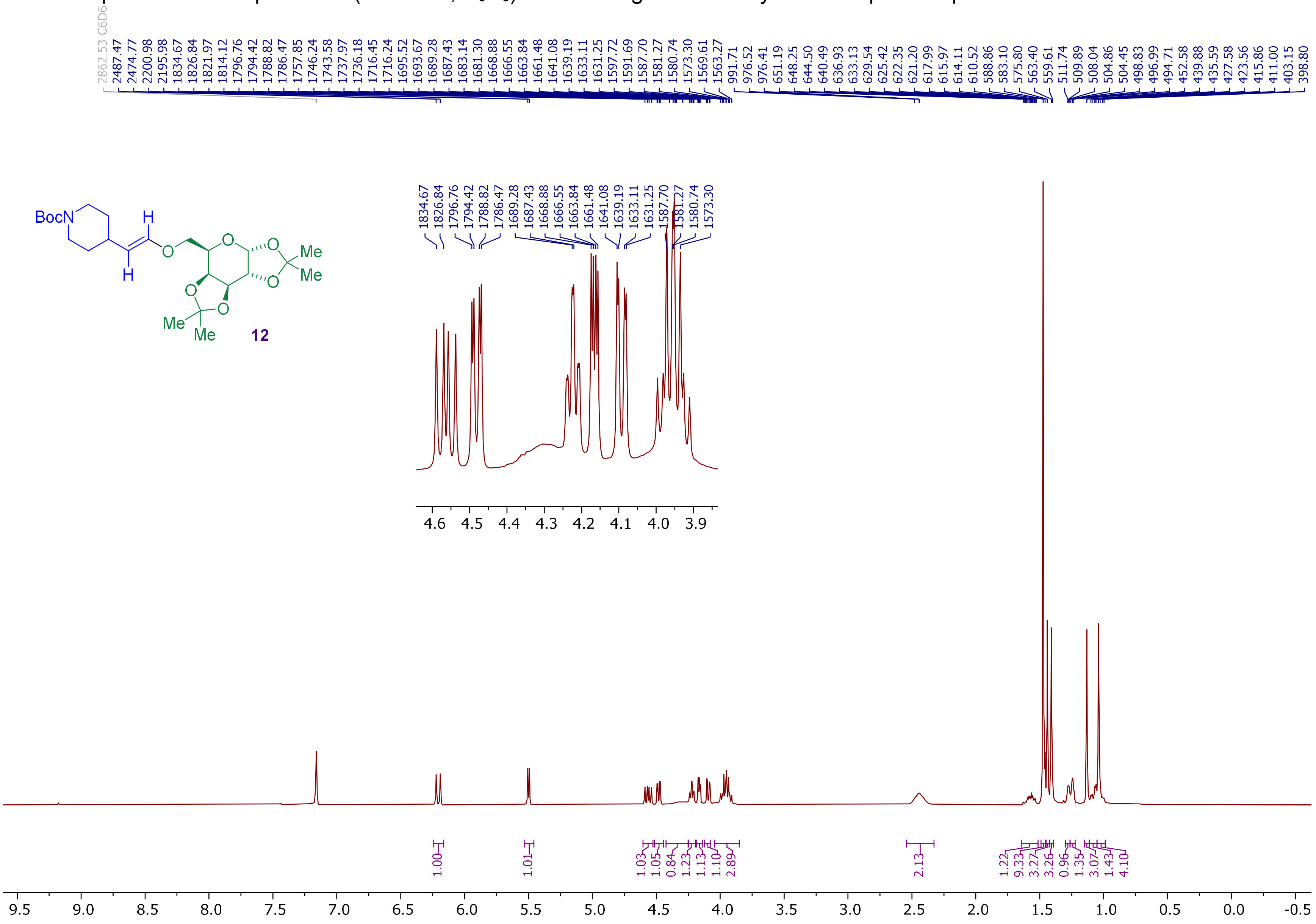

$^{13}C\{^{1}H\}$ NMR spectrum of compound **12** (101 MHz, C_6D_6) - containing trace aldehyde decomposition product

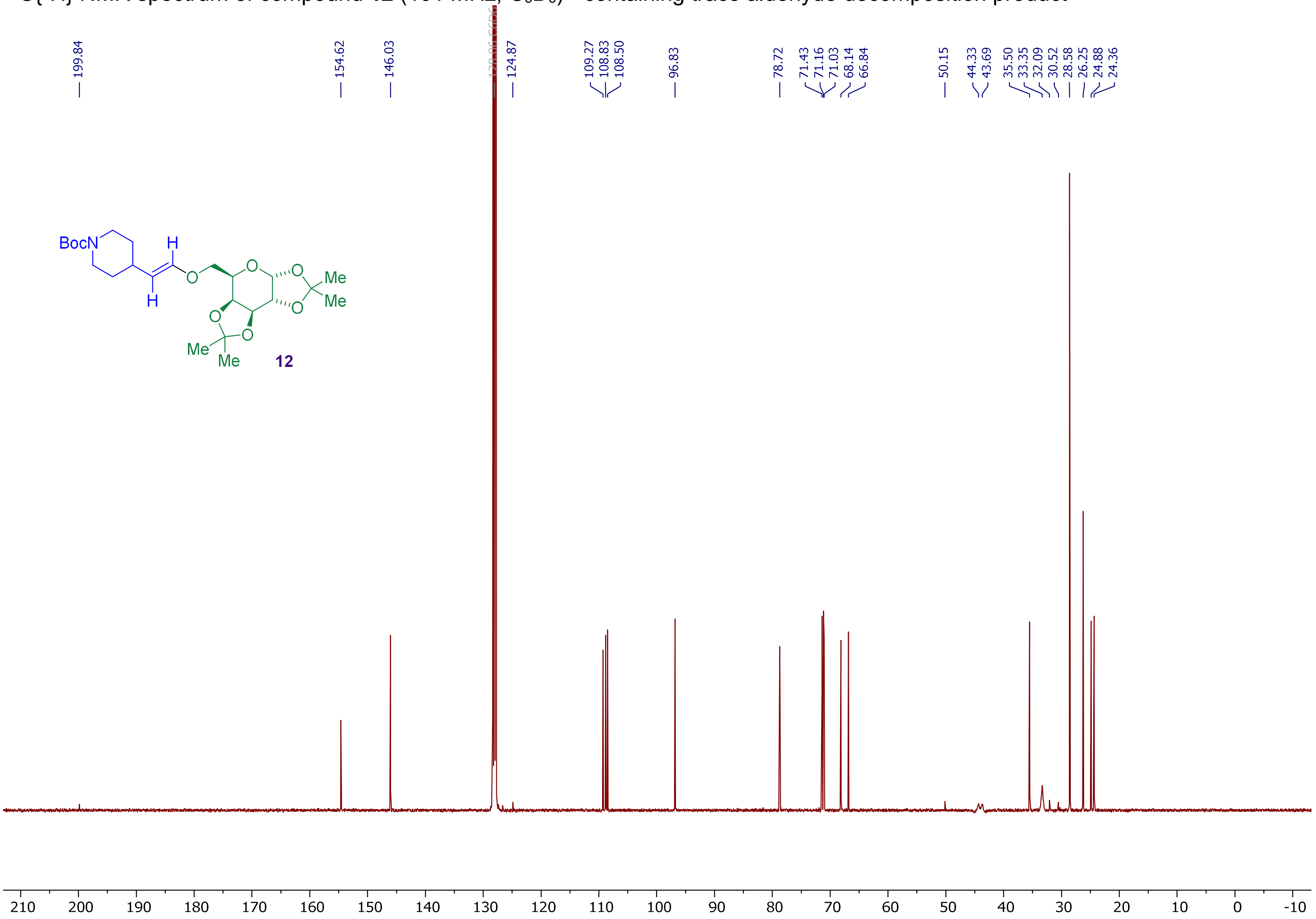

HSQC spectrum of compound **12** (400 MHz, C_6D_6) - containing trace aldehyde decomposition product

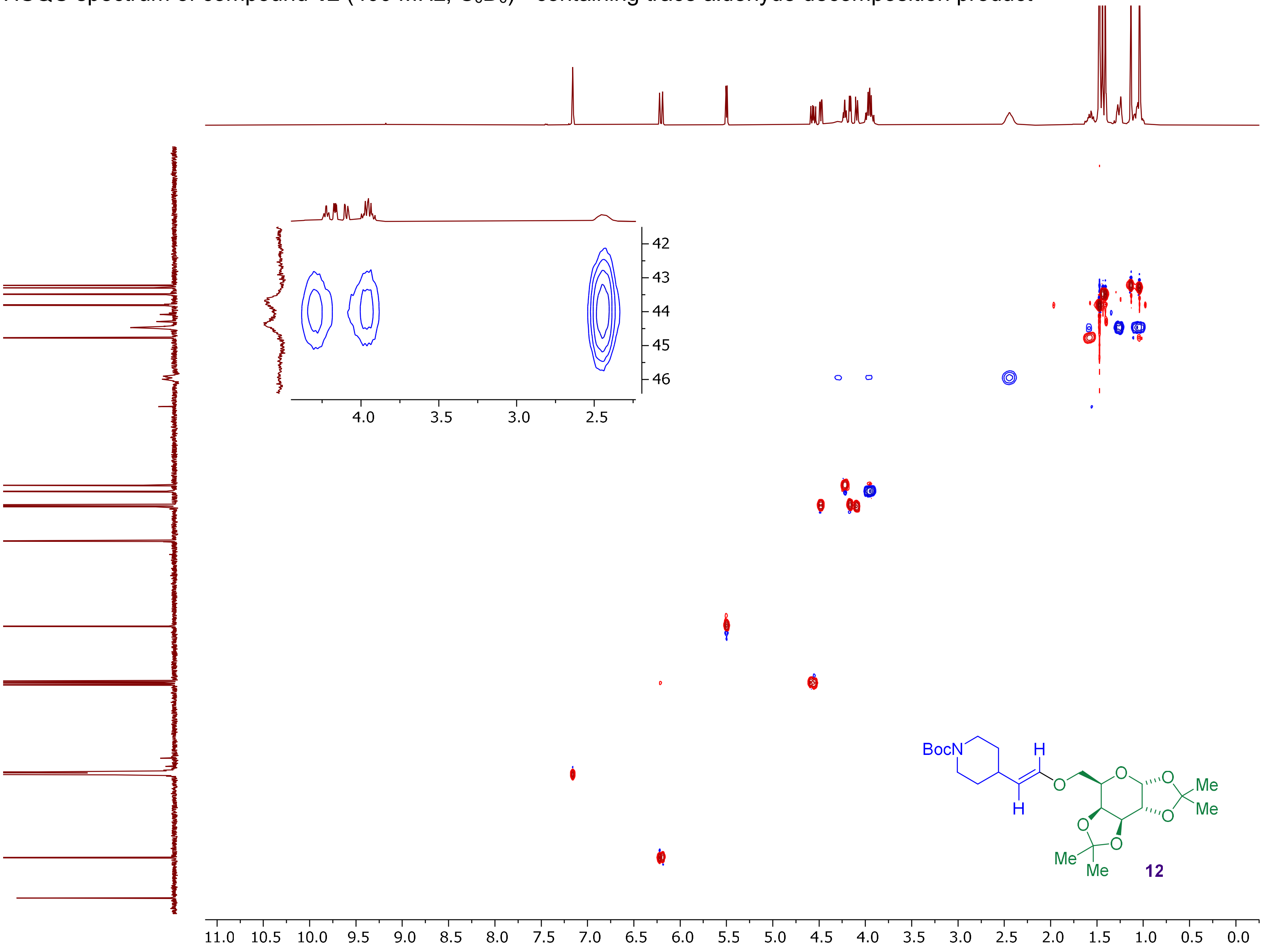

Expansion: HSQC spectrum of compound **12** (400 MHz, C_6D_6)

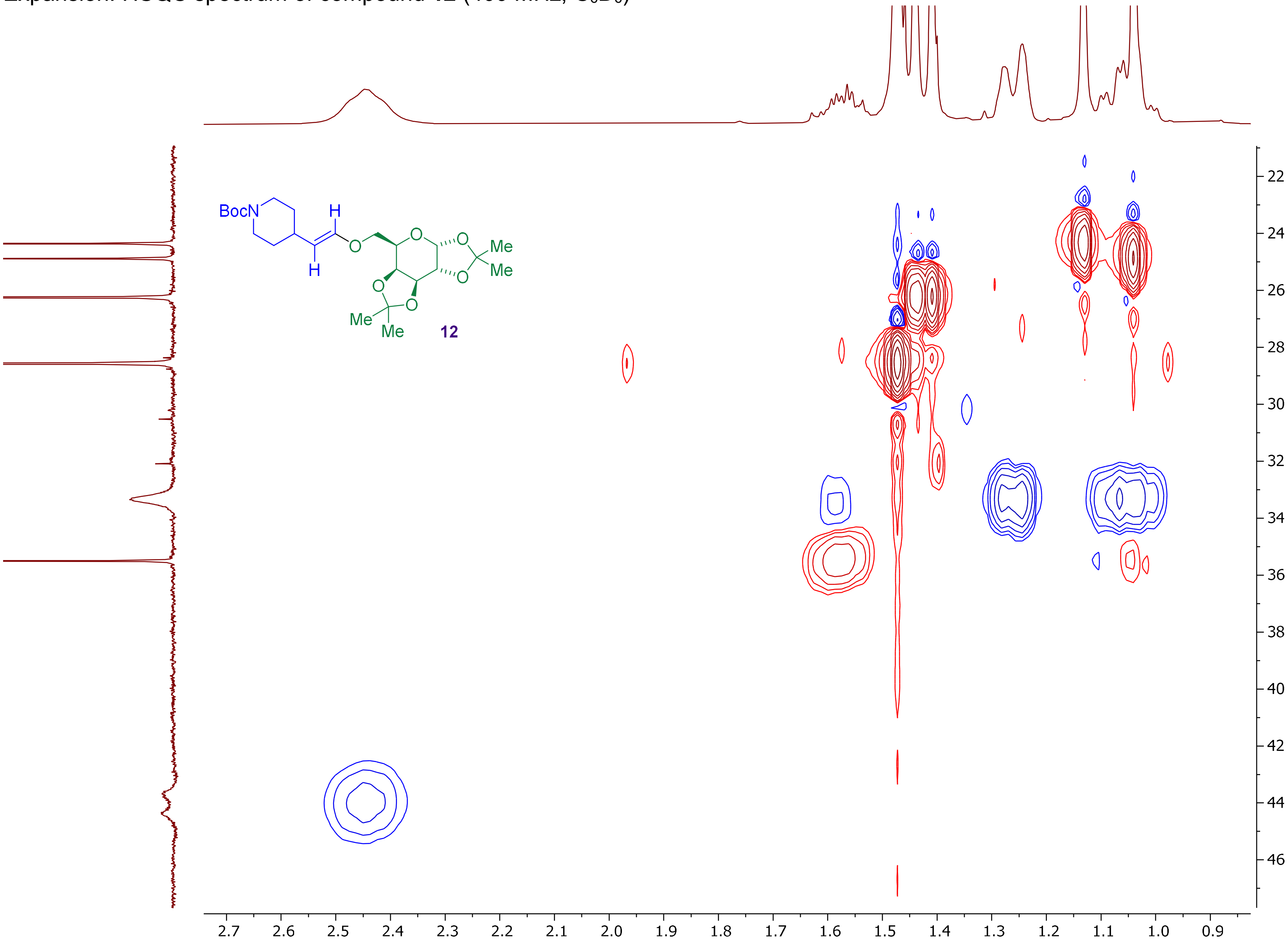

^{1}H NMR spectrum of compound **13** (600 MHz, C_6D_6, 60 °C)

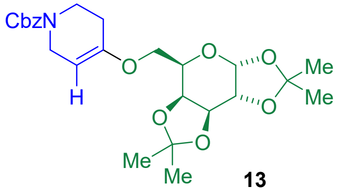

13

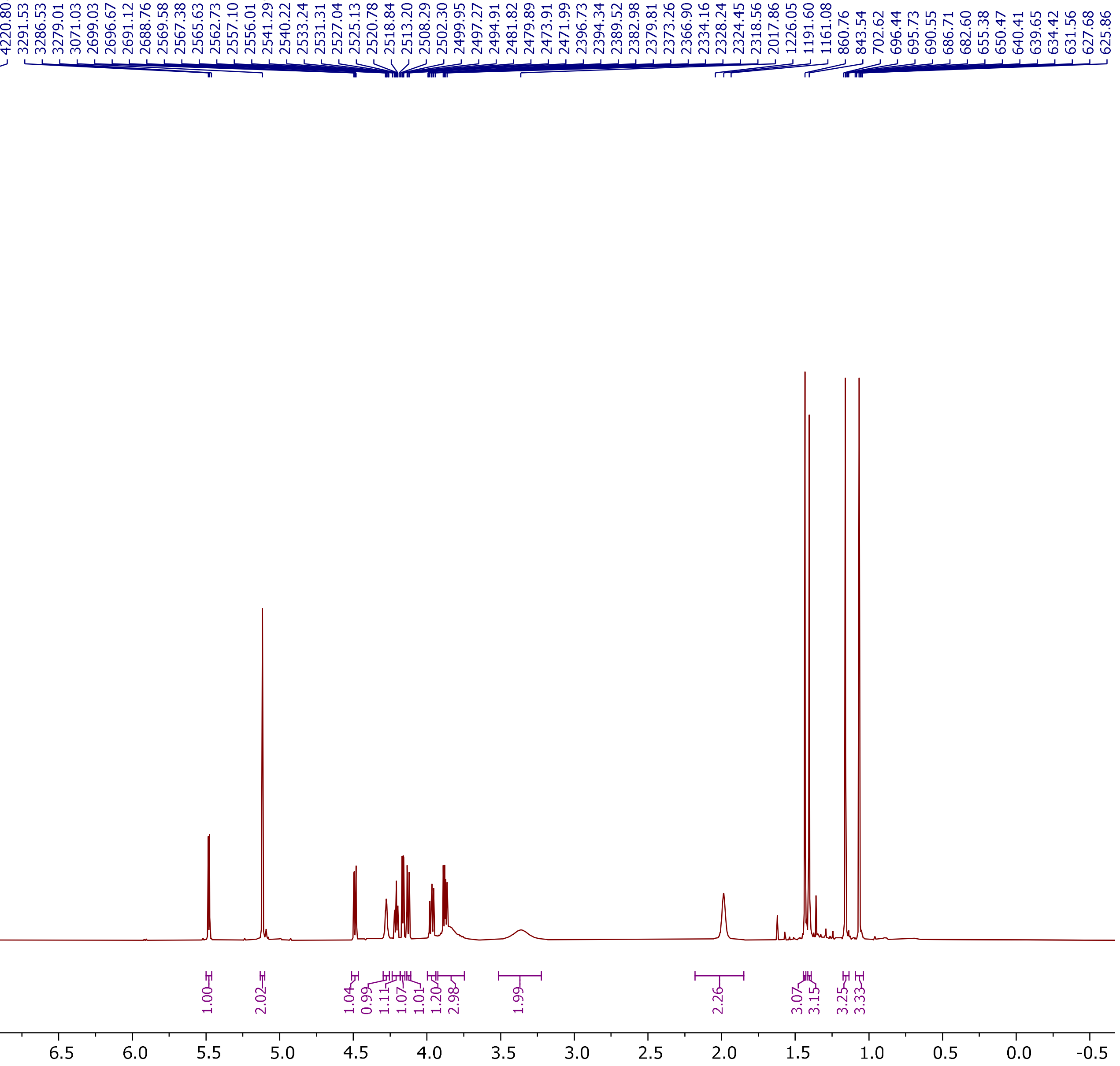

$^{13}C\{^1H\}$ NMR spectrum of compound **13** (151 MHz, C_6D_6, 60 °C)

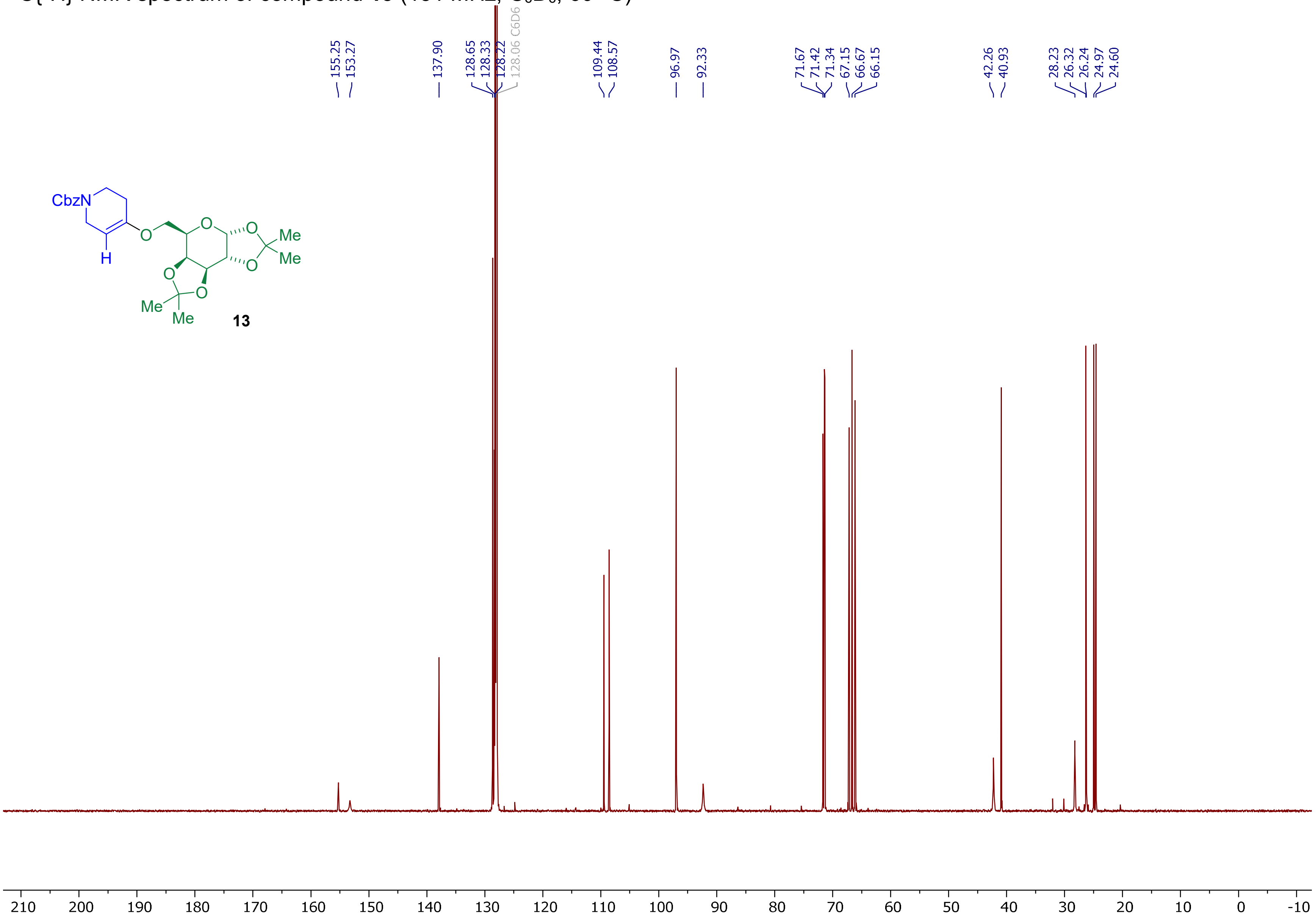

HMBC spectrum of compound **13** (600 MHz, C_6D_6, 60 °C)

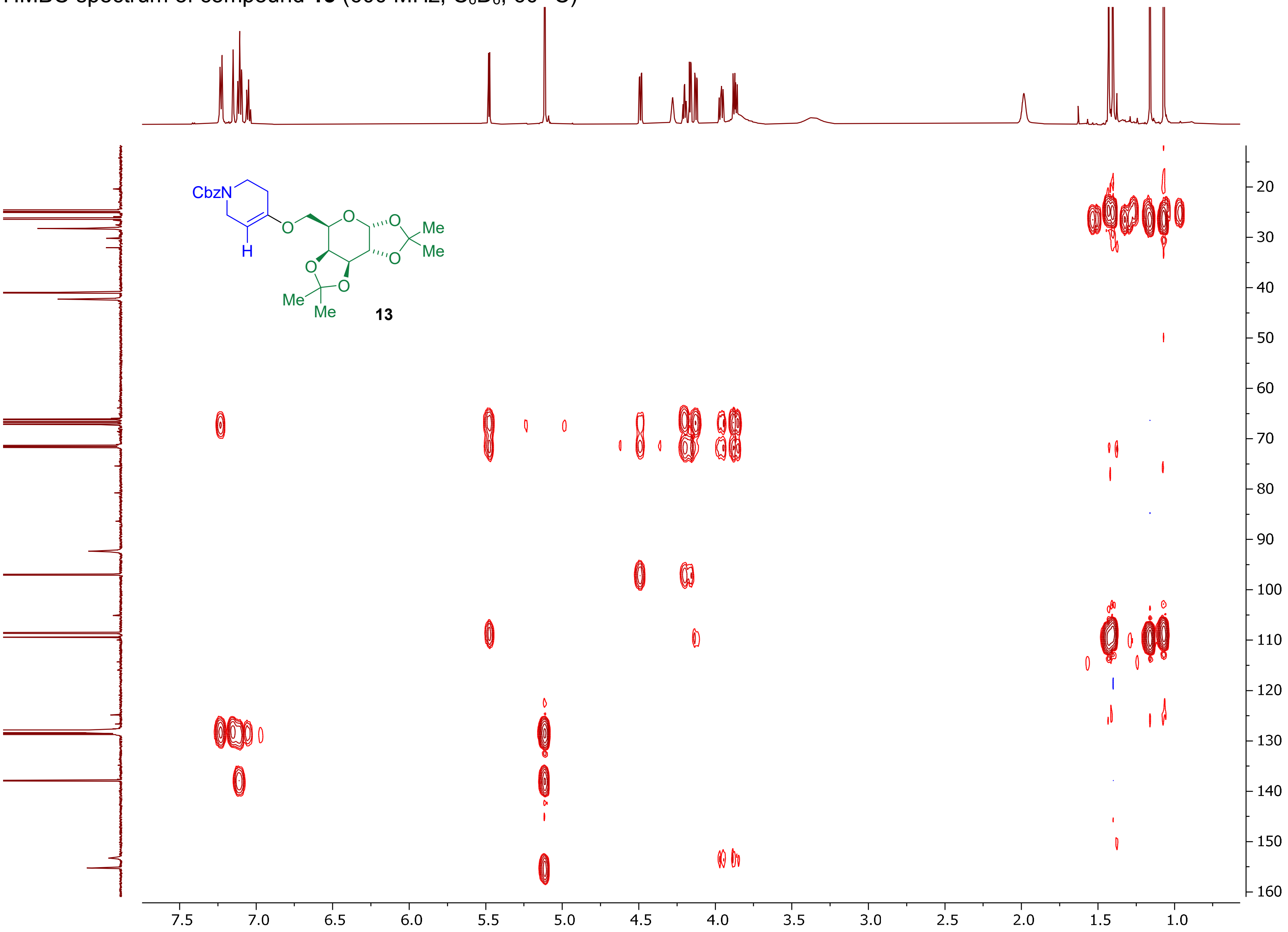

^{1}H NMR spectrum of compound **13** (400 MHz, C_6D_6, 20 °C)

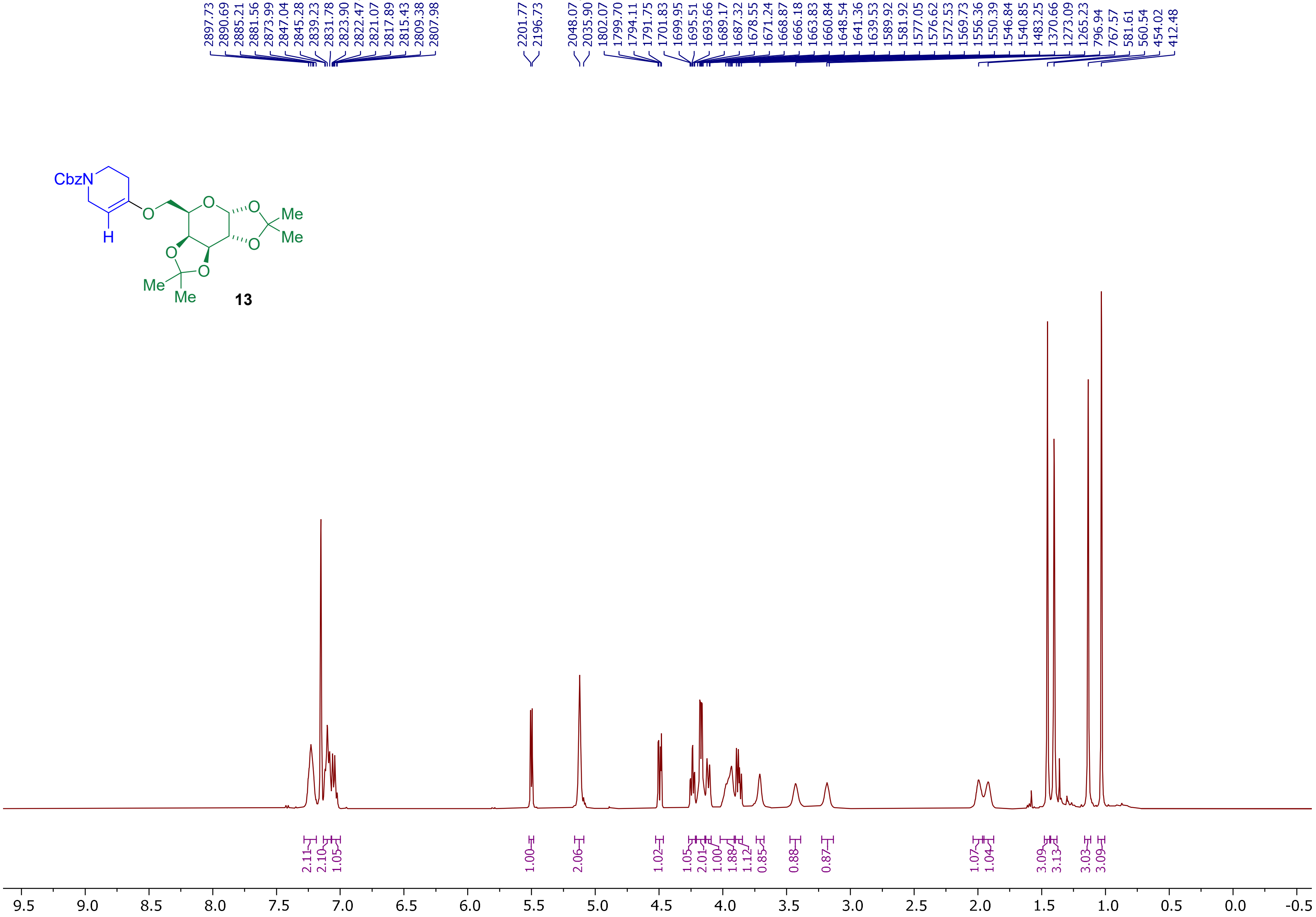

$^{13}C\{^{1}H\}$ NMR spectrum of compound **13** (400 MHz, C_6D_6, 20 °C)

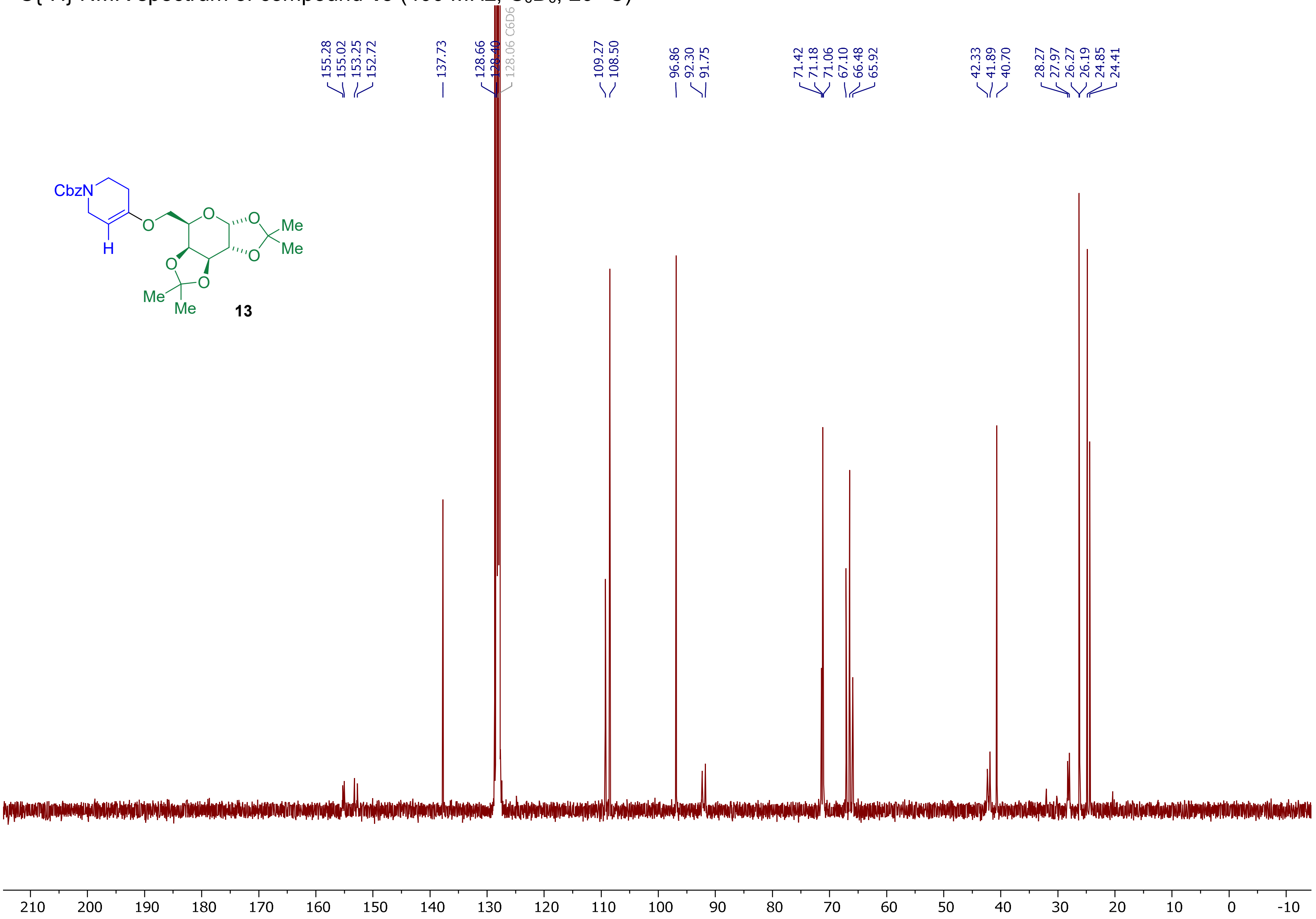

HSQC spectrum of compound **13** (400 MHz, C_6D_6, 20 °C)

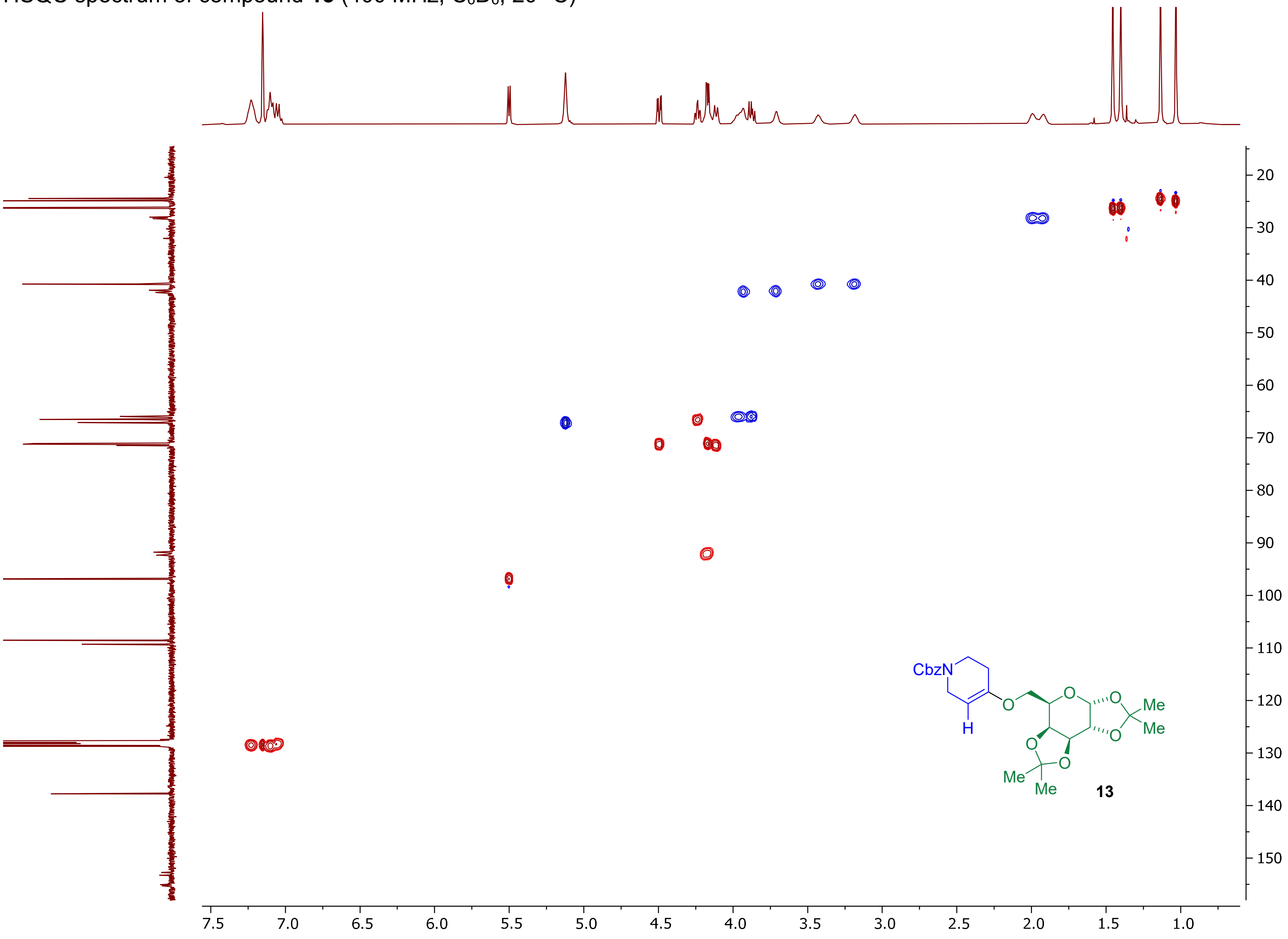

HMBC spectrum of compound **13** (400 MHz, C_6D_6, 20 °C)

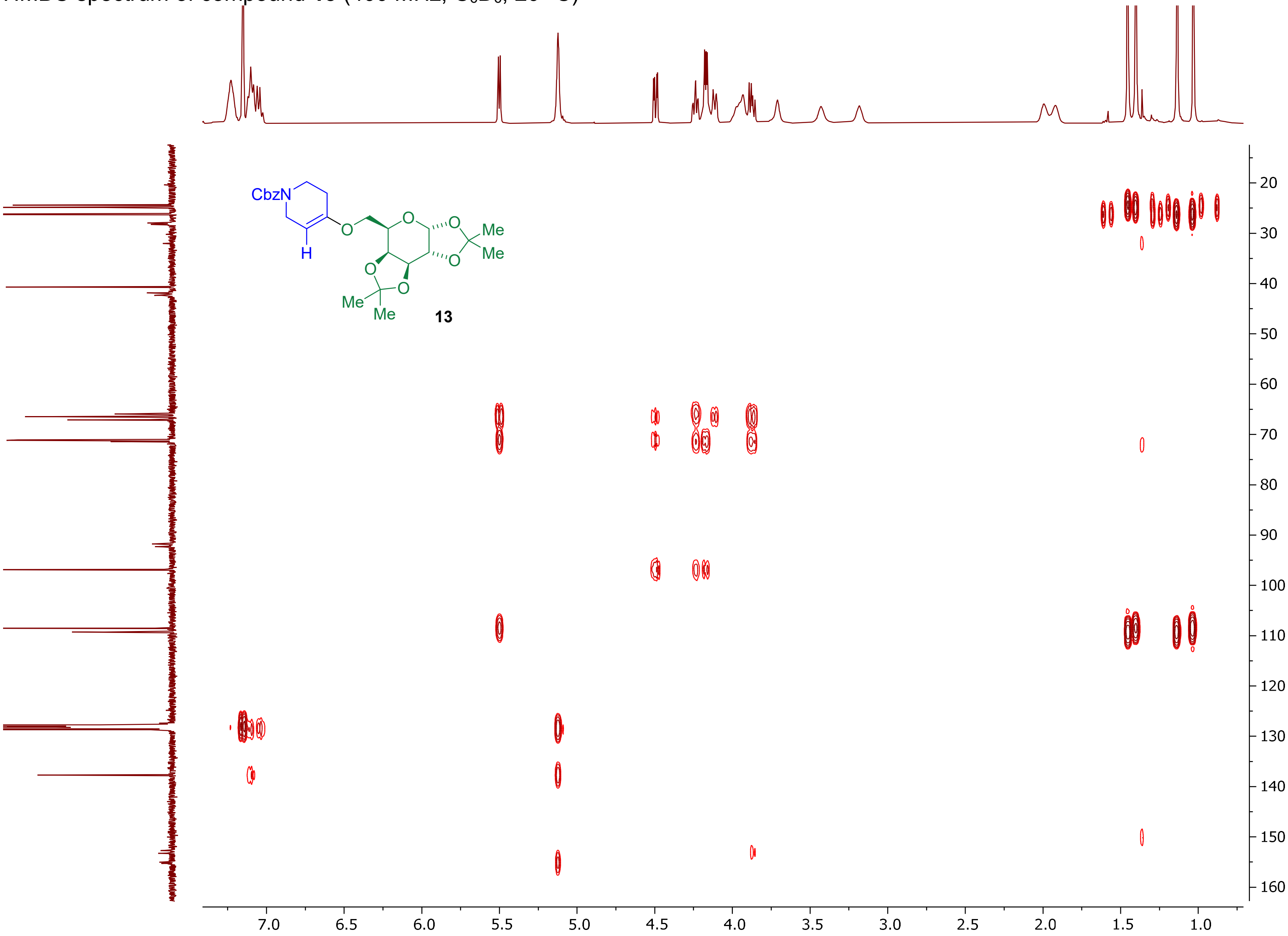

^{1}H NMR spectrum of compound **14** (400 MHz, C_6D_6)

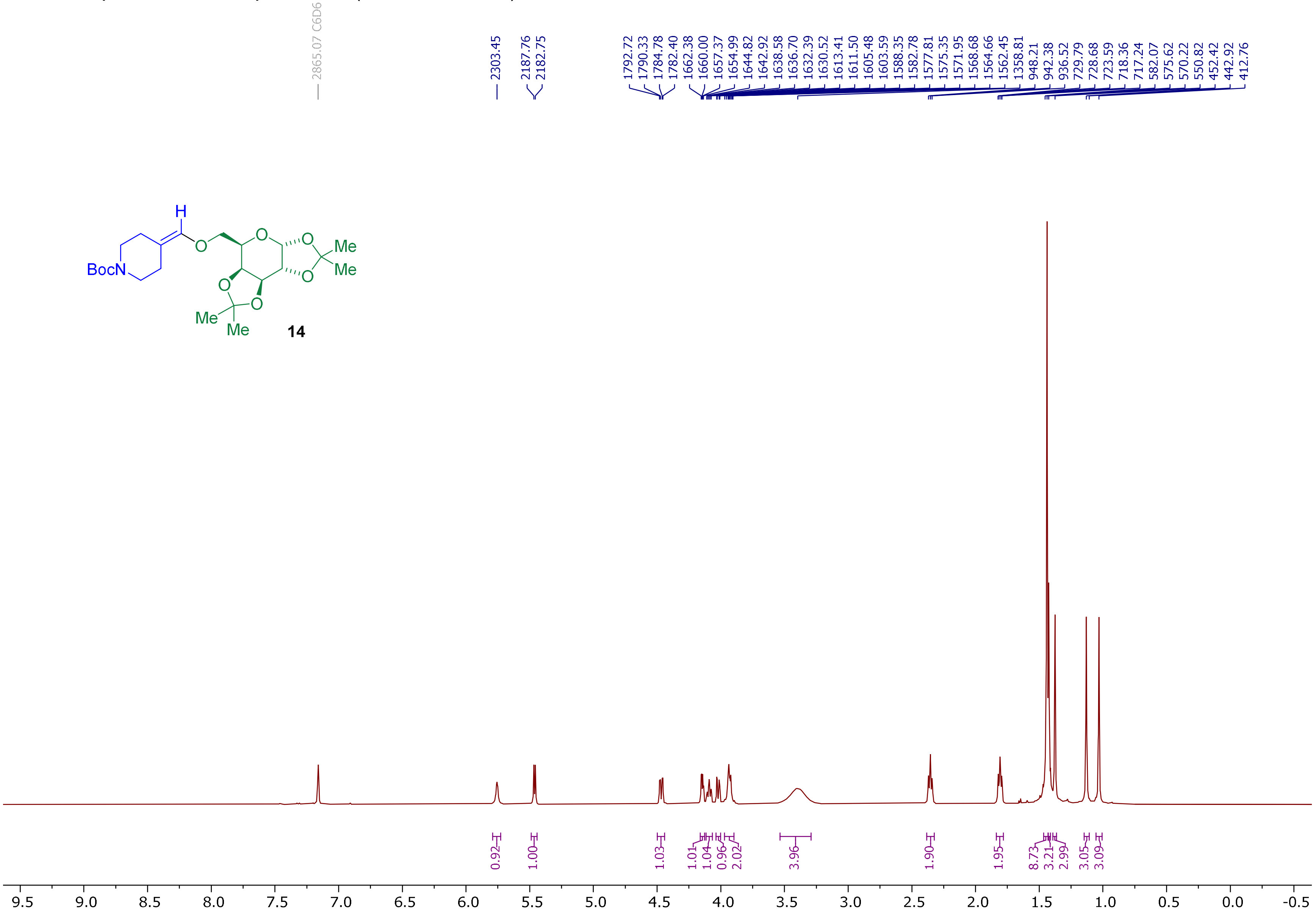

^{1}H NMR spectrum of compound **14** (600 MHz, C_6D_6) – 8-second relaxation delay *peaks are not as defined as 400 MHz spectrum

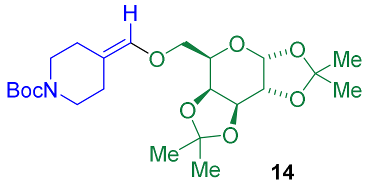

14

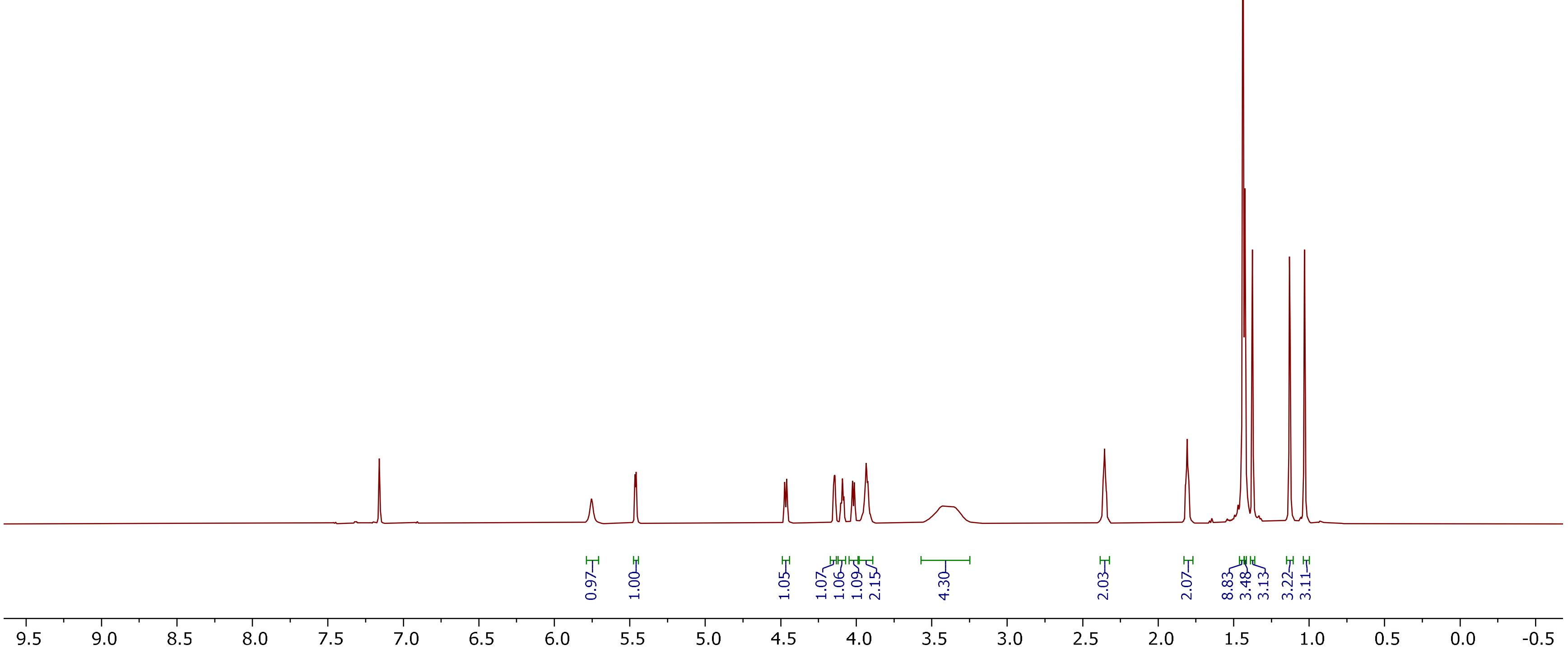

$^{13}C\{^{1}H\}$ NMR spectrum of compound **14** (151 MHz, C_6D_6) – 8-second relaxation delay

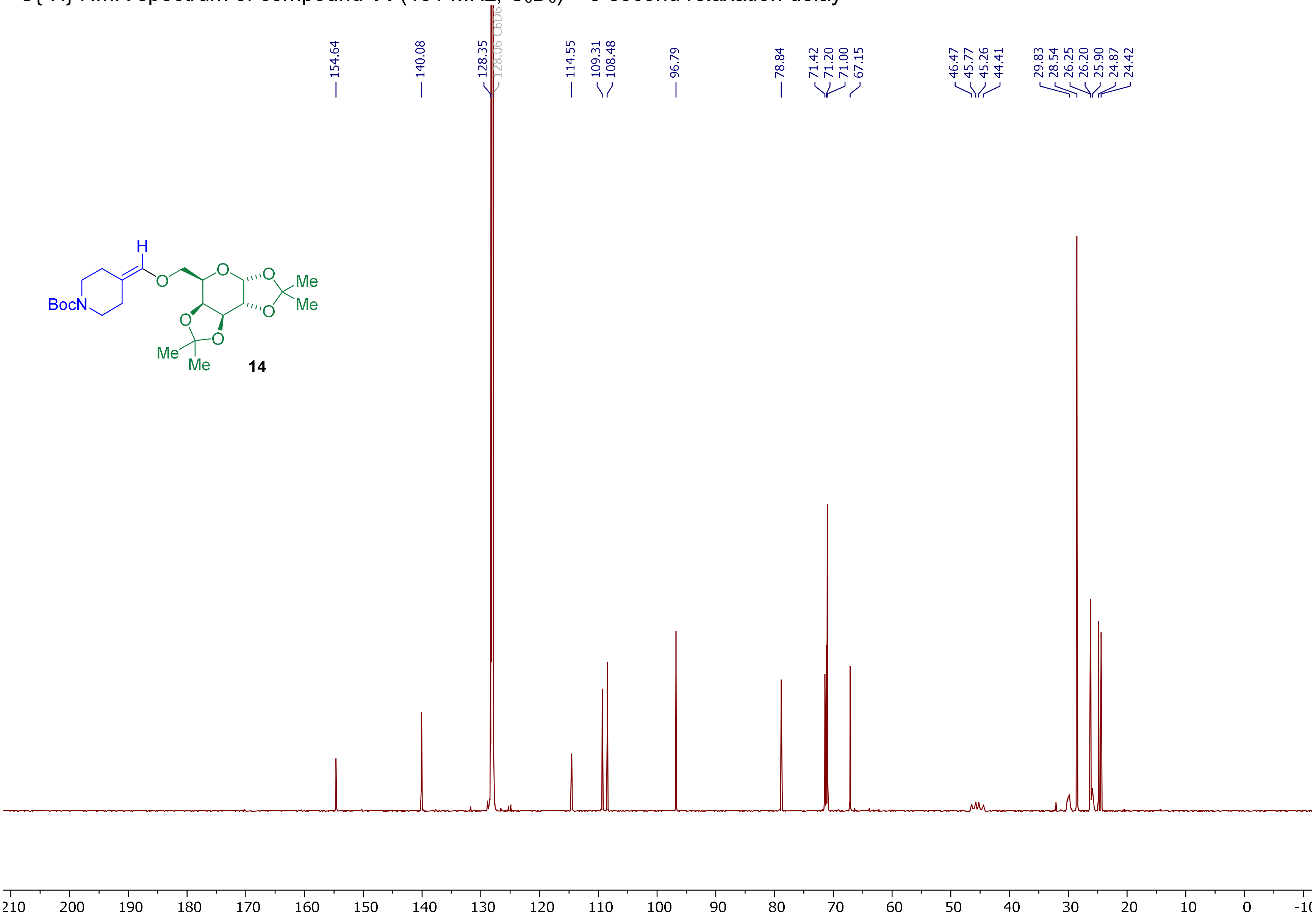

HSQC spectrum of compound **14** (600 MHz, C_6D_6) – 8-second relaxation delay

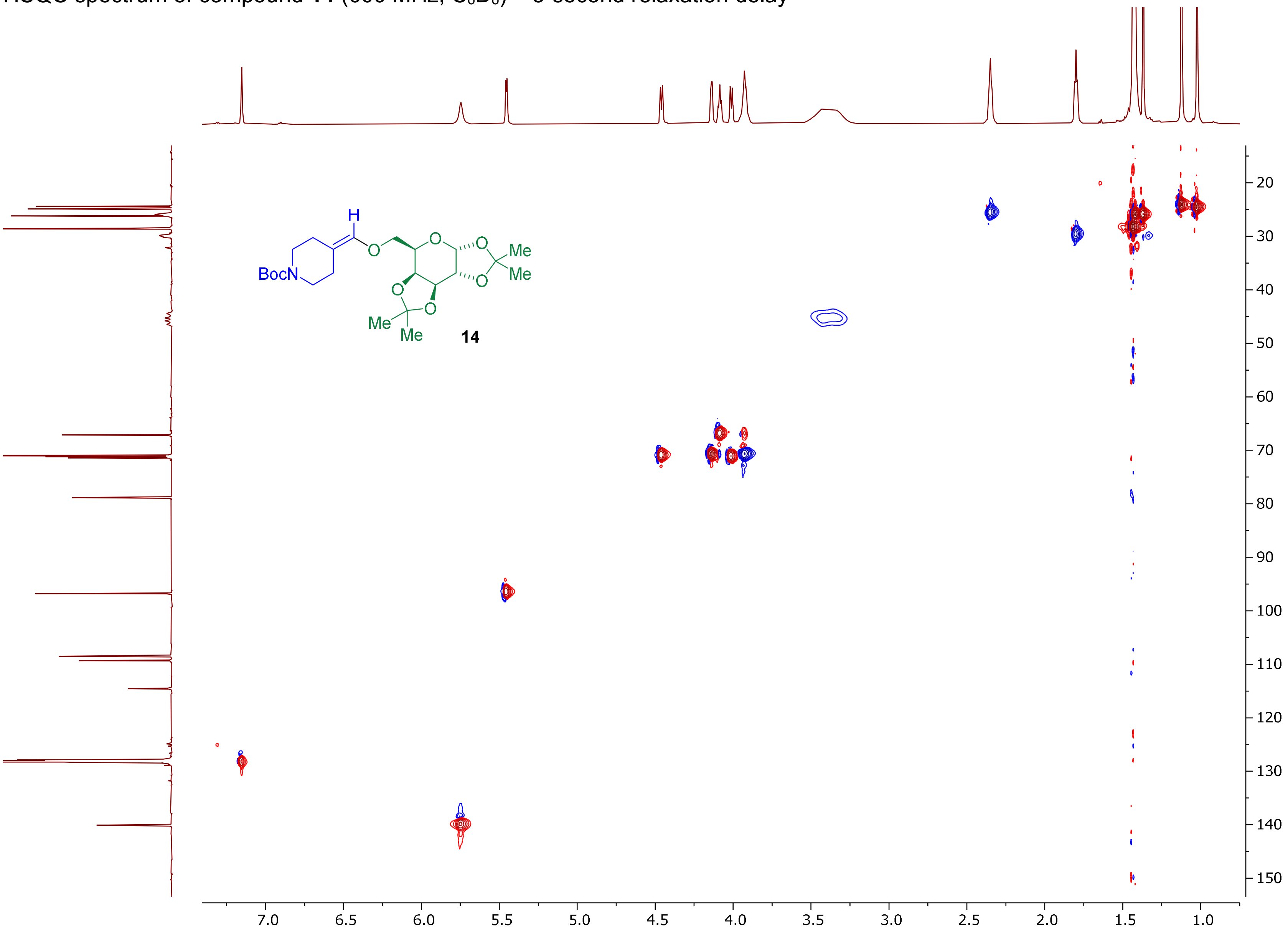

^{1}H NMR spectrum of compound **15** (>10 : 1 *Z*/*E*) (400 MHz, $CDCl_3$)

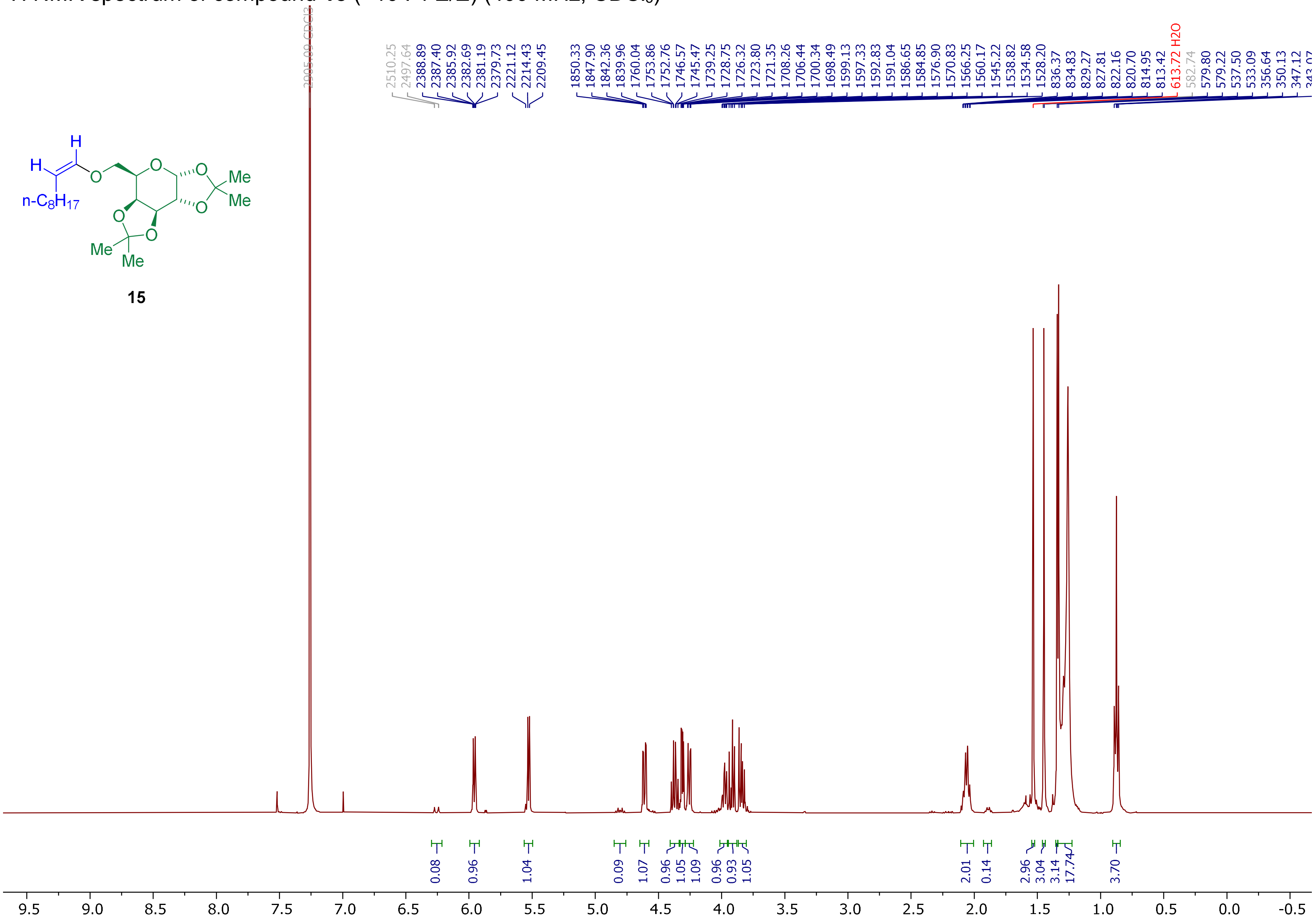

^{1}H NMR spectrum of compound **16** (400 MHz, C_6D_6) – SLP-ON-110-C-VE

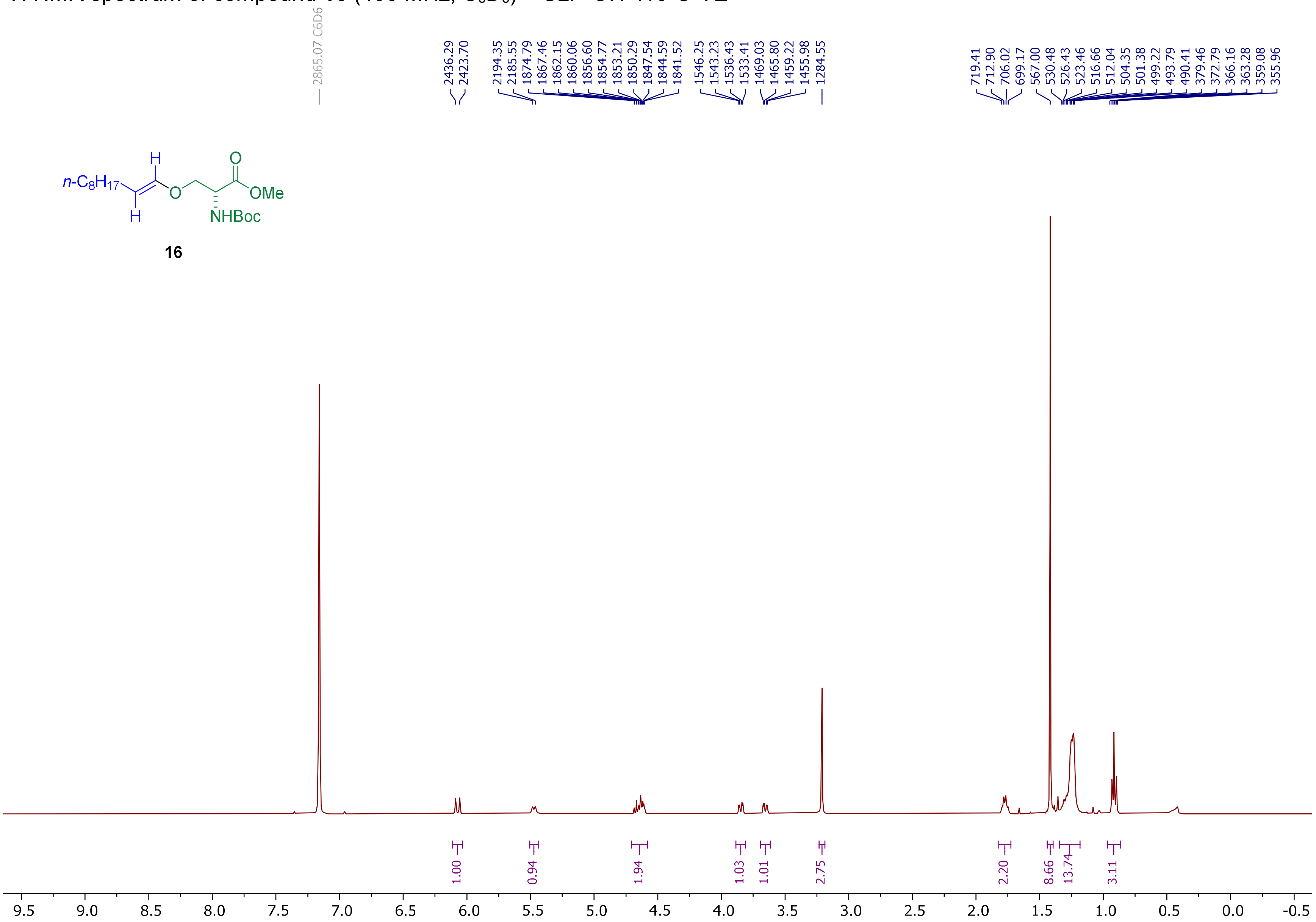

$^{13}C\{^1H\}$ NMR spectrum of compound **16** (101 MHz, C_6D_6)

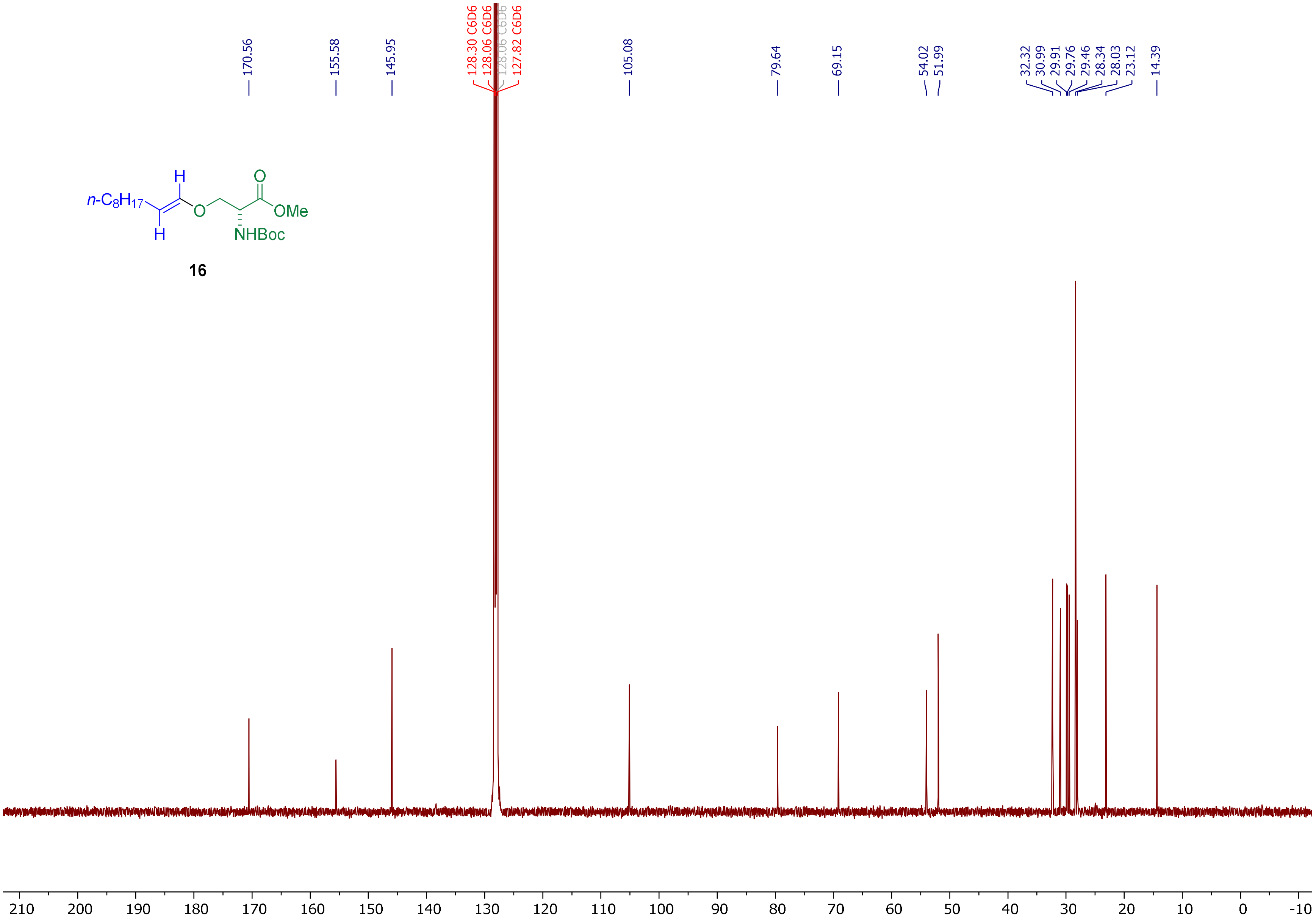

^{1}H NMR spectrum of compound **17** (400 MHz, C_6D_6)

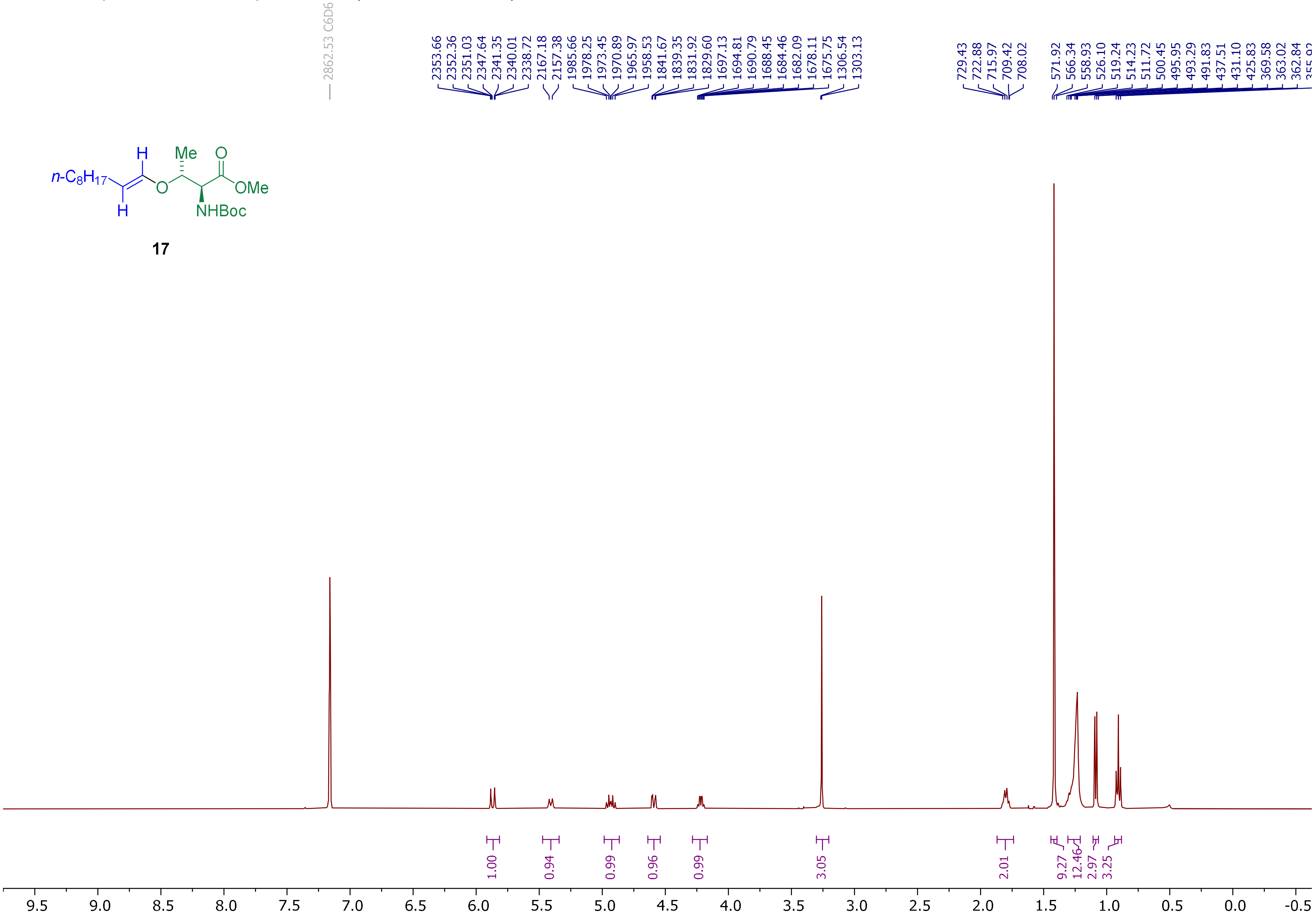

$^{13}C\{^{1}H\}$ NMR spectrum of compound **17** (101 MHz, C_6D_6)

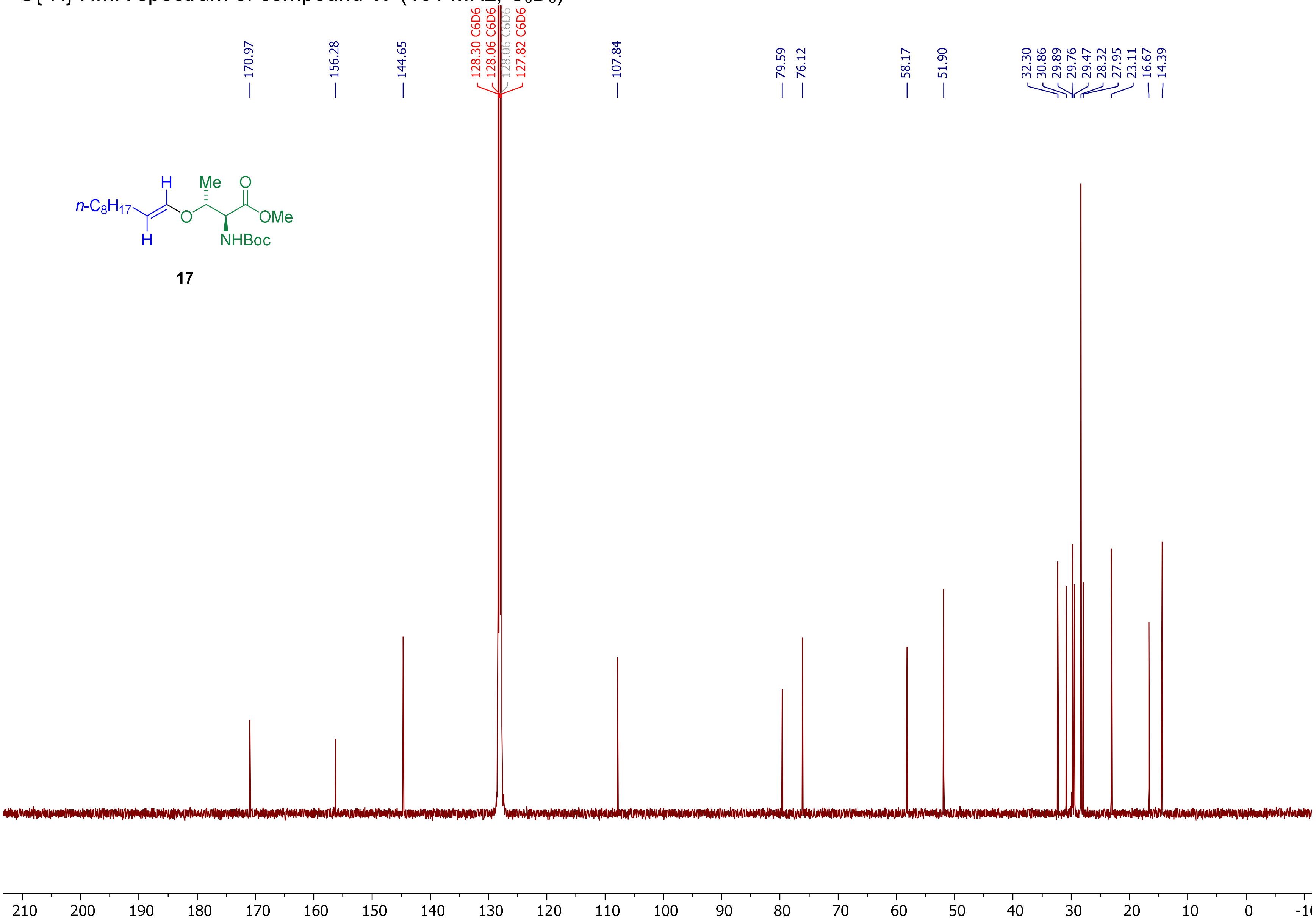

^{1}H NMR spectrum of compound **18** (400 MHz, C_6D_6)

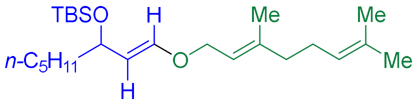

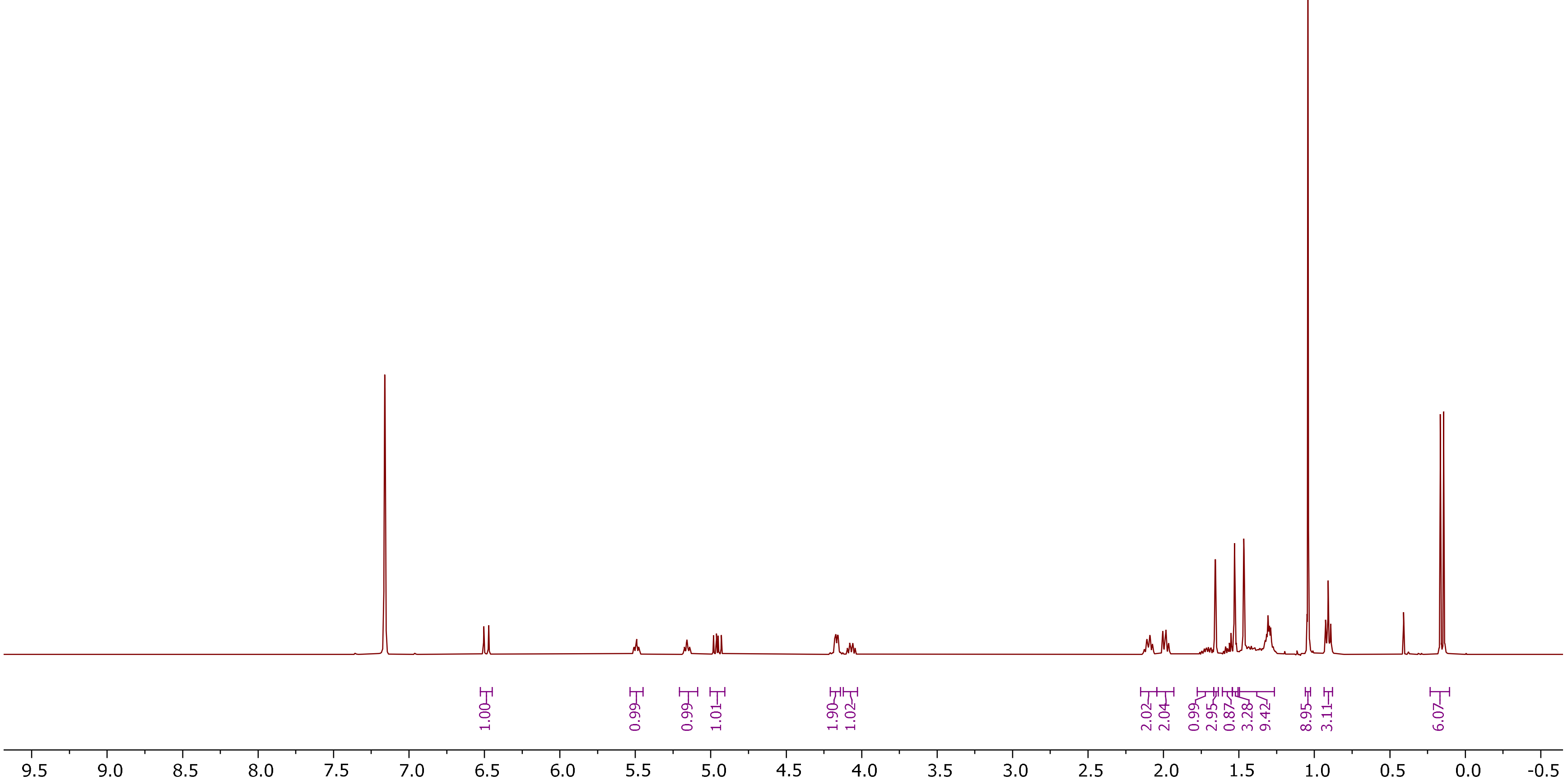

$^{13}C\{^1H\}$ NMR spectrum of compound **18** (101 MHz, C_6D_6)

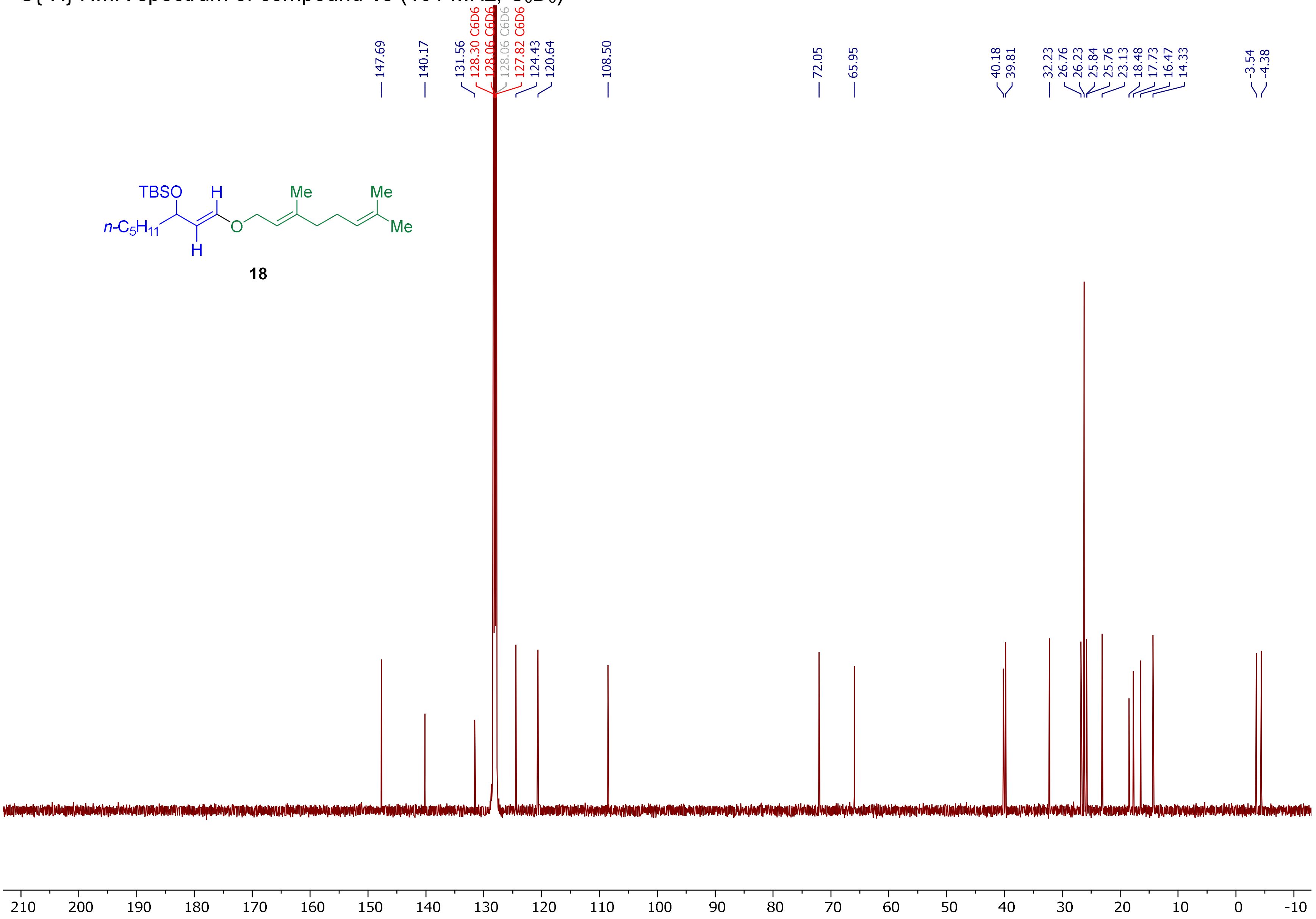

^{1}H NMR spectrum of compound **19** (400 MHz, C_6D_6)

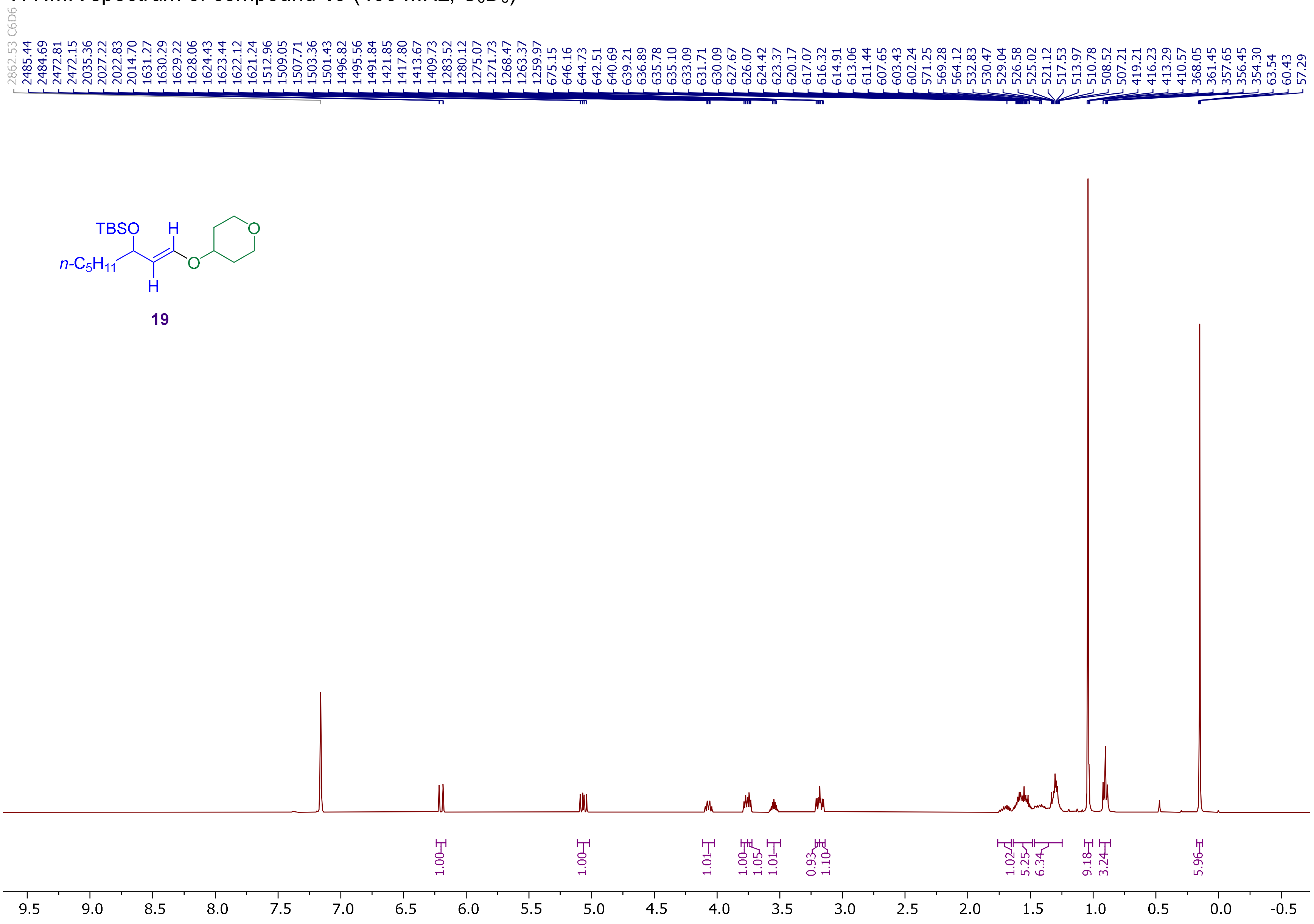

^{1}H NMR spectrum of compound **19** (400 MHz, C_6D_6)

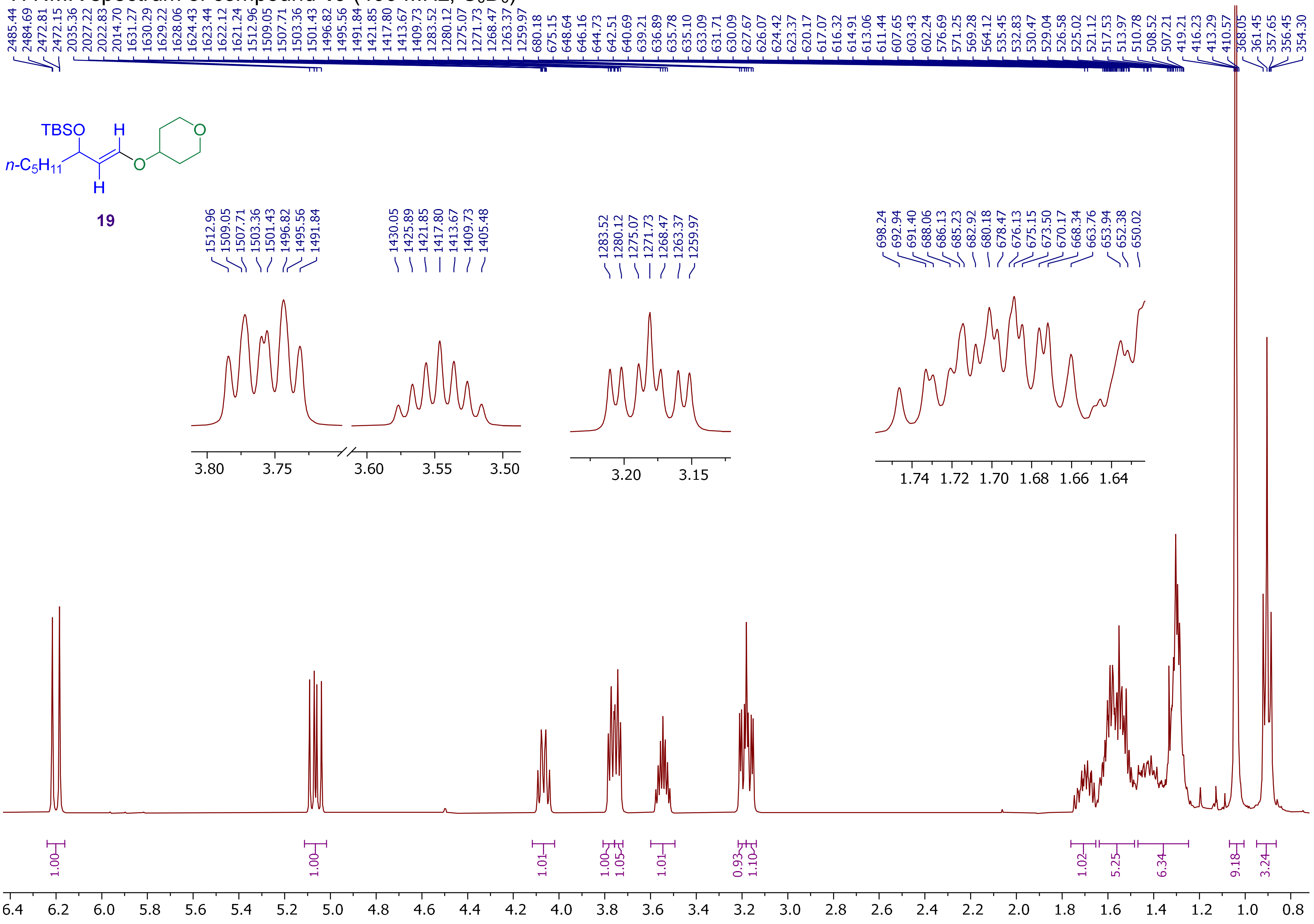

$^{13}C\{^{1}H\}$ NMR spectrum of compound **19** (101 MHz, C_6D_6)

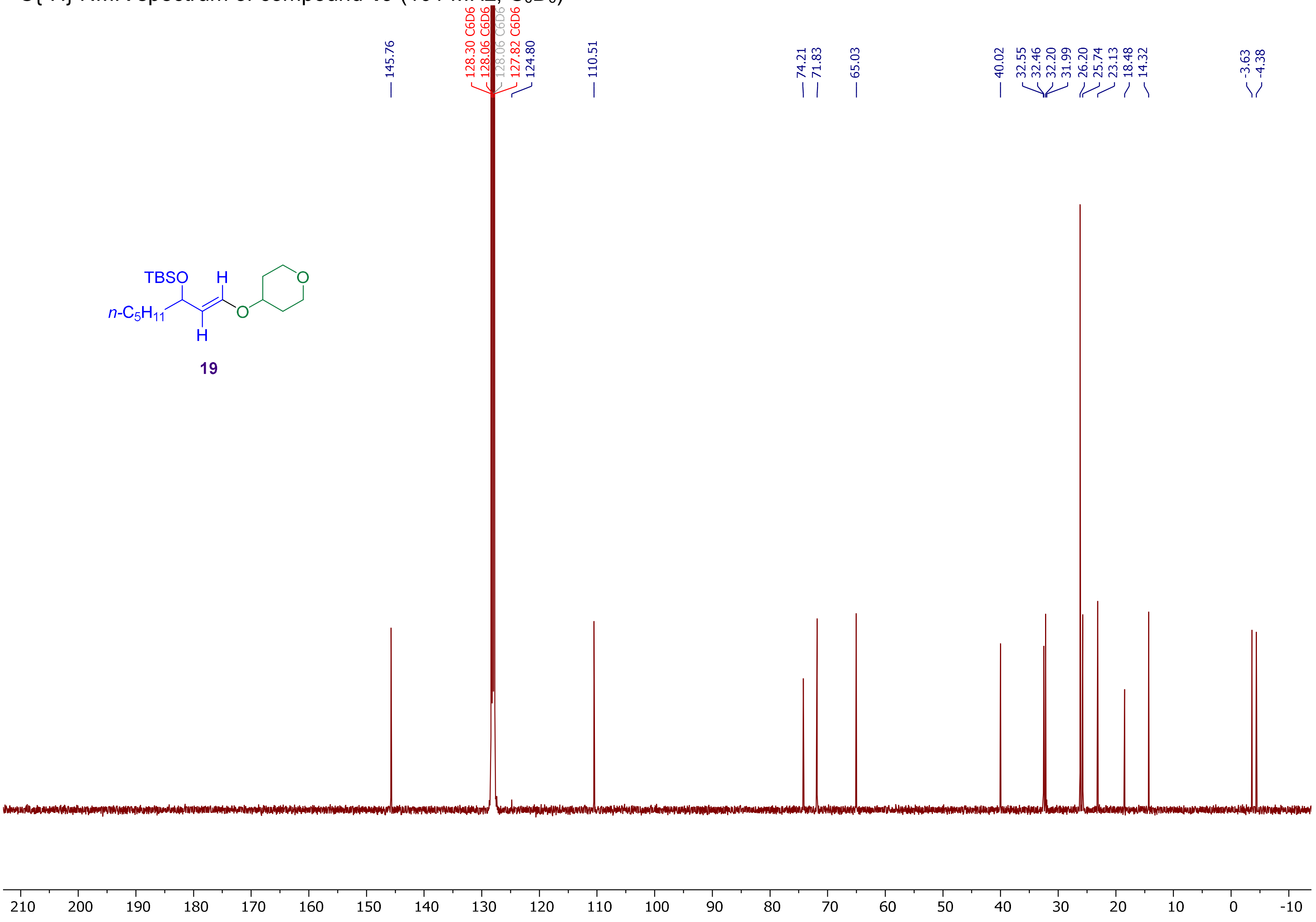

^{1}H NMR spectrum of compound **20** (600 MHz, C_6D_6)

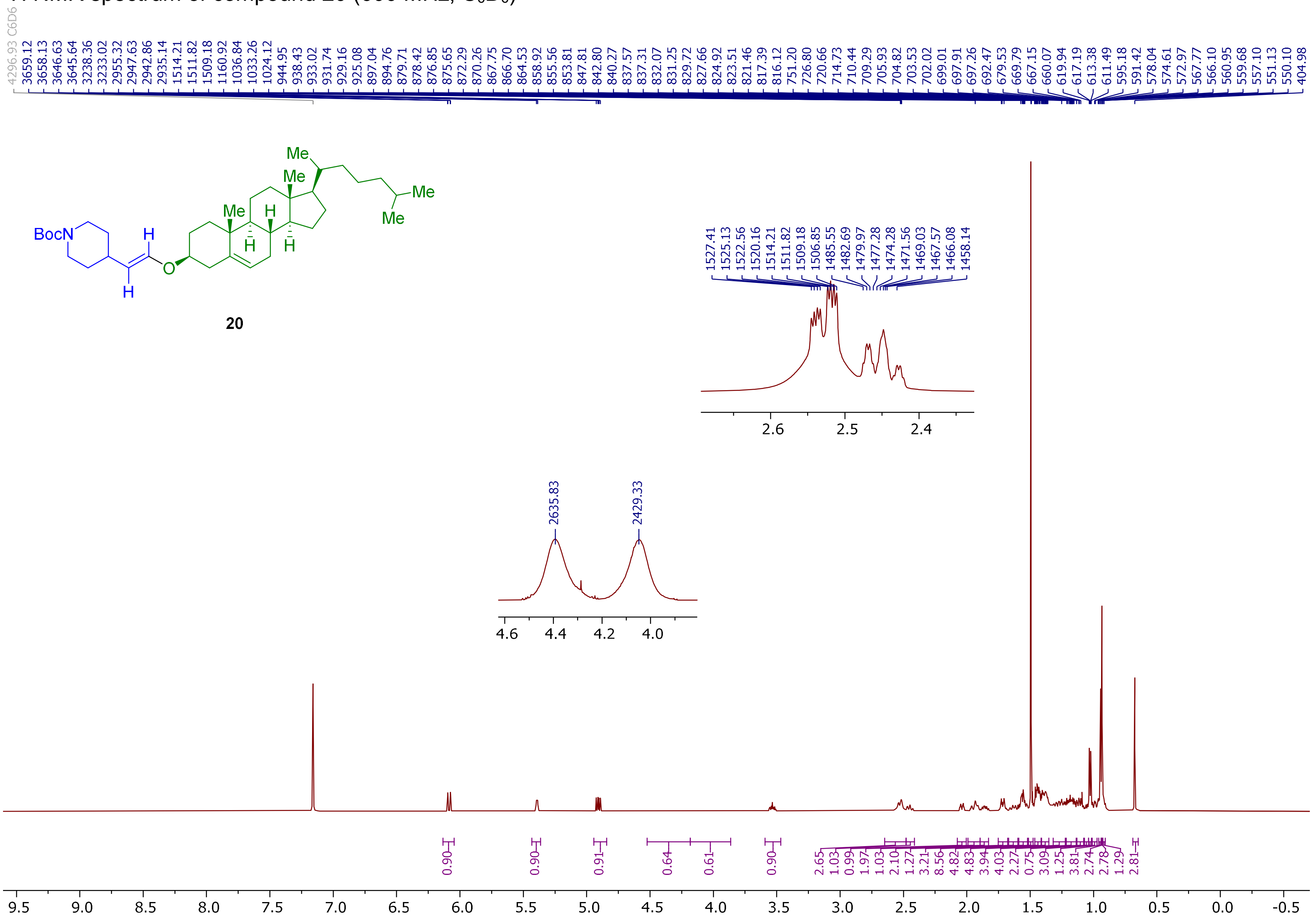

Expansion: ^{1}H NMR spectrum of alkyl region of compound **20** (600 MHz, C_6D_6)

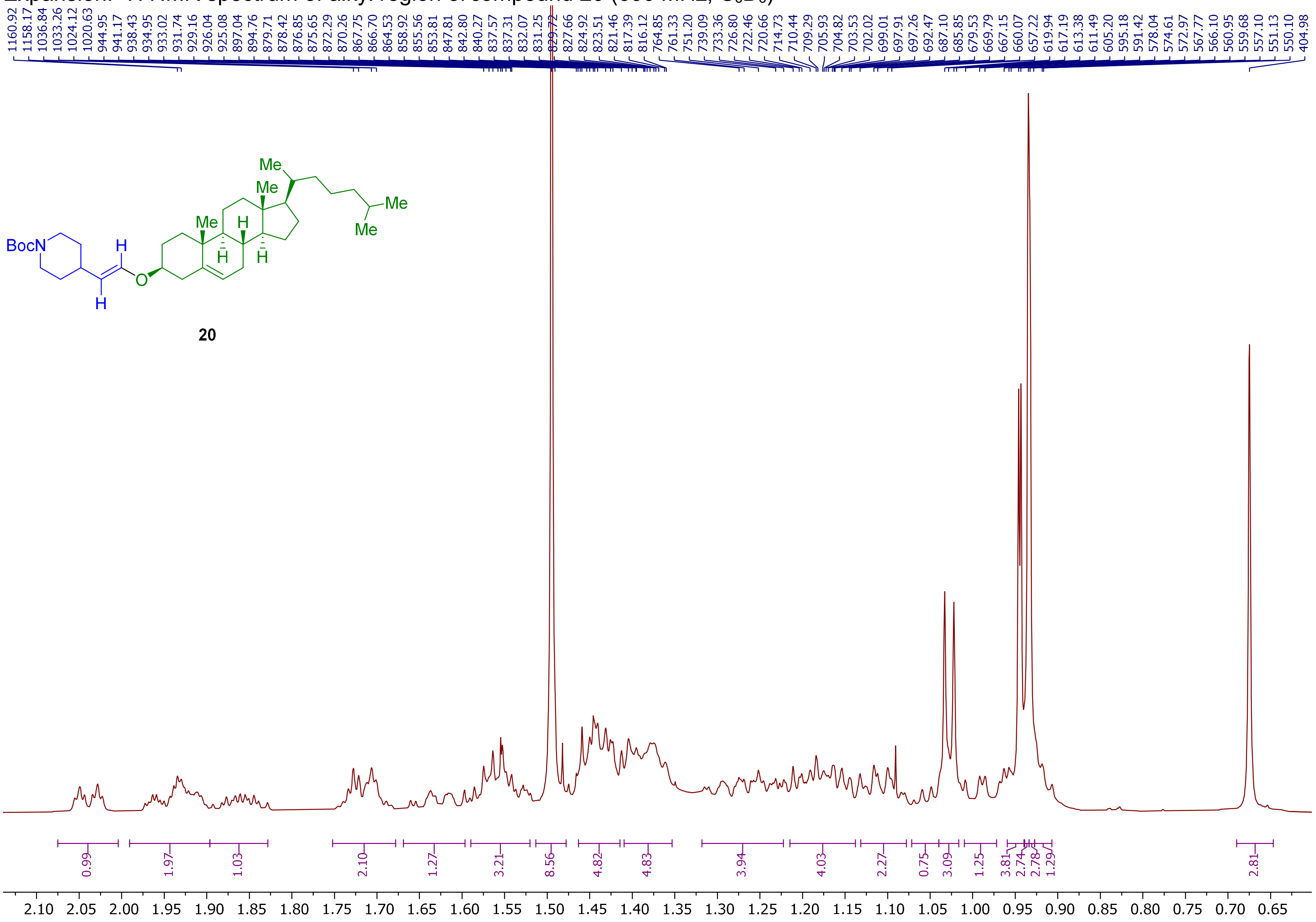

$^{13}C\{^1H\}$ NMR spectrum of compound **20** (151 MHz, C_6D_6)

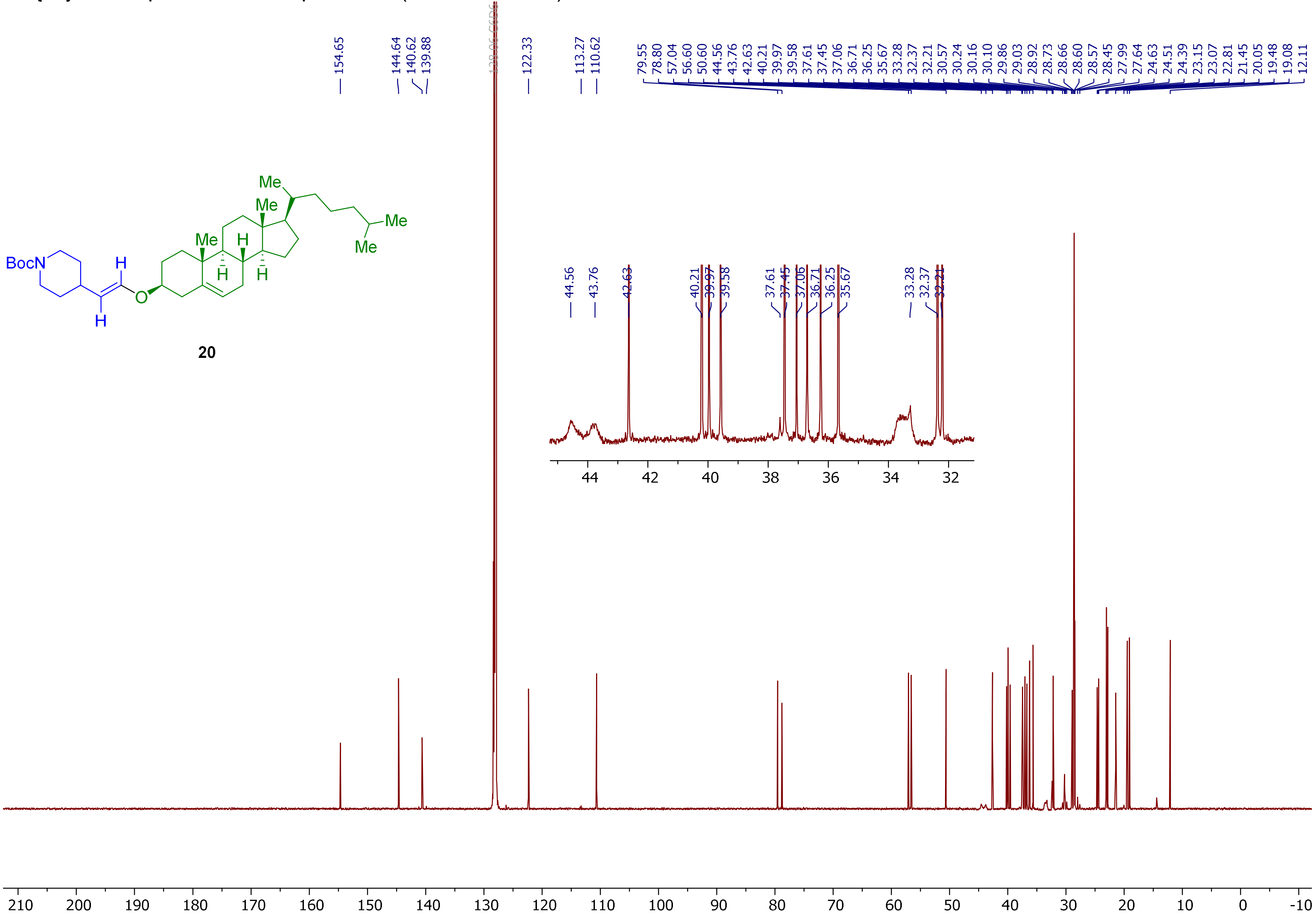

HSQC spectrum of compound **20** (600 MHz, C_6D_6)

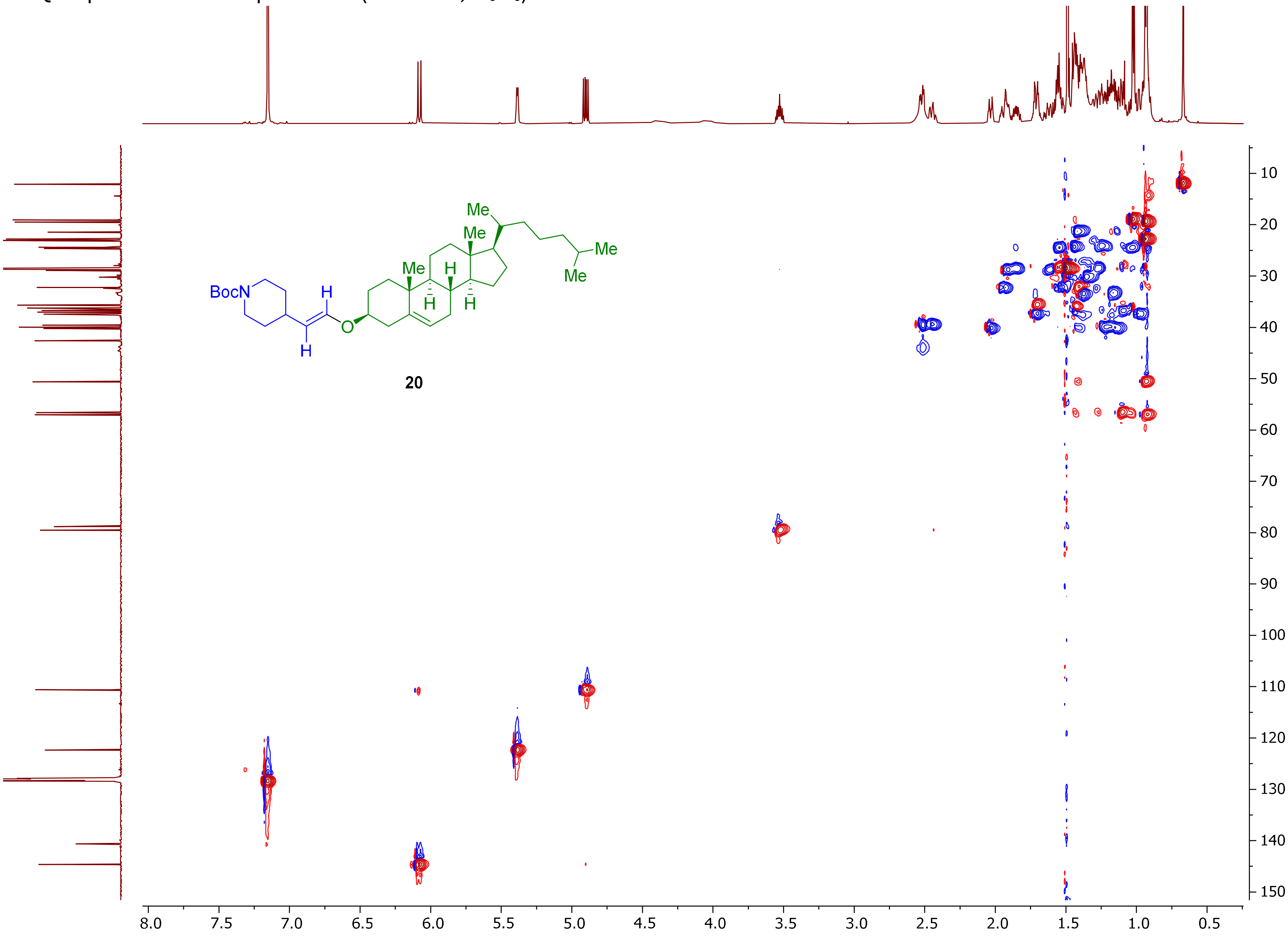

Expansion: HSQC spectrum of compound **20** (600 MHz, C_6D_6)

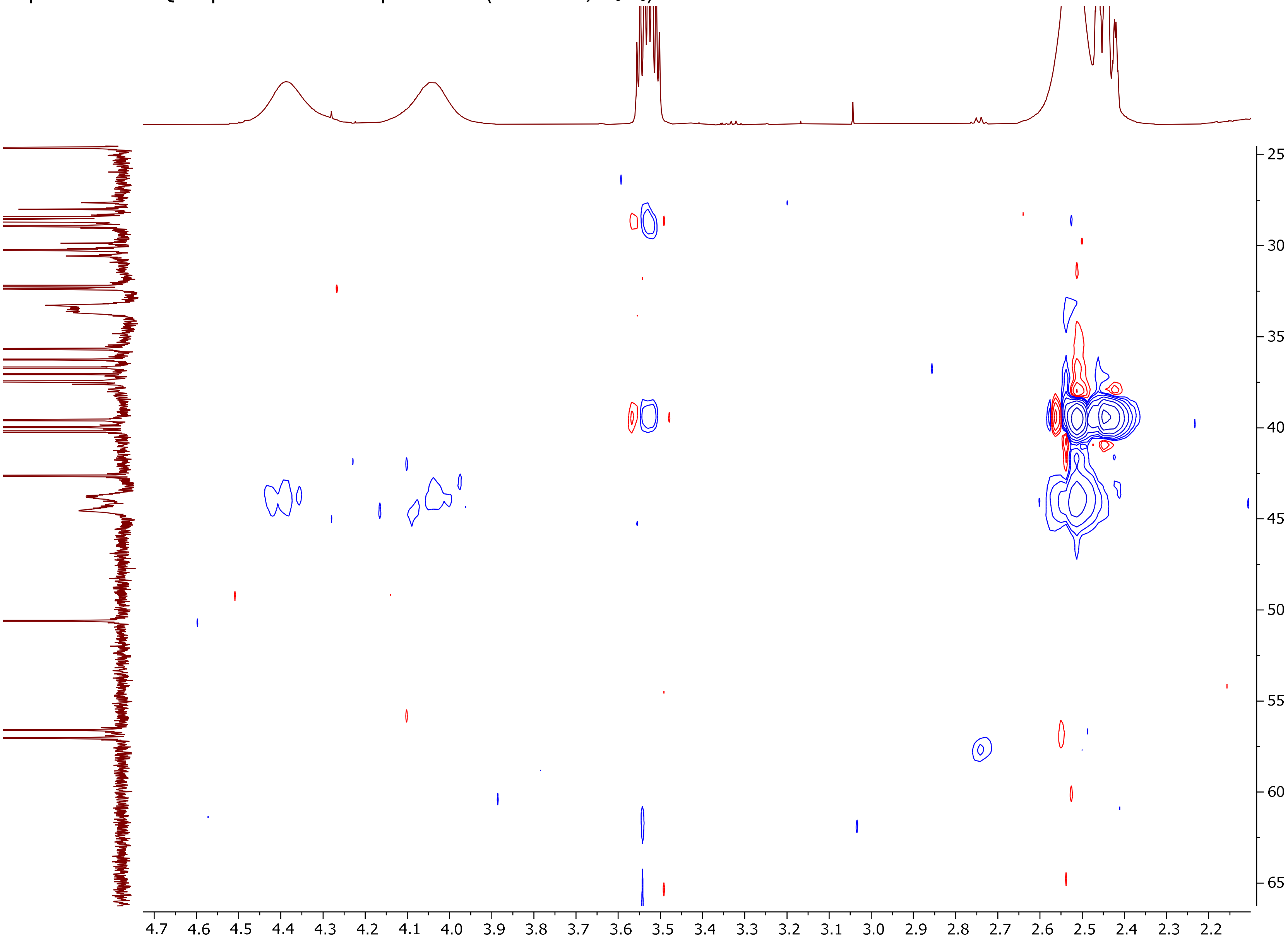

Expansion: HSQC spectrum of compound **20** (600 MHz, C_6D_6)

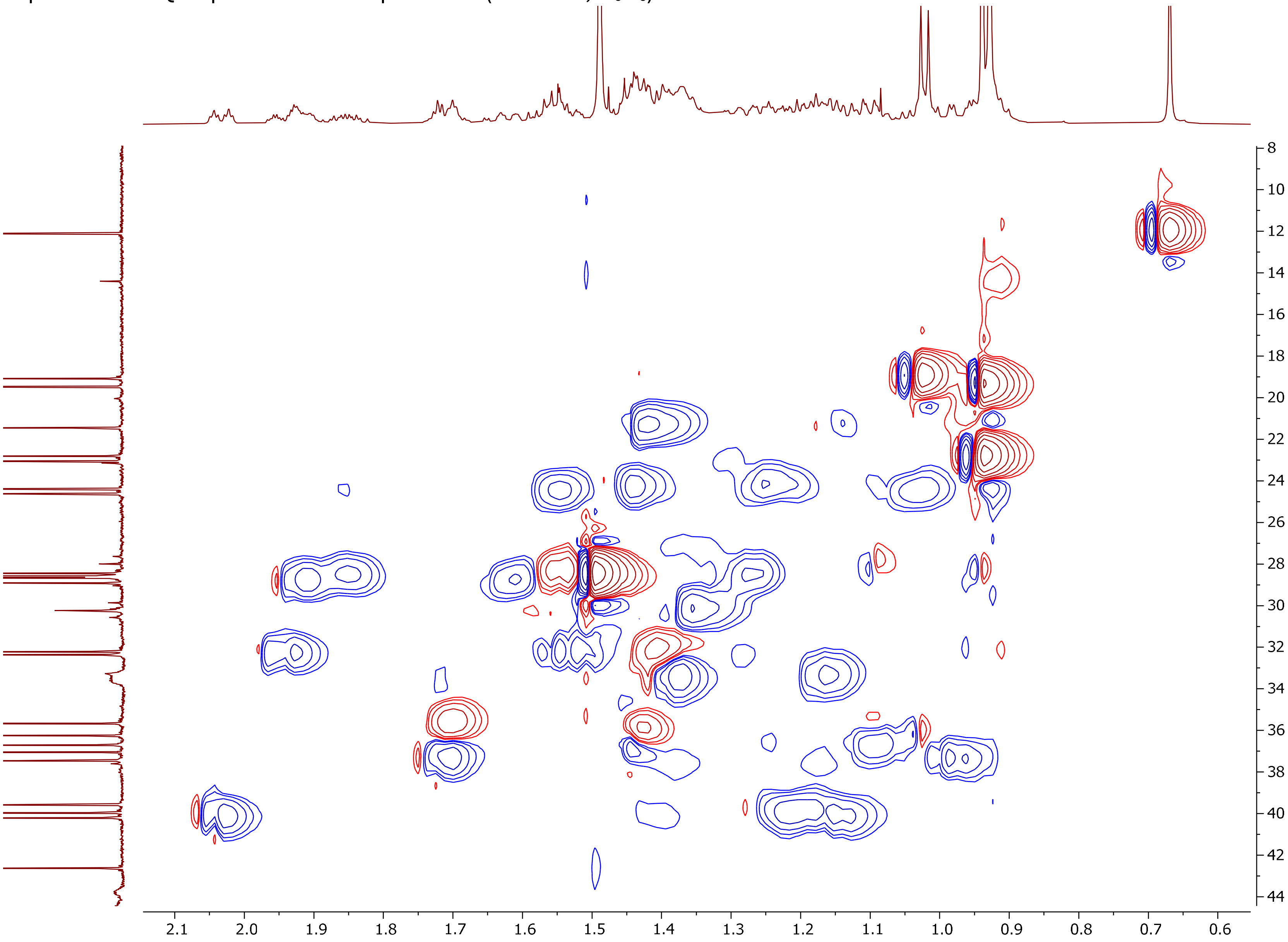

COSY spectrum of compound **20** (600 MHz, C_6D_6)

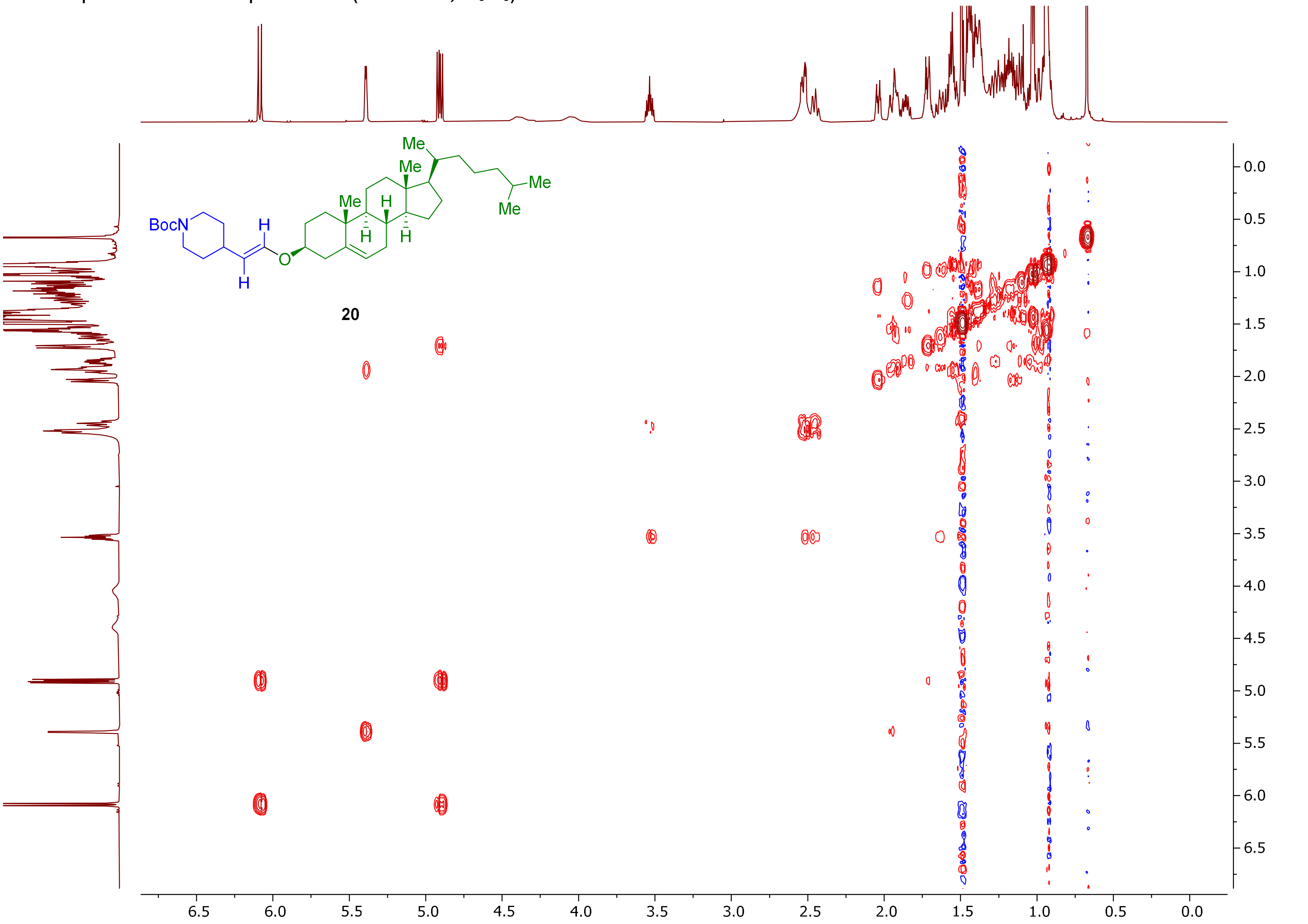

^{1}H NMR spectrum of compound **21** (400 MHz, $CDCl_3$)

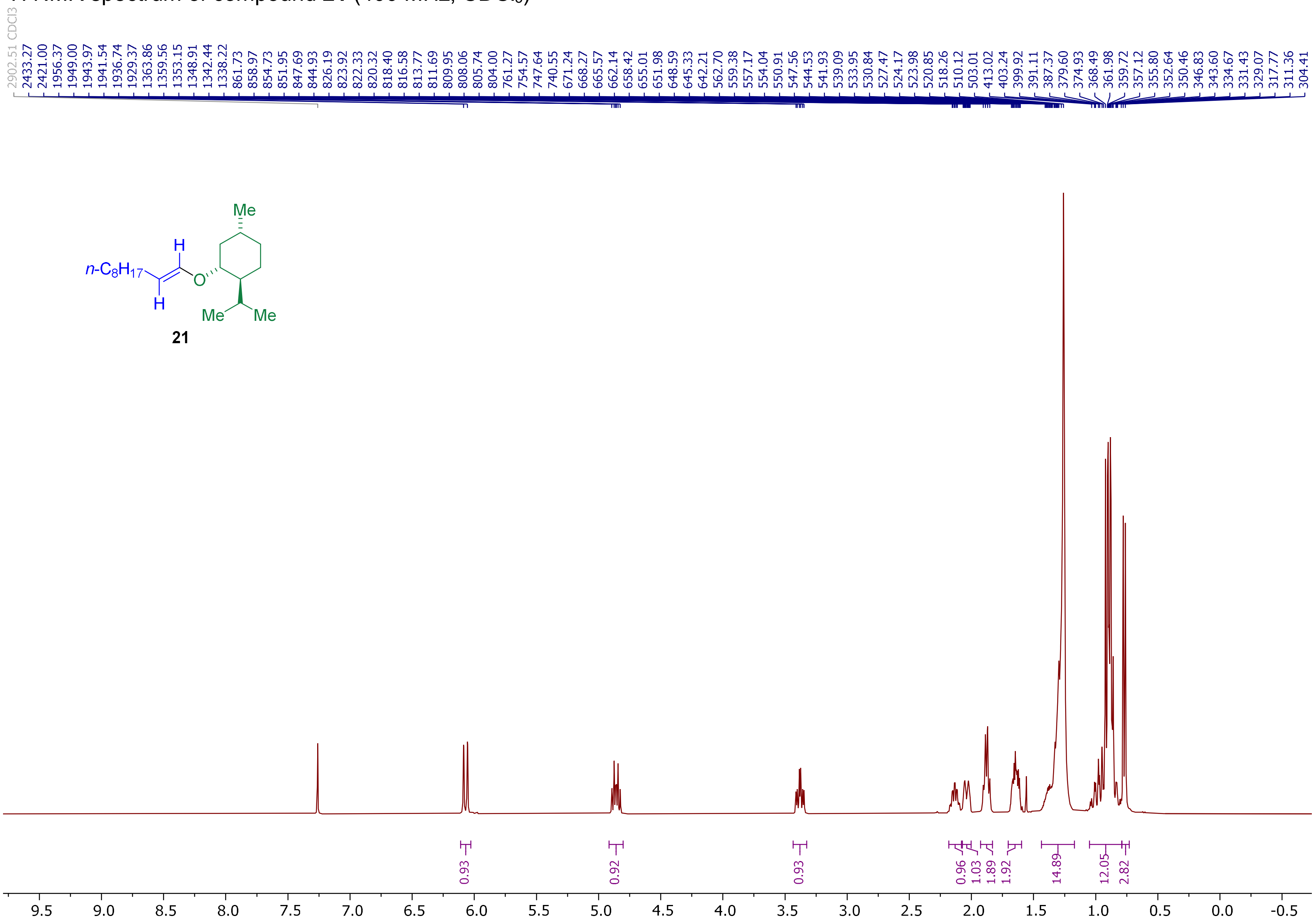

21

^1H NMR spectrum of compound **21** (400 MHz, C_6D_6)

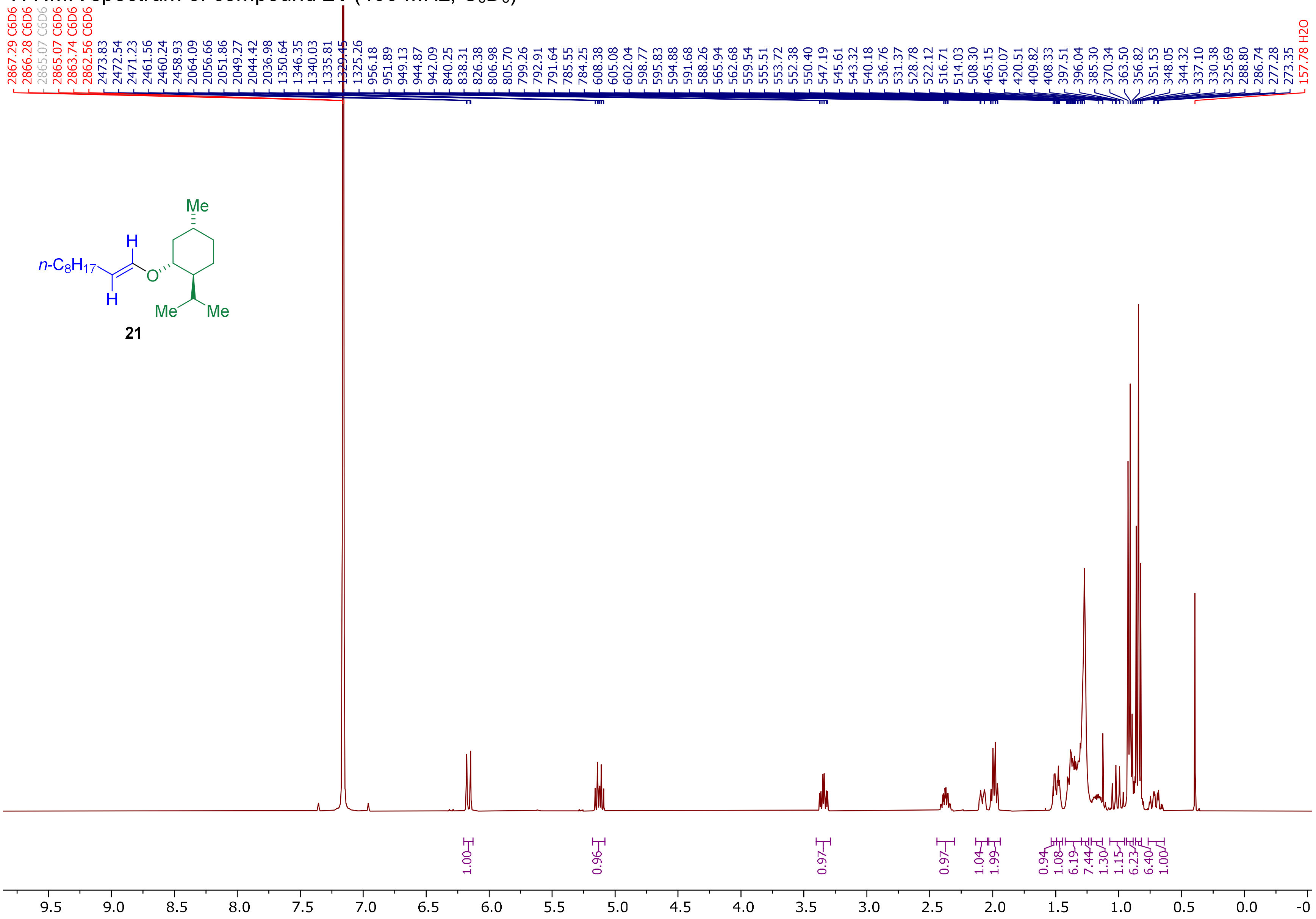

21

Expansion: ^{1}H NMR spectrum of compound **21** (400 MHz, C_6D_6)

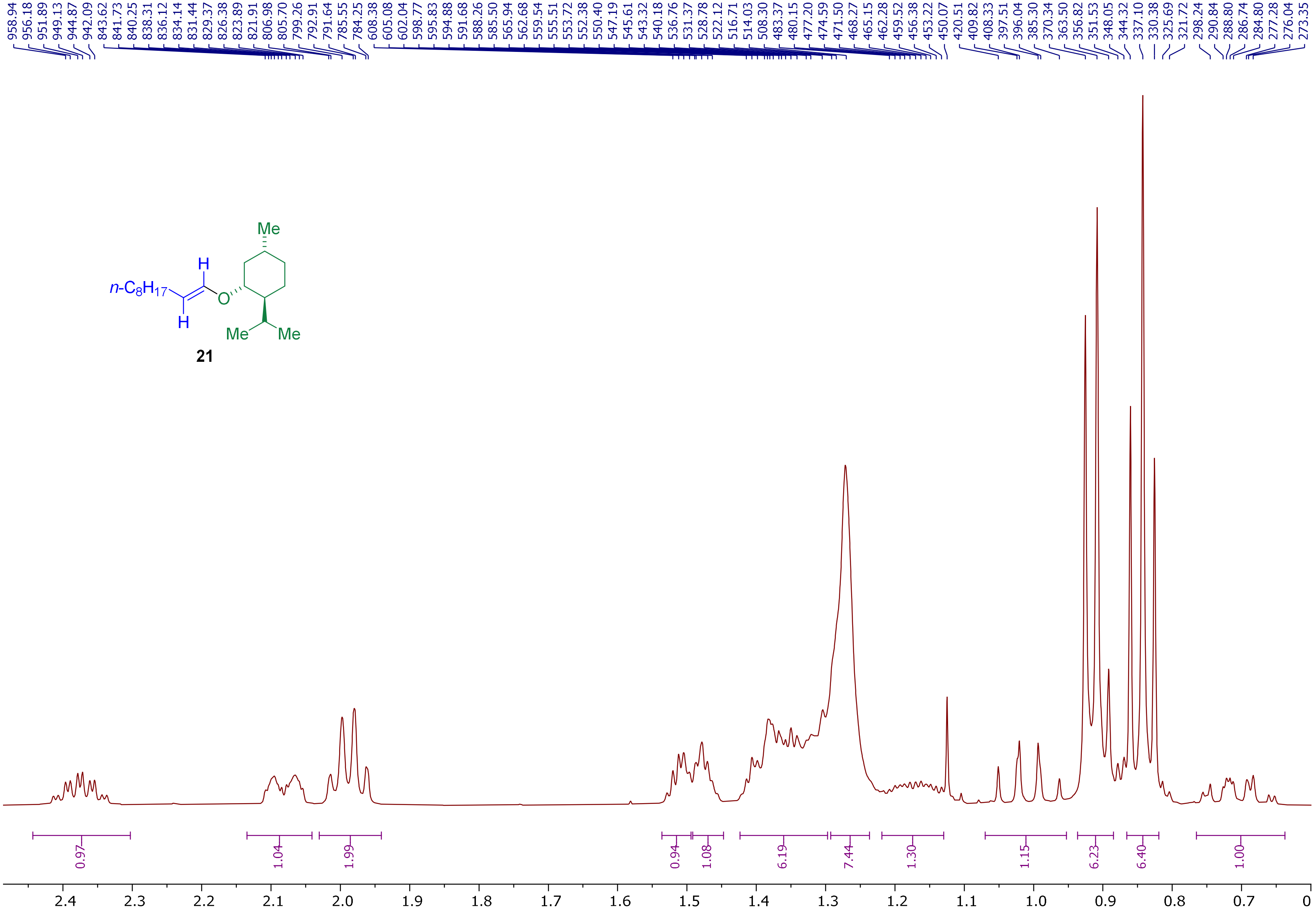

^{1}H NMR spectrum of compound **22** (400 MHz, C_6D_6)

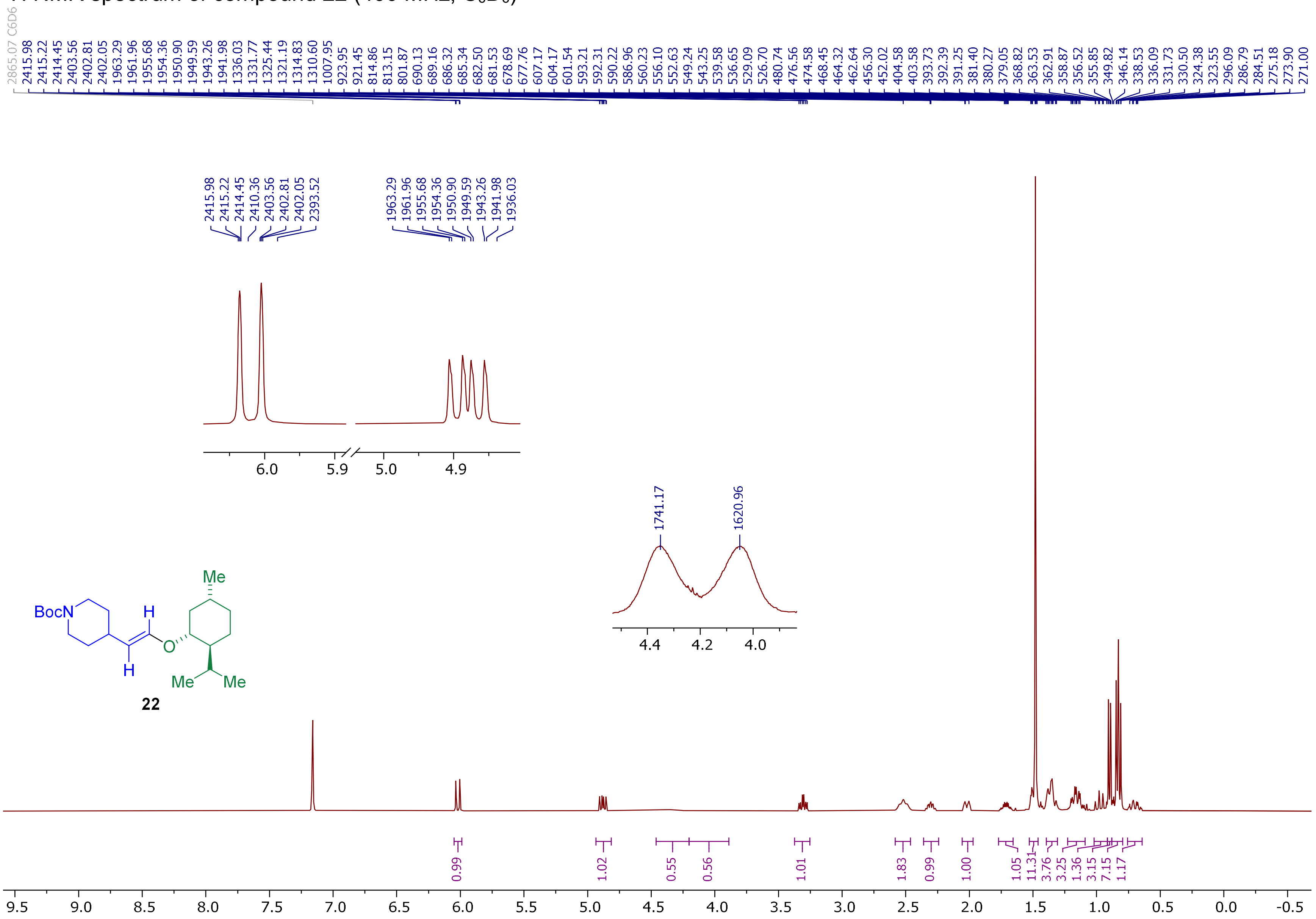

Expansion: ^{1}H NMR spectrum of compound **22** (400 MHz, C_6D_6)

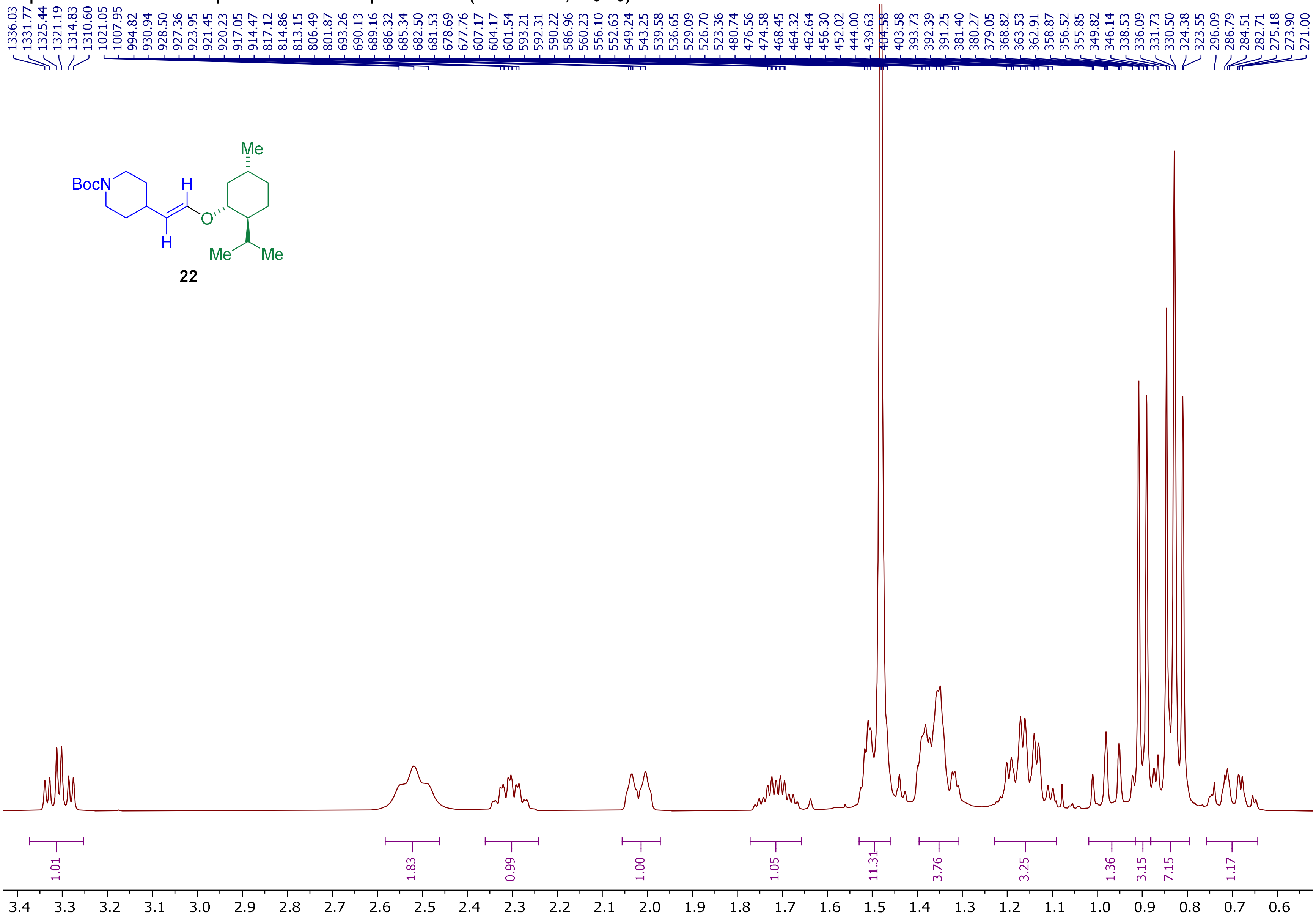

$^{13}C\{^1H\}$ NMR spectrum of compound **22** (101 MHz, C_6D_6)

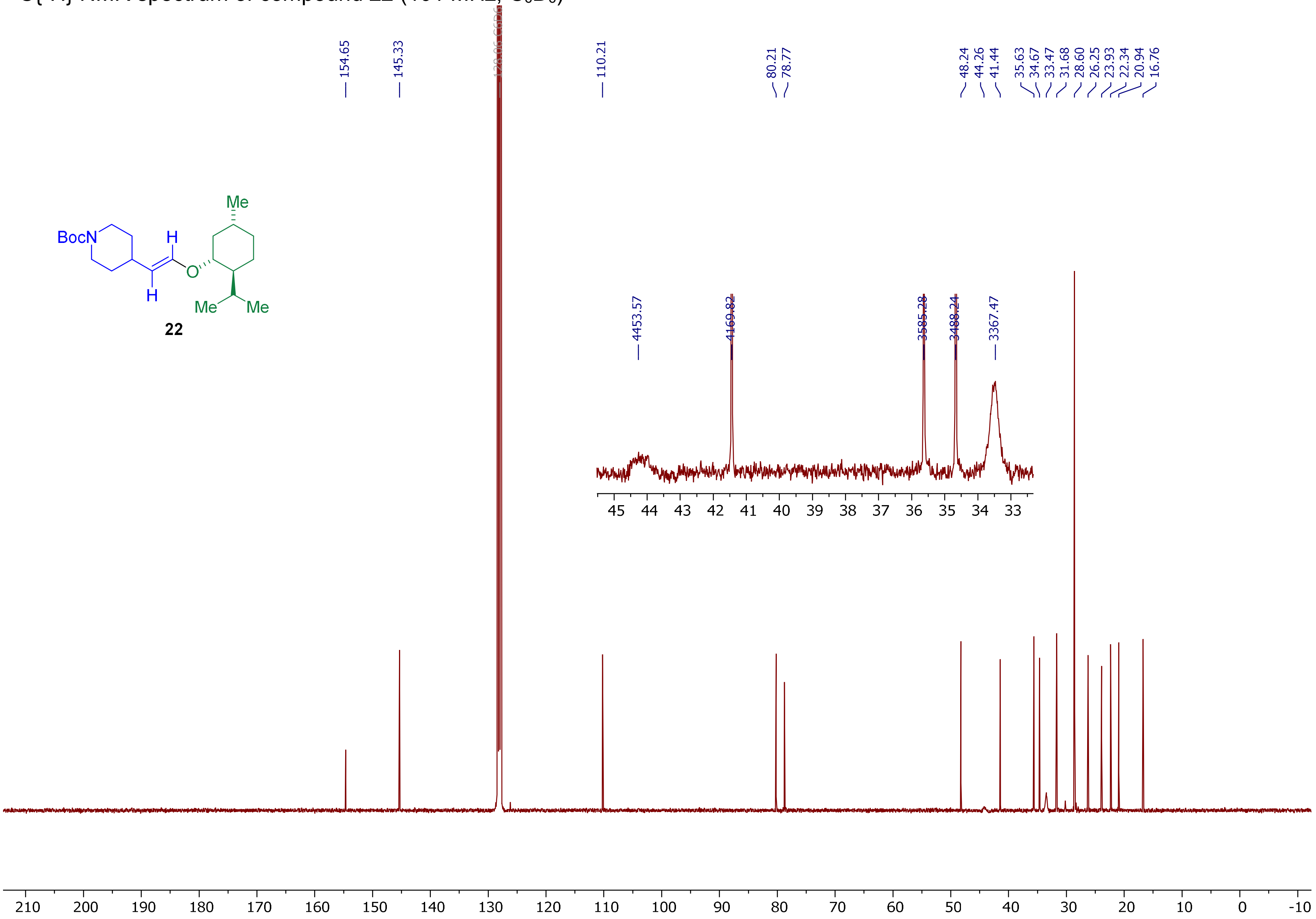

COSY spectrum of compound **22** (400 MHz, C_6D_6)

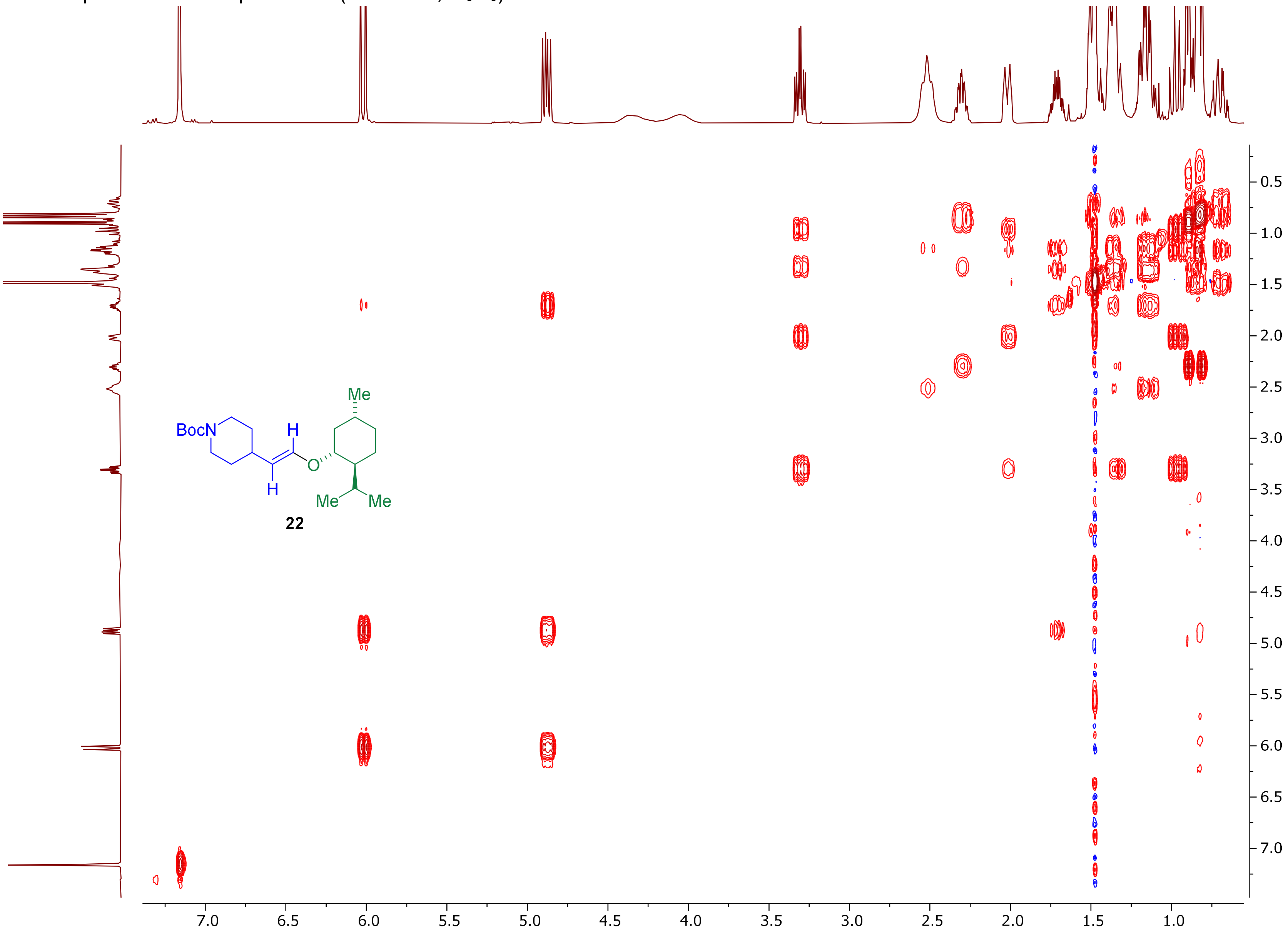

Expansion: COSY spectrum of compound **22** (400 MHz, C_6D_6)

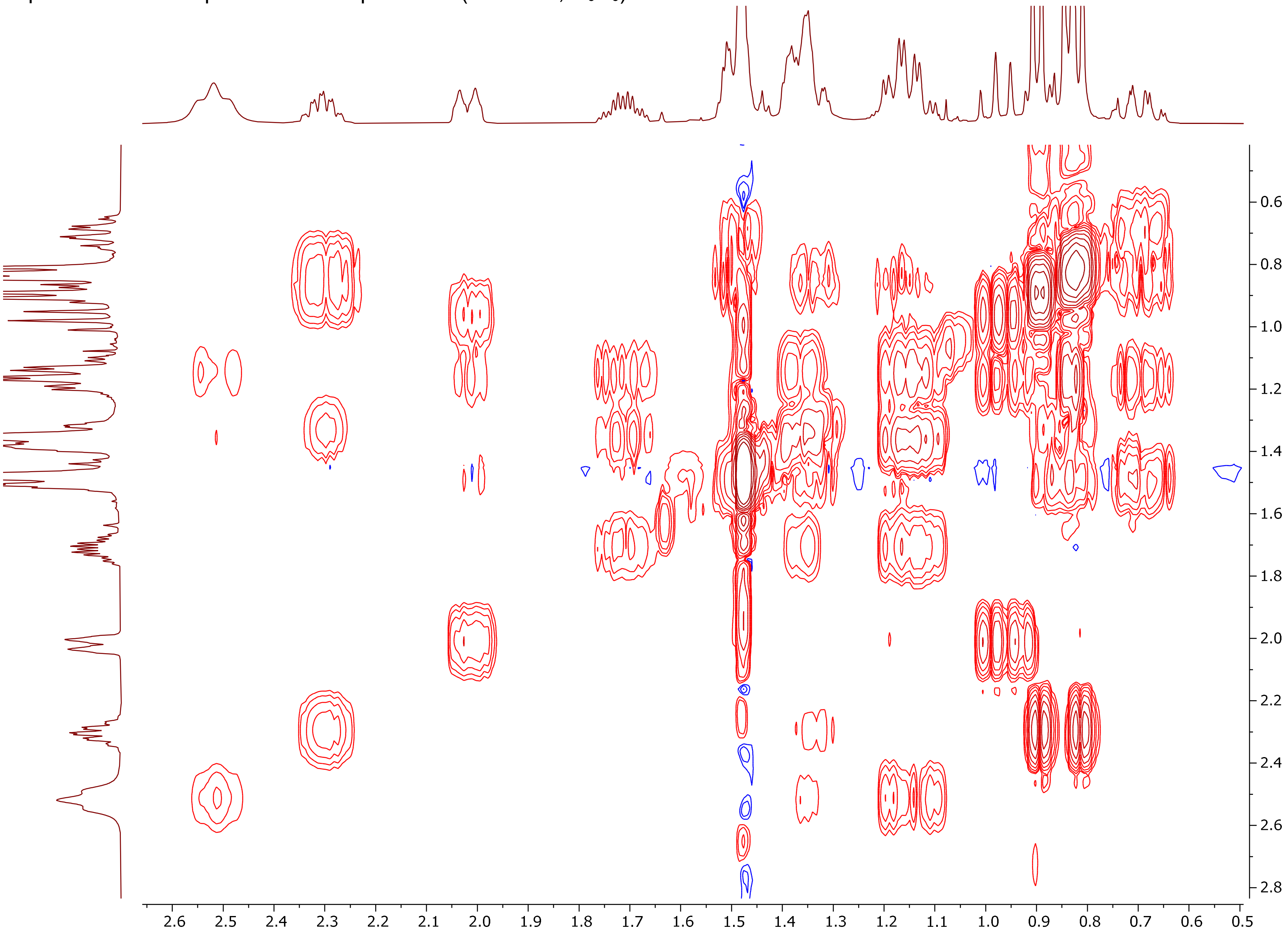

HSQC spectrum of compound **22** (400 MHz, C_6D_6)

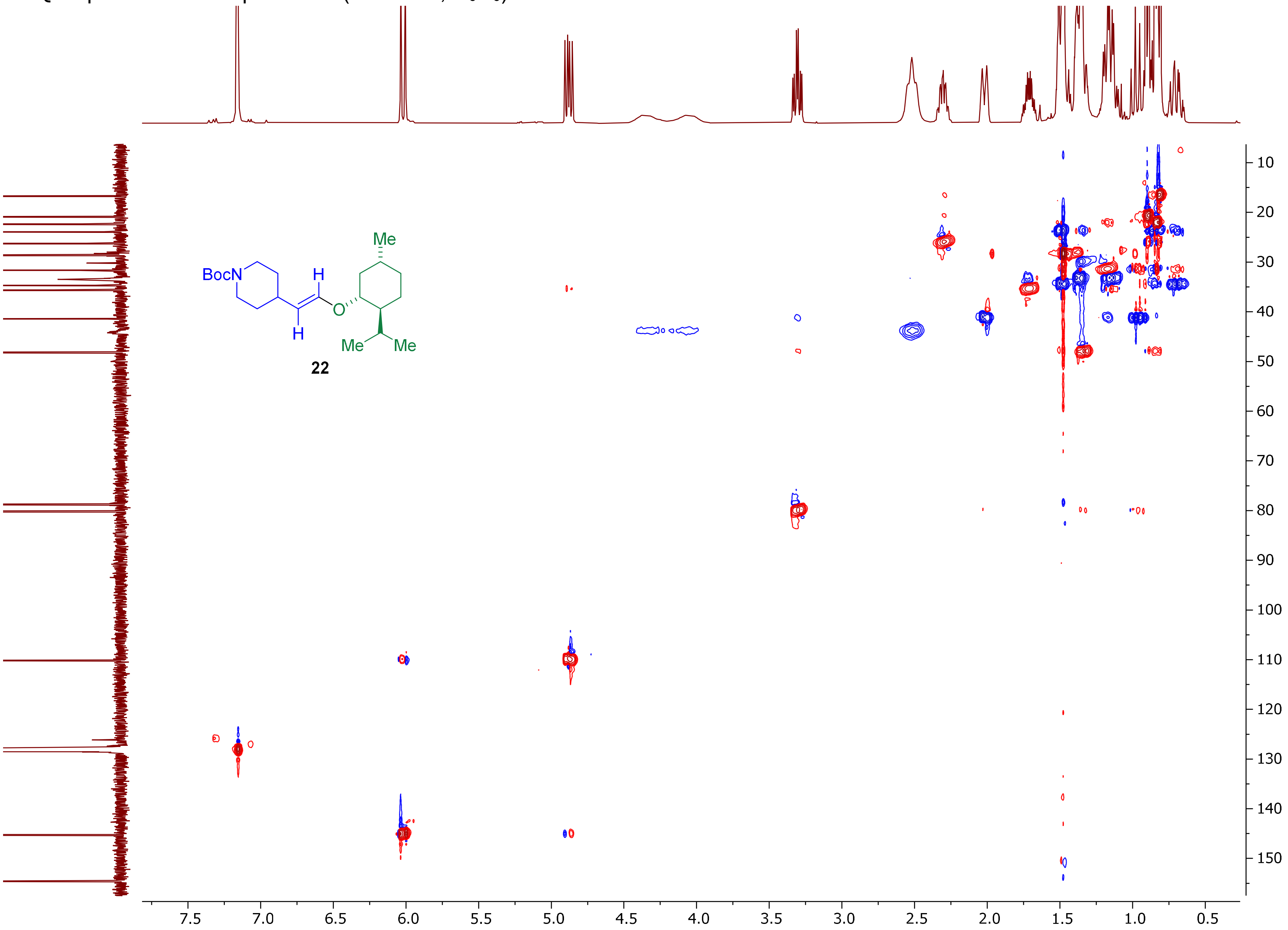

Expansion: HSQC spectrum of compound **22** (400 MHz, C_6D_6)

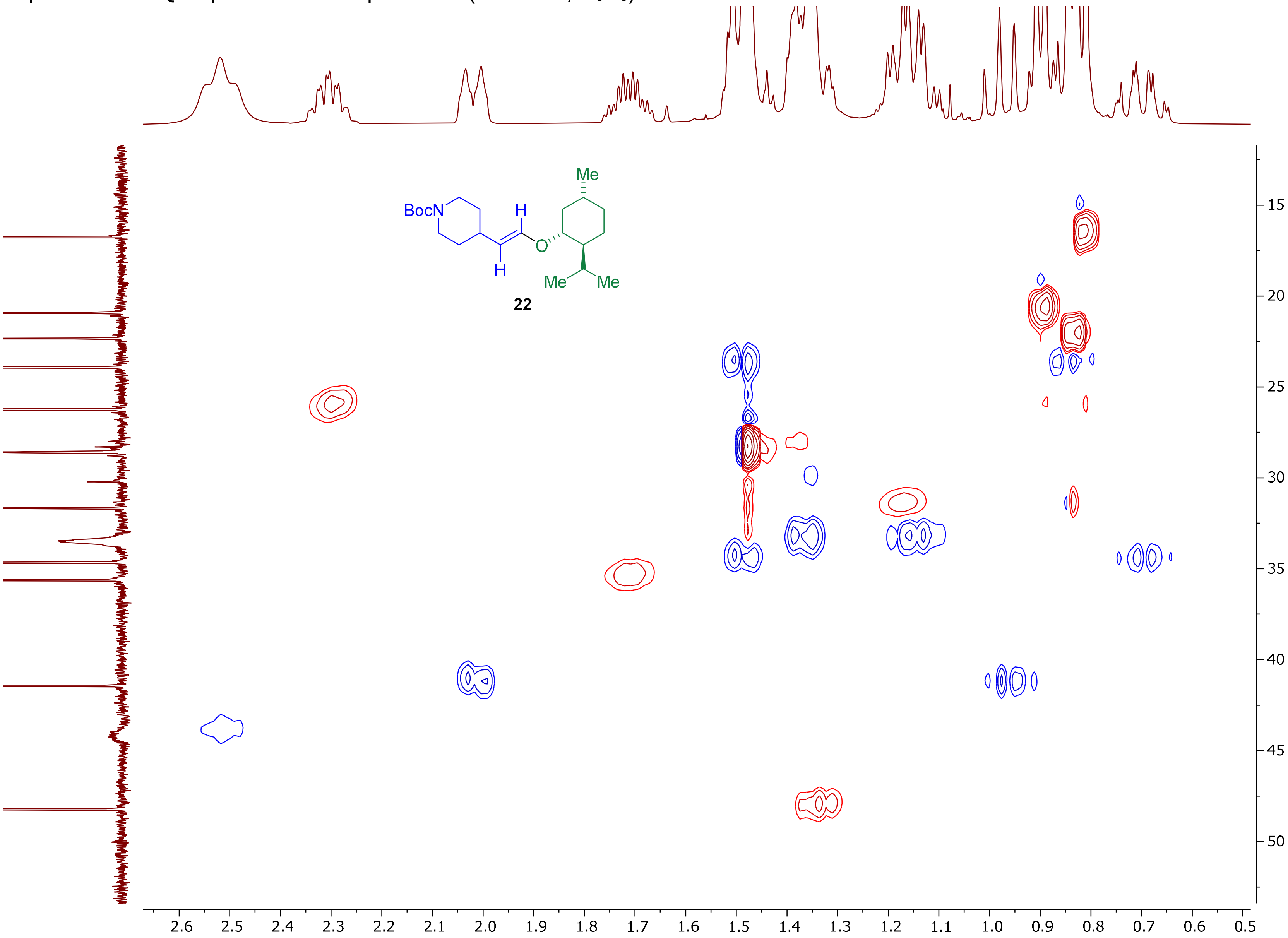

^{1}H NMR spectrum of compound **23** (400 MHz, C_6D_6)

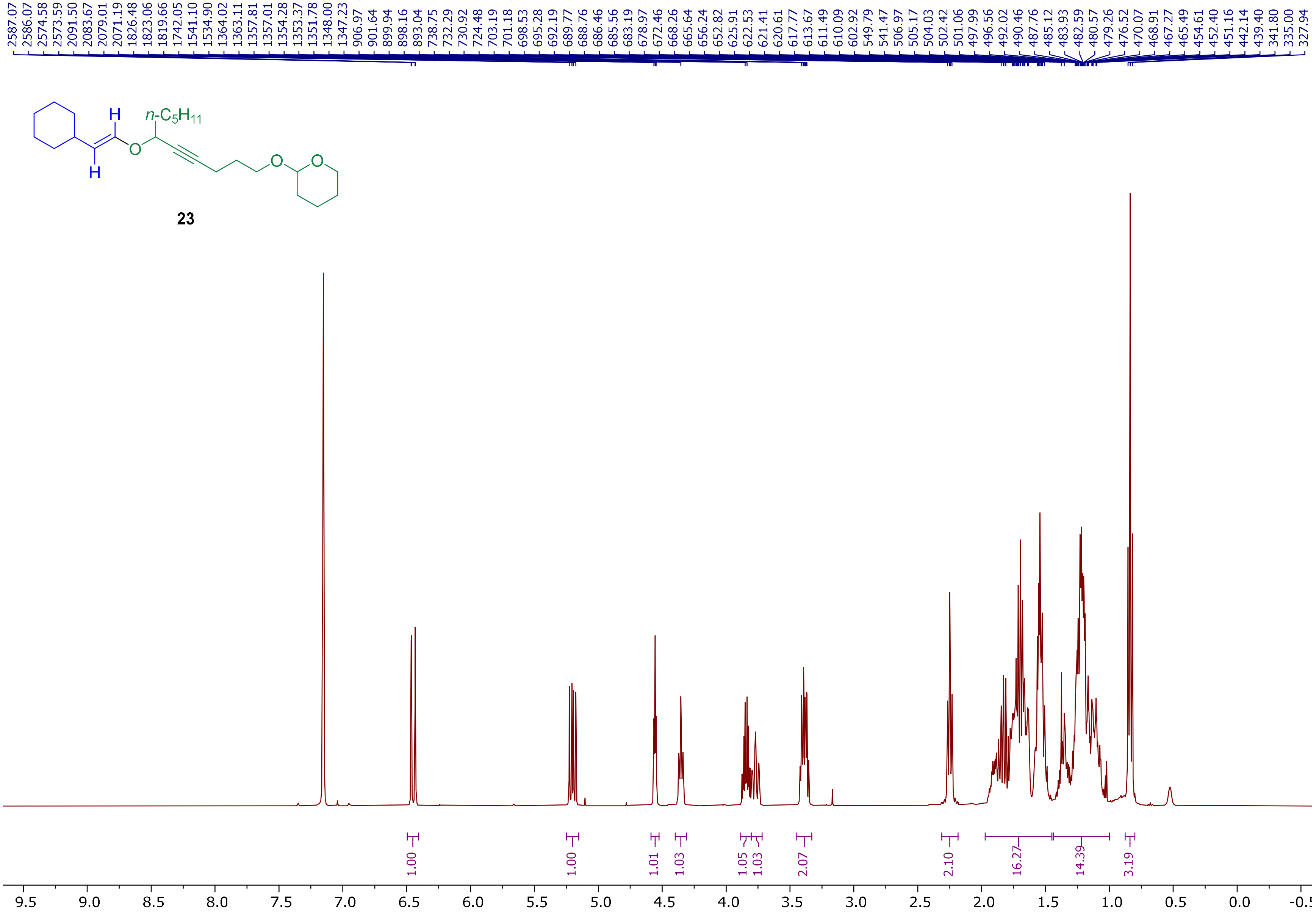

$^{13}C\{^{1}H\}$ NMR spectrum of compound **23** (101 MHz, C_6D_6)

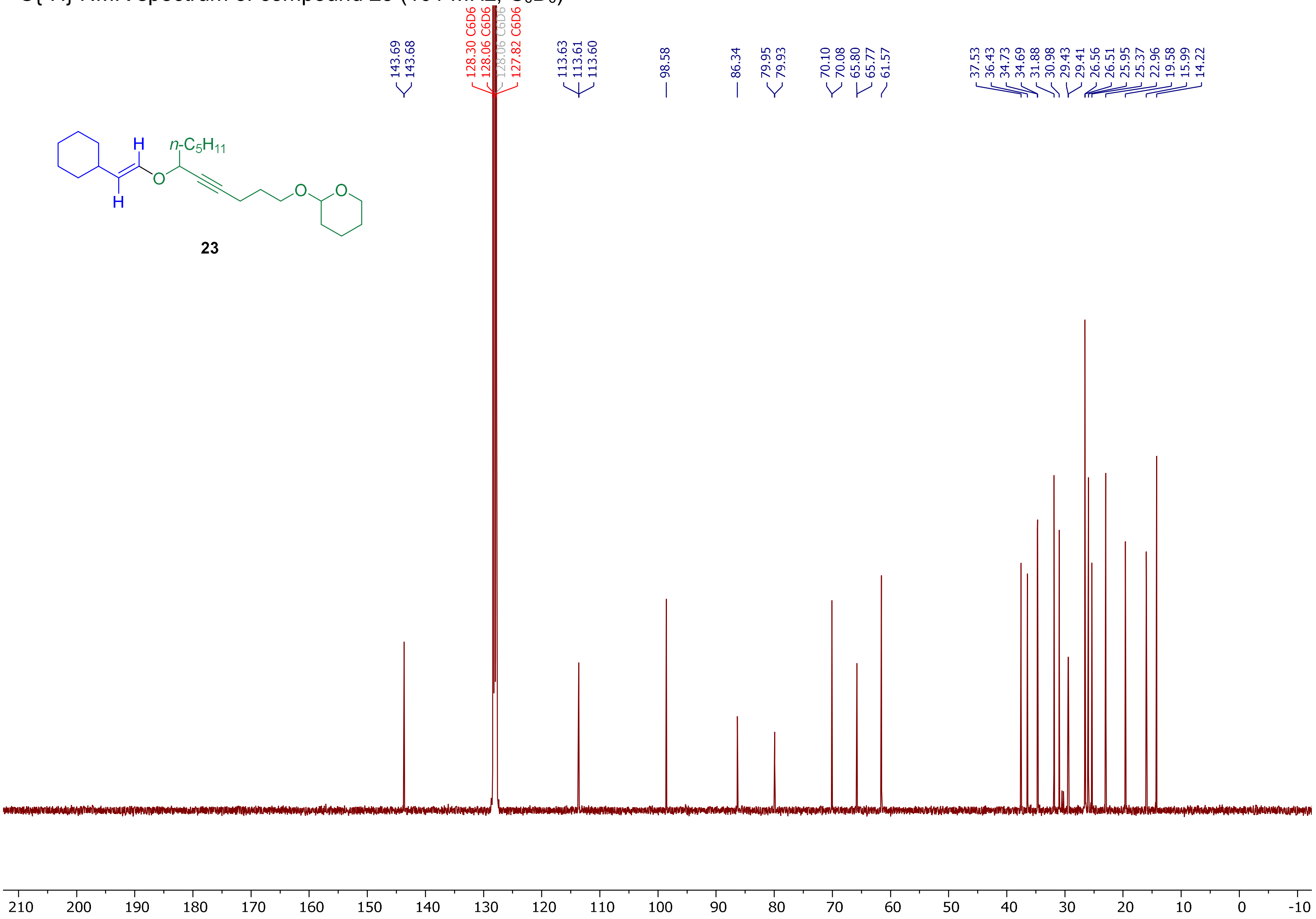

HSQC spectrum of compound **23** (400 MHz, C_6D_6)

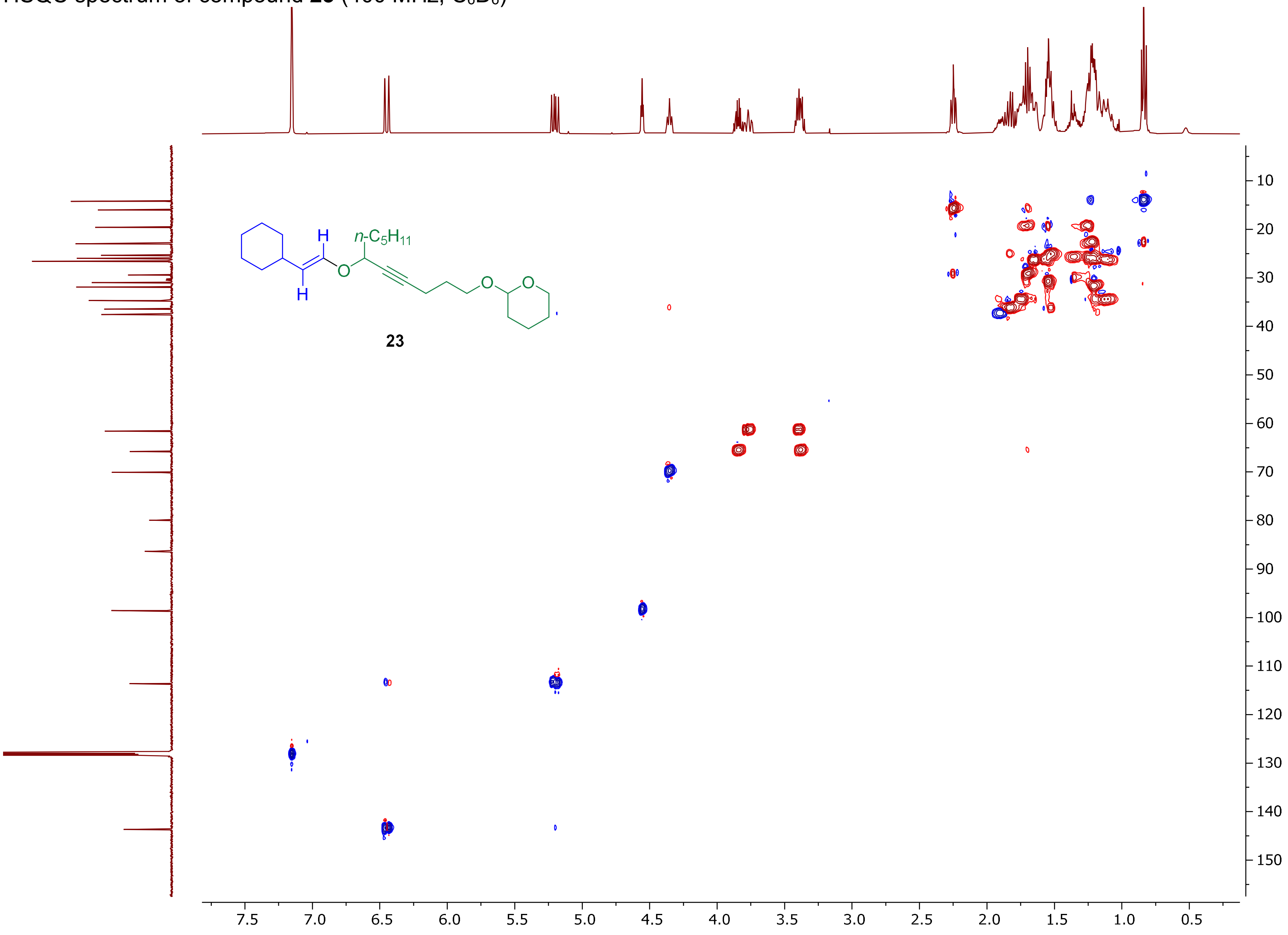

Expansion: HSQC spectrum of compound **23** (400 MHz, C_6D_6)

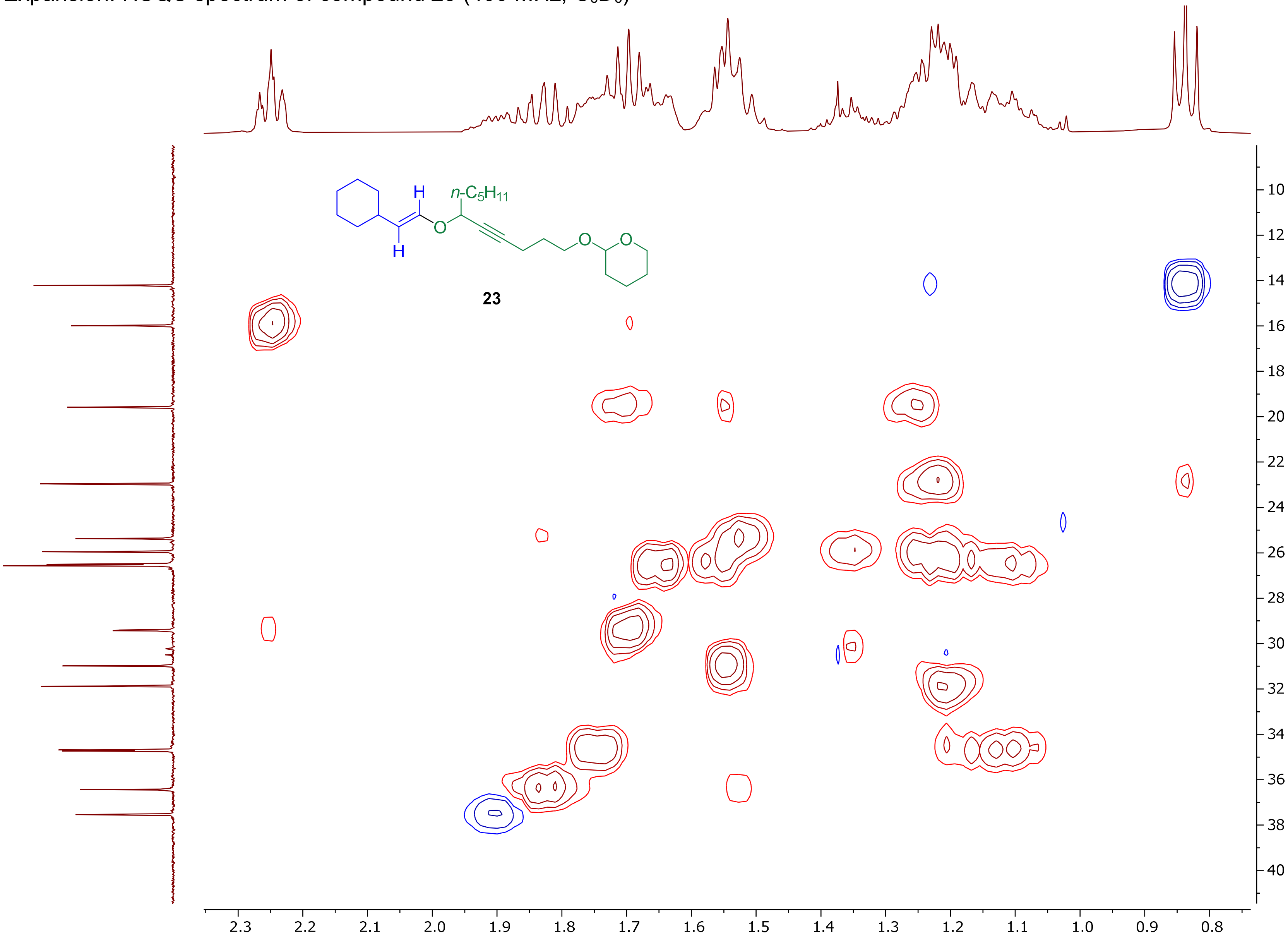

^{1}H NMR spectrum of compound **24** (400 MHz, C_6D_6)

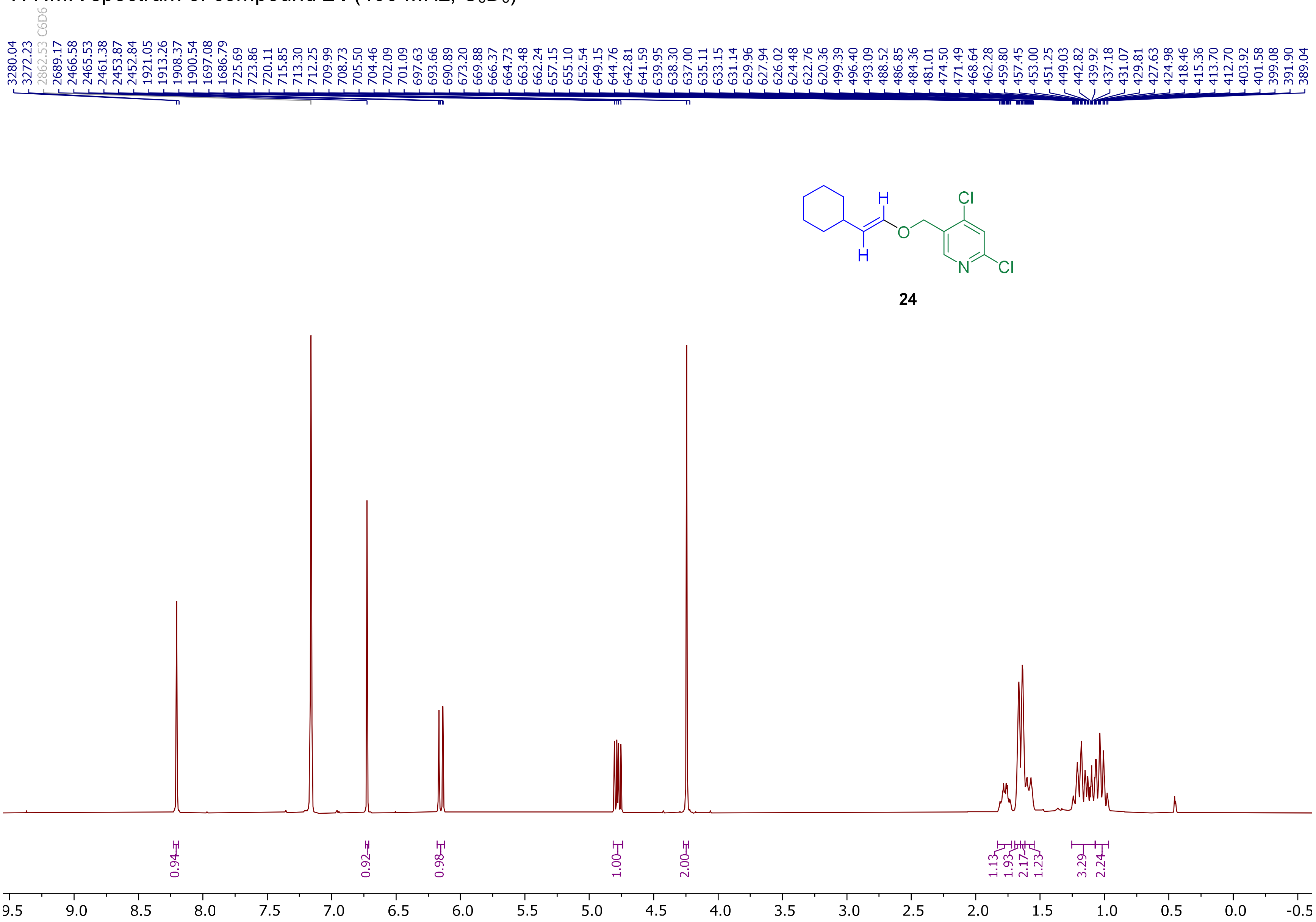

$^{13}C\{^{1}H\}$ NMR spectrum of compound **24** (101 MHz, C_6D_6)

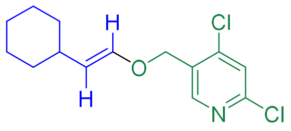

24

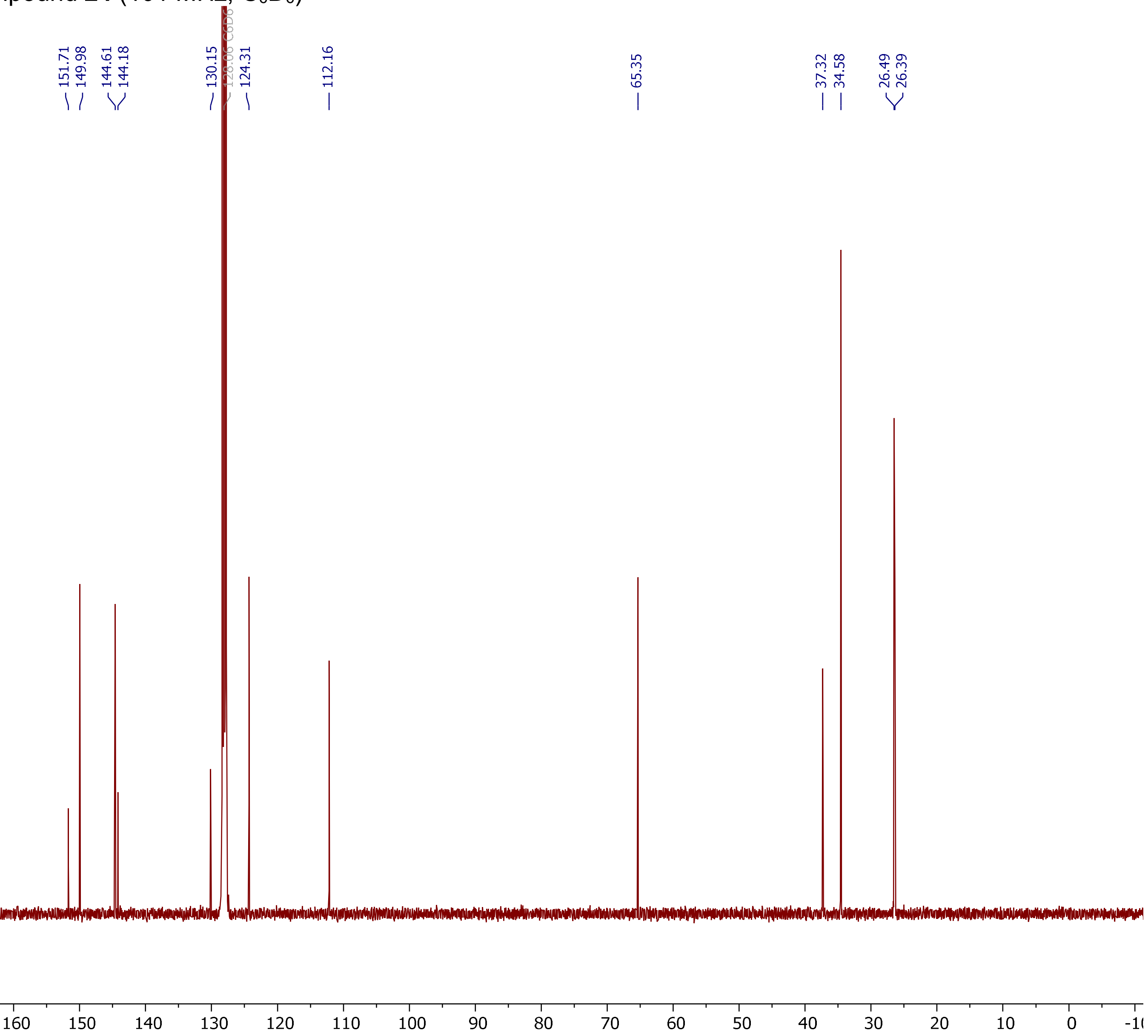

^{1}H NMR spectrum of compound **25** (400 MHz, C_6D_6)

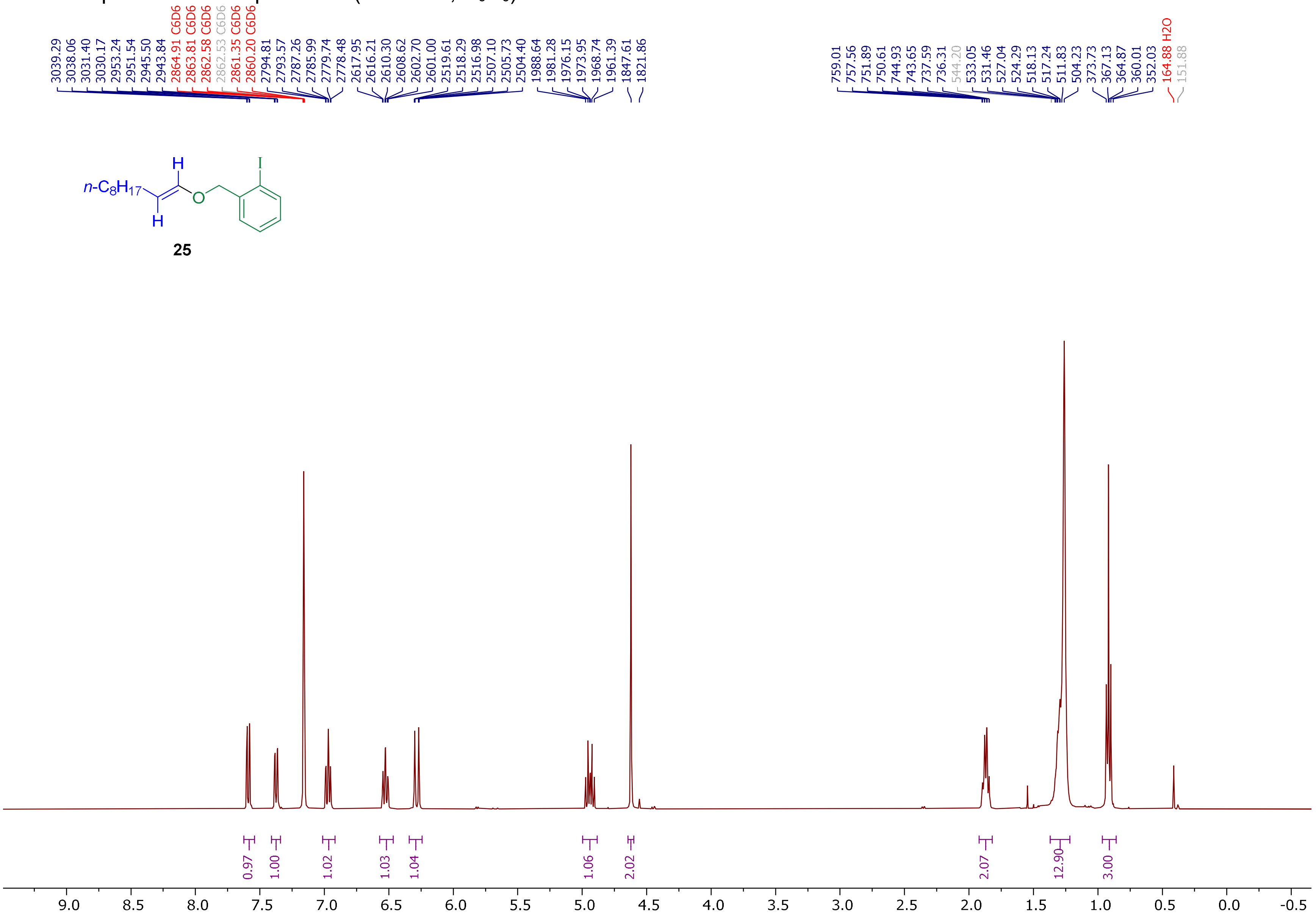

Expansion: ^{1}H NMR spectrum of compound **25** (400 MHz, C_6D_6)

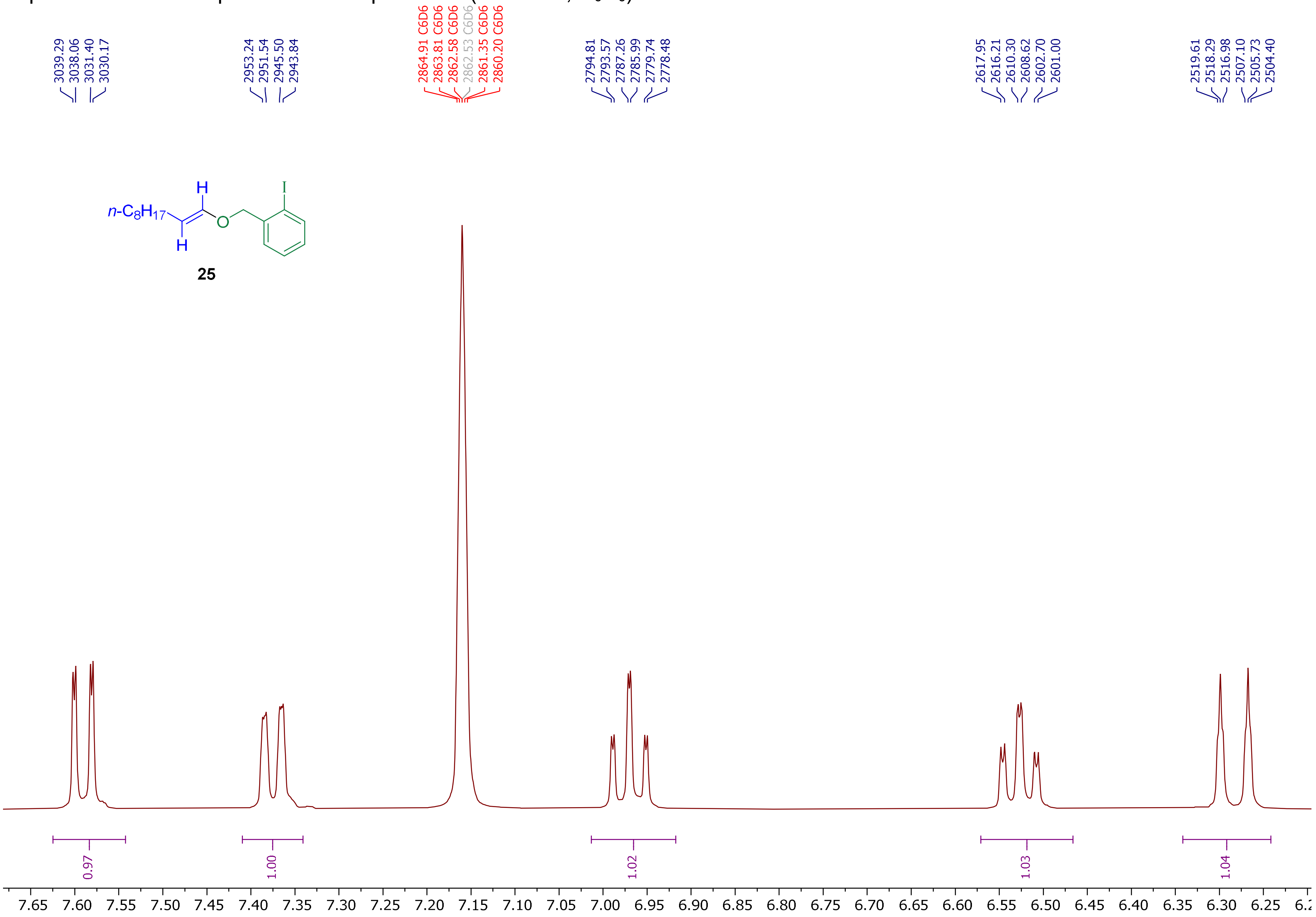

$^{13}C\{^1H\}$ NMR spectrum of compound **25** (101 MHz, C_6D_6)

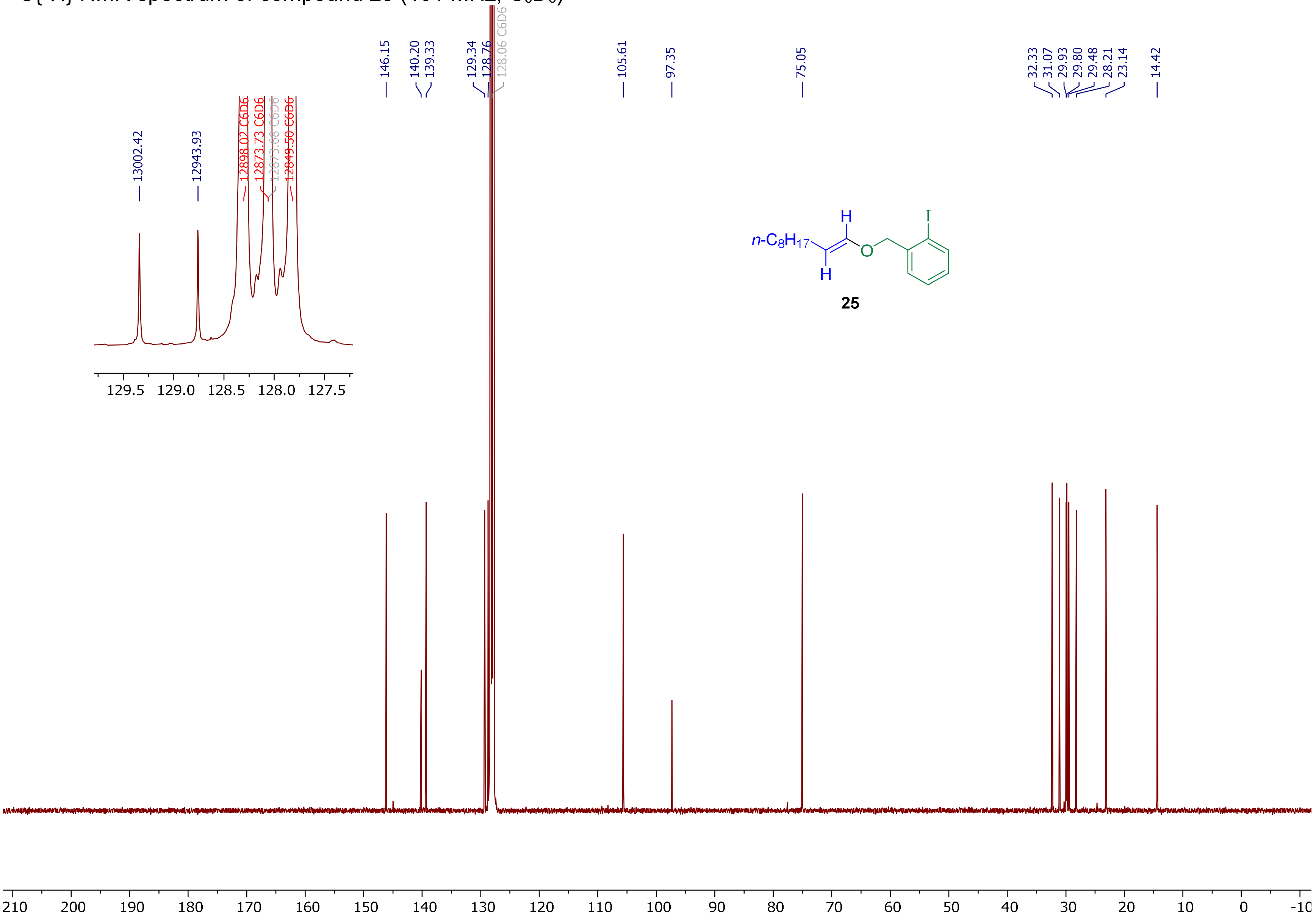

^{1}H NMR spectrum of compound **26** (400 MHz, C_6D_6)

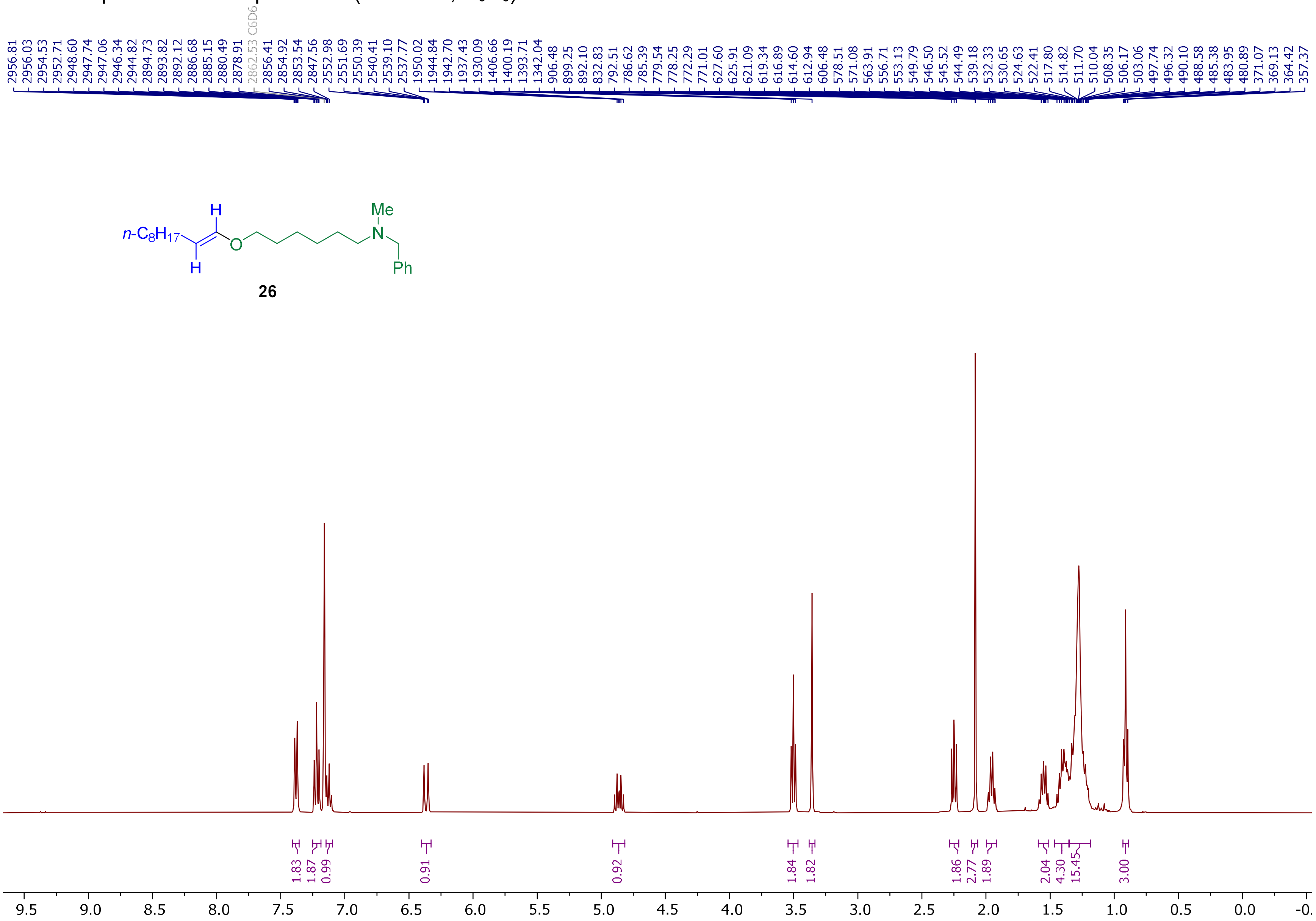

$^{13}C\{^1H\}$ NMR spectrum of compound **26** (101 MHz, C_6D_6)

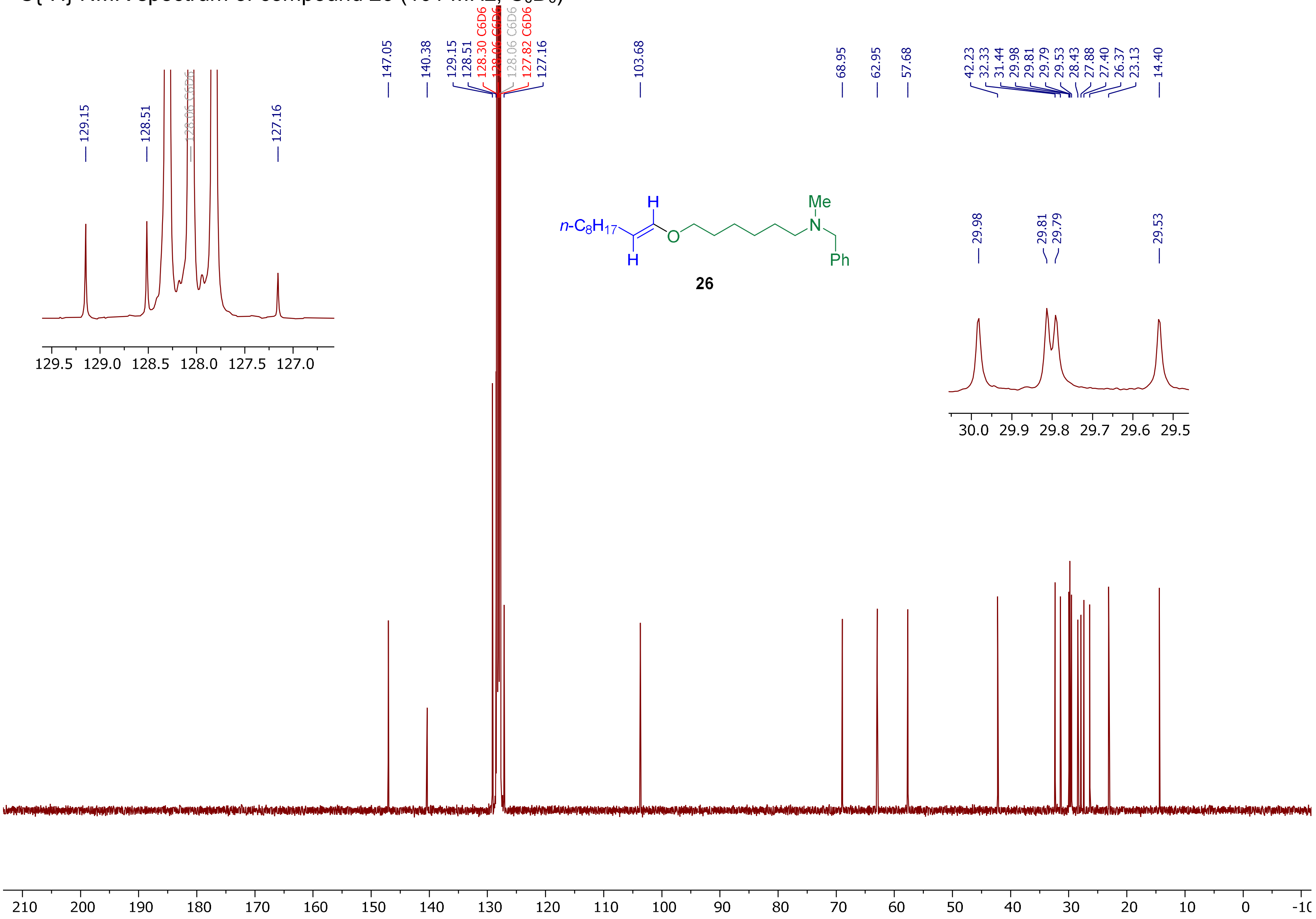

^{1}H NMR spectrum of compound **32** (400 MHz, C_6D_6) – mixture of 0.35 : 1.0 tolyl Bpin **31** /tolyl ether **32**, containing traces of hexanes from column chromatography

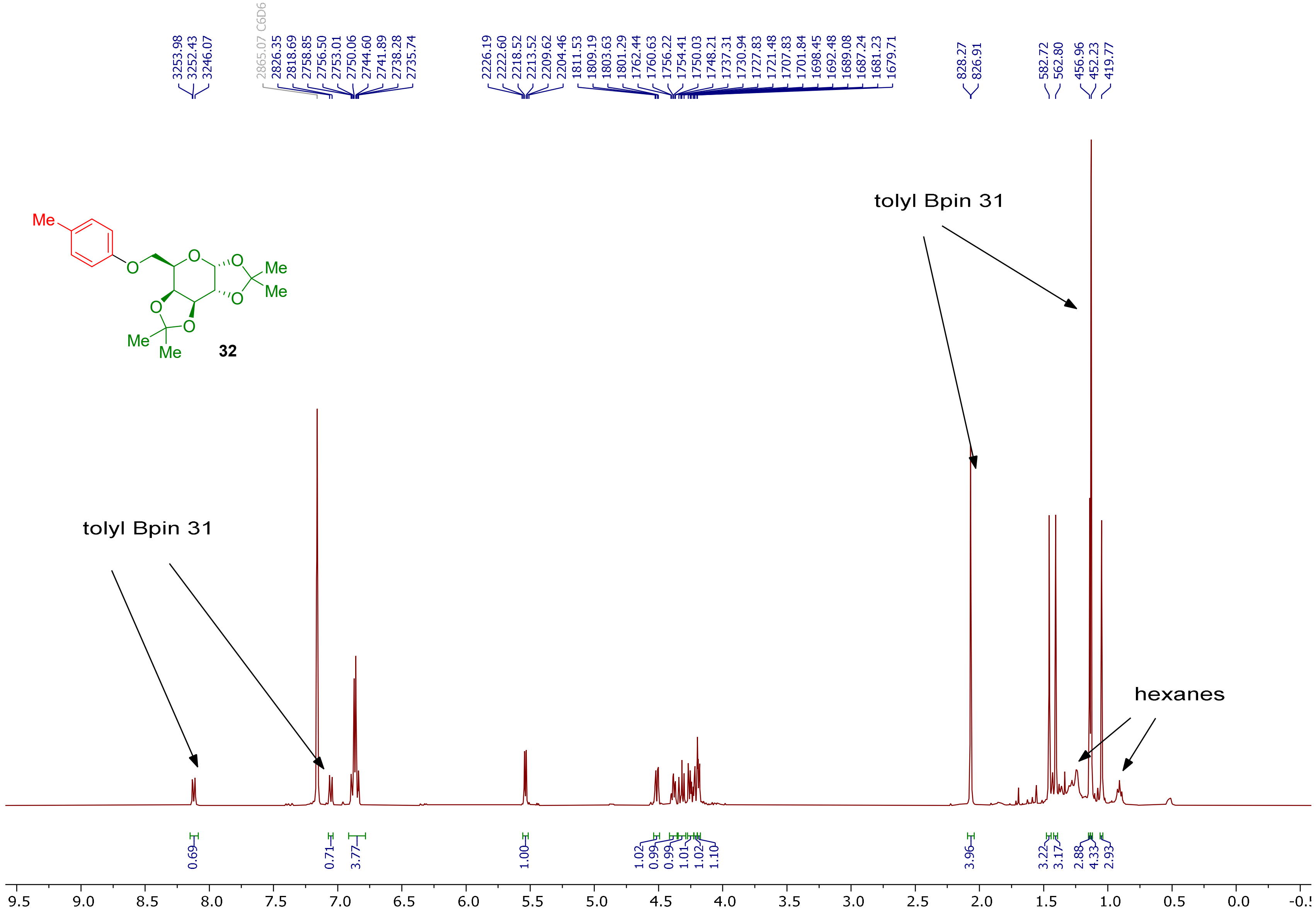

^{13}C{^{1}H} NMR spectrum of compound **32** (101 MHz, C_6D_6)

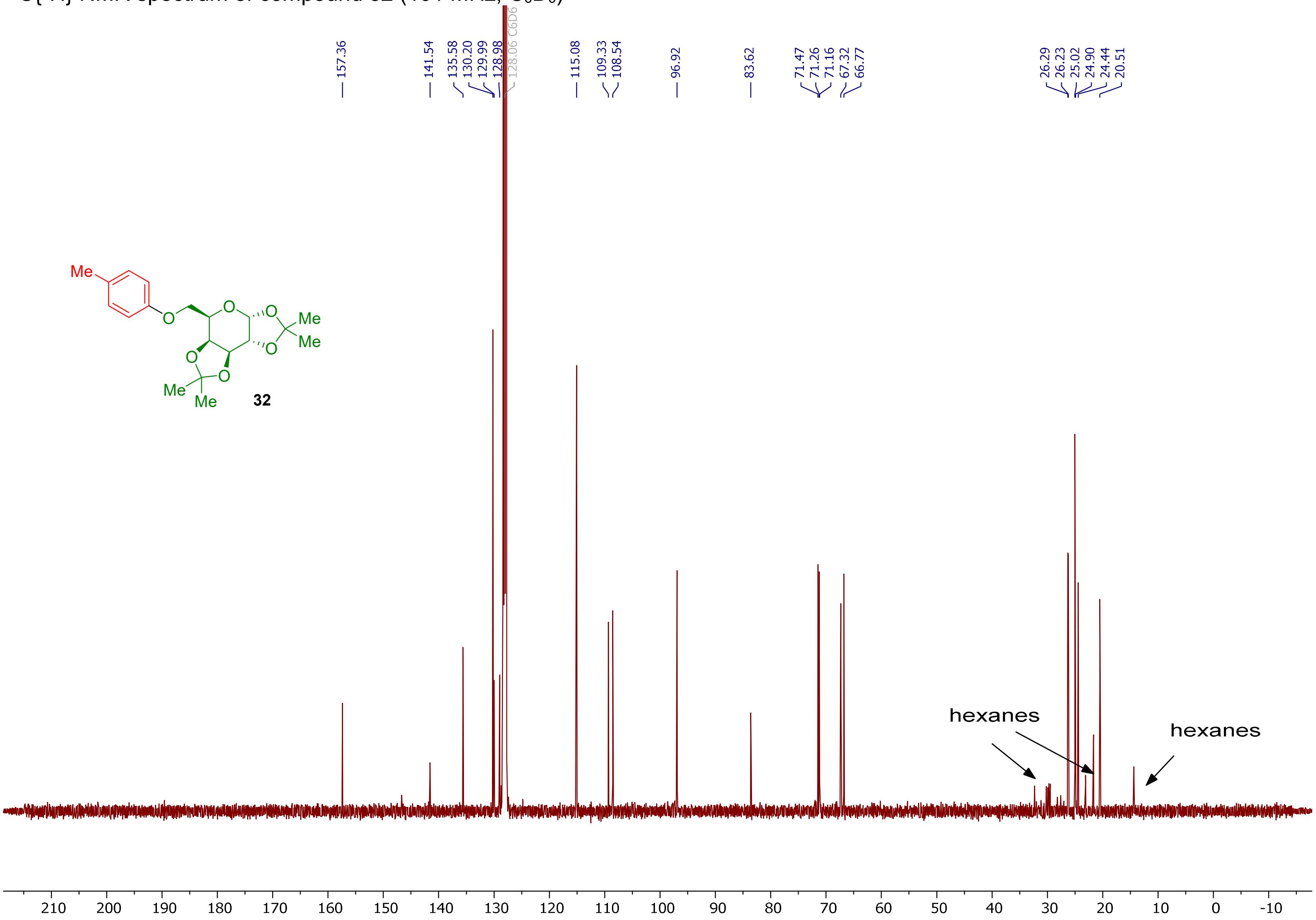

HSQC NMR spectrum of compound **32** (400 MHz, C_6D_6)

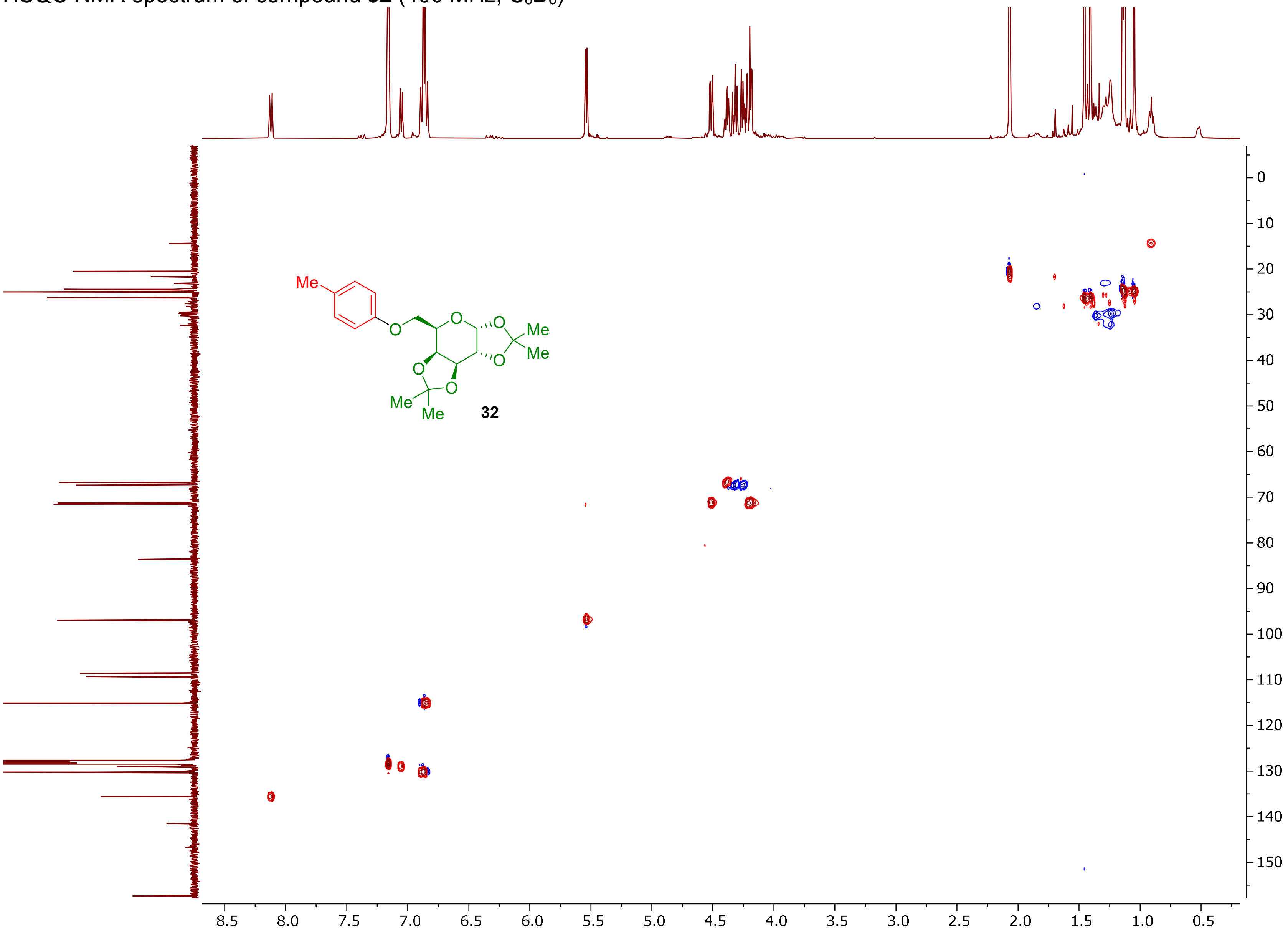

HMBC NMR spectrum of compound **32** (400 MHz, C_6D_6)

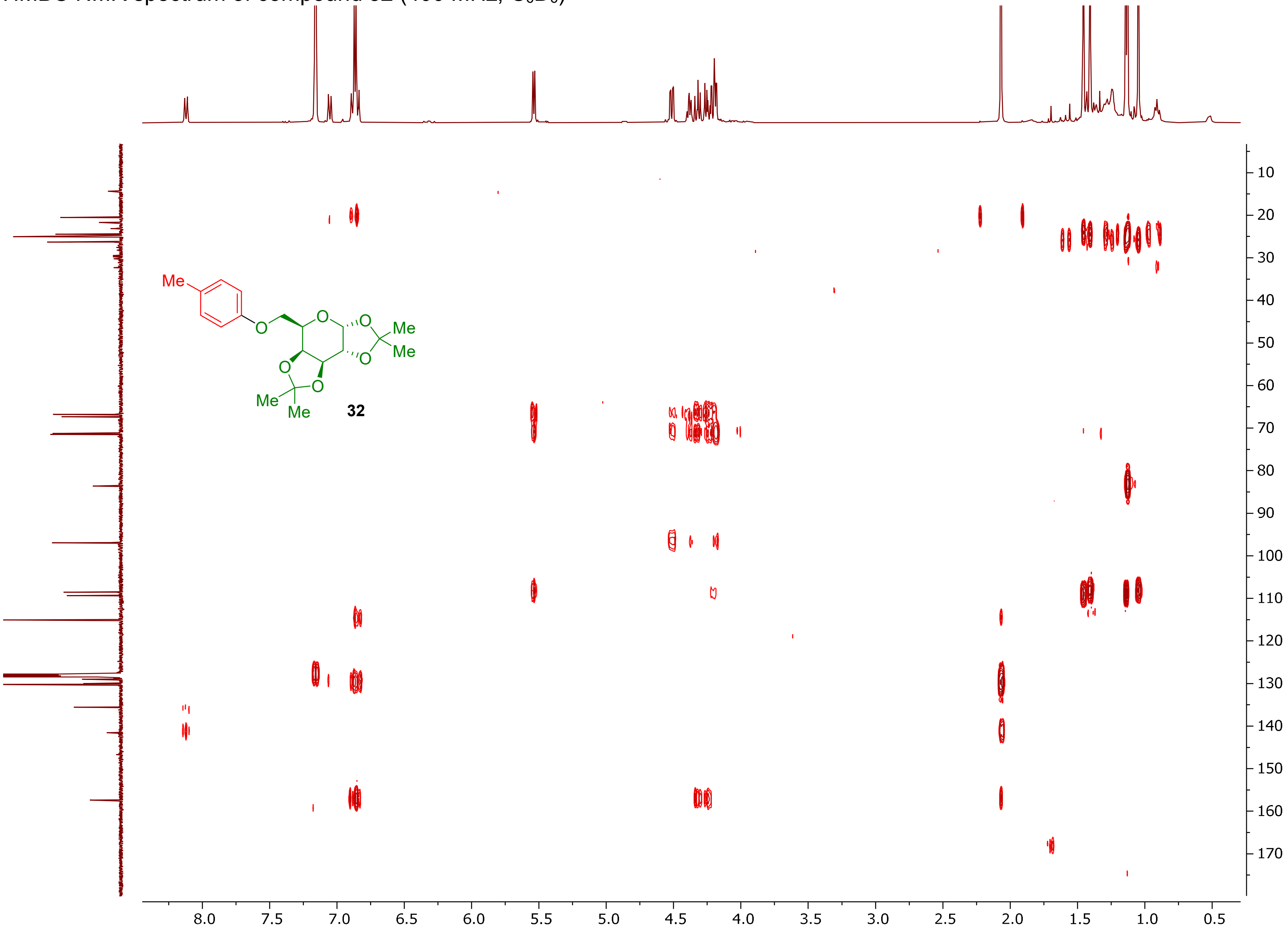

Supplement: Supplementary file 1 [file ol5c01966_si_001.pdf]
